# The Interplay Between Visual Traits and Forest in Bumblebee Communities Across Sweden

**DOI:** 10.1002/ece3.70635

**Published:** 2024-12-23

**Authors:** Pierre Tichit, Liam Kendall, Peter Olsson, Gavin Taylor, Christoph Rau, Paul Caplat, Henrik G. Smith, Emily Baird

**Affiliations:** ^1^ Department of Biology Lund University Lund Sweden; ^2^ Department of Zoology Stockholm University Stockholm Sweden; ^3^ Department of Wildlife, Fish and Environmental Studies Swedish University of Agricultural Sciences Umeå Sweden; ^4^ Centre for Environmental and Climate Science Lund University Lund Sweden; ^5^ Institute for Globally Distributed Open Research and Education (IGDORE) São Carlos Brazil; ^6^ Diamond Light Source Oxfordshire UK; ^7^ School of Biological Sciences Queen's University Belfast Belfast UK

**Keywords:** animal senses, community assembly, compound eyes, functional ecology, X‐ray microtomography

## Abstract

Understanding how ecological communities assemble in relation to natural and human‐induced environmental changes is critical, particularly for communities of pollinators that deliver essential ecosystem services. Despite widespread attention to interactions between functional traits and community responses to environmental changes, the importance of sensory traits has received little attention. To address this, we asked whether visual traits of bumblebee communities varied at large geographical scales along a habitat gradient of increased tree cover. Because trees generate challenging light conditions for flying insects, in particular a reduced light intensity, we hypothesised that differences in tree cover would correlate with shifts in the visual and taxonomical composition of bumblebee communities. We quantified 11 visual traits across 36 specimens from 20 species of bumblebees using micro‐CT and optical modelling of compound eyes and ocelli, and investigated how these traits scale with body size. Using an inventory of bumblebee communities across Sweden and our visual trait dataset, we then explored how visual traits (both absolute and relative to body size) differed in relation to tree cover. We found positive shifts of the community weighted means of visual traits along the increasingly forested habitat gradient (facet diameter, inter‐ommatidial angle, eye parameter of the compound eye and alignment of the three ocelli) that were consistent regardless of body size, while other traits decreased when more forest was present in the landscape (facet number). These functional patterns were associated with differences in the abundance of six common species that likely explains the community‐wide shift of visual traits along the habitat gradient. Our study demonstrates the interaction between vision, habitat and community assembly in bumblebees, while highlighting a promising research topic at the interface between sensory biology and landscape ecology.

## Graphical abstract

Do visual traits of bumblebee communities vary at large scales with increasing tree cover? We found shifts of visual traits along forested habitat gradient, suggesting that sensory properties not only of species, but also of whole communities interact with the optical constraints of their habitat niche.

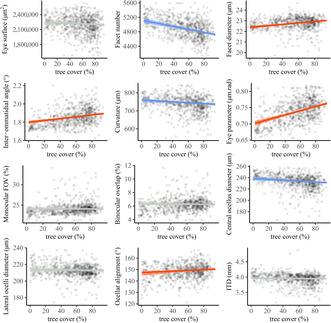

## 1 Introduction

Recent alarming declines and community shifts (van Klink et al. 2020; Warren et al. 2021) highlight the necessity for a better understanding of how environmental changes affect the assembly of arthropod communities. Functional trait analyses are a powerful tool for revealing how arthropod communities are assembled in relation to the environment (Wong, Guénard, and Lewis 2019). Traits that can be measured across individual organisms all have the potential to affect their fitness in relation to environmental conditions, including biotic interactions and can thus be considered functional (Sobral 2021). Since functional traits are often characteristic of species, environmental filtering of functional traits in relation to environmental gradients generated by natural or human processes also shapes the taxonomical composition of communities (Wong, Guénard, and Lewis 2019). This, in turn, facilitates the task of suggesting appropriate conservation measures in the face of biodiversity decline (Seibold et al. 2019; Wong, Guénard, and Lewis 2019).

Previous studies investigating the relationship between functional traits and the assembly of arthropod communities have considered a range of ecologically relevant morphological, feeding, life history, physiological and behavioural traits (Moretti et al. 2017). However, these studies rarely integrate sensory traits (Salmon et al. 2014; Winck et al. 2017), which is surprising given that adaptive behaviours are primarily driven by information from sensory systems (Dangles et al. 2009). In fact, sensory traits might be more informative than widely used traits that are very multifactorial (e.g., body size) to provide a mechanistic understanding of how communities assemble and persist. For many arthropods, vision plays a major role in determining how species interact with their environments (Cronin et al. 2014). In the present study, we explore the novel idea that differences in the composition of arthropod communities along a habitat gradient are associated with shifts of visual traits.

The ecological niche of a population or species generates optical constraints that affects visual systems (Scales and Butler 2016; Streinzer and Spaethe 2014; Warrant 2008). This is particularly true for the compound eyes of arthropods that comprise several thousands of optical units called ommatidia. The spacing and size of ommatidia determine their capacity to maintain spatial acuity (or resolution) while capturing enough photon and cannot simultaneously increase without reaching unsustainable eye sizes (Snyder 1979). The eyes of arthropods must therefore find a balance of visual traits that matches the optical requirements of their ecological niche (Warrant 1999). For compound eyes, the trade‐off between light sensitivity and spatial resolution is well expressed by the eye parameter (the product of the angular spacing and diameter of ommatidia on a compound eye, [Cronin et al. 2014; Land 1997]). Niches with a high degree of habitat closeness such as forests are most prominently characterised by a scarcity of light relative to their open counterparts (Feller et al. 2020; Taylor et al. 2015), as well as the frequent occlusion of the horizon by vegetation and a shift in the light spectrum (Endler 1993; Nilsson, Smolka, and Bok 2022). Environmental filtering should thus lead the visual properties of not only arthropod species, but also of whole communities to differ between forested and open habitats, but this idea has rarely been tested.

Bumblebees (*Bombus*) are an important focus for conservation due to the major contribution they make to the pollination of wild and domesticated plants (Ollerton 2017) and their alarming decline (Powney et al. 2019; Rollin et al. 2020; Soroye, Newbold, and Kerr 2020). Bumblebees typically live in open or semi‐open habitats such as pastures, meadows, shrublands and forest edges (Goulson 2010), but significant interspecific variations in habitat association exist (Herbertsson et al. 2021; Lundberg and Ranta 1980; Svensson et al. 2000). For example, the early bumblebee ( *Bombus pratorum* ) often forages in forest understories while the buff‐tailed bumblebee ( *Bombus terrestris* ) is most often found in bright open grasslands (Bartholomée et al. 2023; Reinig 1972). Bumblebee visual systems comprise three simple eyes—known as ocelli—and a pair of apposition compound eyes. Information from these eyes is necessary to accomplish essential behaviours: flight control (Dyhr and Higgins 2010; Linander, Dacke, and Baird 2015), flower detection (Goulson 2010), navigation (Mandal 2018) and mating (Paxton 2005). However, the link between the eyes and the occurrence in diverse habitats of these visually‐driven insects has received little attention to date.

To our knowledge, few studies have investigated how variations in light properties due to differences in vegetation cover relate to the taxonomical composition of communities (Gossner 2009; Théry 2001), and only one has explicitly investigated if this relationship extends to their functional composition (Bartholomée et al. 2023). In this study, communities of bumblebees were monitored in two boreal forest patches in Southern Sweden. The eye parameter of communities was shifted along a gradient of ambient light and this was likely linked to a differential exploitation by species of light microhabitat niches in variously shaded forest patches. The present study thoroughly explores the dataset of visual traits presented in Bartholomée et al. (2023) and scales it up by investigating the interplay between visual traits, tree cover and community assembly at a regional scale.

To investigate this, we quantified a wide range of visual traits in 36 museum and collection specimens across 20 bumblebee species in Sweden and investigated the effect of size on these traits. This is because body size influences the performance of organisms across biological functions, including visual abilities and varies within *Bombus* species (Jander and Jander 2002; Kapustjanskij et al. 2007; Streinzer, Huber, and Spaethe 2016), such that it may correlate with shifts in community composition (Theodorou et al. 2021). We then applied these visual traits to an existing inventory of bumblebee communities in grasslands across Sweden to explore interactions between visual traits of communities and tree cover (the proportion of forest in the landscape).

## 2 Material and Methods

### 2.1 Sample Collection

The need for extensive community monitoring data made it impossible to measure visual traits directly on inventoried specimens. Instead, bumblebee workers were collected between 2017 and 2019 in the vicinity of Lund (Scania County, Sweden) and around Abisko Scientific Research Station (Norrbotten County, Sweden) and promptly anesthetised with carbon dioxide for dissection. Additionally, dried specimens were obtained from the entomological collections of the biological museum at Lund University (Sweden).

### 2.2 Sample Preparation

The inter‐tegular distance (ITD)—a proxy of body size—was measured using callipers (Cane 1987). Compound eyes, ocelli and heads of bumblebees were prepared following a standard protocol to allow micro‐CT imaging (see Appendix S1 for further details).

### 2.3 Sample Scanning

To accurately measure visual traits on bumblebee ocelli and compound eyes, high‐resolution images of the whole optical structures with three‐dimensional X‐ray micro‐CT were obtained at the Diamond‐Manchester Imaging Beamline I13‐2 (Pešić et al. 2013; Rau et al. 2011) at the Diamond Light Source, UK (beamtime numbers: MT13848, MT16052, MT17632‐1, MT20385). The scanning procedure was the same as in Taylor et al. (2019), as were the data for *B. terrestris* . Small compound eyes and heads were imaged using ×4 total magnification (1.6 μm effective pixel size), while large samples were imaged with ×2 total magnification (2.6 μm effective pixel size).

### 2.4 Volumetric and Computational Analysis

Volumetric analyses were performed in Amira (FEI, Hillsboro, USA), where images were resampled at coarser resolution (pixel size = 4–5 μm). With a few exceptions, we followed the same volumetric analysis procedure as in Taylor et al. (2019) (Appendix S1), which involved three main steps: labelling of eye and head features, measurement of facet dimensions, registration of the eye and head in 3D space.

### 2.5 Calculating Visual Traits

**FIGURE 1:**
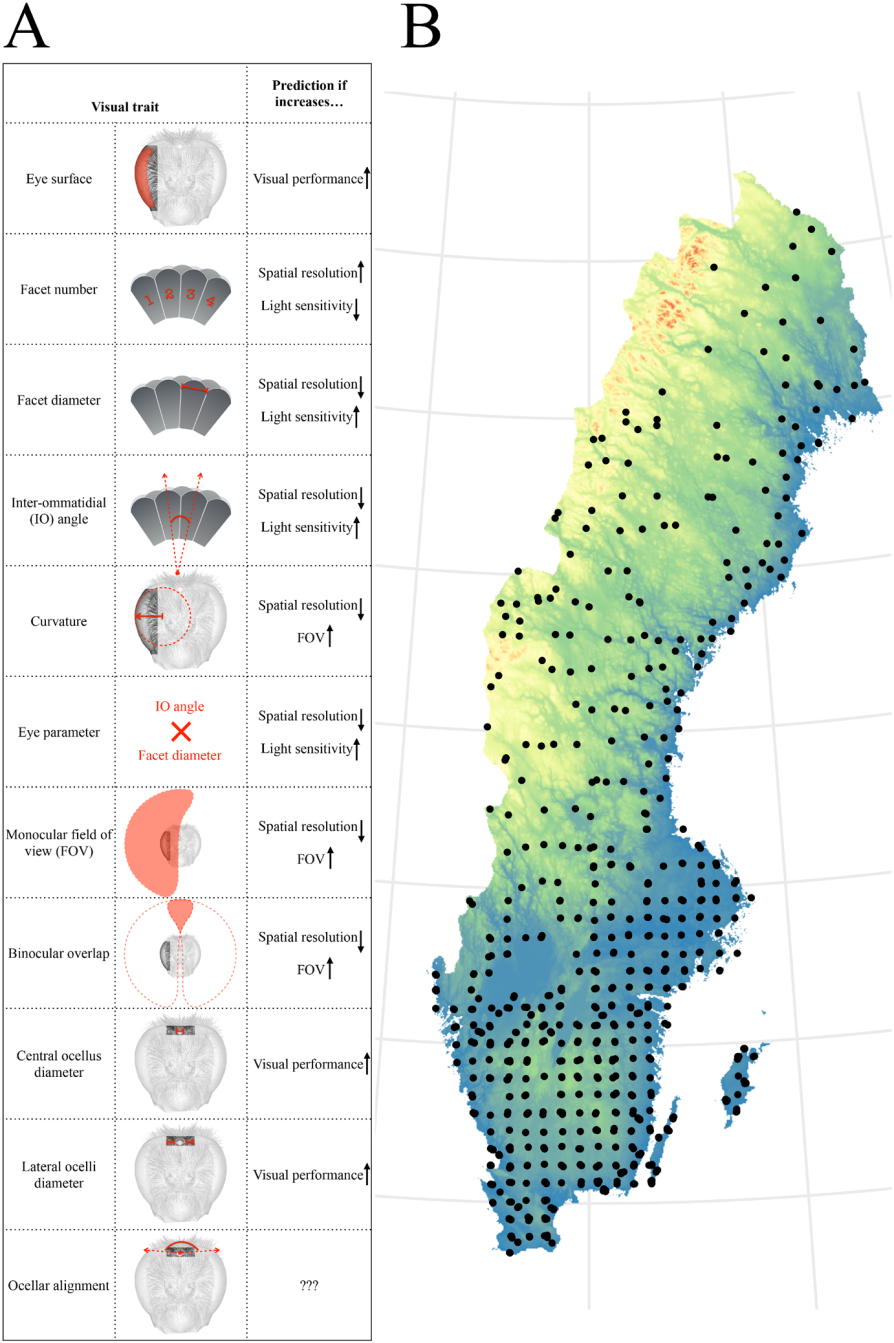
Visual traits and community inventories. Schematic representation of the 11 visual traits quantified in this study (A). The last column indicates the theoretical effect of an increase in the visual trait for a bee, all other parameters remaining equal. Distribution of the 812 inventoried bumblebee communities on a topological map of Sweden (B).

For each specimen, 11 compound eye traits were calculated (Figure 1A), including the compound eye surface (the area of the outer cornea of the eye in μm^2^), the number of facets, the extent of the monocular field of view and of the binocular overlap (percentage of the world seen by one or both eyes respectively). Local compound eye properties that vary topologically over the field of view were averaged across the eye to obtain a visual trait for each specimen. These visual traits are: facet diameter (in μm), radius of curvature of the eye (in μm), corneal inter‐ommatidial (IO) angle (in°) and eye parameter (the local product of IO angle and facet diameter, in μm rad). Three ocelli traits were calculated (Figure 1A, Appendix S1): the central and lateral ocellar diameters (in μm) and the alignment angle between the three ocelli (in°).

### 2.6 Repeatability

The difficulty of optical measurements implied that we were able to estimate visual traits for up to a handful of workers per species. This could question the representativeness of visual traits estimates; we therefore assessed repeatability for six species where we had measured more than one individual. Repeatability was the ratio of trait variance explained by interspecific differences from a general linear mixed‐effects model with body size as a fixed effect and species as a grouping factor calculated with package *rptR* (Stoffel, Nakagawa, and Schielzeth 2017).

### 2.7 Visual Traits and Body Size

To take into account the link between body size and visual traits, we modelled the effect of ITD on each visual trait averaged per species using generalised linear models with the R package *brms* based on *Stan* (Bürkner 2017; Stan Development Team 2024). We run two version of these allometric models with and without phylogenetic control. See Appendix S1 for more details about modelling procedure. Visual traits and ITD were log transformed. Traces of the sampling behaviour of each predictor and comparisons of modelled and observed data were computed to evaluate the quality of the models. The residuals of these models were back transformed into trait space to obtain estimate of visual traits relative to body size (Appendix S1).

### 2.8 Inventories of Bumblebee Communities

We used freely available inventories of bumblebees in Swedish meadows and pastures (https://Landskap.Slu.Se/, 2024). The program was launched in 2006 by the Swedish Board of Agriculture (Jordbruksverket) and the Swedish Environmental Agency (Naturvårdsverket) as part of the National Inventory of Landscapes in Sweden (NILS). The Abundance of 33 species was monitored every 5 years from 2007 to 2020 during field transects at 812 sites located across Sweden (Figure 1B; Sandring 2023). The procedure is described in more details in Appendix S1.

### 2.9 Ecological Indicators

During inventories of bumblebee communities, the proportion of the surface covered by nectar bearing flowers within a 4 m wide corridor either side of the transect was estimated and is hereafter referred as floral resource (in ‰; Sandring 2023). The percentage of the landscape covered by trees within a 2 km radius centred around the centroid of each grassland was computed using the land cover map of Sweden in the National Land Cover (NMD) Database (Swedish Environmental Protection Agency (Naturvårdsverket) 2018). We chose a radius of 2 km because the mean foraging distance of bumblebees from their nest is around 500 m and rarely exceeds a few km, although these may differ both between species and landscape types (Crowther et al. 2019; Kendall et al. 2022; Redhead et al. 2016; Westphal, Steffan‐Dewenter, and Tscharntke 2006).

### 2.10 Community Weighted Means of Visual Traits

Visual traits (both absolute and relative to body size) were averaged per species (Table S1). Visual traits could not be obtained for 4 out of 24 inventoried species (17%), although this corresponded only to 7% percent of specimens observed in the field, which mostly represented rare species (Figure S1). Missing traits for rare species were imputed using package *mice* (Penone et al. 2014) by resampling 30 times from the pool of species with available visual traits. Communities sampled across several years were aggregated since the present study does not investigate how time may interact with assembly of visual communities. To calculate functional metrics, sites with at least one specimen from the inventory across Sweden were used in the analysis. To characterise shifts in visual traits across communities, we calculated the community weighted means (CWM), that is the average of a trait across the bumblebee species recorded at each sampled site weighted by their observed abundance.

### 2.11 Effects of Tree Cover on Visual Traits Across Communities

To study the interplay between tree cover and visual traits at the community‐level, we used generalised linear models using Bayesian inference with the R package *brms* based on *Stan* (Bürkner 2017; Stan Development Team 2024). The CWM of visual traits (both absolute and relative to body size) were modelled as a function of the scaled tree cover (the tested predictor) and of scaled covariates to disentangle the effects of tree cover from geophysical, ecological and methodological factors: the logarithm of floral resource, latitude, longitude, elevation and the simple interaction of latitude and longitude. See Appendix S1 for more details about modelling procedure. Traces of the sampling behaviour of each predictor and comparisons of modelled and observed data were computed to evaluate the quality of the models.

### 2.12 Underlying Shifts in Community Composition

We used the same modelling procedure as above to investigate the effects of tree cover on each of the 10 most abundant species, except that the abundance (response variable) was modelled with a hurdle Poisson distribution.

## 3 Results and Discussion

### 3.1 Visual Traits

We measured 11 visual traits across 20 species of bumblebees (*n* = 36 female workers, Figure 1A, Table S1). On average, compound eyes consisted of 5000 ± 696 ommatidia with a facet diameter of 23.0 ± 1.6 μm, an IO angle of 1.79° ± 0.15° and an eye parameter of 0.72 ± 0.06 μm rad (Table S2). The average eye surface, facet number, facet diameter and IO angle were similar to those previously measured with different methods (Kapustjanskij et al. 2007; Spaethe 2003; Streinzer and Spaethe 2014), indicating that our novel micro‐CT based method provides reliable measurements of visual traits. The eye parameter *p* is a key trait that reflects the typical light conditions in which compound eyes operate (Snyder 1979). The average *p* across our dataset of 20 *Bombus* species concurred with the one of diurnal species in other bee genera (*p*_*Apis*_ = 0.7 μm rad, *p*_*Xylocopa*_ = 0.5–0.8 μm rad, *p*_*Tetragonula*_ = 0.9 μm rad, calculated from Jezeera et al. 2022; Somanathan et al. 2009; Taylor et al. 2019) but was lower than in a nocturnal bee (*p*_*Megalopta*_ = 1.2 μm rad, calculated from Warrant et al. 2004), confirming that bumblebee compound eyes are matched to the broad ecological requirements of an insect flying in daylight.

The difficulty of accurately measuring visual traits on a high number of specimens prevented us from including more than a handful of individuals per species, often only one. However, when comparing compound eye traits of six species with more than one replicate (there were no replicates of ocellar traits), we found that the majority of the variability was linked to interspecific differences (repeatability > 0.5, Table S3). This suggests that visual traits measured on a limited number of bees provide a sufficient representation of the true visual properties of species, provided that the effect of body size is taken into account.

### 3.2 Visual Traits and Body Size

**FIGURE 2:**
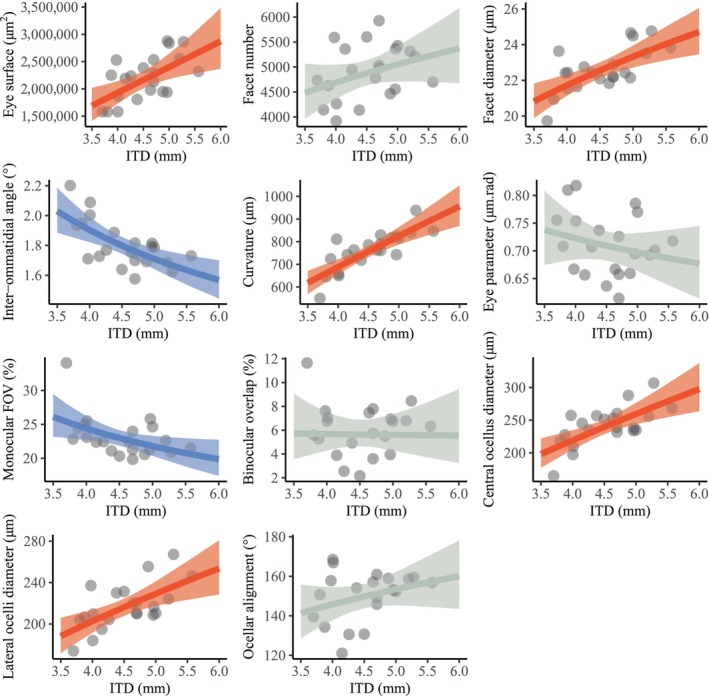
Relationships between body size (inter‐tegular distance—ITD) and 11 visual traits in Swedish true bumblebees (grey points, *n* = 21). Dark lines are the estimated effects modelled with Bayesian inference and shaded areas represent the Bayesian 95% credible intervals. Significantly positive and negative effects are drawn in red and blue respectively, while non‐significant relationships are in grey.

As expected, many visual traits scaled with the body size of workers across bumblebee species (Figure 2, Table S4). The eye surface, facet diameter, curvature, central ocellus diameter, lateral ocelli diameter significantly increased, while the IO angle and monocular FOV decreased with body size. These trends are similar to previously reported relationships between traits and eye size in *B. terretris* and across 11 species of bumblebees (Streinzer and Spaethe 2014; Taylor et al. 2019). Differences in visual traits were weakly constrained by phylogenetic proximity in bumblebees (Figure S2), suggesting that phylogenetic constraint is not a significant driver of the diversification of compound eyes and ocelli.

### 3.3 Effects of Tree Cover on Visual Traits Across Communities

**FIGURE 3:**
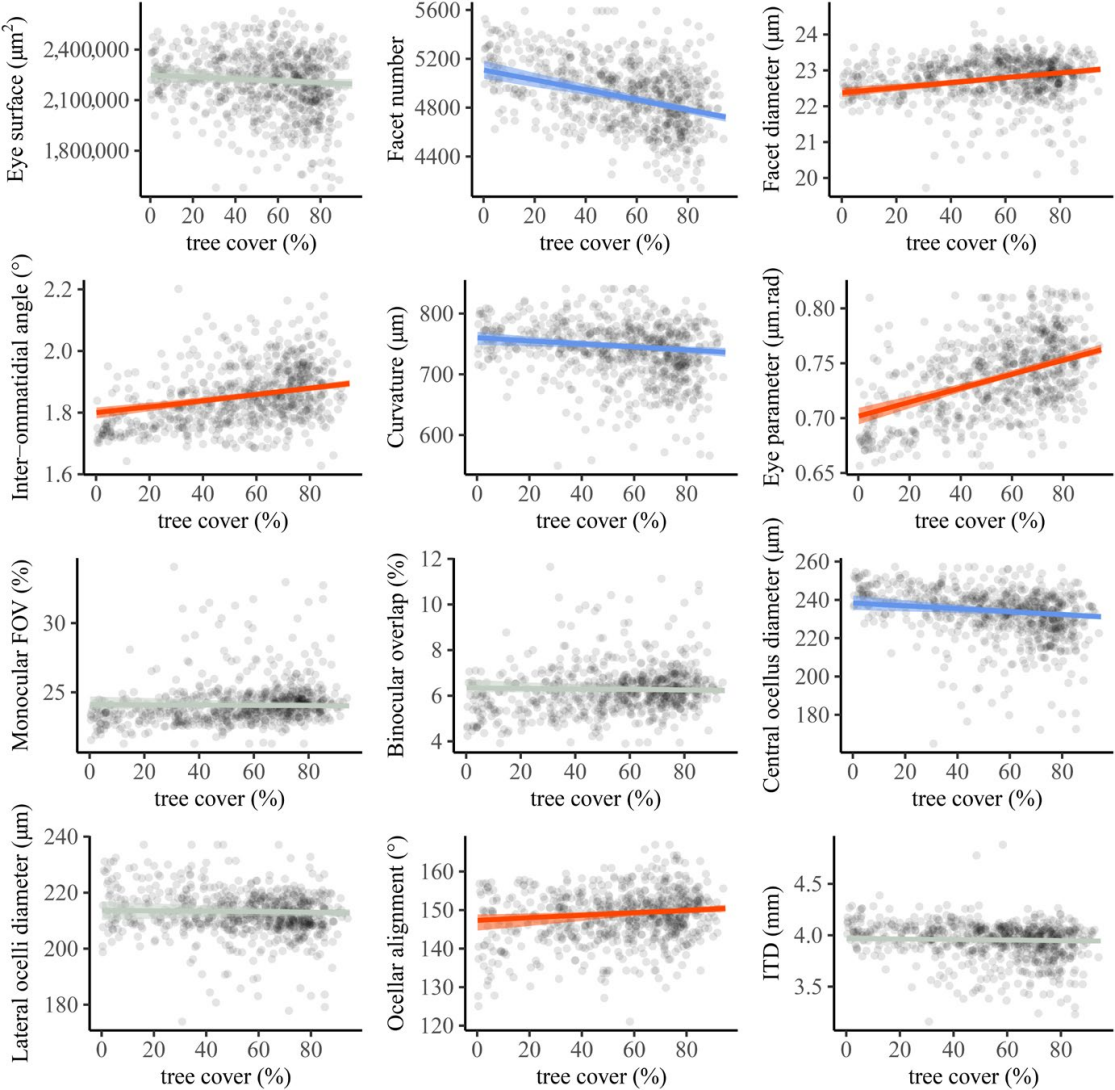
Effect of tree cover on the community weighted means of eleven visual traits and of ITD in bumblebee communities within grasslands across Sweden (*n* = 812). Grey circles represent the original data. Dark lines are the estimated effects of tree cover modelled with Bayesian inference and shaded areas represent the Bayesian 95% credible intervals. Significantly positive and negative effects are drawn in red and blue respectively, while non‐significant relationships are in grey.

There were significant relationships between the percentage of tree cover within a 2 km radius and the community weighted means (CWM) of visual traits in grasslands across Sweden (Figure 3, Table S5). With every 10% increase of tree cover, the facet diameter, IO angle, eye parameter and ocellar alignment increased by 0.30%, 0.56%, 0.89% and 0.21% respectively, while the facet number, curvature and central ocellus diameter decreased by 0.84%, 0.32%, 0.33%. Body size and the other visual traits did not significantly vary with tree cover. These relationships were consistent for the facet diameter, IO angle, eye parameter, facet number and ocellar alignment when calculated relative to body size, but contrasting relationships were found for the curvature, field of views and ocellar diameters (Figure S3, Table S6).

To our knowledge, this is the first time that community‐wide shifts of visual traits are found across a large geographic scale. Differences are small: less than 10% from an afforested to a fully forested landscape, but likely not without functional implications for bumblebee communities (Figure 1A). Communities in grassland surrounded by more trees have compound eyes with fewer but bigger facets than their open‐habitat counterparts, enhancing light capture. The angle between each ommatidium is wider, which would also improve light sensitivity (Snyder 1979). However, because sampling units are fewer and farther apart, these modifications come with the cost of reduced spatial resolution. As an indicator of this trade‐off, the eye parameter increased with tree cover, as is typically the case in eyes that operate under dim conditions. A change from about 0.7 to 0.8 μm rad from afforested to fully forested habitats, while less dramatic than differences between day and night active insects (the latest typically > 1 μm rad, [Snyder 1979; Warrant et al. 2004]), likely indicates that visual abilities of communities in wooded habitats match the subtle decrease of light intensity in forests. Using in part this dataset of visual traits, similar effects on the eye parameter were found at a smaller scale in bumblebee communities distributed along light intensity gradients in a boreal forest (Bartholomée et al. 2023). Overall, the community‐wide differences in compound eye traits in our study are likely related to the altered light properties of wooded habitats, in particular to the reduced light intensity (Nilsson, Smolka, and Bok 2022).

We also found that the three ocelli became consistently more aligned along the habitat gradient. Several mutually compatible functions have been suggested for bee ocelli, including head and body stabilisation (Parsons, Krapp, and Laughlin 2006; Wilson 1978) and compass‐based navigation (Wellington 1974). It is possible that the differences of ocellar alignment in forested habitats reflect a change in the position or type of navigational cues that the ocelli perceive. However, too little is known about the visual role of the ocellar alignment to allow a robust functional interpretation of these results, highlighting the need for more research to link structure and function in arthropod ocelli.

### 3.4 Underlying Shifts in Community Composition

**FIGURE 4:**
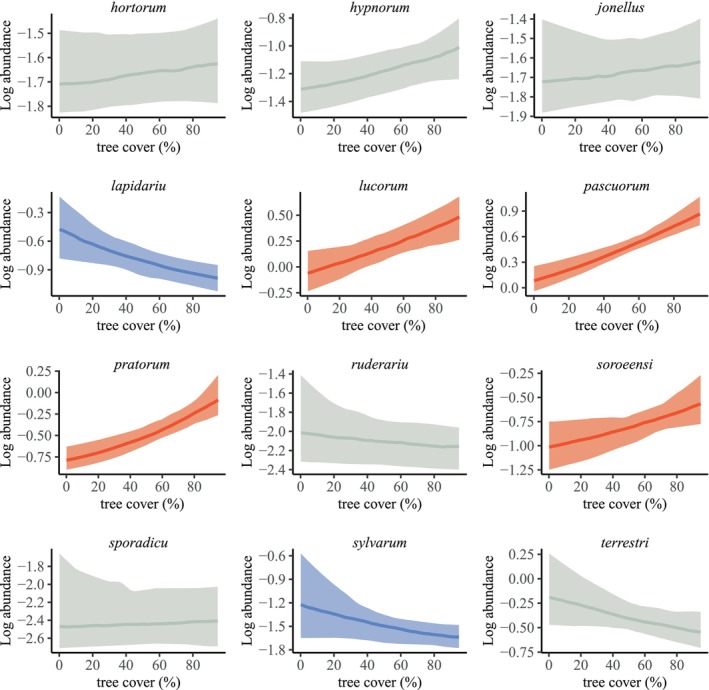
Effect of tree cover on the abundance of the twelve most common species in communities within grasslands across Sweden (*n* = 812). Dark lines are the estimated effects of tree cover on a log scale modelled with Bayesian inference and shaded areas represent the Bayesian 95% credible intervals. Significantly positive and negative effects are drawn in red and blue respectively, while non‐significant relationships are in grey.

Grassland communities surrounded by more trees did not include more species than those in open habitats (Figure S4, Table S4) but their composition changed. In particular, four common species— *B. lucorum* (including cryptic species), *B. pascuorum* , *B. pratorum* and *B. soroeensis* —became more abundant with increasing tree cover (Figure 4, Table S4). *B. lucorum* has a known affinity for forested areas (Geue and Thomassen 2020; Herbertsson et al. 2021; Svensson et al. 2000), while *B. pascuorum* and *B. pratorum* are habitat generalists with a high relative abundance around and within forests (Reinig 1972; Svensson et al. 2000). The habitat associations of *B. soroeensis* concurred with previous studies (Bartholomée et al. 2023; Reinig 1972). In parallel, we found that *B. lapidarius* and *B. sylvarum* became less abundant with increasing tree cover, which agrees with previous work (Reinig 1972; Svensson et al. 2000).

The dominant species, *B. pratorum* and *B. pascuorum* (when averaging both sub‐species), had the two highest eye parameters among the 20 bumblebee species measured (Table S1). These two species are increasingly abundant in wooded habitat and may thus be largely responsible for the observed community‐wide shift in eye parameter. In a patch of boreal forest (Bartholomée et al. 2023), gynes of *B. pratorum* preferentially foraged under low light intensities compared to other species, supporting the hypothesis of an association between visual performance and habitat use. Although other rarer species are probably associated with forest (e.g., *B. consobrinus* ) or open‐landscape (e.g., *B. muscuorum*), it is likely that the six species presented above account for most of the community‐wide patterns of visual traits in this study.

### 3.5 Vision and the Importance of Forest for Bumblebee Communities

Bees occurring in grassland or fields are affected by the presence of forest in the wider landscape (Bailey et al. 2014). Bumblebees depend on forests for nesting and hibernating sites, as well as for mate‐searching and food gathering (Kreyer et al. 2004; Mola et al. 2021; Svensson et al. 2000). At the very least, these behaviours would require bees to visually navigate to the edge of a wood patch. Foraging inside a forest would pose an added constraint on visual performance, as bees would need to locate flowers in dimmer light and shaded areas (Bartholomée et al. 2023). Species such as *B. pascuorum* and *B. pratorum* are known to utilise flower resources in forest understory (Andresen 2019; Bartholomée et al. 2023; Kreyer et al. 2004), which is consistent with the higher eye parameter measured for these species.

In our study, the visual and taxonomical composition of bumblebee communities in grasslands changed when they were surrounded by more trees. Forest may thus benefit bumblebees by providing habitat to species that trade‐off vision in dim light over spatial resolution (e.g., *B. pratorum* ). These species are also found in open grasslands, even though they would in theory be less visually‐competitive there than open‐habitat species because of their reduced resolution. The fact that tree cover generates new optical niches that are exploited by distinct visual communities is in line with previous research (Sõber et al. 2020). Wooded areas within the foraging range of a community may give a competitive advantage to bees that are more adapted to the associated visual constraints than open‐habitat species.

### 3.6 Limitations

This work is among the first to integrate sensory—in this case visual—traits to investigate visual correlates of community assembly. It is exploratory in nature and provides opportunities for further study. However, it also has limitations. First, we measured visual traits in parallel with the community inventory, as it was not feasible to measure directly on a representative subset of more than 800 monitored communities at such a large geographic scale. Consequently, our approach did not enable us to take into account adaptations of visual traits to local conditions. Second, due to the difficulty of accurately measuring visual traits, our estimates were based on up to a handful of individuals per species. Hence, the representativeness of measures for species could be questioned, although we found that the repeatability of measurements was relatively high. Furthermore, we cannot separate intra‐ and interspecific scaling of visual traits with body size, and thus had to rely on the assumption that trait‐body size relationships are similar within and between species. We recommend developing methods that trade‐off accuracy and speed to quantify—ideally directly on specimens from community surveys—the full distribution of sensory traits for each species in the community across tens of replicates. Finally, we took into account multiple possible confounding factors in our analysis, but it is possible that landscape properties such as habitat fragmentation and agricultural intensity that may co‐vary with tree cover, are in turn associated with community shifts (Gérard et al. 2020; Persson et al. 2015).

## 4 Conclusion

Despite some caveats, this work increases our understanding of how sensory traits interplay with the assembly of arthropod communities at large scales and underlines how the presence of forest in the landscape may help to sustain diverse bumblebee communities. This is very valuable information for strengthening pollinator conservation programs. This study demonstrates at an unprecedented large scale that visual properties not only of species, but also of whole communities interact with the optical constraints of their habitat niche. It opens new avenues in functional ecology to disentangle the role of sensory traits in community assembly. Conversely, sensory trait‐based studies represent an opportunity for neurophysiologists, behavioural scientists and anatomists in sensory biology to investigate the ecological significance of their findings.

## Author Contributions

**Pierre Tichit:** conceptualization (lead), data curation (lead), formal analysis (lead), investigation (lead), methodology (lead), project administration (supporting), software (lead), supervision (supporting), validation (lead), visualization (lead), writing – original draft (lead), writing – review and editing (lead). **Liam Kendall:** formal analysis (supporting), investigation (supporting), methodology (supporting), software (supporting), supervision (supporting), validation (supporting), writing – review and editing (supporting). **Peter Olsson:** data curation (supporting), formal analysis (supporting), methodology (supporting), writing – review and editing (supporting). **Gavin Taylor:** investigation (supporting), methodology (supporting), software (supporting), supervision (supporting), writing – review and editing (supporting). **Christoph Rau:** methodology (supporting), writing – review and editing (supporting). **Paul Caplat:** conceptualization (supporting), funding acquisition (equal), methodology (supporting), supervision (supporting), writing – review and editing (supporting). **Henrik G. Smith:** conceptualization (supporting), formal analysis (supporting), funding acquisition (equal), methodology (supporting), supervision (supporting), validation (supporting), writing – review and editing (supporting). **Emily Baird:** conceptualization (supporting), data curation (supporting), formal analysis (supporting), funding acquisition (equal), methodology (supporting), project administration (lead), software (supporting), supervision (lead), validation (supporting), visualization (supporting), writing – original draft (supporting), writing – review and editing (supporting).

## Conflicts of Interest

The authors declare no conflicts of interest.

## Supporting information


Appendix S1.

Appendix S2.

Appendix S3.

## Data Availability

The full dataset of visual trait determined in this study is available as a Supporting Information. The inventories of bumblebees used in this study are freely available online (https://landskap.slu.se/, 2024). Graphs to assess the quality of Bayesian models are available as a Supporting Information.
