# Supplementary material for: The Interplay Between Visual Traits and Forest in Bumblebee Communities Across Sweden
Source: Ecol Evol. 2024 Dec 23;14(12):e70635. doi: 10.1002/ece3.70635 (PMC11664236; doi:10.1002/ece3.70635)
Supplement: Supplementary file 1 — Appendix S1. [file ECE3-14-e70635-s001.pdf]

Appendix for the article "The interplay between visual traits and forest in bumblebee communities across Sweden"

Text S1

Sample preparation.

The left compound eyes of freshly collected specimens were dissected, transferred to a fixative solution of paraformaldehyde and glutaraldehyde, stained with osmium tetroxide, dehydrated with a graded alcohol series, and embedded in epoxy resin as described in Taylor et al. (2019). In a handful of cases where the left compound eye was damaged, the right compound eye was instead used. Specimens preserved in ethanol were directly dehydrated with a graded alcohol series followed by critical point drying. Bumblebee heads were dissected, fixated, dehydrated with a graded alcohol series and critical point dried.

Sample scanning.

The average photon energy was approximately 22 keV, using the so called "pink beam", filtering lower high and low harmonics of the undulator beam.

Volumetric and computational analysis.

The original image stacks reconstructed from the scans were cropped and re-saved in 8-bit files in Drishti Paint (Limaye, 2012). In the present study, the inner layers of the compound eyes were not manually labelled. Instead, the entire eye and head volumes were segmented semi-automatically and the outer surface of the lens was labelled by manually drawing a geodesic path around the cornea. The labels and measurements obtained during volumetric analysis were imported to *MATLAB* (The MathWorks Inc., 2016) and analysed as in Taylor et al. (2019) with two differences: *(1)* the mirroring of a compound eye (either because it was a right instead of a left compound eye, or to obtain the binocular overlap on the field of view of the left compound eye) was performed by flipping the eye with respect to the plane of symmetry of the head and *(2)* the validation of calculation was less strict, as calculations were repeated until the number of simulated ommatidial axes was as close as possible to the facet number, but not necessarily with a 5 % margin.

Calculating visual traits.

Ocellar visual traits were measured on the head registered in world coordinates in Amira. One vertical, one horizontal and two diagonal segments were drawn on the surface of each of the three ocelli. The 3D positions of the segments were imported to *MATLAB* and were used to calculate visual traits. In a few cases where ocelli and compound eye traits were measured on different specimens, ocellar diameters were scaled to match the size of the head used to measure compound eye traits.

Visual trait allometry.

To control for phylogenetic relationships when fitting allometric models, we used a published high-resolution molecular phylogeny obtained from the analysis of nuclear and mitochondrial DNA sequences (Hines, 2008). The phylogeny was trimmed to a subset of 20 Swedish true bumblebee species for which visual traits were measured. The two subspecies of *B. pascuorum smithianus* and *B. pascuorum pallidofacies* were included and separated by an arbitrary small distance. The phylogeny was converted into a covariance matrix for statistical modelling.

Each of the eleven log transformed visual traits averaged per bumblebee species was modelled as a function of the log transformed ITD (simple allometric model). The procedure was repeated with the addition of phylogenetic covariance (phylogenetically controlled

allometric model). Response variables were drawn from a gaussian distribution. Flat uniform priors (over [-∞;+∞]) were used to model the effects of predictors, a weakly informative group-level factor was drawn from a Student's t-distribution (df = 3, mean μ = 0, s.d. = 2.5). Models were run using 4 chains for 5000 iterations (including 2500 to warm-up, which is sufficient given the high convergence speed of Bayesian algorithms in *brms* (Bürkner, 2017). Traces of the sampling behaviour of each predictor were scrutinised to verify that there was little autocorrelation between successive iterations and that the models converged towards reliable predictions. A posterior predictive check was used to compare modelled and observed data and thus evaluate the quality of the models. The significance of each effect being different from zero was assessed using Bayesian 95 % credible intervals.
To calculate traits relative to body size, the residual of the simple allometric models were back transformed from log space using the following formula: Observed trait – exp(fitted trait).

Inventories of bumblebee communities.
Study grasslands were located in squares (n = 631) systematically distributed across Sweden with an increasing size towards higher latitude to account for a decreasing agricultural activity (Sandring, 2023). Pollinator communities including bumblebees were inventoried every five years. The number of transects per site depended on the size of the grassland, there might be several transects per site, and thus a large variation in the cumulated length of transects (min = 25 m, mean = 1200 m, max = 10000 m). Transects were aligned from South to North or East to West and set up parallel to each other at a distance of more than 20 m to avoid resampling the same individuals. Data collection took place only in favourable weather conditions (> 17 °C, little to no wind, no rain). Participants recorded every bumblebee encountered within 5 m either side of the transect while walking at a constant speed of approximately 50 m per min. When necessary, specimens were captured and euthanised to allow identification in the laboratory. The cryptic species *B. cryptarum* and *B. magnus* were registered as *B. lucorum*.

Ecological indicators.
NMD provides maps of 25 land cover categories with a 10 m resolution. Tree cover (in %) was calculated by aggregating all forest layers in NMD (categories 111 to 128) divided by the surface of a 2 km disk.

Effects of tree cover on visual traits across communities.
Longitude and latitudes were projected using a coordinate system that preserves true distances (SWEREF 99 TM). Flat uniform priors (over [-∞;+∞]) were used to model the effects of predictors, a weakly informative group-level factor was drawn from a Student's t-distribution (df = 3, mean μ = 0, s.d. = 2.5). Models were run using 3 chains for 5000 iterations (including 2500 to warm-up, which is sufficient given the high convergence speed of Bayesian algorithms in *brms* (Bürkner, 2017)). Traces of the sampling behaviour of each predictor were scrutinised to verify that there was little autocorrelation between successive iterations and that the models converged towards reliable predictions. Posterior predictive checks were used to compare modelled and observed data and thus evaluate the quality of the models. The significance of each effect being different from zero was assessed using Bayesian 95 % credible intervals.

When not otherwise specified, values are given as mean ± s.d.

References to text S1.
Bürkner, P. C. (2017). brms: An R package for Bayesian multilevel models using Stan. *Journal of Statistical Software*, *80*(1). https://doi.org/10.18637/jss.v080.i01

Hines, H. M. (2008). Historical biogeography, divergence times, and diversification patterns of bumble bees (Hymenoptera: Apidae: *Bombus*). *Systematic Biology*, *57*(1), 58–75. https://doi.org/10.1080/10635150801898912

Limaye, A. (2012). Drishti: a volume exploration and presentation tool. *Developments in X-Ray Tomography VIII*, *8506*(October 2012), 85060X. https://doi.org/10.1117/12.935640

Penone, C., Davidson, A. D., Shoemaker, K. T., Di Marco, M., Rondinini, C., Brooks, T. M., Young, B. E., Graham, C. H., & Costa, G. C. (2014). Imputation of missing data in life-history trait datasets: which approach performs the best? *Methods in Ecology and Evolution*, *5*(9), 961–970. https://doi.org/10.1111/2041-210X.12232

Sandring, S. (2023). *Fältinstruktion för fjärilar och humlor i ängs- och betesmarker.*

The MathWorks Inc. (2016). *MATLAB version: 9.1.0 (R2016b)*. The MathWorks Inc.

Table S1. Dataset of visual traits and body size (ITD) for workers of 20 species (including two subspecies) of *Bombus* present in Sweden. These values were quantified using a combination of micro-CT imaging and optical modelling, and averaged over N replicates per species.

Species	N	ITD (mm)	Eye surface (μm^2)	Facet number	Facet diameter (μm)	Inter-ommatidial angle (°)	Curvature (μm)	Eye parameter (μm.rad)	Monocular FOV (%)	Binocular overlap (%)	Central ocellus diameter (μm)	Lateral ocelli diameter (μm)	Ocellar alignment (°)
B. alpinus	3	5.6	2319826	4701	23.8	1.73	847.2	0.72	21.5	6.3	268.2	246.6	156.8
B. balteatus	4	5.3	2862842	5217	24.8	1.62	938.6	0.70	20.9	8.5	306.8	267.2	159.5
B. cingulatus	2	4.0	1580979	3919	21.5	2.00	657.9	0.75	23.1	6.7	197.5	184.0	168.5
B. consobrinus	1	5.0	2839101	5425	24.5	1.79	816.5	0.77	24.6	6.7	235.0	210.4	152.3
B. hortorum	3	5.0	2876512	5365	24.7	1.81	823.8	0.79	25.8	6.9	238.1	216.7	152.7
B. hypnorum	1	4.7	2290451	5033	22.9	1.82	761.1	0.73	24.0	7.8	231.6	210.0	145.9
B. jonellus	1	3.7	1581194	4737	19.7	2.20	549.5	0.76	34.1	11.6	164.9	174.0	139.5
B. lapidarius	1	5.0	1937442	4555	22.1	1.79	742.7	0.69	21.3	4.0	233.9	208.7	153.3
B. lapponicus	1	4.4	1805178	4137	22.4	1.89	718.2	0.74	21.1	4.9	256.9	230.1	154.1
B. lucorum	1	5.2	2553696	5317	23.5	1.69	840.5	0.69	22.6	6.8	256.0	224.3	158.7
B. monticola	1	4.6	1977935	4780	21.8	1.75	763.2	0.67	22.5	7.5	252.9	219.6	157.1
B. muscorum	1	4.5	2383976	5602	22.1	1.64	785.9	0.64	20.3	2.2	251.5	231.4	130.7
B. pascuorum pallidofacies	3	3.9	2250633	4625	23.6	1.95	724.3	0.81	24.4	5.3	227.7	206.7	134.3
B. pascuorum smithianus	1	4.3	2234906	4944	22.8	1.77	763.6	0.71	22.5	2.6	235.4	204.3	130.6
B. pratorum	1	4.0	1862308	4264	22.4	2.09	649.9	0.82	25.5	7.1	209.8	209.7	166.9
B. pyrrhopygus	1	4.9	1947825	4464	22.4	1.69	817.3	0.66	20.6	5.5	287.9	255.4	159.0
B. soroeensis	1	3.8	1579786	4143	20.9	1.93	645.1	0.71	22.8	5.6	217.8	204.0	150.7
B. subterraneus	1	4.7	2533570	5927	22.2	1.58	829.9	0.61	19.8	3.6	260.3	210.3	149.0
B. sylvarum	1	4.2	2190375	5360	21.6	1.73	742.2	0.66	22.4	3.9	245.3	195.0	121.1
B. terrestris	6	4.0	2527738	5592	22.4	1.71	811.3	0.67	25.0	7.6	257.2	237.1	157.8
B. wurflenii	1	4.7	2134739	4984	22.2	1.70	790.0	0.66	21.3	5.7	239.0	220.8	160.9

Figure S1. Distribution of missing visual trait data across communities (n = 812) from zero (no specimen had measured visual traits) to 100 % (all specimens in the community had measured visual traits).

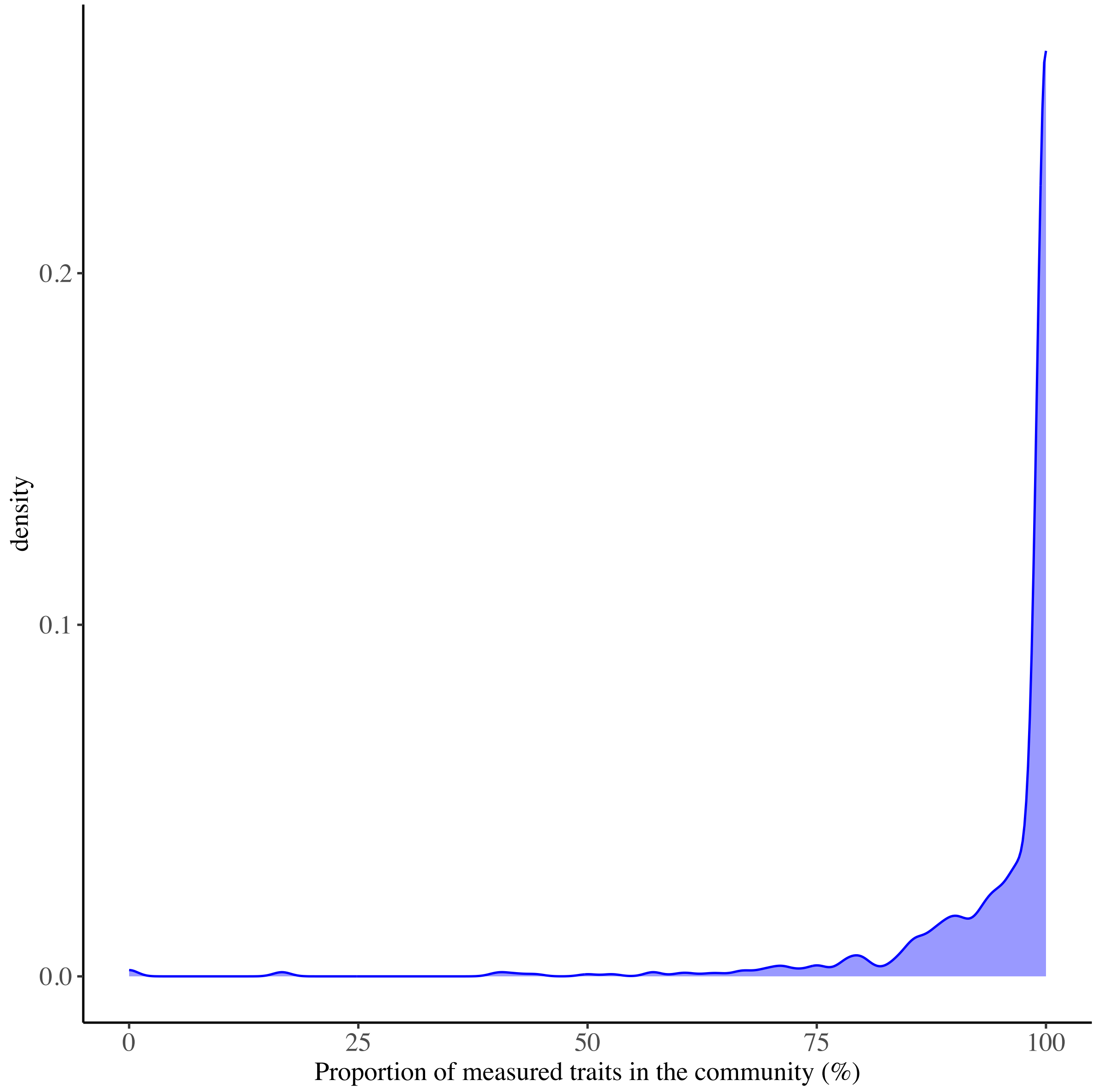

Table S2. Average of visual traits and body size (ITD) for workers of 20 species (including two subspecies) of *Bombus* present in Sweden. These values were quantified using a combination of micro-CT imaging and optical modelling, and averaged over 20 species.

	ITD (mm)	Eye surface (μm^2)	Facet number	Facet diameter (μm)	Inter-ommatidial angle (°)	Curvature (μm)	Eye parameter (μm.rad)	Monocular FOV (%)	Binocular overlap (%)	Central ocellus diameter (μm)	Lateral ocelli diameter (μm)	Ocellar alignment (°)
mean	4.5	2203381	4909	22.6	1.80	762.8	0.71	23.1	6.0	241.6	217.43	150.45
s.d.	0.5	407190	548	1.2	0.16	86.4	0.06	3.1	2.2	30.2	22.18	12.55

Table S3. Repeatability of visual trait measurements. Repeatability was the ratio of trait variance explained by interspecific differences from a general linear mixed-effects model with

body size as a fixed effect and species as a grouping factor. The model was performed on compound eye traits of six species for which replicates were available (n = 21 specimens).

	Repeatability
Eye surface	0.55
Facet diameter	0.65
Inter-ommatidial angle	0.85
Facet number	0.62
Eye parameter	0.80
Eye curvature	0.76
Monocular FOV	0.76
Binocular overlap	0.51

Table S4. Summary of the allometric scaling of visual traits with body size modelled with Bayesian inference without phylogenetic control (Estimate: estimated effect, l-95 % CI: lowest 95 % credible interval, u-95 % CI: upper 95 % credible interval). Significantly positive and negative effects scaling are highlighted in red and blue respectively. Visual traits and ITD were log transformed to account for non-isometric scaling, such that we modelled solutions to the equation: Trait = log b + a × log ITD.

		Intercept (log ***b***)	Scaling exponent (***a***)
Eye surface	Estimate	13.11	0.98
	l-95% CI	12.18	0.36
	u-95% CI	14.05	1.60
Facet diameter	Estimate	2.64	0.32
	l-95% CI	2.38	0.15
	u-95% CI	2.90	0.49
Inter-ommatidial angle	Estimate	1.30	-0.48
	l-95% CI	0.89	-0.74
	u-95% CI	1.71	-0.21
Facet number	Estimate	7.98	0.34
	l-95% CI	7.30	-0.11
	u-95% CI	8.65	0.79
Eye parameter	Estimate	-0.10	-0.16
	l-95% CI	-0.58	-0.47
	u-95% CI	0.37	0.15
Eye curvature	Estimate	5.41	0.81
	l-95% CI	4.95	0.52
	u-95% CI	5.85	1.11
Central ocellus diameter	Estimate	4.34	0.76
	l-95% CI	3.75	0.37

	u-95% CI	4.93	1.15
Lateral ocelli diameter	Estimate	4.54	0.55
	l-95% CI	4.06	0.23
	u-95% CI	5.03	0.87
Ocellar alignment	Estimate	4.67	0.23
	l-95% CI	4.16	-0.11
	u-95% CI	5.18	0.56
Monocular FOV	Estimate	3.91	-0.51
	l-95% CI	3.26	-0.94
	u-95% CI	4.56	-0.08
Binocular overlap	Estimate	1.80	-0.05
	l-95% CI	-0.70	-1.70
	u-95% CI	4.29	1.63

Figure S2. Phylogenetic signals in allometric relationships. Distribution of the percentage of residual variance accounted by the phylogenetic covariance matrix modelled with Bayesian inference. Colours correspond to the significance of the scaling exponent in the allometric relationship without the phylogenetic control (red: positive, blue: negative, grey: no significance).

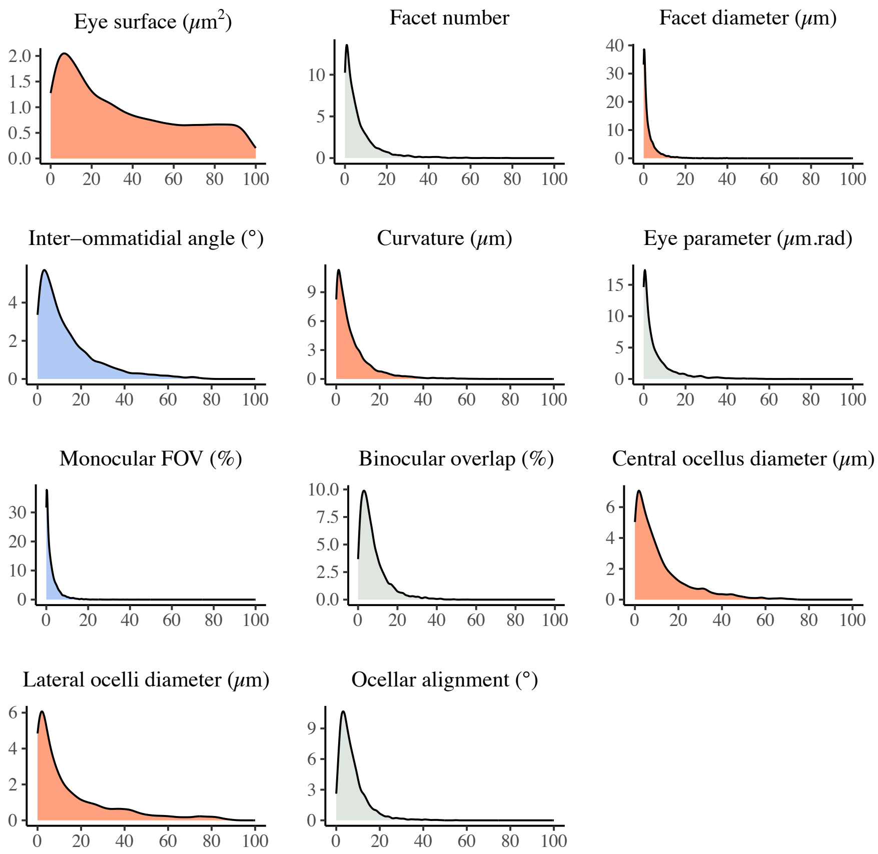

Table S5. Summary of the effects of geophysical variables, tree cover and covariates on the community-weighted means of visual traits and body size, and on species richness and single species abundances modelled with Bayesian inference (Estimate: estimated effect, l-95 % CI: lowest 95 % credible interval, u-95 % CI: upper 95 % credible interval). Significantly positive and negative effects are drawn in red and blue respectively, while non-significant relationships are in grey.

		Intercept	Elevation	Latitude	Tree cover	Longitude	Floral resource	Latitude x Longitude
Eye surface	Estimate	2.22E+06	-5.40E+04	-1.10E+04	-1.25E+04	-1.40E+03	-7.39E+03	-2.10E+04
	l-95% CI	2.19E+06	-7.47E+04	-3.70E+04	-2.87E+04	-1.87E+04	-2.11E+04	-3.45E+04
	u-95% CI	2.24E+06	-3.30E+04	1.63E+04	3.67E+03	1.59E+04	6.05E+03	-7.49E+03
Facet diameter	Estimate	2.28E+01	-2.35E-01	4.11E-02	1.62E-01	-7.45E-02	-3.26E-02	-9.53E-02
	l-95% CI	2.27E+01	-3.00E-01	-4.61E-02	1.12E-01	-1.31E-01	-7.63E-02	-1.37E-01
	u-95% CI	2.28E+01	-1.68E-01	1.28E-01	2.14E-01	-1.87E-02	1.04E-02	-5.15E-02
Inter-ommatidial angle	Estimate	1.85E+00	3.36E-02	9.92E-03	2.45E-02	1.26E-03	-4.73E-03	1.22E-02
	l-95% CI	1.84E+00	2.48E-02	-1.48E-03	1.80E-02	-6.27E-03	-1.04E-02	6.43E-03
	u-95% CI	1.86E+00	4.22E-02	2.15E-02	3.09E-02	8.77E-03	1.06E-03	1.79E-02
Facet number	Estimate	4.89E+03	-2.60E+01	-3.33E+01	-9.75E+01	2.64E+01	-2.45E+00	-7.31E+00
	l-95% CI	4.85E+03	-5.49E+01	-7.04E+01	-1.20E+02	1.56E+00	-2.14E+01	-2.60E+01
	u-95% CI	4.93E+03	2.87E+00	4.33E+00	-7.47E+01	5.12E+01	1.64E+01	1.18E+01
Eye parameter	Estimate	7.37E-01	3.82E-03	4.18E-03	1.55E-02	-2.63E-03	-2.70E-03	9.83E-04
	l-95% CI	7.33E-01	2.97E-04	-2.44E-04	1.29E-02	-5.65E-03	-5.00E-03	-1.30E-03
	u-95% CI	7.40E-01	7.26E-03	8.74E-03	1.82E-02	4.02E-04	-3.55E-04	3.25E-03
Eye curvature	Estimate	7.46E+02	-1.66E+01	-2.16E+00	-5.63E+00	-1.08E+00	-3.49E-02	-6.42E+00
	l-95% CI	7.39E+02	-2.10E+01	-8.48E+00	-9.12E+00	-5.10E+00	-3.11E+00	-9.36E+00
	u-95% CI	7.50E+02	-1.19E+01	3.97E+00	-1.83E+00	2.88E+00	2.94E+00	-3.32E+00
Central ocellus diameter	Estimate	2.34E+02	-5.66E+00	-1.56E+00	-1.84E+00	-8.54E-01	4.16E-01	-2.04E+00
	l-95% CI	2.32E+02	-6.96E+00	-3.42E+00	-2.86E+00	-1.97E+00	-4.79E-01	-2.93E+00
	u-95% CI	2.36E+02	-4.26E+00	4.05E-01	-8.23E-01	2.57E-01	1.27E+00	-1.10E+00
Lateral ocelli diameter	Estimate	2.13E+02	-2.50E+00	-1.16E+00	-1.71E-01	-1.11E-01	-5.46E-02	-1.24E+00
	l-95% CI	2.11E+02	-3.49E+00	-2.32E+00	-1.02E+00	-9.02E-01	-7.04E-01	-1.86E+00
	u-95% CI	2.15E+02	-1.46E+00	8.62E-02	6.42E-01	6.70E-01	5.76E-01	-5.56E-01
Ocellar alignment	Estimate	1.49E+02	-1.37E-01	7.29E-01	7.55E-01	9.84E-02	-1.40E-02	-3.93E-01
	l-95% CI	1.48E+02	-8.64E-01	-2.19E-01	2.13E-01	-5.39E-01	-5.10E-01	-8.66E-01
	u-95% CI	1.50E+02	6.24E-01	1.64E+00	1.35E+00	7.33E-01	4.67E-01	8.55E-02
Monocular FOV	Estimate	2.41E+01	8.27E-01	3.94E-02	-3.72E-02	2.78E-01	-1.28E-01	2.86E-01
	l-95% CI	2.39E+01	6.62E-01	-1.55E-01	-1.83E-01	1.48E-01	-2.28E-01	1.85E-01
	u-95% CI	2.43E+01	9.82E-01	2.90E-01	7.83E-02	4.09E-01	-2.20E-02	3.91E-01
Binocular overlap	Estimate	6.27E+00	4.39E-01	1.39E-01	-3.09E-02	1.95E-01	-1.00E-01	1.19E-01
	l-95% CI	6.13E+00	3.32E-01	8.75E-03	-1.25E-01	1.08E-01	-1.70E-01	5.24E-02
	u-95% CI	6.42E+00	5.44E-01	2.63E-01	5.62E-02	2.82E-01	-3.02E-02	1.84E-01

ITD	Estimate	3.96E+00	-8.40E-02	-2.28E-02	-5.76E-03	-8.43E-03	4.41E-03	-2.70E-02
	l-95% CI	3.94E+00	-1.00E-01	-4.24E-02	-1.77E-02	-2.36E-02	-6.74E-03	-3.74E-02
	u-95% CI	3.97E+00	-6.77E-02	-3.24E-03	6.11E-03	6.61E-03	1.56E-02	-1.67E-02
Observed species number	Estimate	1.68E+00	1.22E-02	8.45E-02	-9.15E-03	-7.25E-03	1.46E-01	-4.33E-02
	l-95% CI	1.65E+00	-3.51E-02	2.85E-02	-4.35E-02	-5.15E-02	1.14E-01	-7.38E-02
	u-95% CI	1.72E+00	5.93E-02	1.40E-01	2.57E-02	3.81E-02	1.80E-01	-1.23E-02
B. ruderarius (abundance)	Estimate	-8.70E-01	-4.37E-01	5.55E-02	-1.88E-01	-3.94E-02	3.60E-01	6.83E-01
	l-95% CI	-1.64E+00	-1.42E+00	-5.86E-01	-5.71E-01	-5.11E-01	1.48E-01	8.10E-02
	u-95% CI	-2.46E-01	4.59E-01	6.88E-01	1.88E-01	4.47E-01	5.84E-01	1.29E+00
B. sporadicus (abundance)	Estimate	-6.49E-01	-2.39E-01	7.94E-01	8.07E-02	-2.53E-02	3.06E-01	-7.42E-02
	l-95% CI	-1.98E+00	-8.96E-01	-2.32E-01	-4.56E-01	-1.67E+00	2.79E-02	-6.36E-01
	u-95% CI	6.03E-01	4.14E-01	1.85E+00	6.22E-01	1.55E+00	5.98E-01	4.92E-01
B. hortorum (abundance)	Estimate	-6.95E-01	2.38E-01	-2.58E-01	7.81E-02	-1.19E-01	4.35E-01	2.57E-01
	l-95% CI	-1.10E+00	-1.64E-01	-7.57E-01	-1.99E-01	-5.17E-01	3.01E-01	1.85E-02
	u-95% CI	-3.42E-01	6.28E-01	2.45E-01	3.56E-01	2.76E-01	5.75E-01	4.94E-01
B. hypnorum (abundance)	Estimate	-2.05E-01	-1.96E-01	6.04E-01	2.19E-01	-2.86E-01	3.66E-01	1.74E-02
	l-95% CI	-4.86E-01	-4.44E-01	3.06E-01	-3.73E-03	-5.80E-01	2.30E-01	-1.28E-01
	u-95% CI	5.17E-02	5.00E-02	9.22E-01	4.40E-01	2.61E-03	5.05E-01	1.61E-01
B. sylvarum (abundance)	Estimate	-5.29E-01	-5.75E-01	-3.30E-01	-3.56E-01	-7.27E-01	7.71E-01	-4.84E-01
	l-95% CI	-9.75E-01	-1.18E+00	-8.21E-01	-6.42E-01	-1.07E+00	6.45E-01	-1.00E+00
	u-95% CI	-1.35E-01	4.60E-03	1.68E-01	-7.56E-02	-4.00E-01	9.07E-01	1.75E-02
B. jonellus (abundance)	Estimate	-3.35E-01	3.67E-01	3.58E-01	7.63E-02	1.46E-01	2.60E-01	1.39E-01
	l-95% CI	-7.99E-01	6.54E-02	-4.42E-02	-2.35E-01	-3.16E-01	1.41E-01	-7.14E-02
	u-95% CI	8.21E-02	6.75E-01	7.66E-01	3.92E-01	6.25E-01	3.81E-01	3.44E-01
B. soroeensis (abundance)	Estimate	3.60E-01	-2.84E-01	1.52E-01	2.26E-01	-3.89E-01	3.49E-01	1.94E-02
	l-95% CI	1.20E-01	-6.08E-01	-1.33E-01	5.06E-04	-6.54E-01	2.23E-01	-1.90E-01
	u-95% CI	5.86E-01	3.26E-02	4.32E-01	4.55E-01	-1.27E-01	4.76E-01	2.26E-01
B. lapidarius (abundance)	Estimate	2.77E-02	1.29E-01	-3.63E-01	-2.67E-01	2.73E-02	4.62E-01	3.38E-01
	l-95% CI	-2.26E-01	-1.91E-01	-6.61E-01	-4.49E-01	-2.29E-01	3.67E-01	7.22E-03
	u-95% CI	2.55E-01	4.43E-01	-6.58E-02	-8.51E-02	2.95E-01	5.57E-01	6.61E-01
B. terrestris (abundance)	Estimate	4.63E-01	1.76E-01	-6.31E-01	-1.48E-01	1.44E-01	3.31E-01	-2.34E-03
	l-95% CI	2.45E-01	-1.35E-01	-9.07E-01	-3.08E-01	-7.48E-02	2.51E-01	-2.96E-01
	u-95% CI	6.69E-01	4.82E-01	-3.63E-01	3.09E-03	3.60E-01	4.10E-01	2.83E-01
B. pratorum (abundance)	Estimate	1.98E-01	2.10E-01	1.27E-01	3.85E-01	-2.70E-01	3.78E-01	1.13E-01
	l-95% CI	-1.25E-02	1.80E-02	-1.14E-01	1.93E-01	-4.91E-01	3.02E-01	-1.08E-02
	u-95% CI	3.96E-01	4.08E-01	3.67E-01	5.80E-01	-4.78E-02	4.55E-01	2.35E-01
B. lucorum (abundance)	Estimate	8.98E-01	-3.03E-02	1.90E-01	1.92E-01	3.32E-02	8.89E-02	-4.38E-02
	l-95% CI	7.59E-01	-2.11E-01	-5.93E-03	5.96E-02	-1.37E-01	2.99E-02	-1.50E-01
	u-95% CI	1.03E+00	1.50E-01	3.97E-01	3.24E-01	2.00E-01	1.47E-01	5.86E-02
B. pascuorum (abundance)	Estimate	9.17E-01	1.54E-01	-1.63E-01	2.59E-01	4.39E-02	4.00E-01	-3.09E-02
	l-95% CI	8.03E-01	2.17E-02	-3.22E-01	1.46E-01	-8.35E-02	3.47E-01	-1.19E-01

u-95% CI	1.03E+00	2.92E-01	-5.42E-03	3.76E-01	1.74E-01	4.53E-01	5.70E-02

Figure S3. Effect of tree cover on the community weighted means of eleven visual traits relative to body size in bumblebee communities within grasslands across Sweden (n = 812). The residuals back transformed to trait space from the simple allometric models were used to calculate traits relative to body size. Grey circles represent the original data. Dark lines are the estimated effects of tree cover modelled with Bayesian inference and shaded areas represent the Bayesian 95% credible intervals. Significantly positive and negative effects are drawn in red and blue respectively, while non-significant relationships are in grey.

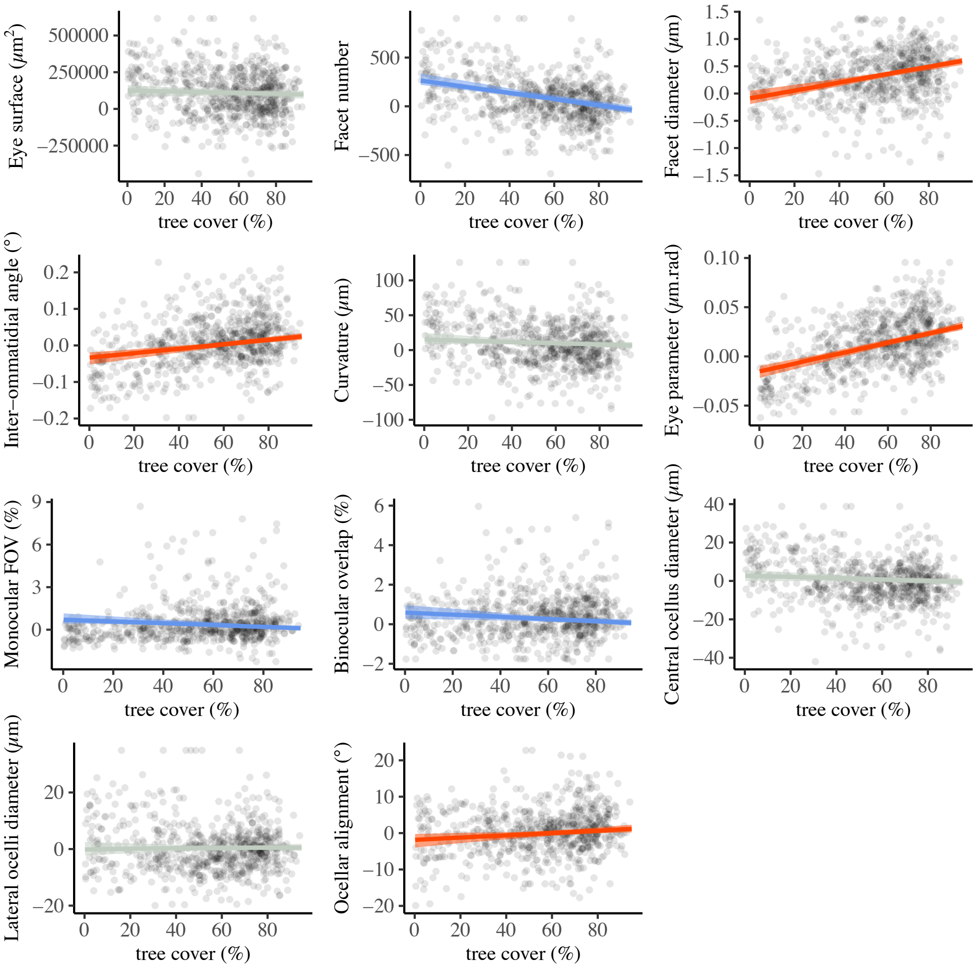

Table S6. Summary of the effects of geophysical variables, tree cover and covariates on the community-weighted means of visual traits relative to body size modelled with Bayesian inference (Estimate: estimated effect, l-95 % CI: lowest 95 % credible interval, u-95 % CI: upper 95 % credible interval). Significantly positive and negative effects are drawn in red and blue respectively, while non-significant relationships are in grey.

		Intercept	Elevation	Latitude	Tree cover	Longitude	Floral resource	Latitude x Longitude
Eye surface	Estimate	1.11E+05	-1.69E+04	-3.72E+04	-5.75E+03	2.89E+03	-8.65E+03	-4.83E+03
	l-95% CI	8.99E+04	-3.46E+04	-6.07E+04	-1.98E+04	-1.23E+04	-2.04E+04	-1.61E+04
	u-95% CI	1.35E+05	8.58E+02	-1.44E+04	8.11E+03	1.81E+04	3.13E+03	6.50E+03

Facet diameter	Estimate	3.06E-01	-1.01E-01	-5.38E-02	1.69E-01	-5.70E-02	-2.98E-02	-4.15E-02
	l-95% CI	2.52E-01	-1.51E-01	-1.18E-01	1.29E-01	-1.00E-01	-6.28E-02	-7.41E-02
	u-95% CI	3.61E-01	-5.15E-02	1.06E-02	2.08E-01	-1.38E-02	3.20E-03	-8.55E-03
Inter-ommatidial angle	Estimate	-4.70E-04	1.88E-02	2.26E-02	1.46E-02	5.58E-04	-2.05E-03	5.58E-03
	l-95% CI	-8.38E-03	1.22E-02	1.38E-02	9.52E-03	-5.04E-03	-6.33E-03	1.16E-03
	u-95% CI	7.74E-03	2.53E-02	3.06E-02	1.99E-02	6.11E-03	2.23E-03	9.69E-03
Facet number	Estimate	9.65E+01	2.77E+00	-5.86E+01	-7.66E+01	2.72E+01	-8.12E+00	4.78E+00
	l-95% CI	6.53E+01	-2.40E+01	-9.25E+01	-9.82E+01	4.55E+00	-2.56E+01	-1.21E+01
	u-95% CI	1.37E+02	2.96E+01	-2.36E+01	-5.63E+01	4.97E+01	9.43E+00	2.24E+01
Eye parameter	Estimate	1.10E-02	2.60E-03	6.80E-03	1.17E-02	-2.29E-03	-1.61E-03	4.21E-04
	l-95% CI	7.55E-03	-3.03E-04	3.18E-03	9.49E-03	-4.65E-03	-3.45E-03	-1.44E-03
	u-95% CI	1.42E-02	5.43E-03	1.05E-02	1.39E-02	5.87E-05	2.72E-04	2.27E-03
Eye curvature	Estimate	1.04E+01	-6.65E+00	-1.14E+01	-2.02E+00	-2.30E-01	-6.07E-01	-2.29E+00
	l-95% CI	6.98E+00	-9.95E+00	-1.55E+01	-4.53E+00	-3.13E+00	-2.81E+00	-4.39E+00
	u-95% CI	1.42E+01	-3.32E+00	-7.13E+00	4.44E-01	2.67E+00	1.61E+00	-1.36E-01
Central ocellus diameter	Estimate	8.26E-01	-2.65E+00	-4.19E+00	-8.08E-01	-5.75E-01	2.45E-01	-7.92E-01
	l-95% CI	-5.91E-01	-3.80E+00	-5.70E+00	-1.70E+00	-1.56E+00	-5.17E-01	-1.54E+00
	u-95% CI	2.21E+00	-1.49E+00	-2.54E+00	9.31E-02	4.10E-01	1.00E+00	-1.36E-02
Lateral ocelli diameter	Estimate	3.19E-01	-4.50E-01	-2.61E+00	1.41E-01	1.51E-01	-7.64E-02	-3.45E-01
	l-95% CI	-6.20E-01	-1.37E+00	-3.82E+00	-5.80E-01	-6.44E-01	-6.88E-01	-9.49E-01
	u-95% CI	1.37E+00	4.76E-01	-1.32E+00	8.33E-01	9.49E-01	5.33E-01	2.66E-01
Ocellar alignment	Estimate	-1.59E-01	5.14E-01	3.14E-01	7.69E-01	1.76E-01	3.12E-03	-1.09E-01
	l-95% CI	-9.80E-01	-1.97E-01	-6.20E-01	2.07E-01	-4.52E-01	-4.75E-01	-5.68E-01
	u-95% CI	6.57E-01	1.23E+00	1.21E+00	1.34E+00	8.08E-01	4.82E-01	3.52E-01
Monocular FOV	Estimate	3.76E-01	6.26E-01	2.02E-01	-1.54E-01	2.68E-01	-9.89E-02	1.96E-01
	l-95% CI	2.40E-01	4.89E-01	4.47E-02	-2.73E-01	1.58E-01	-1.84E-01	1.12E-01
	u-95% CI	6.06E-01	7.52E-01	3.59E-01	-5.53E-02	3.79E-01	-9.50E-03	2.75E-01
Binocular overlap	Estimate	2.88E-01	4.54E-01	1.68E-01	-1.22E-01	2.11E-01	-7.20E-02	1.22E-01
	l-95% CI	1.42E-01	3.37E-01	1.39E-02	-2.21E-01	1.11E-01	-1.50E-01	4.67E-02
	u-95% CI	4.43E-01	5.71E-01	3.11E-01	-2.25E-02	3.11E-01	5.74E-03	1.95E-01

Figure S4. Relationship between tree cover and species number in bumblebee communities within grasslands across Sweden (n = 812). The grey line is the estimated effects of tree cover modelled with Bayesian inference and shaded areas represent the Bayesian 95% credible interval.

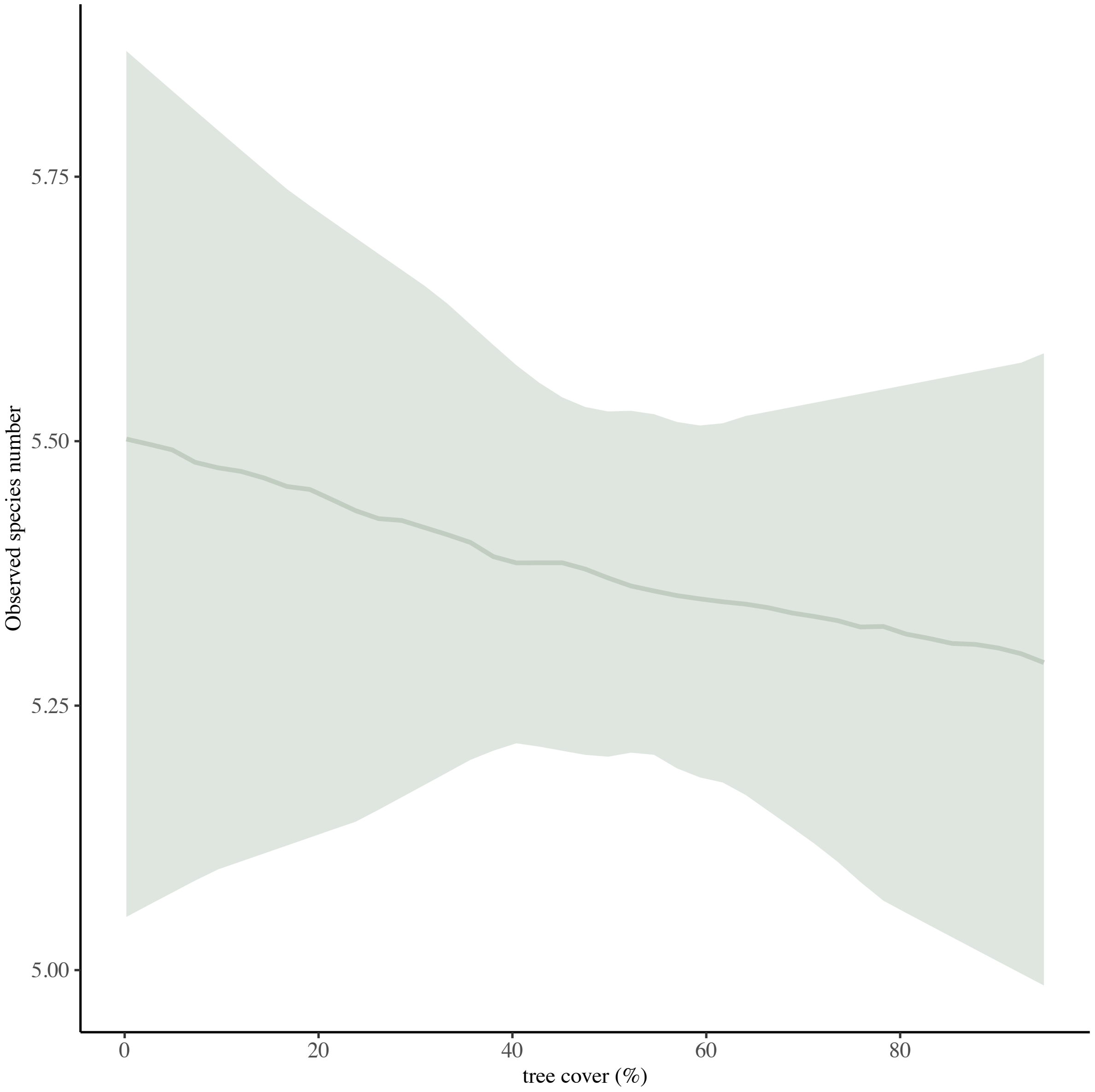
Observed species number
5.75
5.50
5.25
5.00
0
20
40
60
80
tree cover (%)
