# Supplementary material for: The Interplay Between Visual Traits and Forest in Bumblebee Communities Across Sweden
Source: Ecol Evol. 2024 Dec 23;14(12):e70635. doi: 10.1002/ece3.70635 (PMC11664236; doi:10.1002/ece3.70635)
Supplement: Supplementary file 3 — Appendix S3. [file ECE3-14-e70635-s002.pdf]

Allometric models. Model traces and posterior predictive check for each visual trait modelled with Bayesian inference without phylogenetic control.

Eye surface (μm^2)

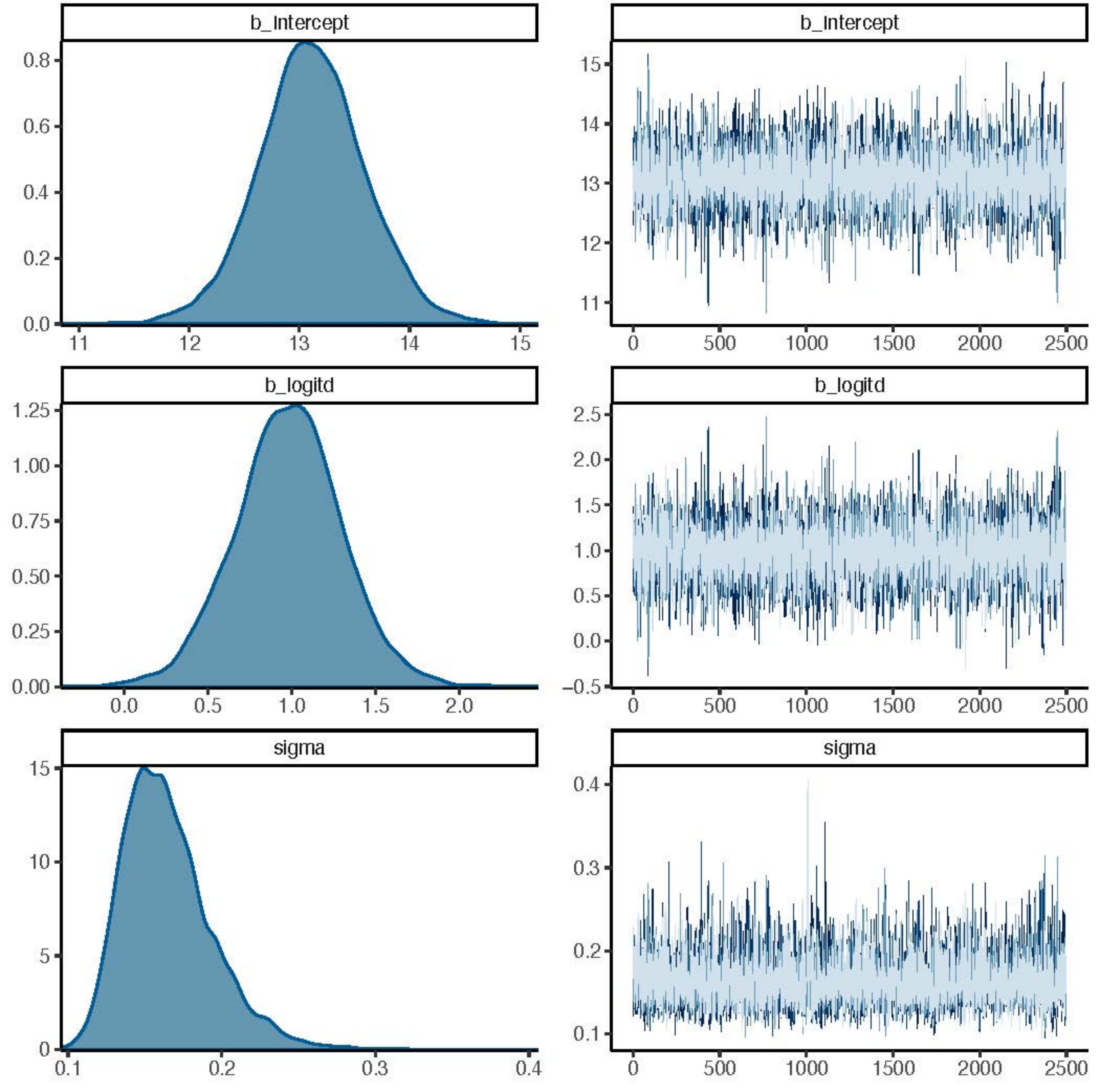

b_Intercept
b_Intercept
b_logitd
b_logitd
sigma
sigma

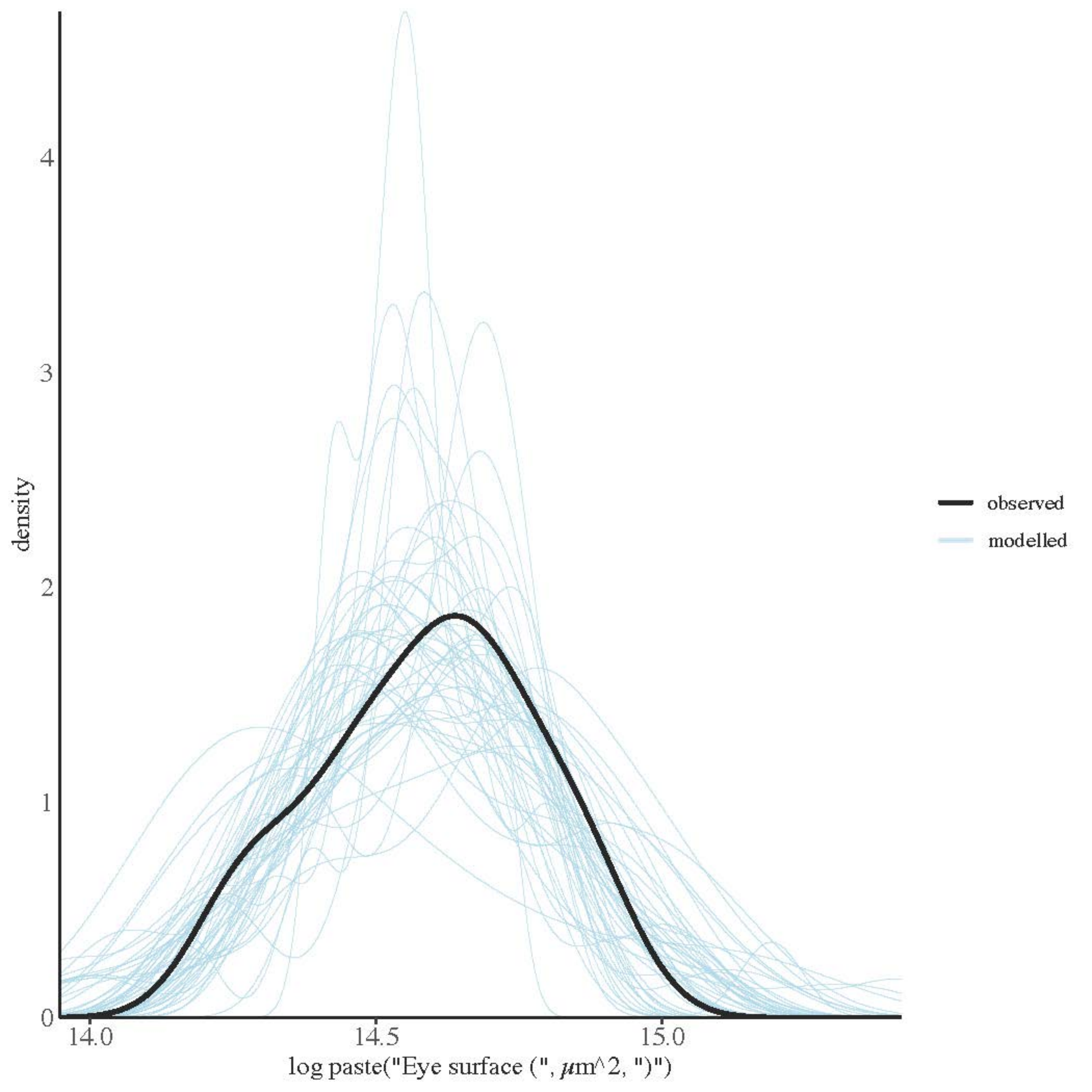

density
4
3
2
1
0
14.0
14.5
15.0
log paste("Eye surface (", μm^2, ")")
observed
modelled

Facet diameter (μm)

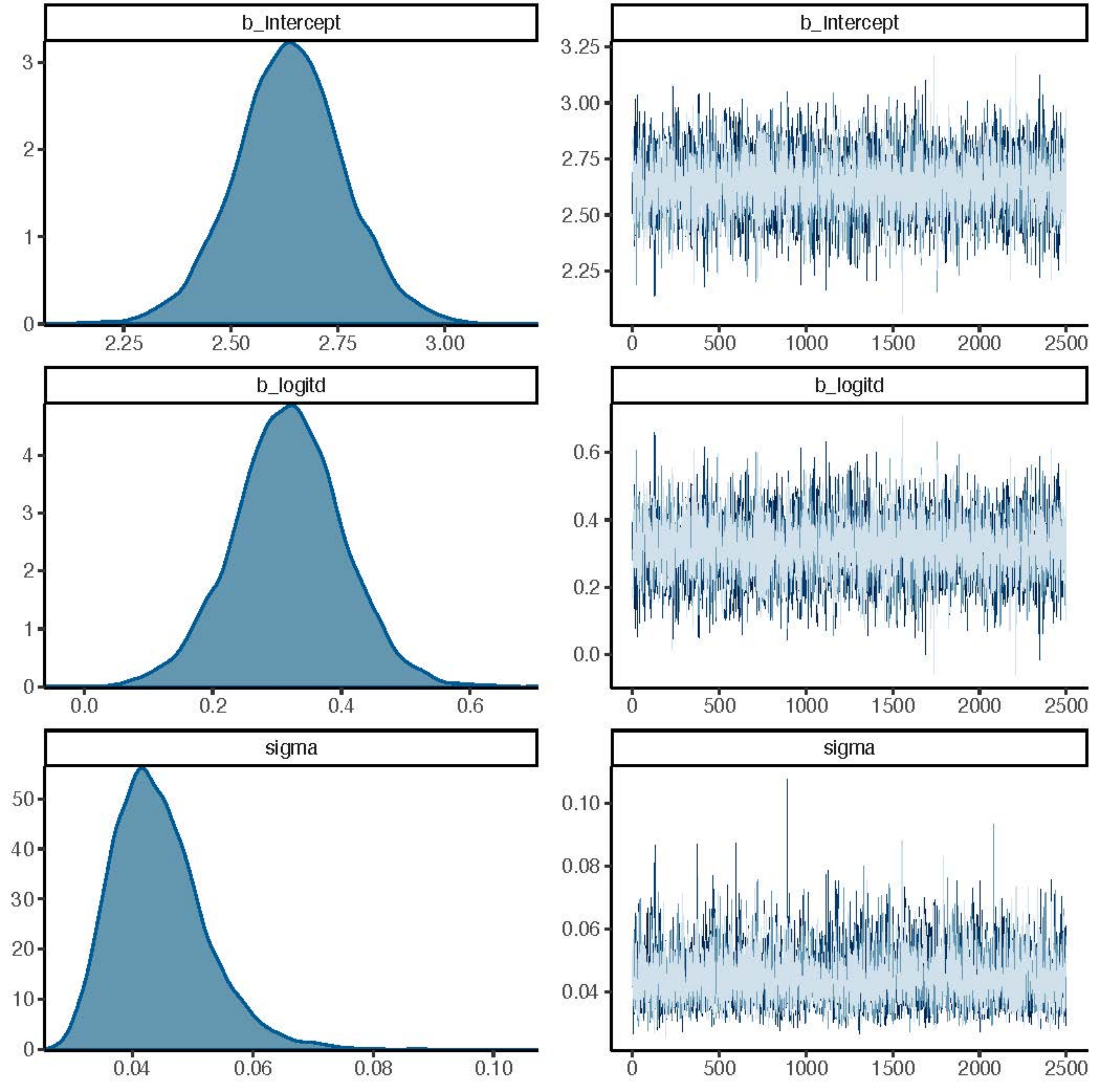

b_Intercept
3
2
1
0
2.25
2.50
2.75
3.00
b_Intercept
3.25
3.00
2.75
2.50
2.25
0
500
1000
1500
2000
2500
b_logitd
4
3
2
1
0
0.0
0.2
0.4
0.6
b_logitd
0.6
0.4
0.2
0.0
0
500
1000
1500
2000
2500
sigma
50
40
30
20
10
0
0.04
0.06
0.08
0.10
sigma
0.10
0.08
0.06
0.04
0
500
1000
1500
2000
2500

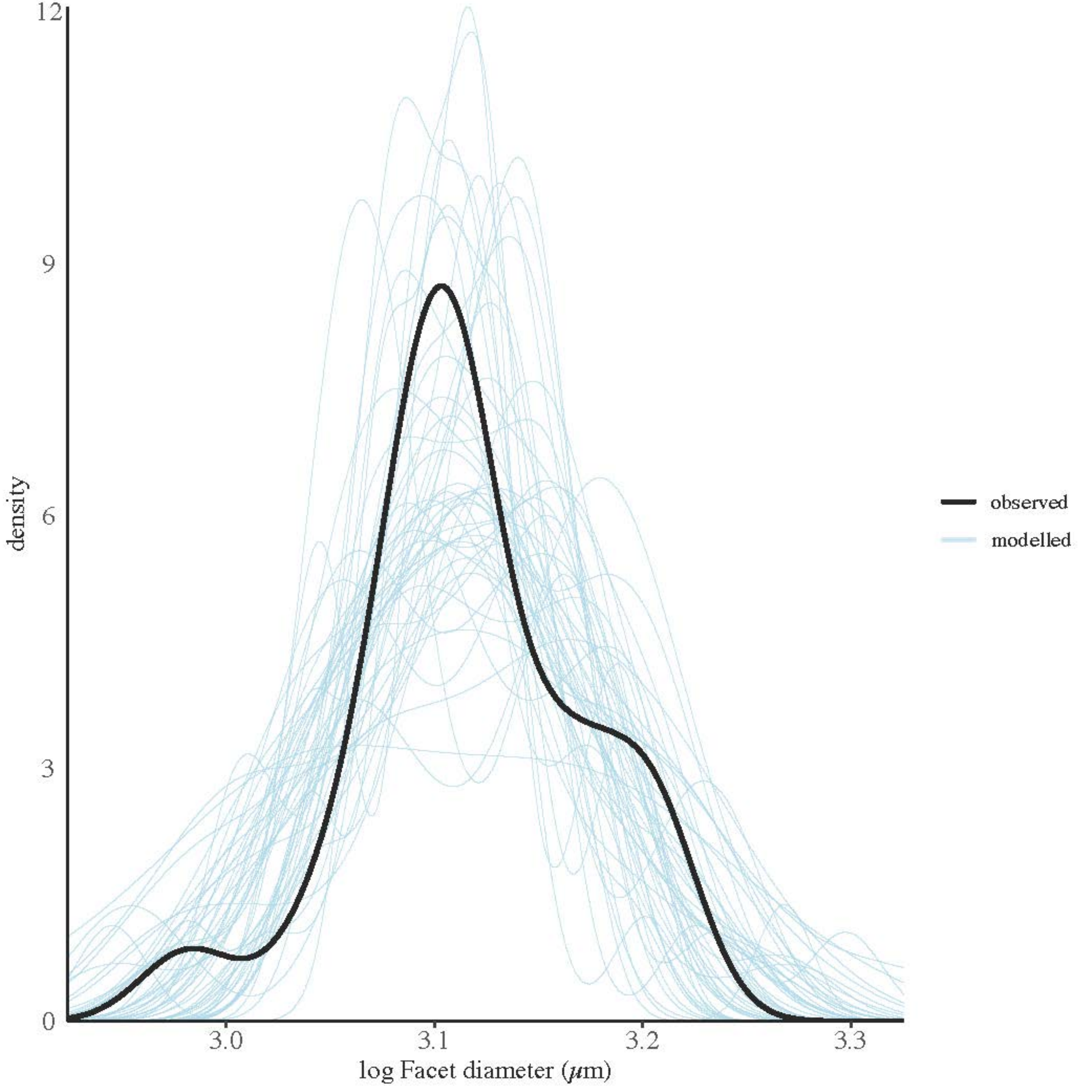
12
9
6
3
0
density
3.0
3.1
3.2
3.3
log Facet diameter (μm)
observed
modelled

Inter–ommatidial angle (°)

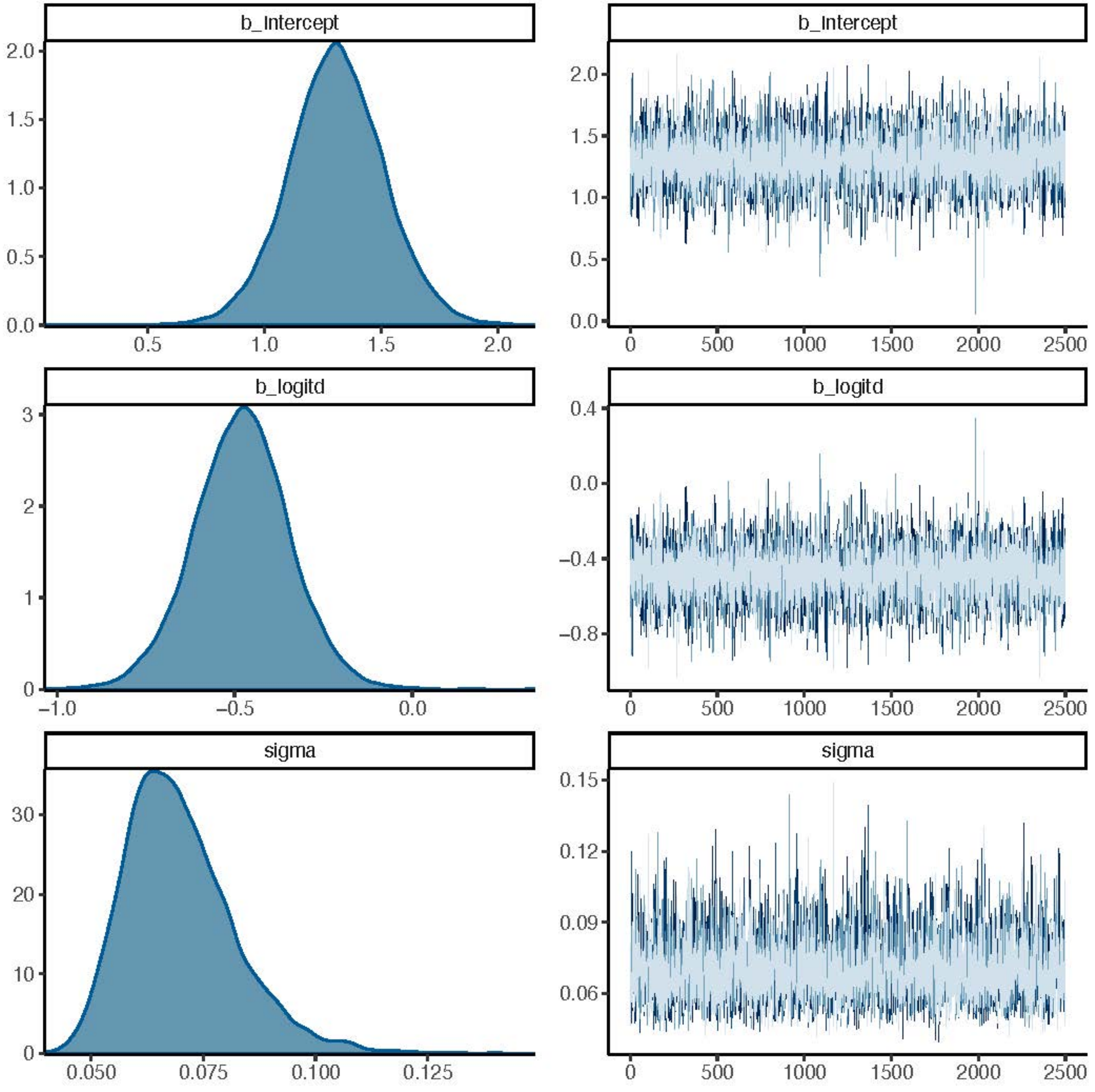

b_Intercept
b_Intercept
b_logitd
b_logitd
sigma
sigma

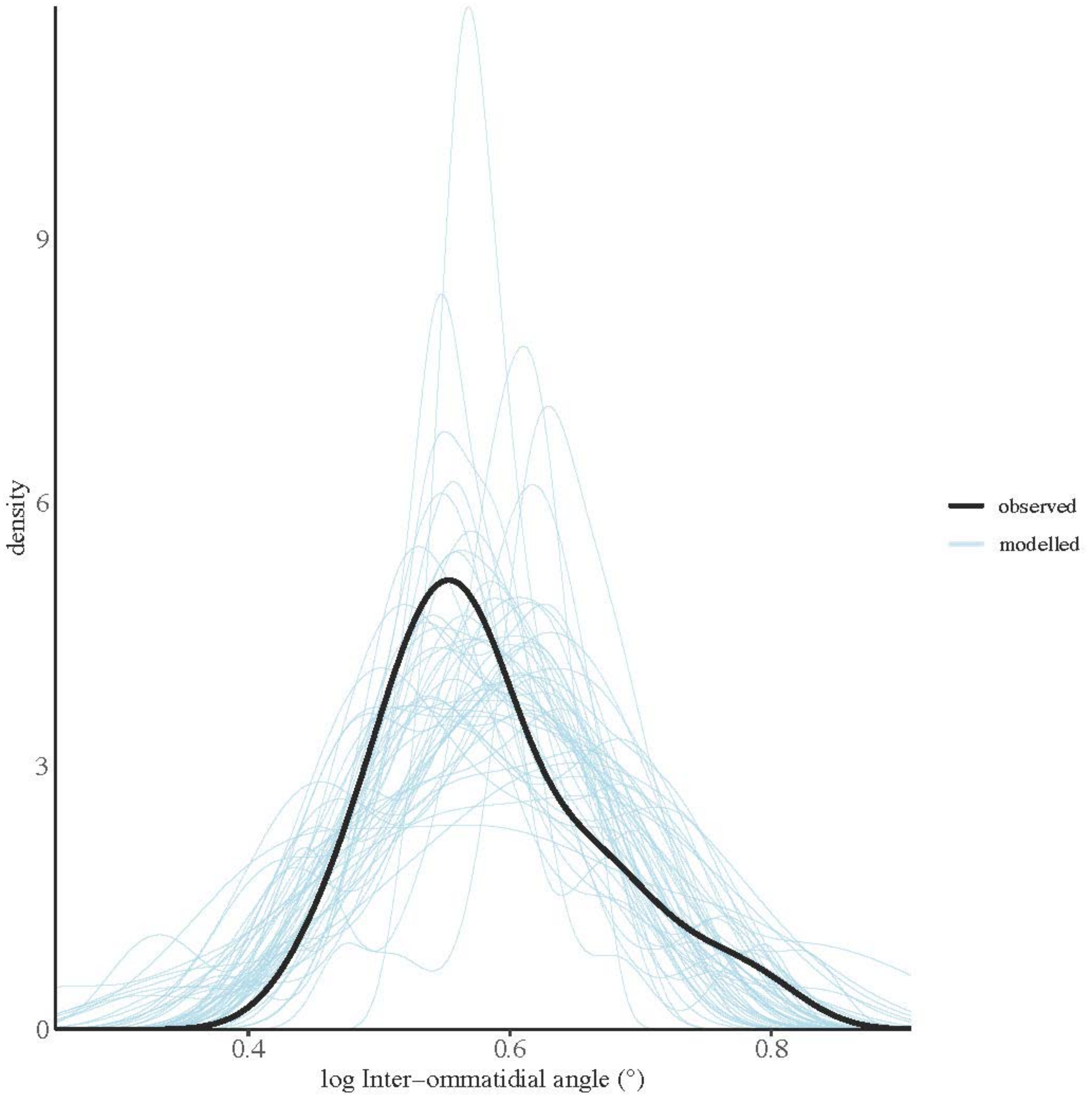

density
9
6
3
0
0.4
0.6
0.8
log Inter−ommatidial angle (°)
observed
modelled

Facet number

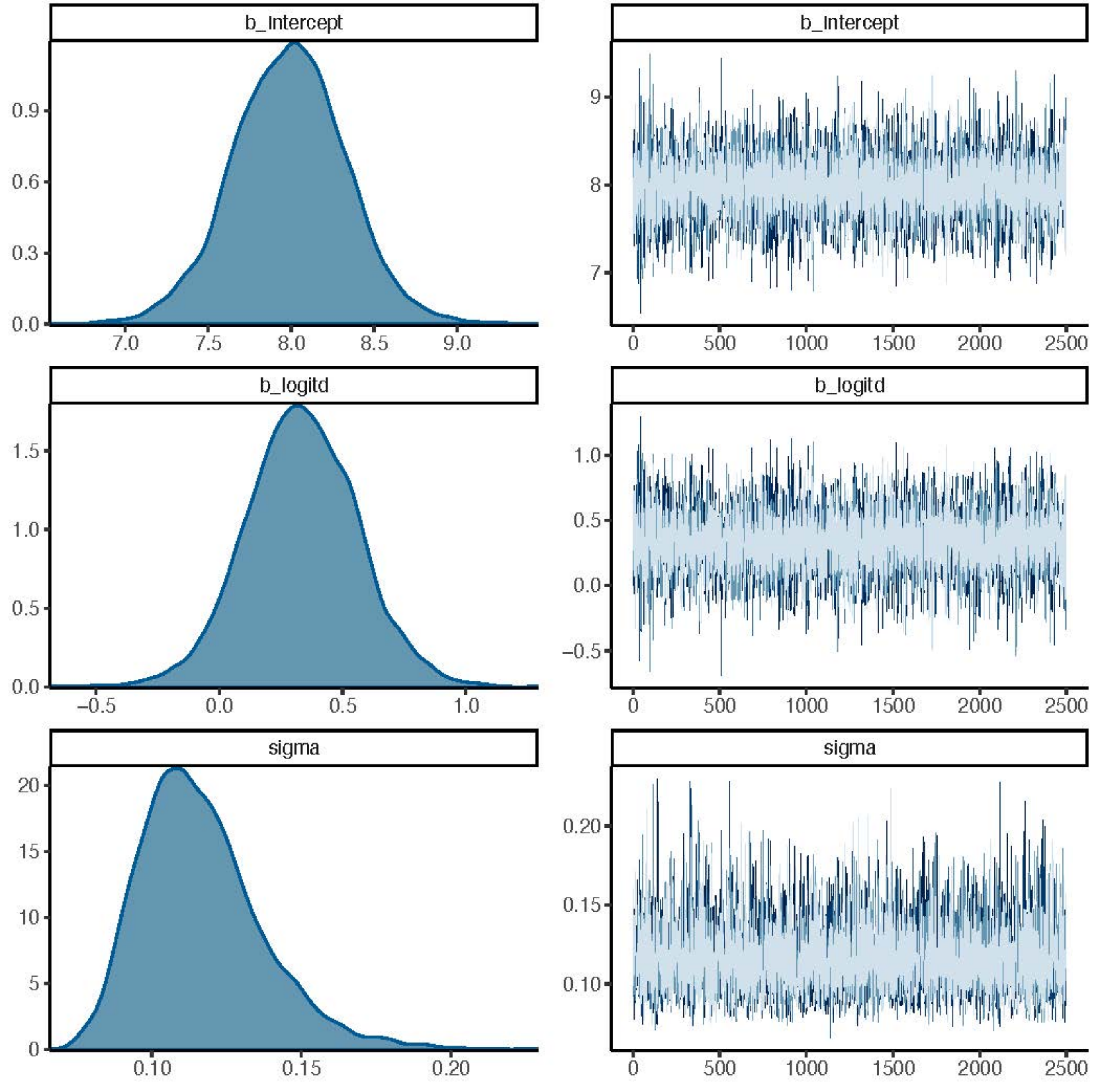
b_Intercept
b_Intercept
b_logitd
b_logitd
sigma
sigma

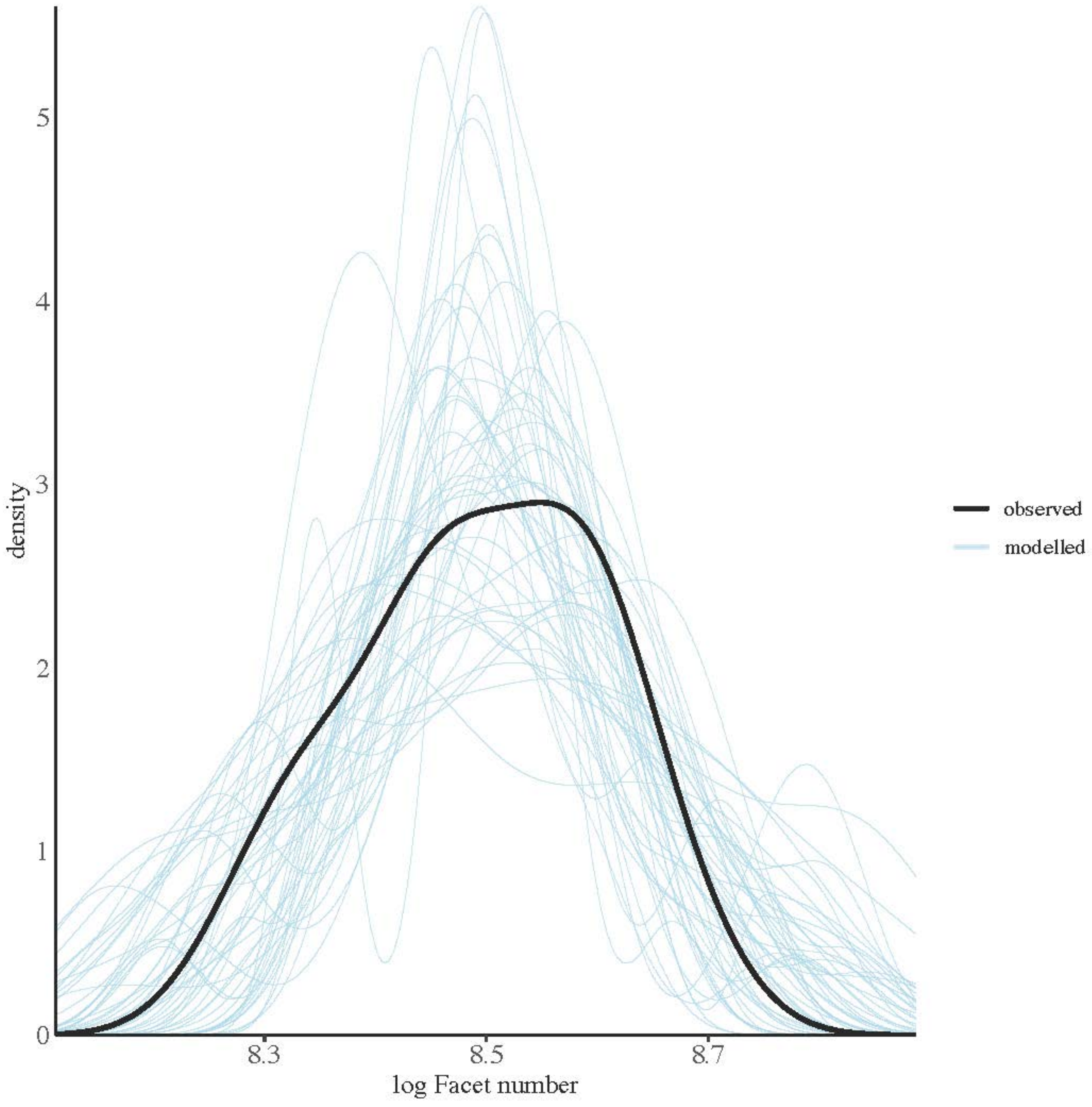

density
0
1
2
3
4
5
8.3
8.5
8.7
log Facet number
observed
modelled

Eye parameter (μm.rad)

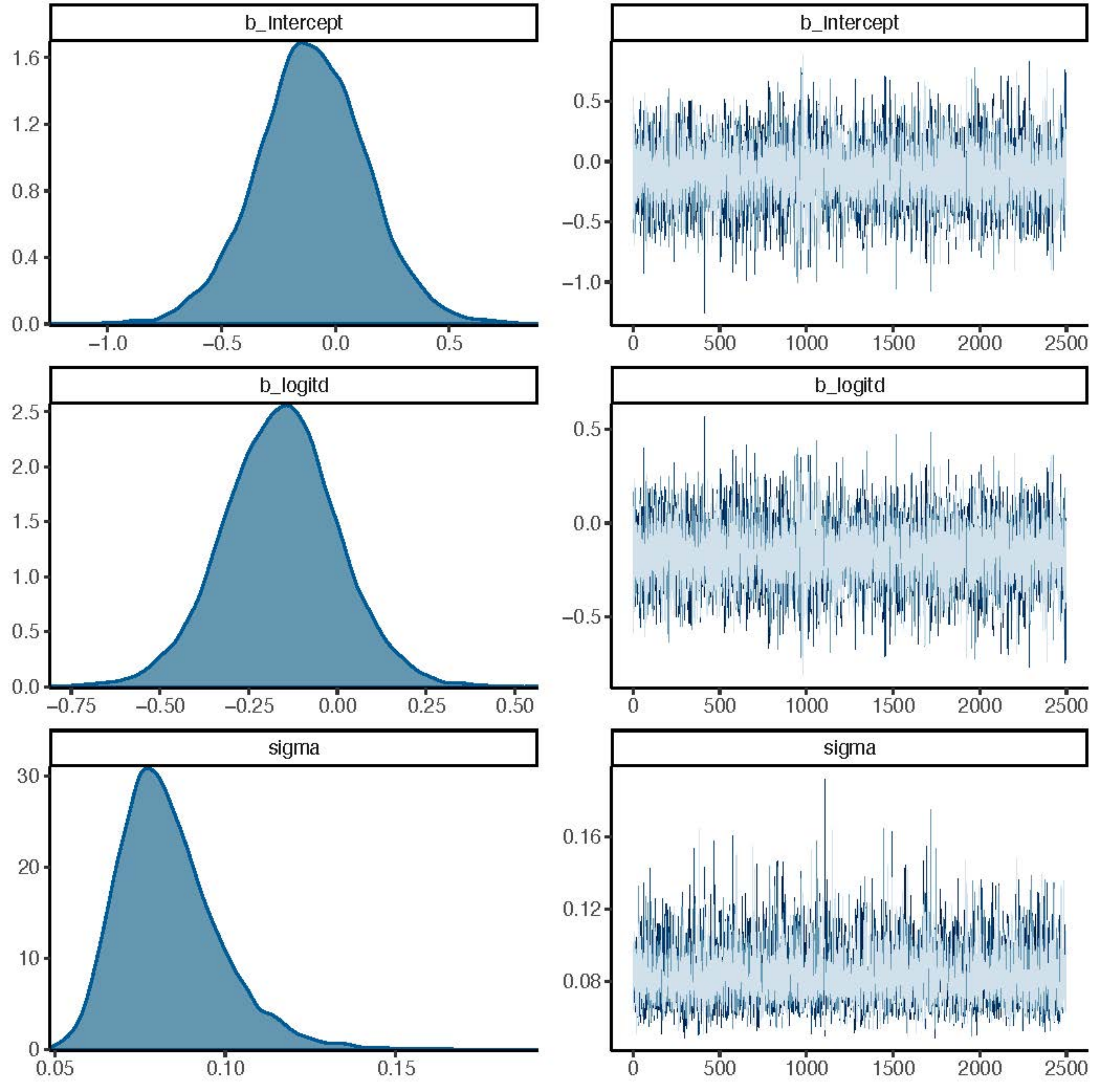

b_Intercept
1.6
1.2
0.8
0.4
0.0
−1.0
−0.5
0.0
0.5
b_Intercept
0.5
0.0
−0.5
−1.0
0
500
1000
1500
2000
2500
b_logitd
2.5
2.0
1.5
1.0
0.5
0.0
−0.75
−0.50
−0.25
0.00
0.25
0.50
b_logitd
0.5
0.0
−0.5
0
500
1000
1500
2000
2500
sigma
30
20
10
0
0.05
0.10
0.15
sigma
0.16
0.12
0.08
0
500
1000
1500
2000
2500

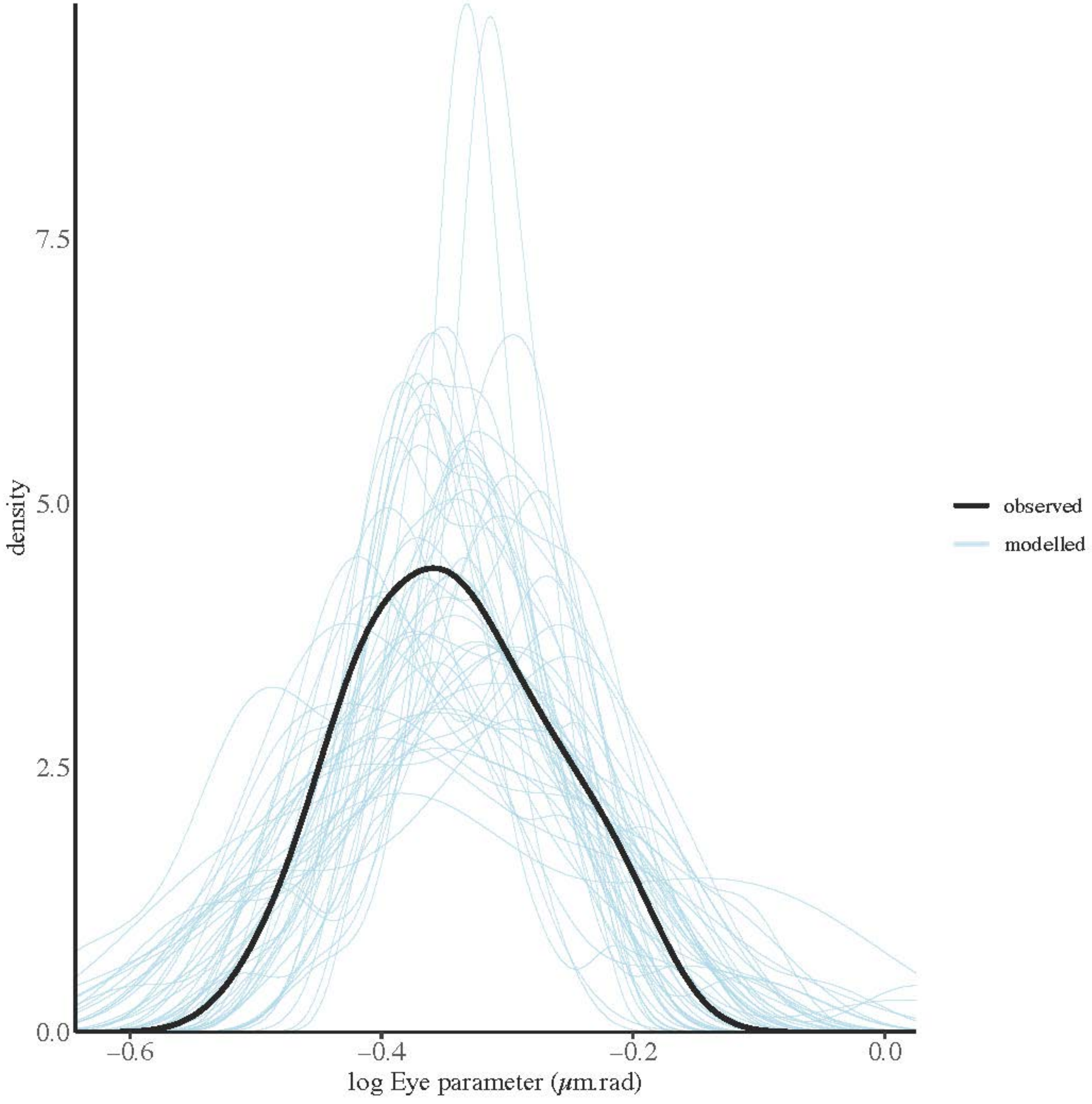

density
7.5
5.0
2.5
0.0
−0.6
−0.4
−0.2
0.0
log Eye parameter (μm.rad)
observed
modelled

Curvature (μm)

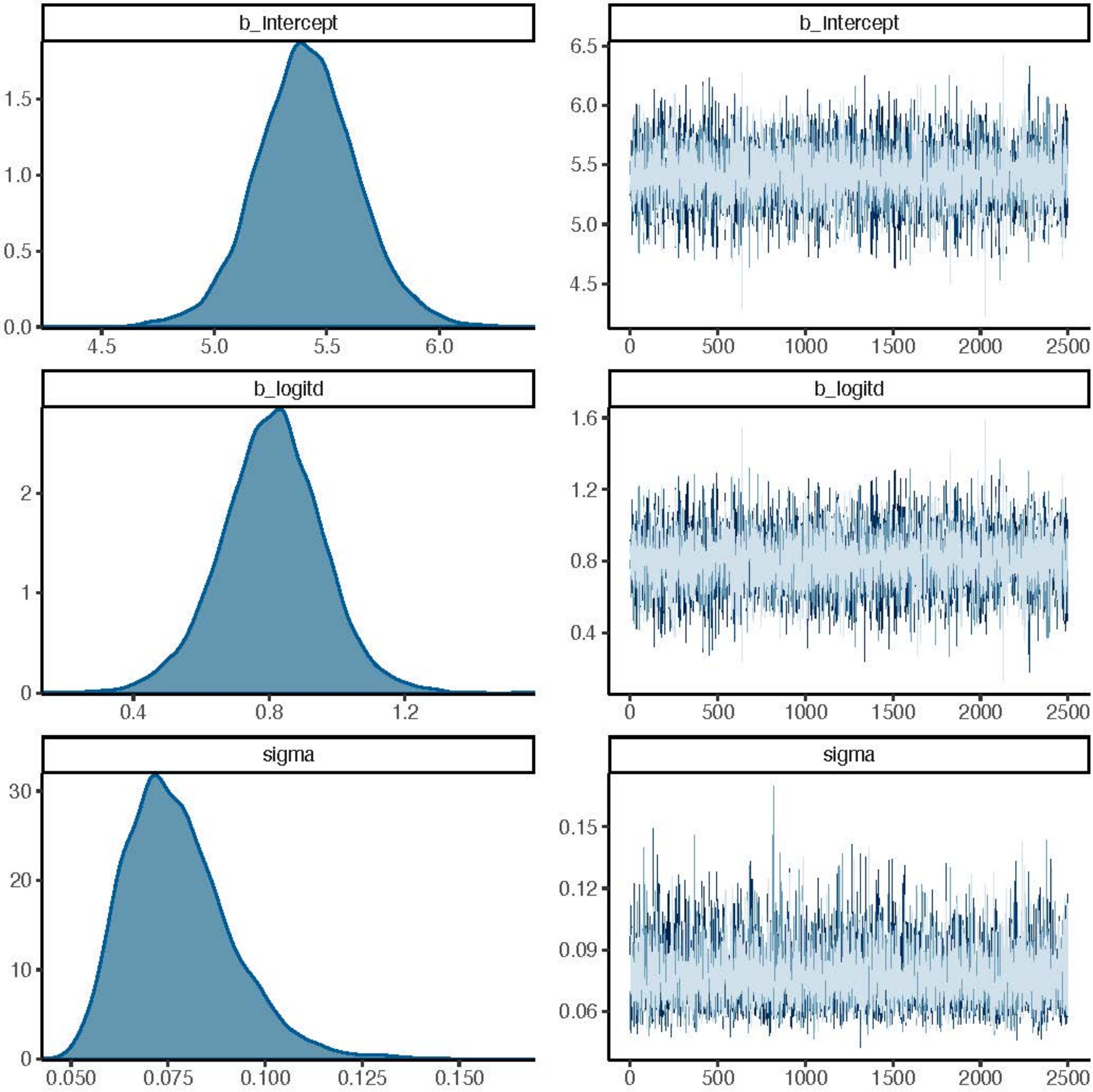

b_Intercept
1.5
1.0
0.5
0.0
4.5
5.0
5.5
6.0
b_Intercept
6.5
6.0
5.5
5.0
4.5
0
500
1000
1500
2000
2500
b_logitd
2
1
0
0.4
0.8
1.2
b_logitd
1.6
1.2
0.8
0.4
0
500
1000
1500
2000
2500
sigma
30
20
10
0
0.050
0.075
0.100
0.125
0.150
sigma
0.15
0.12
0.09
0.06
0
500
1000
1500
2000
2500

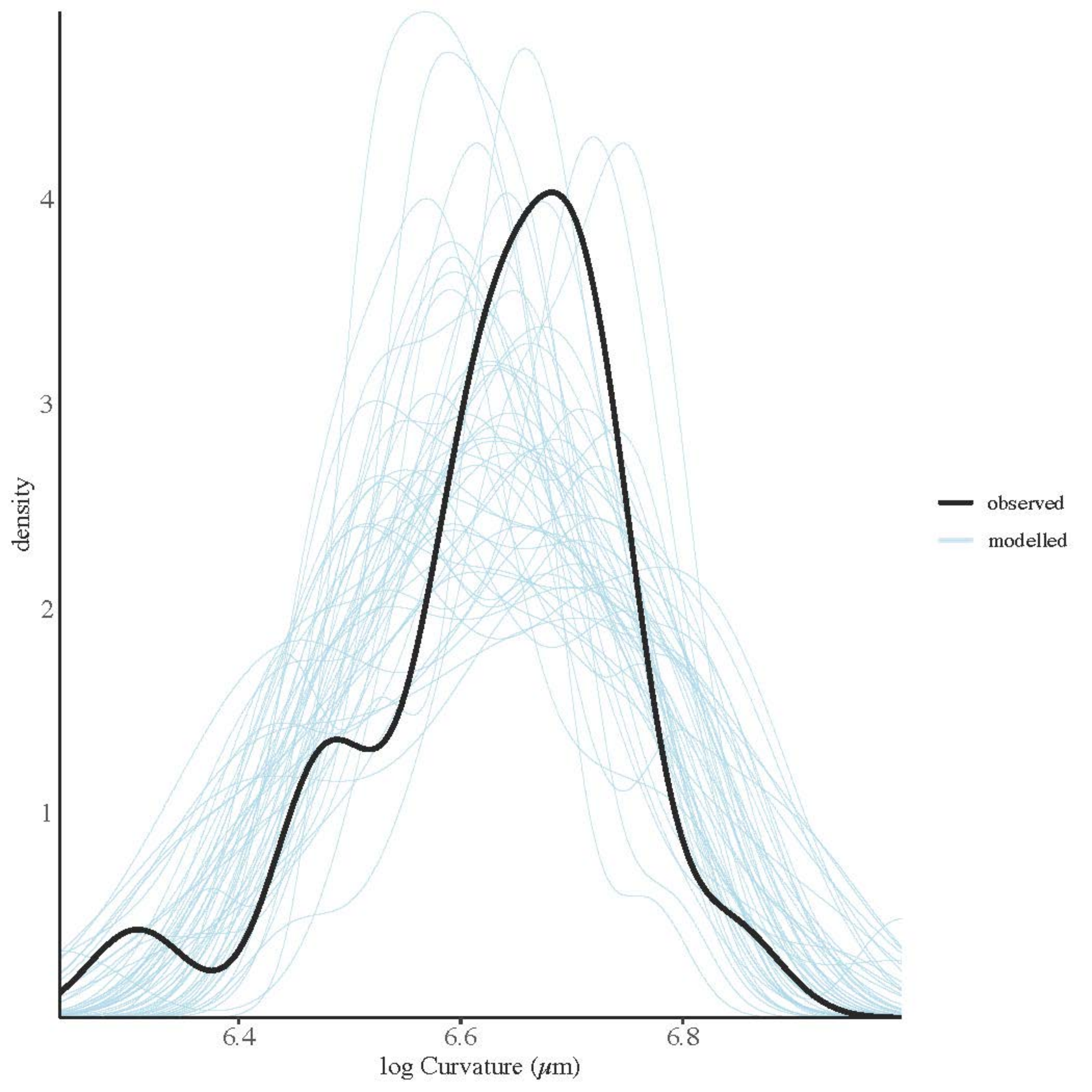

density
1
2
3
4
6.4
6.6
6.8
log Curvature (μm)
observed
modelled

Central ocellus diameter (μm)

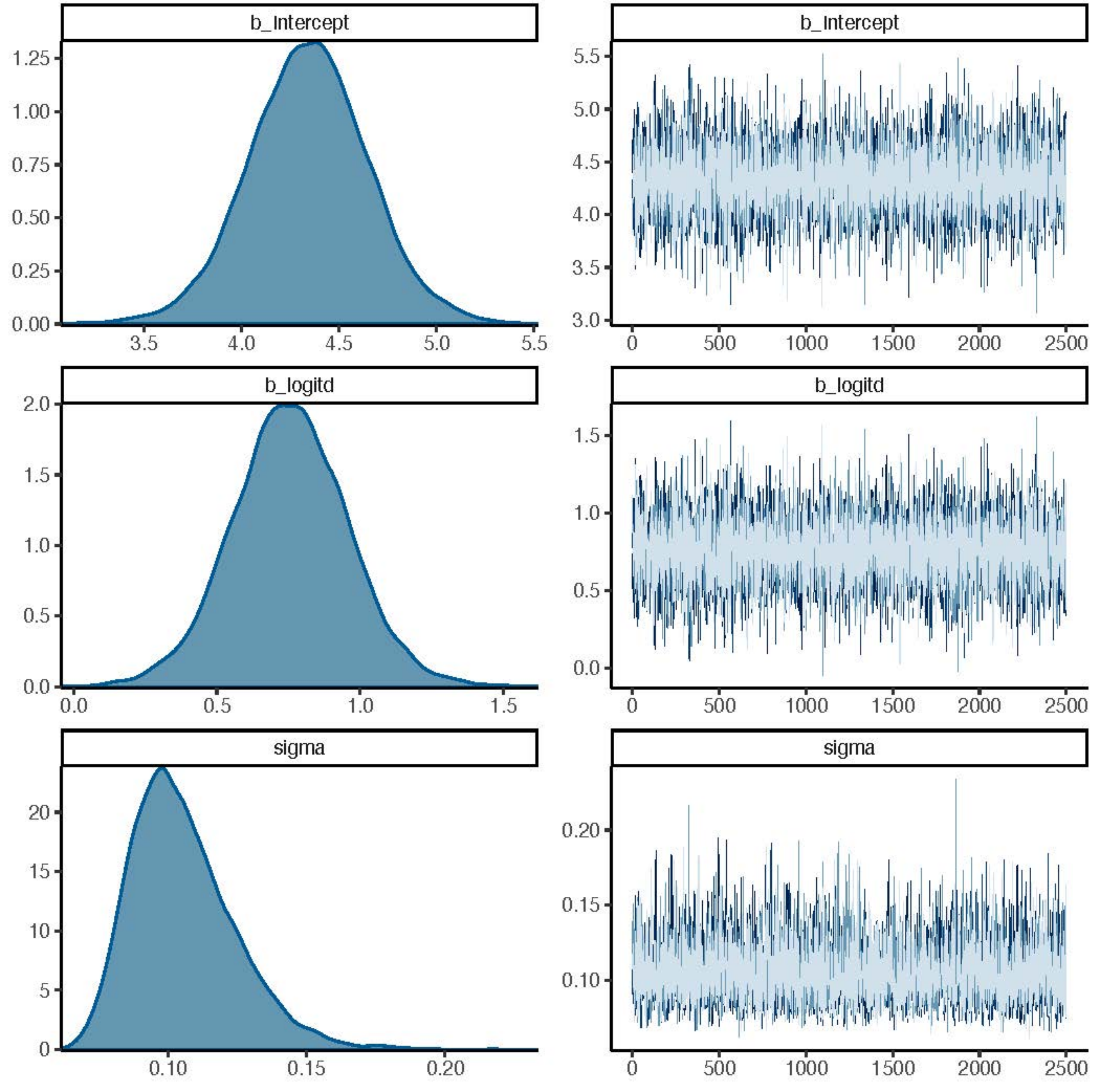
b_Intercept
b_Intercept
b_logitd
b_logitd
sigma
sigma
1.25
1.00
0.75
0.50
0.25
0.00
3.5
4.0
4.5
5.0
5.5
3.0
0
500
1000
1500
2000
2500
2.0
1.5
1.0
0.5
0.0
20
15
10
5
0
0.10
0.15
0.20

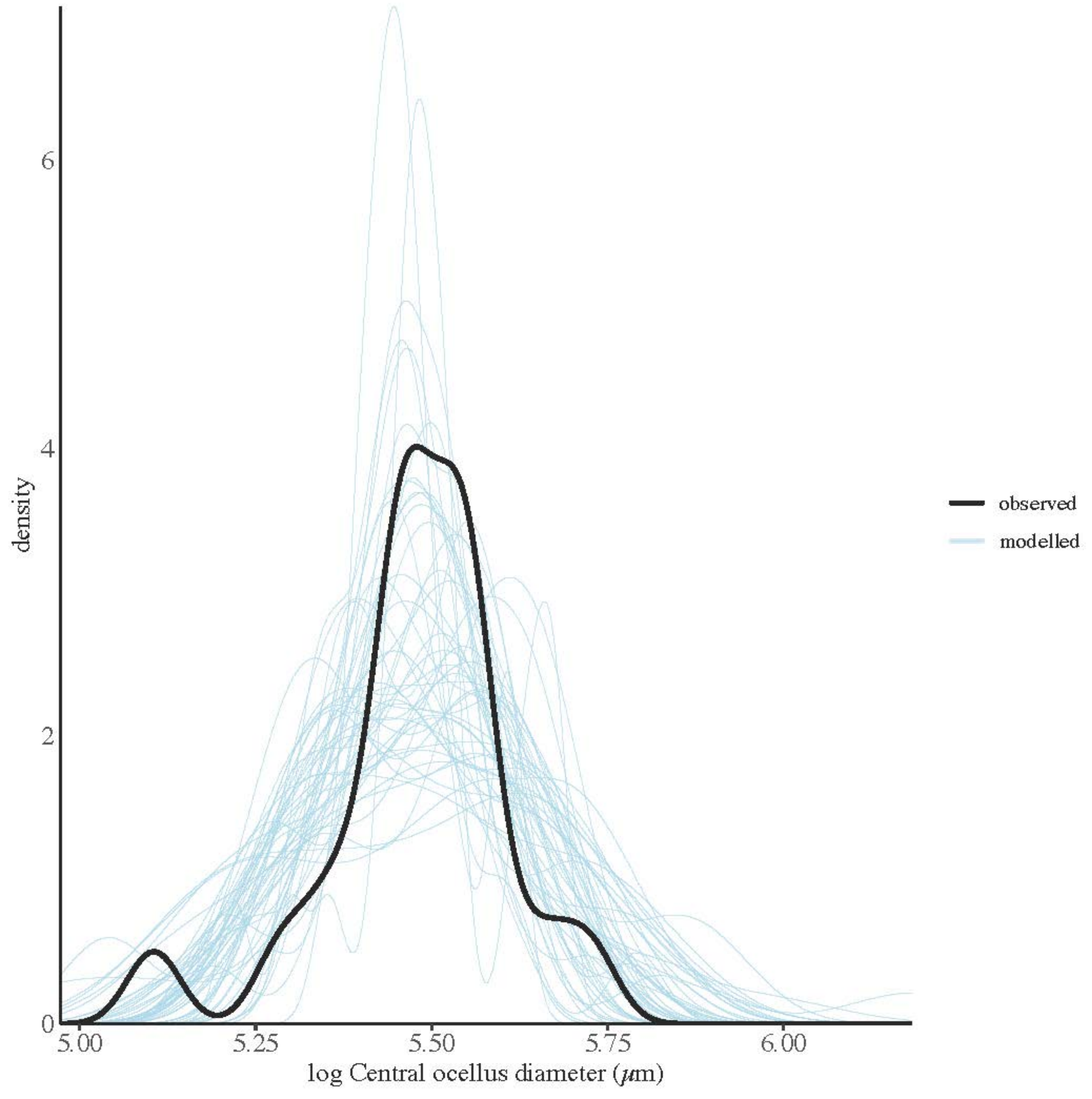

density
6
4
2
0
5.00
5.25
5.50
5.75
6.00
log Central ocellus diameter (μm)
observed
modelled

Lateral ocelli diameter (μm)

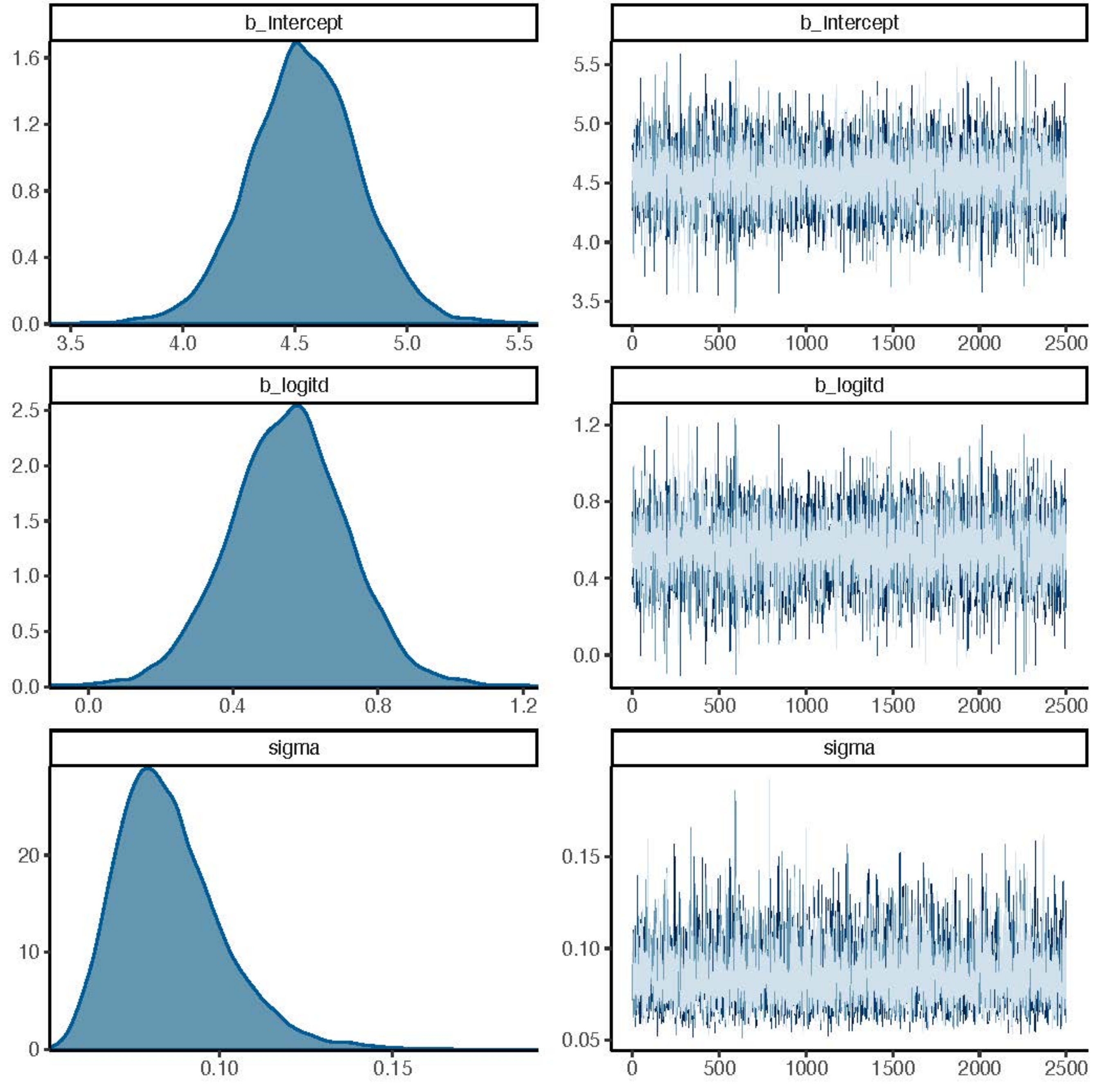

b_Intercept
1.6
1.2
0.8
0.4
0.0
3.5
4.0
4.5
5.0
5.5
b_Intercept
5.5
5.0
4.5
4.0
3.5
0
500
1000
1500
2000
2500
b_logitd
2.5
2.0
1.5
1.0
0.5
0.0
0.0
0.4
0.8
1.2
b_logitd
1.2
0.8
0.4
0.0
0
500
1000
1500
2000
2500
sigma
20
10
0
0.10
0.15
sigma
0.15
0.10
0.05
0
500
1000
1500
2000
2500

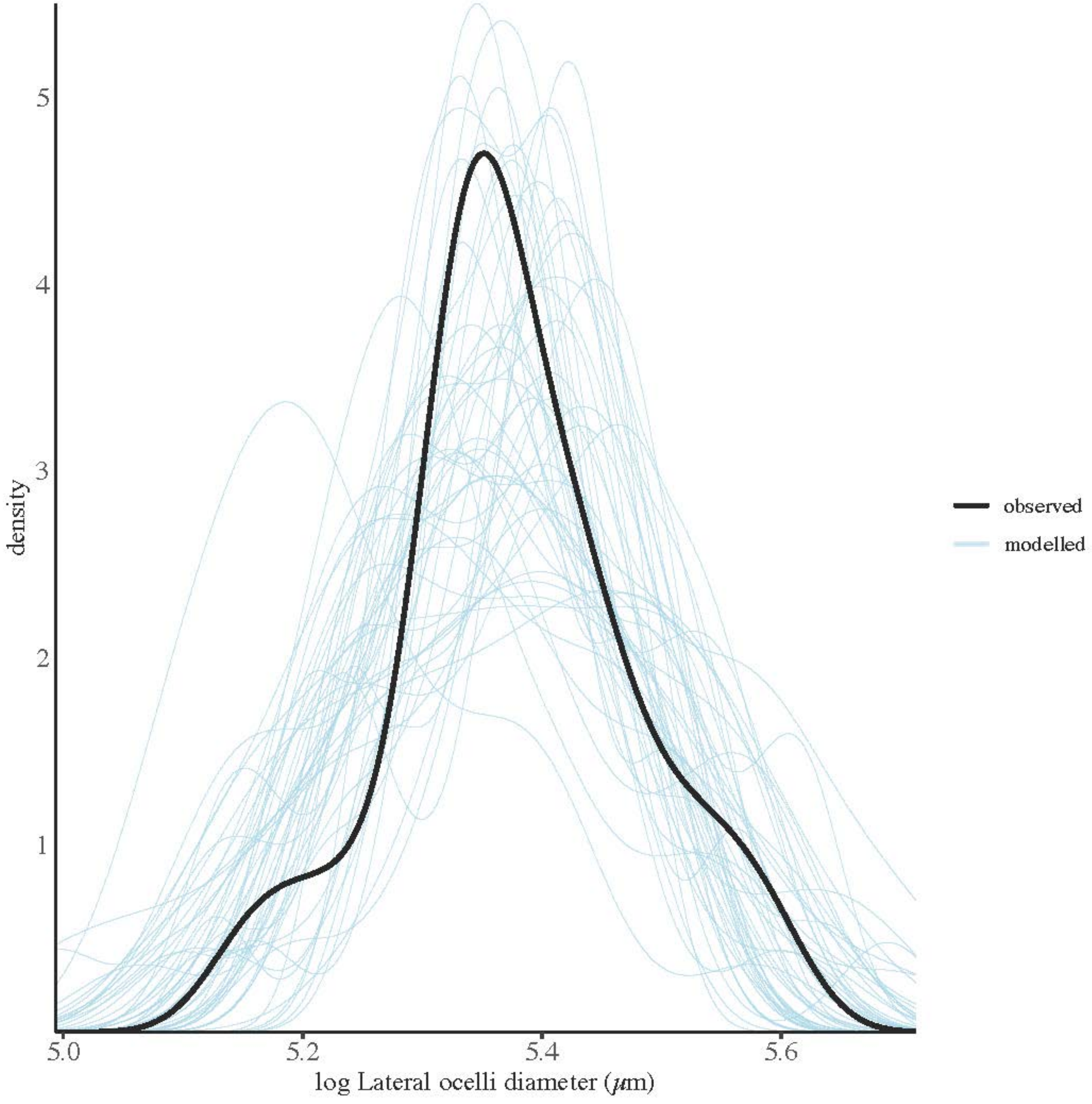

density
5
4
3
2
1
5.0
5.2
5.4
5.6
log Lateral ocelli diameter (μm)
observed
modelled

Ocellar alignment (°)

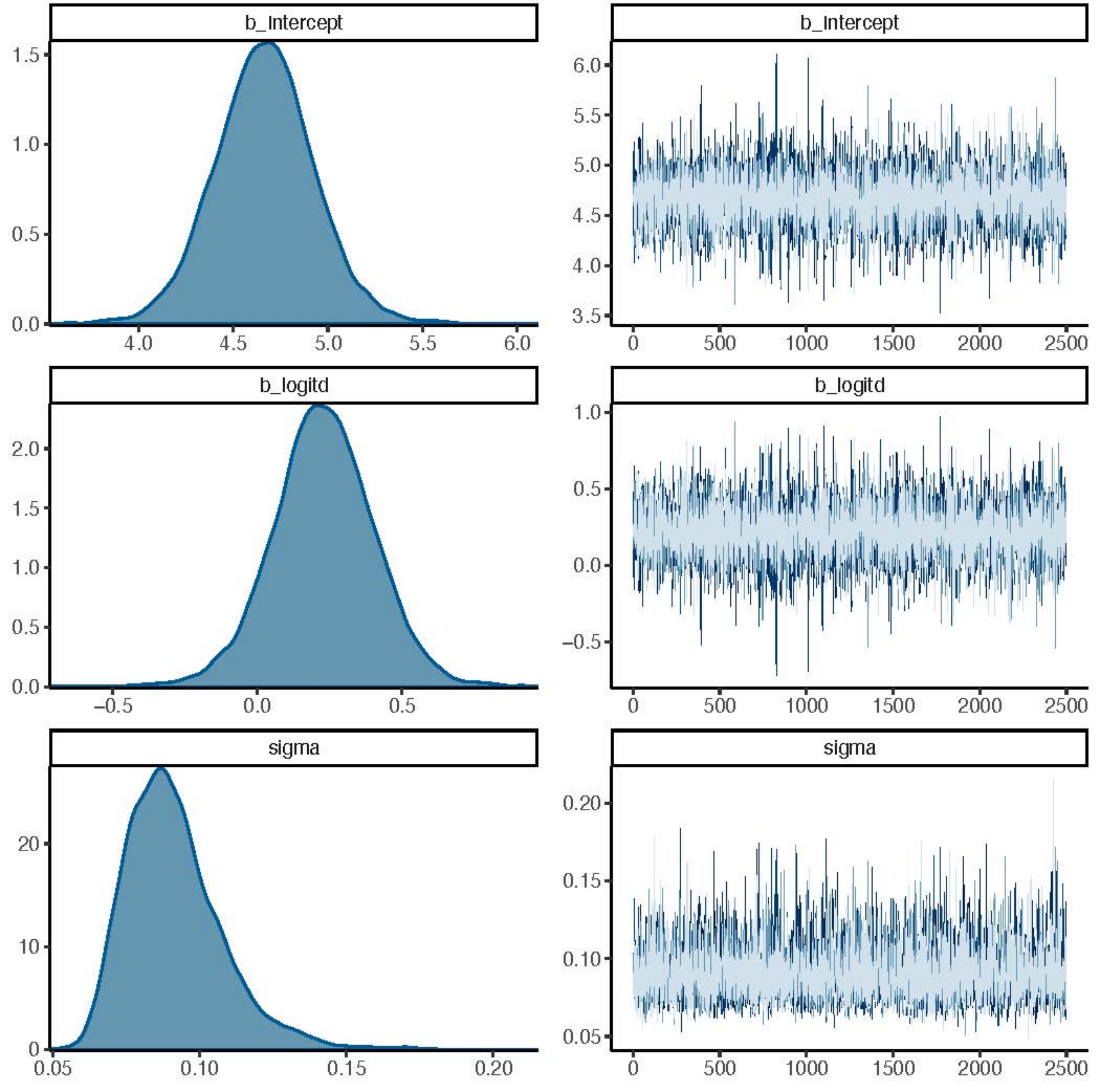

b_Intercept
b_Intercept
b_logitd
b_logitd
sigma
sigma

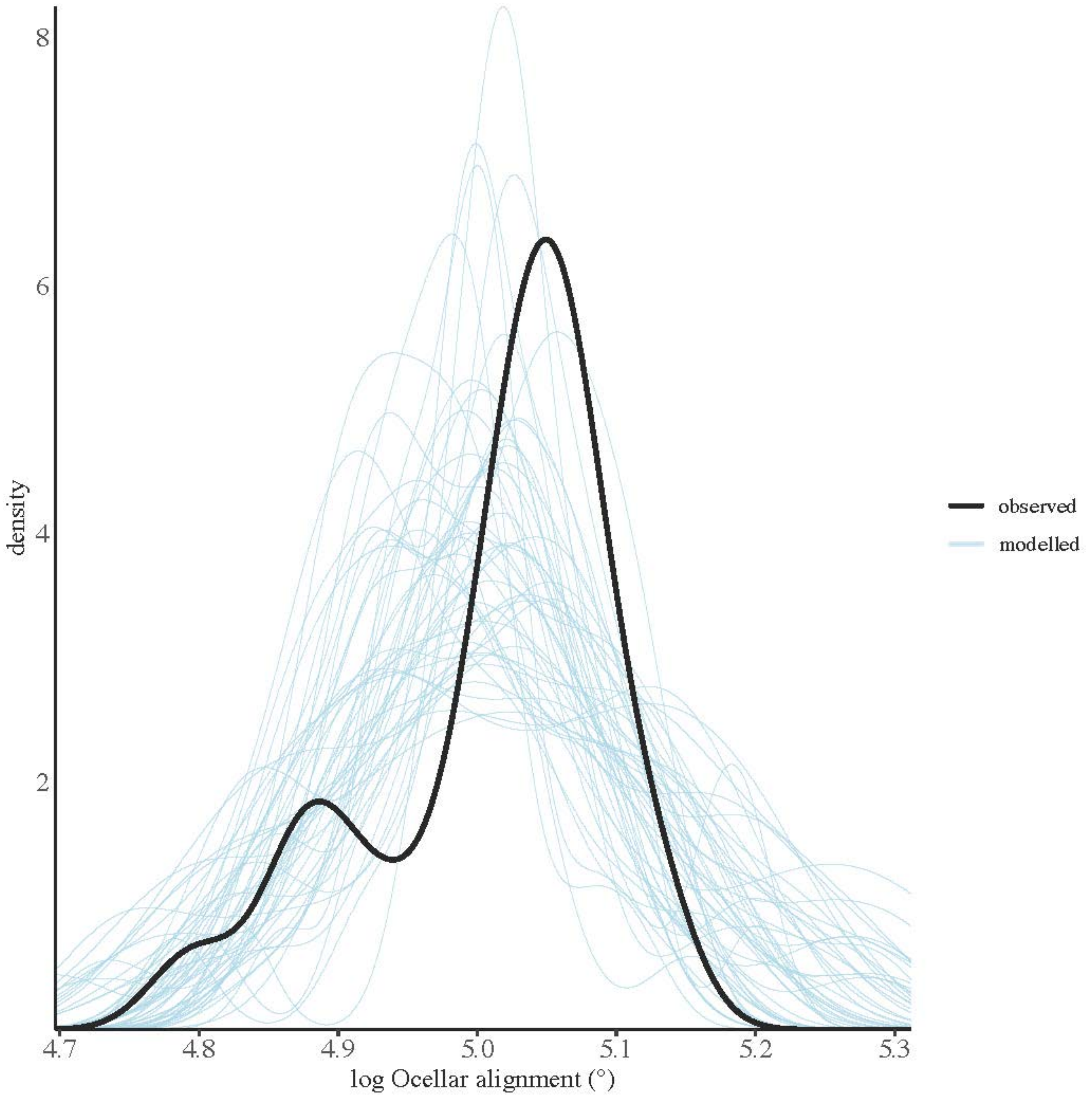

8
6
4
2
density
4.7
4.8
4.9
5.0
5.1
5.2
5.3
log Ocellar alignment (°)
observed
modelled

Monocular FOV (%)

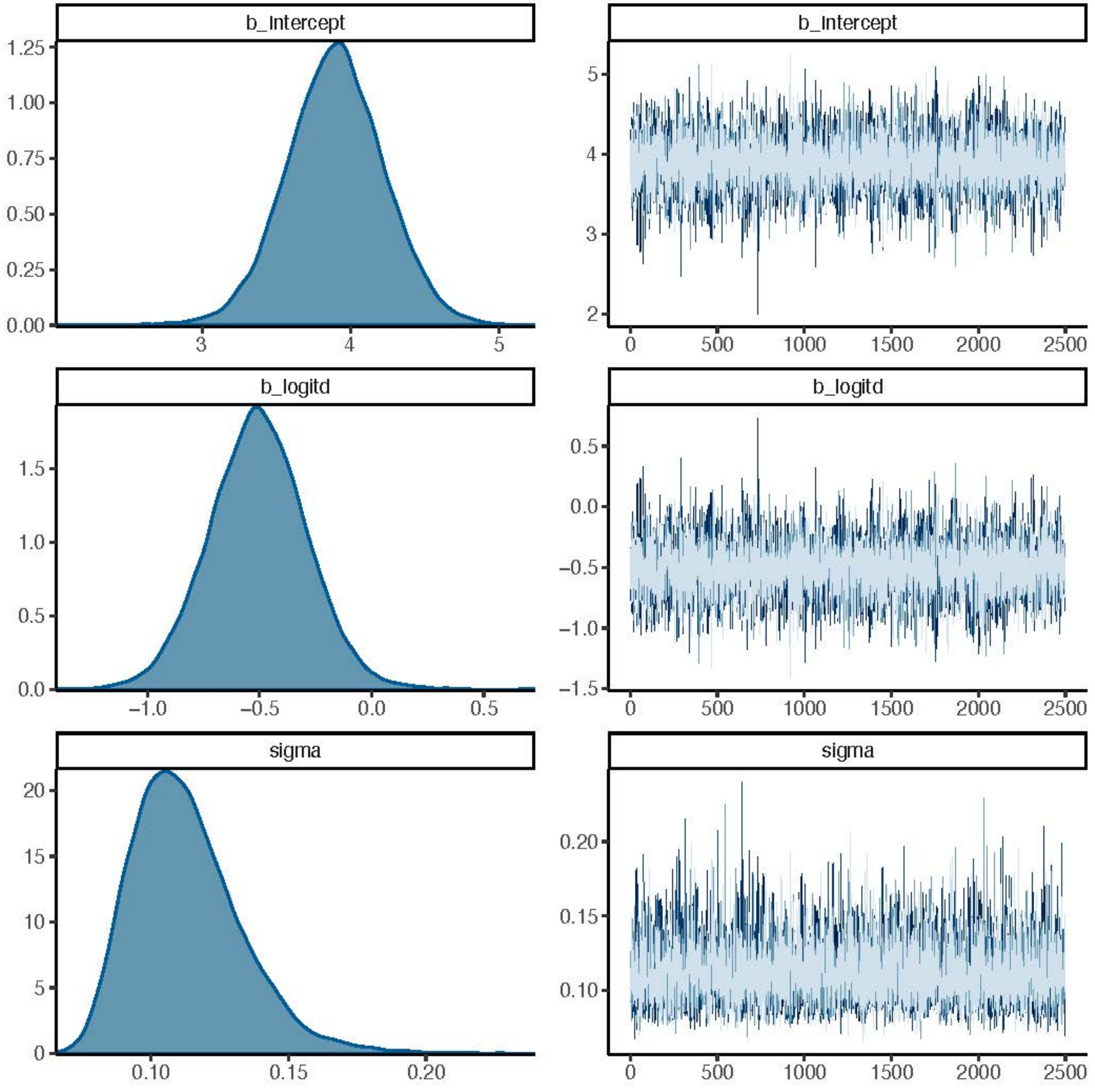

b_Intercept
b_Intercept
b_logitd
b_logitd
sigma
sigma
1.25
1.00
0.75
0.50
0.25
0.00
3
4
5
2
0
500
1000
1500
2000
2500
1.5
1.0
0.5
0.0
−1.0
−0.5
−1.5
20
15
10
0.10
0.15
0.20

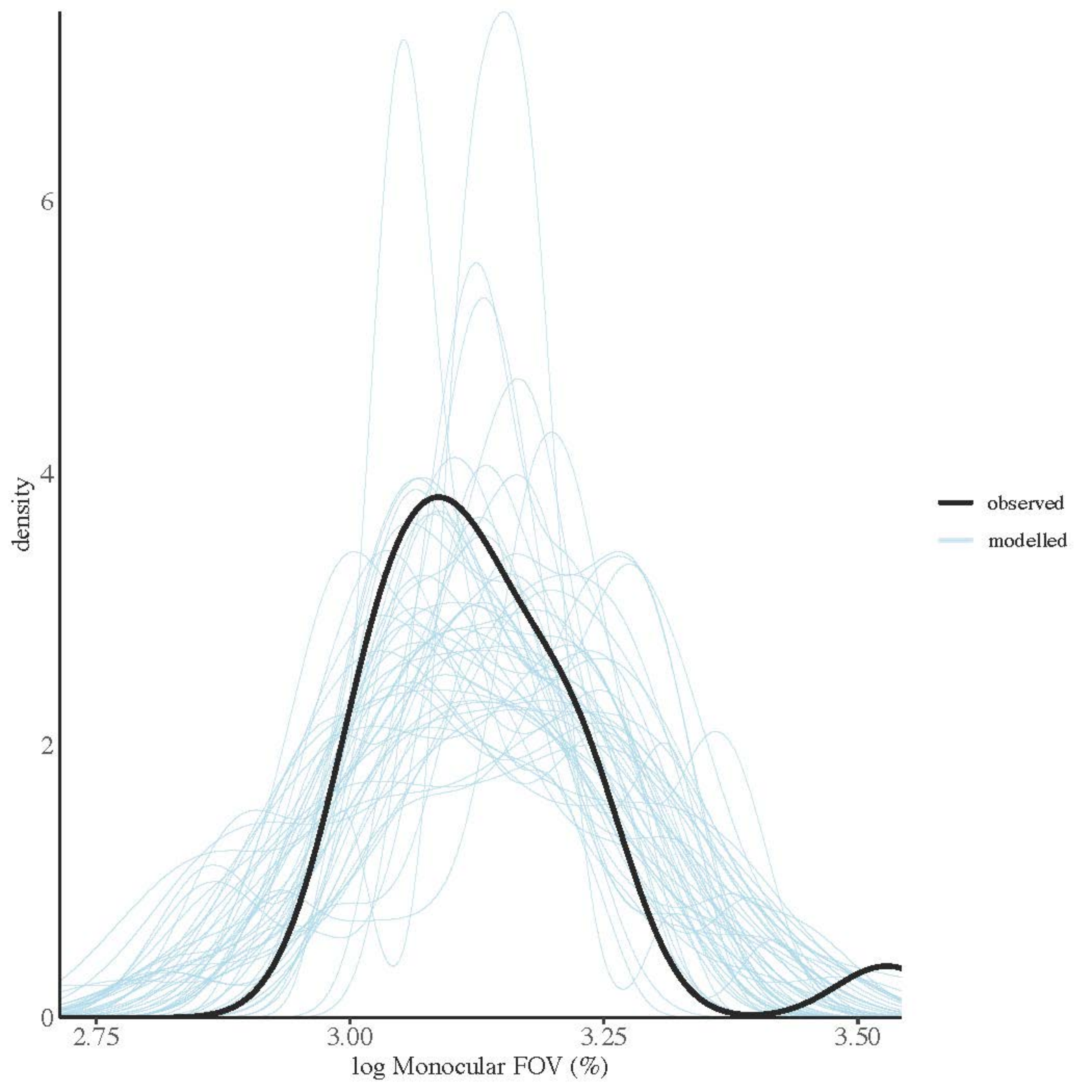

density
6
4
2
0
2.75
3.00
3.25
3.50
log Monocular FOV (%)
observed
modelled

Binocular overlap (%)

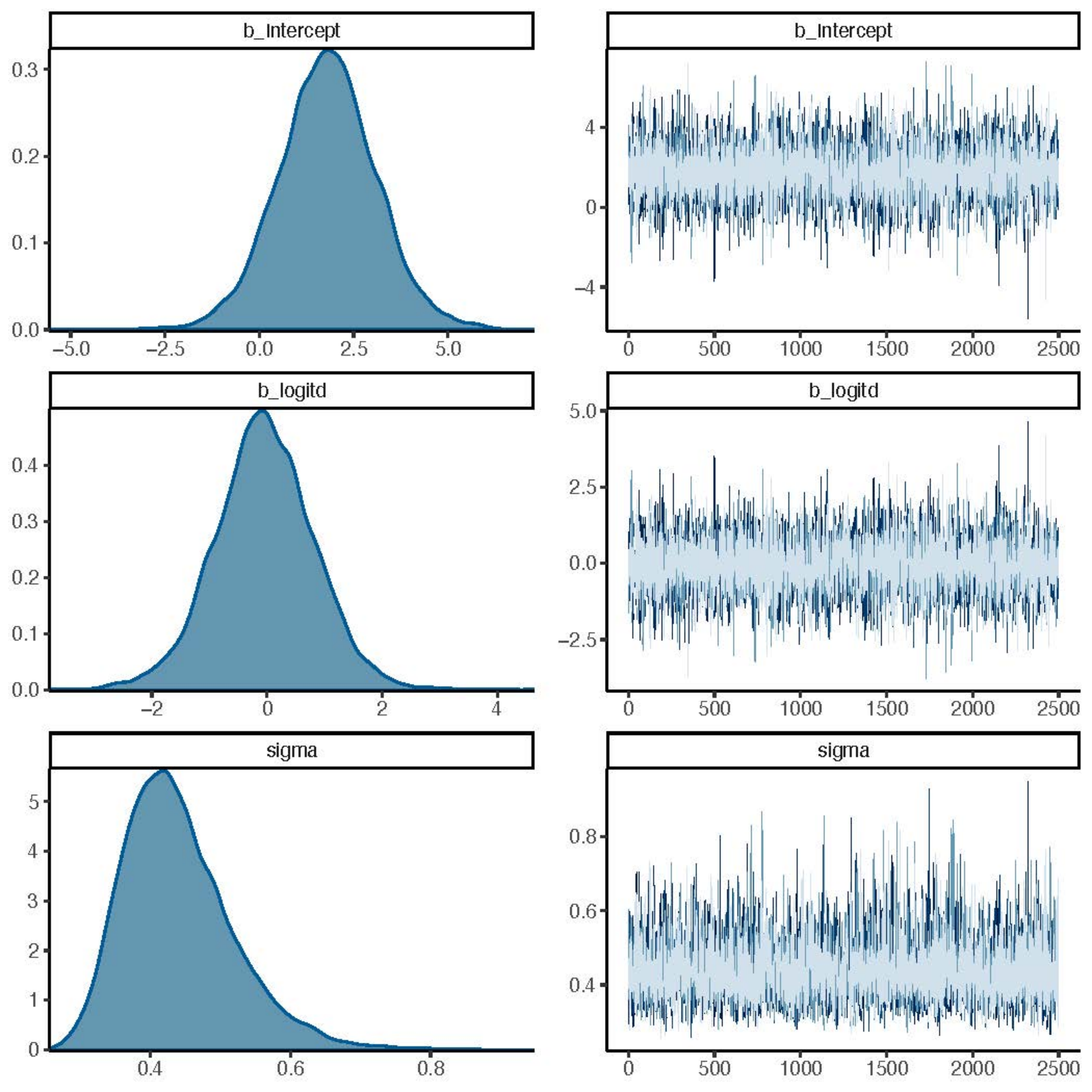

b_Intercept
0.3
0.2
0.1
0.0
−5.0
−2.5
0.0
2.5
5.0
b_Intercept
4
0
−4
0
500
1000
1500
2000
2500
b_logitd
0.4
0.3
0.2
0.1
0.0
−2
0
2
4
b_logitd
5.0
2.5
0.0
−2.5
0
500
1000
1500
2000
2500
sigma
5
4
3
2
1
0
0.4
0.6
0.8
sigma
0.8
0.6
0.4
0
500
1000
1500
2000
2500

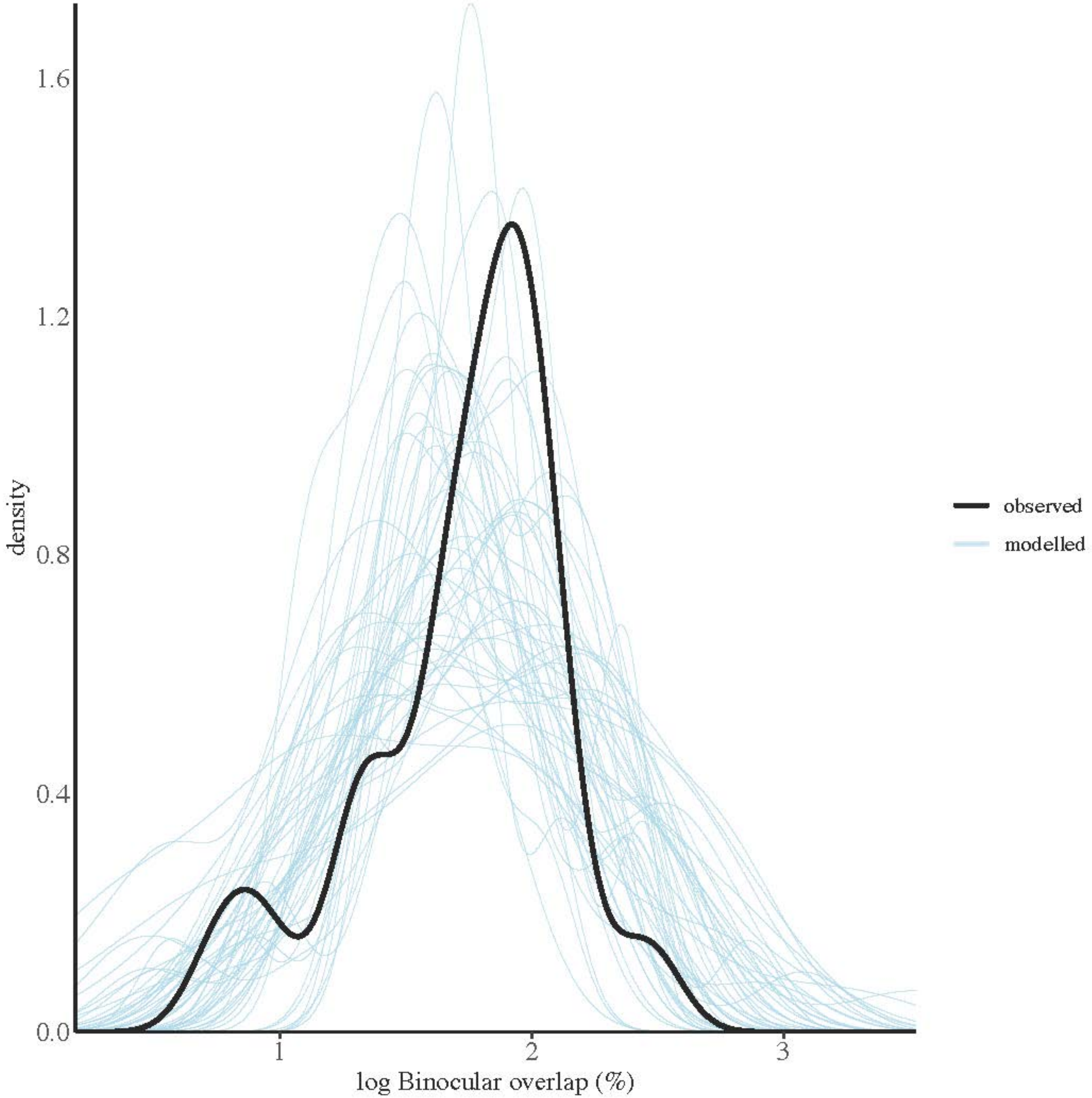
1.6
1.2
0.8
0.4
0.0
density
1
2
3
log Binocular overlap (%)
observed
modelled

Effects of tree cover on the community weighted means of visual traits and ITD in bumblebee communities within grasslands across Sweden. Model traces and posterior predictive check for each visual trait modelled with Bayesian inference.

Eye surface (μm^2)

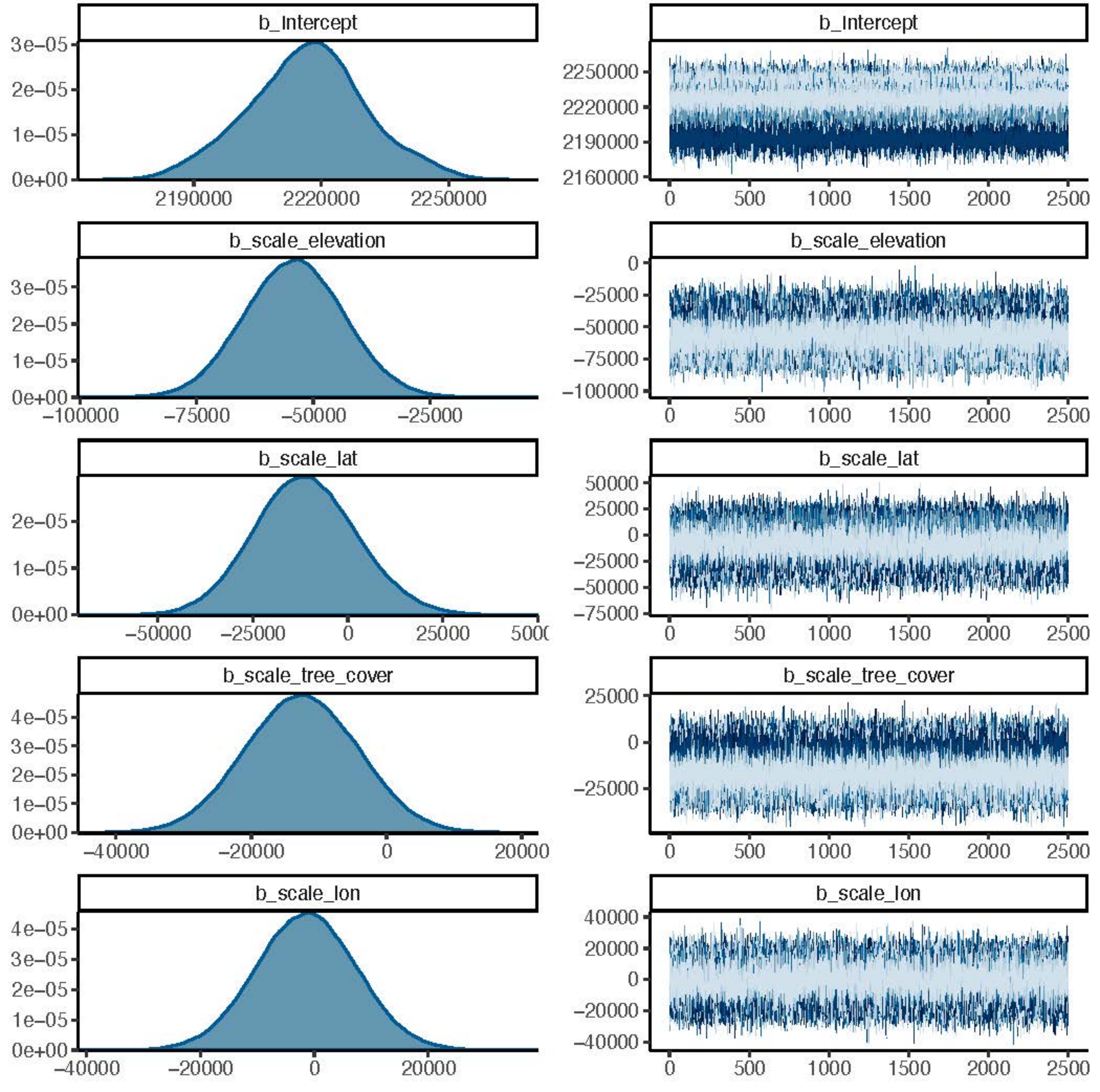
b_Intercept
b_scale_elevation
b_scale_lat
b_scale_tree_cover
b_scale_lon

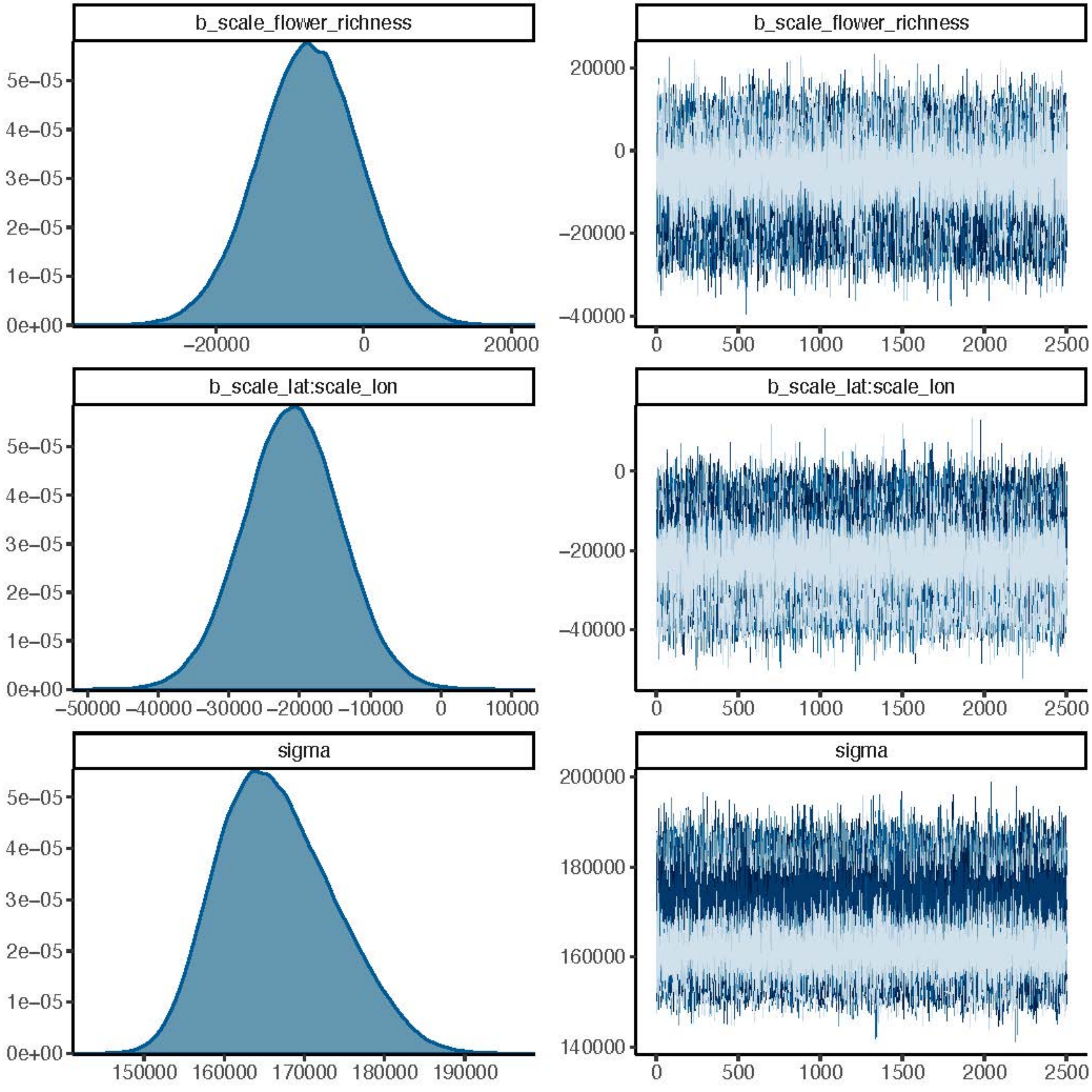
b_scale_flower_richness
b_scale_flower_richness
b_scale_lat:scale_lon
b_scale_lat:scale_lon
sigma
sigma
5e−05
4e−05
3e−05
2e−05
1e−05
0e+00
−20000
0
20000
−40000
500
1000
1500
2000
2500
−50000
−30000
−10000
10000
150000
160000
170000
180000
190000
200000
140000

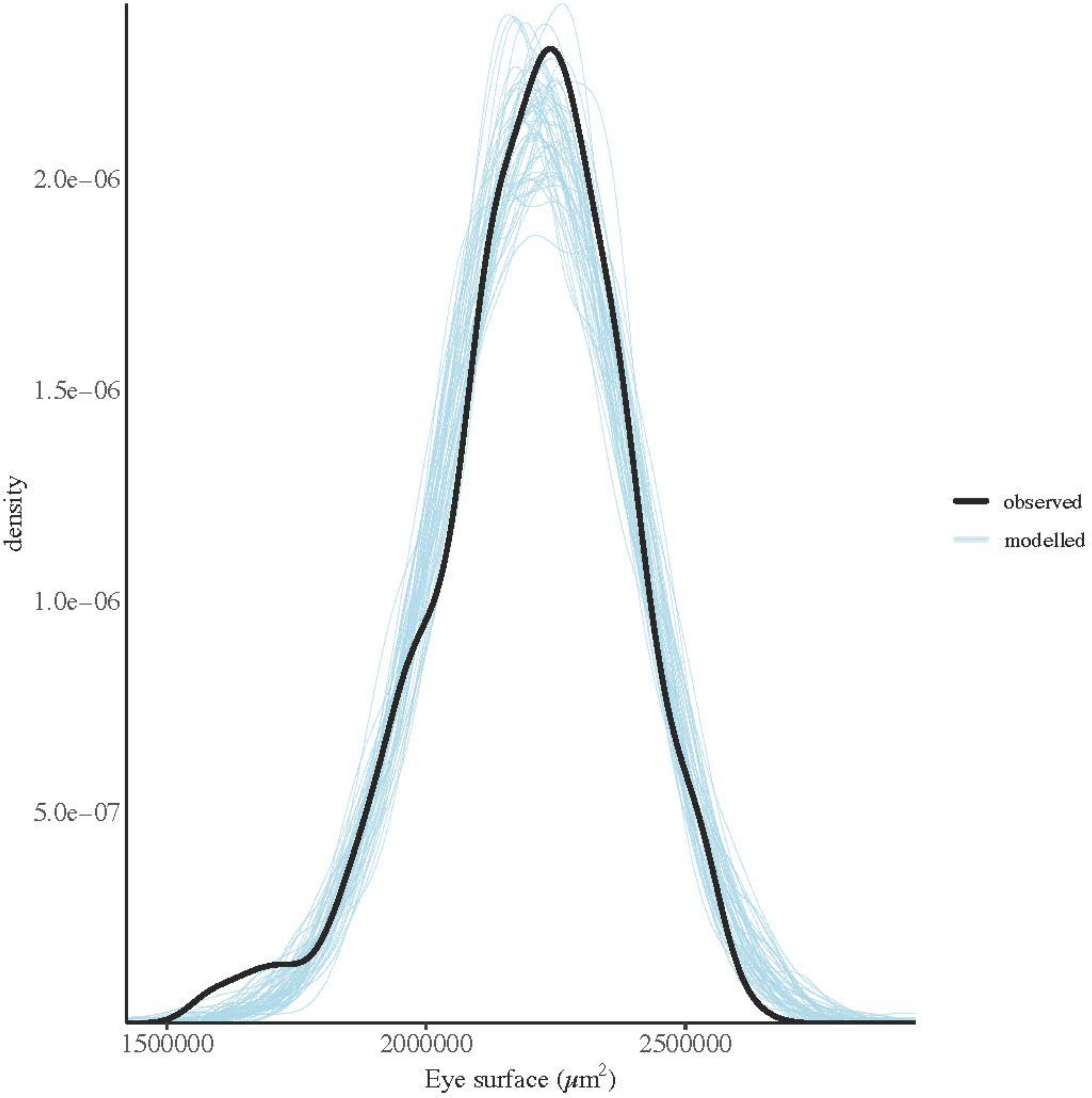

2.0e−06
1.5e−06
1.0e−06
5.0e−07
density
1500000
2000000
2500000
Eye surface (μm^2)
observed
modelled

Facet diameter (μm)

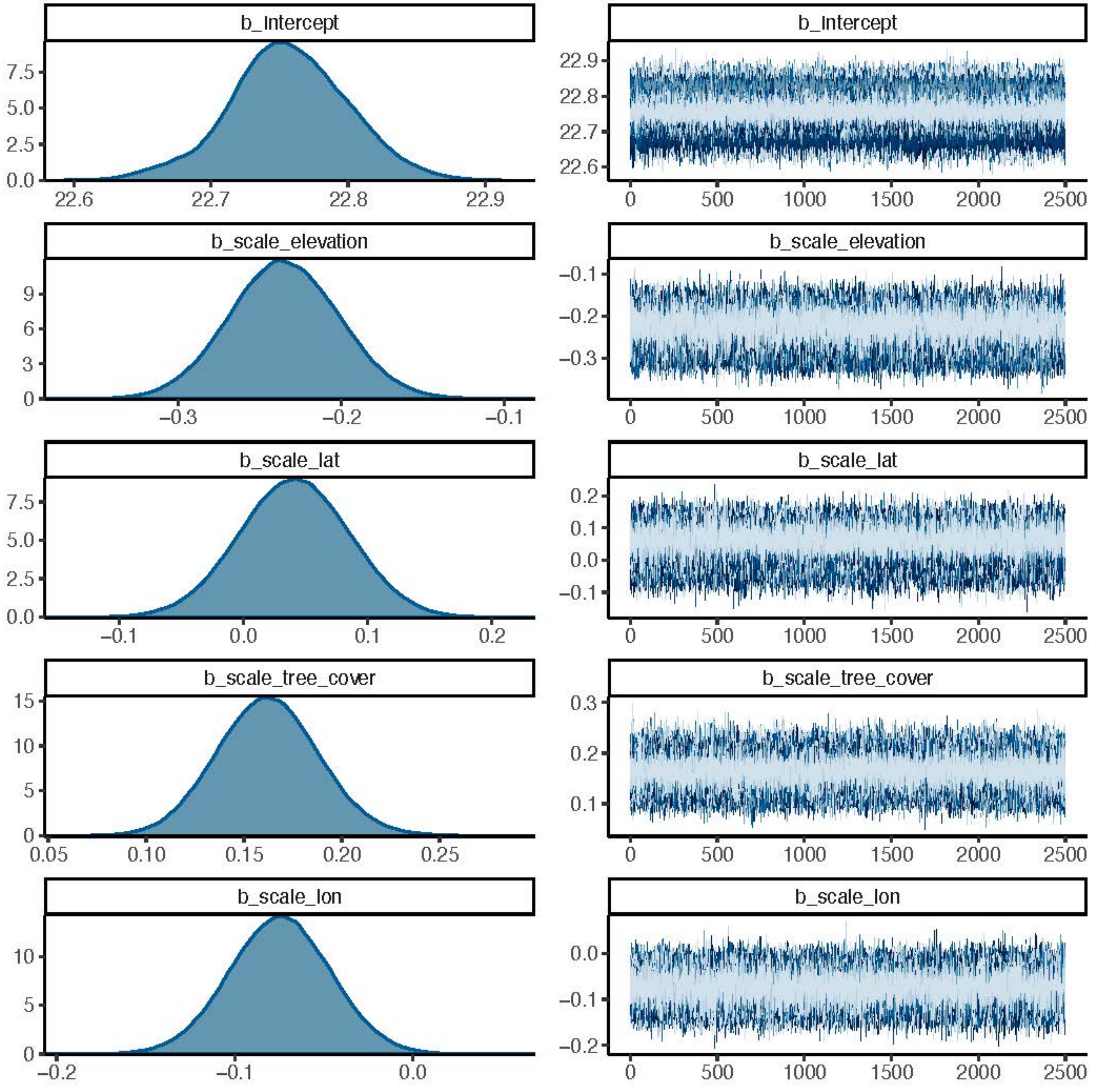
b_Intercept
b_scale_elevation
b_scale_lat
b_scale_tree_cover
b_scale_lon

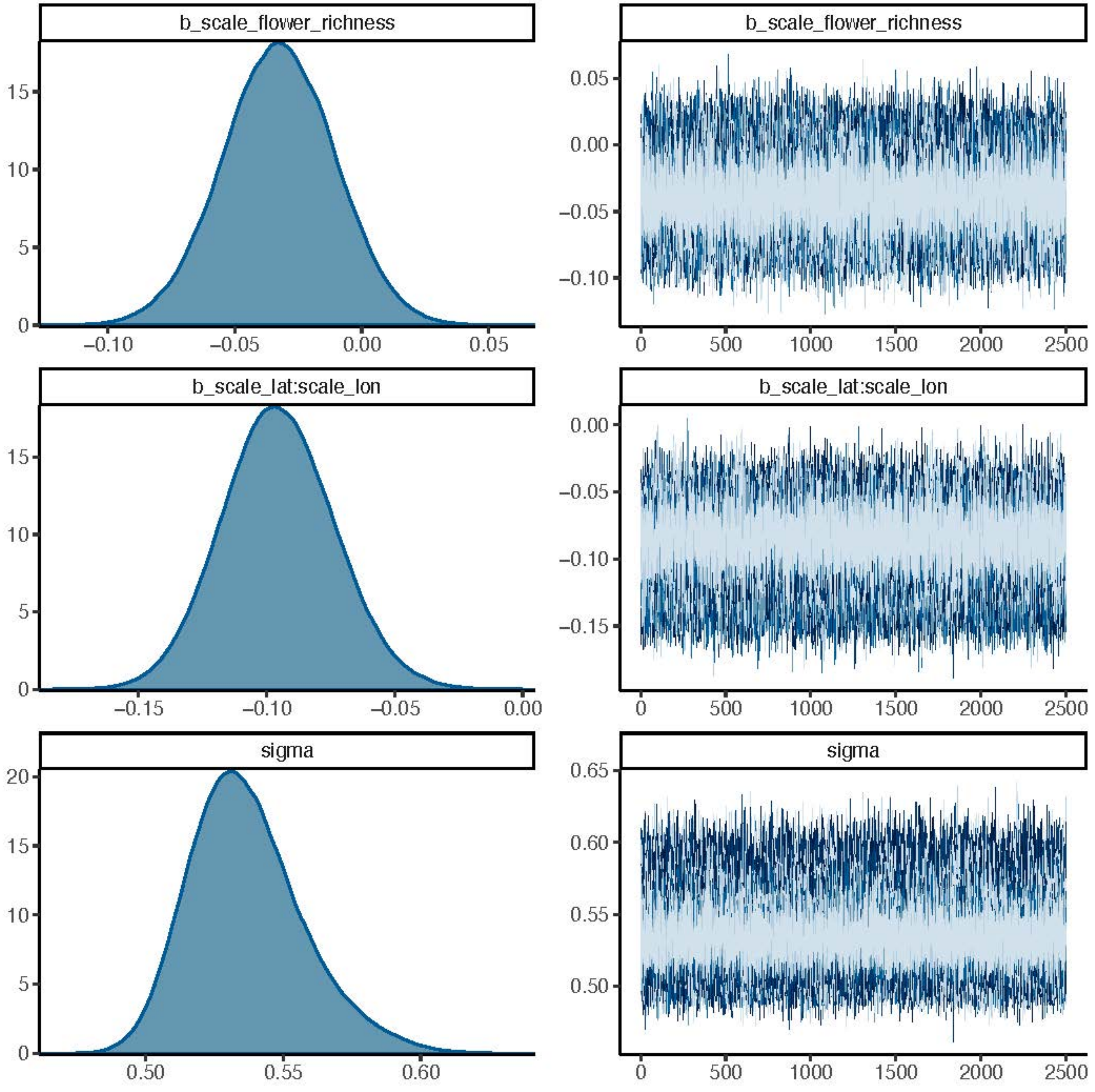

b_scale_flower_richness
b_scale_flower_richness
b_scale_lat:scale_lon
b_scale_lat:scale_lon
sigma
sigma

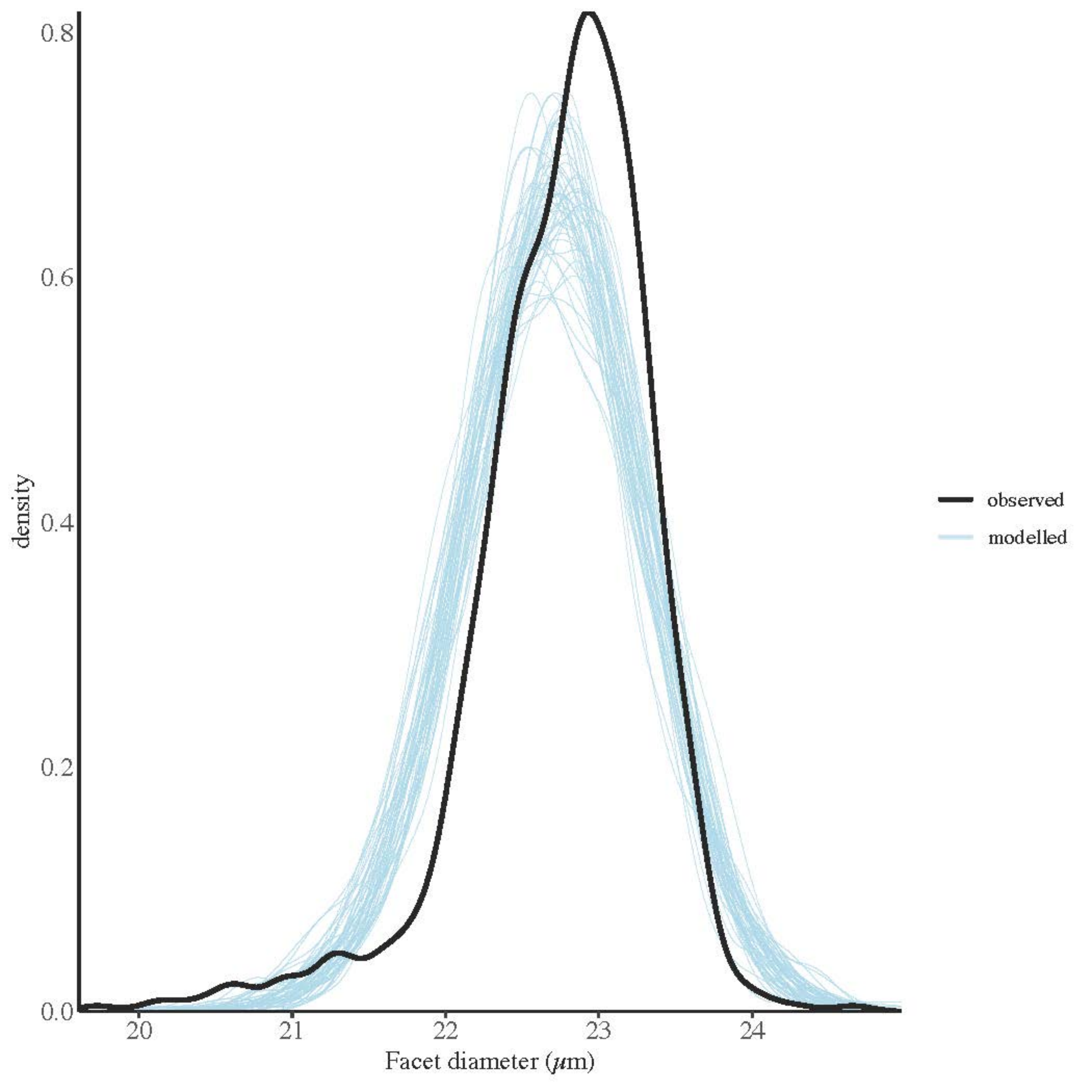
0.8
0.6
0.4
0.2
0.0
density
20
21
22
23
24
Facet diameter (μm)
observed
modelled

Inter–ommatidial angle (°)

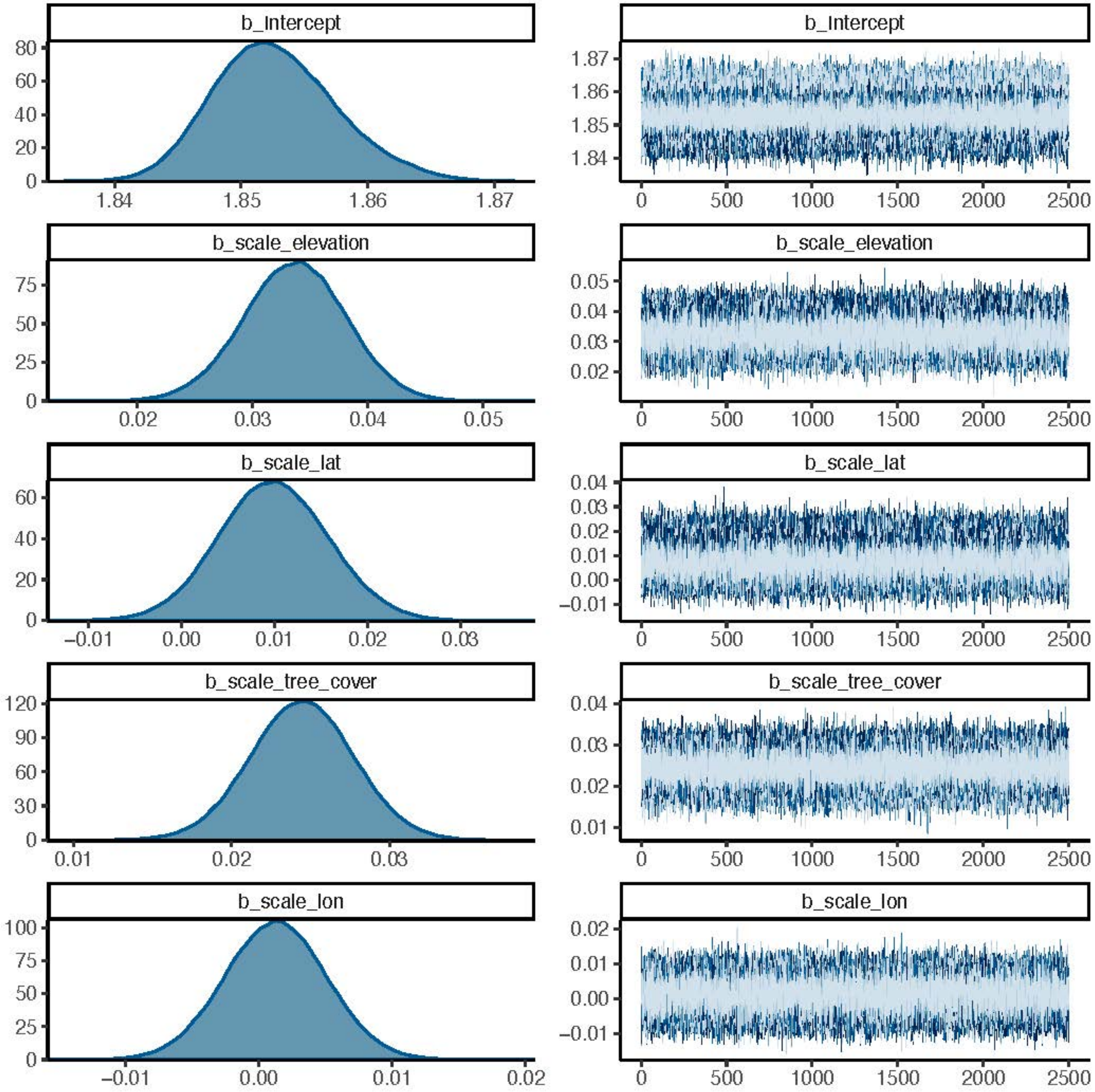
b_Intercept
b_scale_elevation
b_scale_lat
b_scale_tree_cover
b_scale_lon

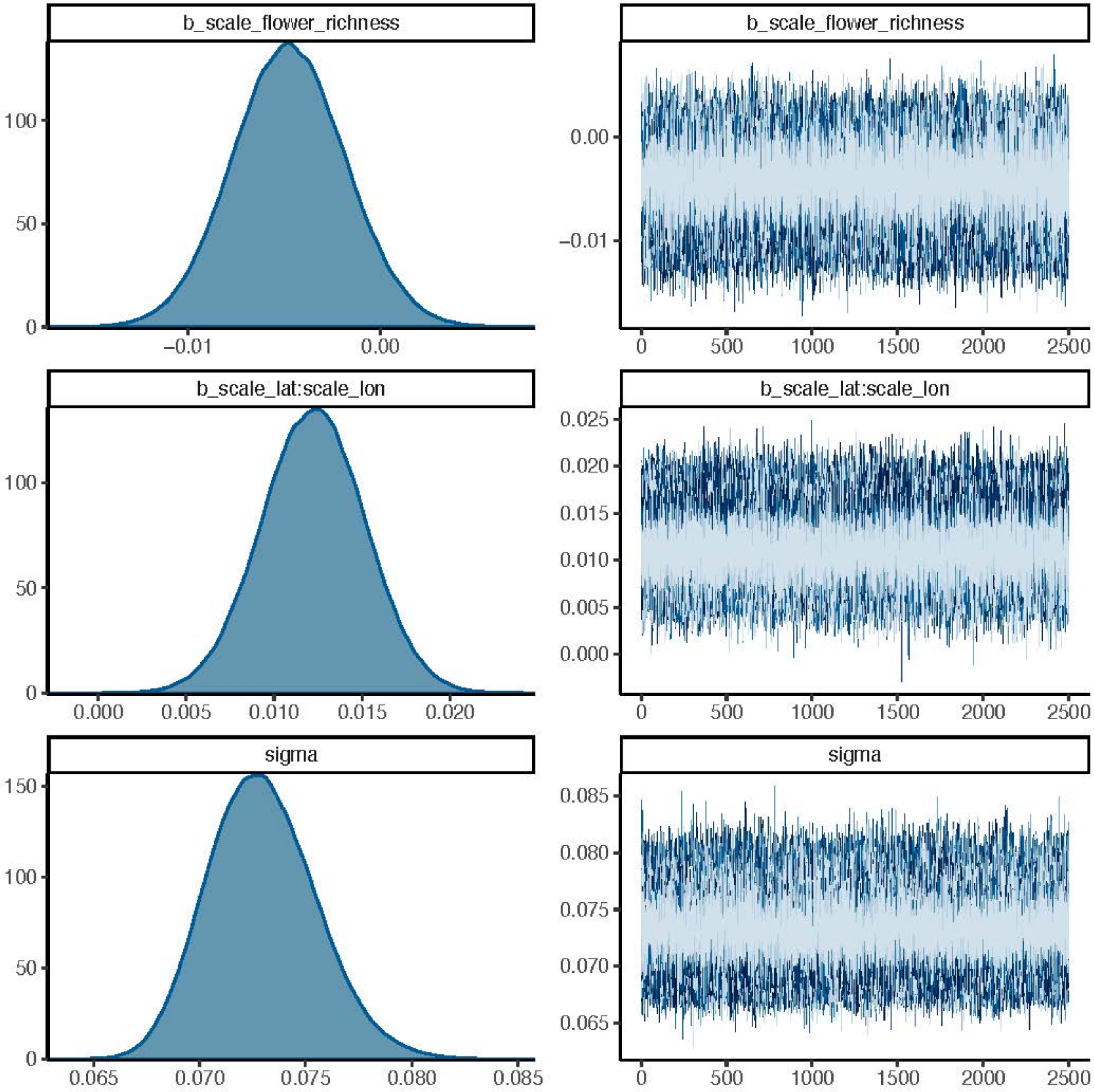
b_scale_flower_richness
b_scale_flower_richness
b_scale_lat:scale_lon
b_scale_lat:scale_lon
sigma
sigma
0
50
100
150
−0.01
0.00
0.000
0.005
0.010
0.015
0.020
0.025
0.065
0.070
0.075
0.080
0.085
500
1000
1500
2000
2500

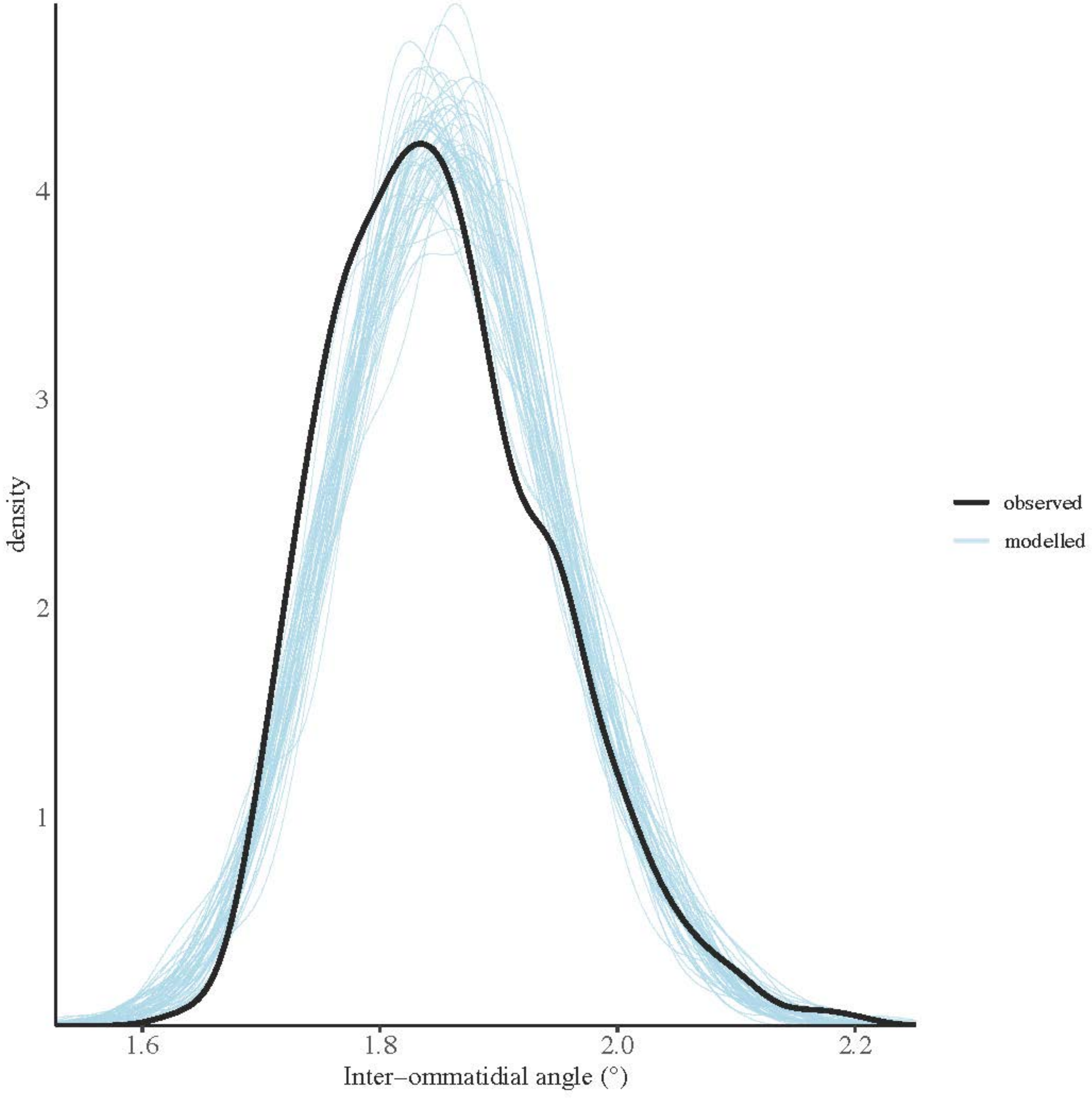

density
4
3
2
1
1.6
1.8
2.0
2.2
Inter–ommatidial angle (°)
observed
modelled

Facet number

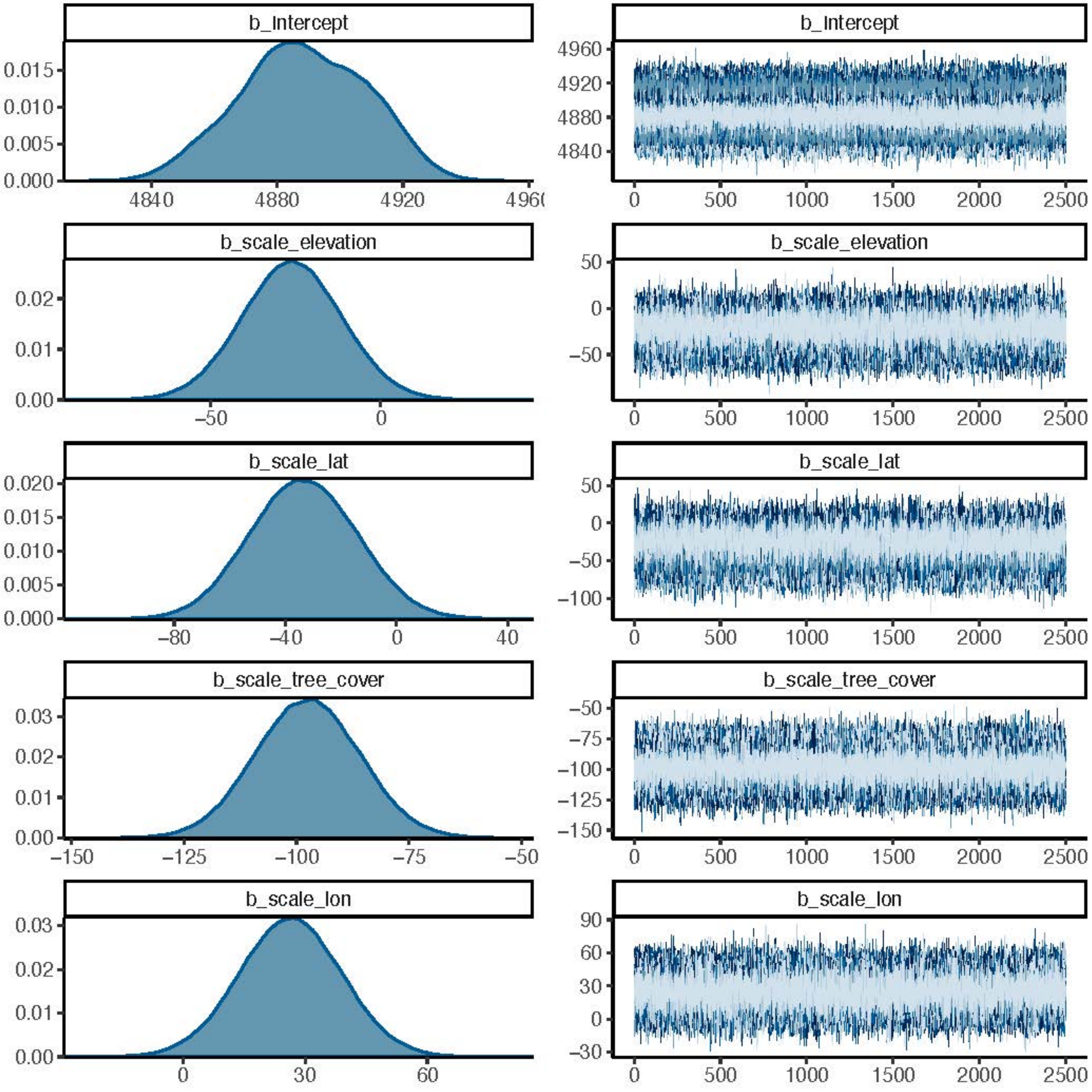

b_Intercept
b_Intercept
b_scale_elevation
b_scale_elevation
b_scale_lat
b_scale_lat
b_scale_tree_cover
b_scale_tree_cover
b_scale_lon
b_scale_lon

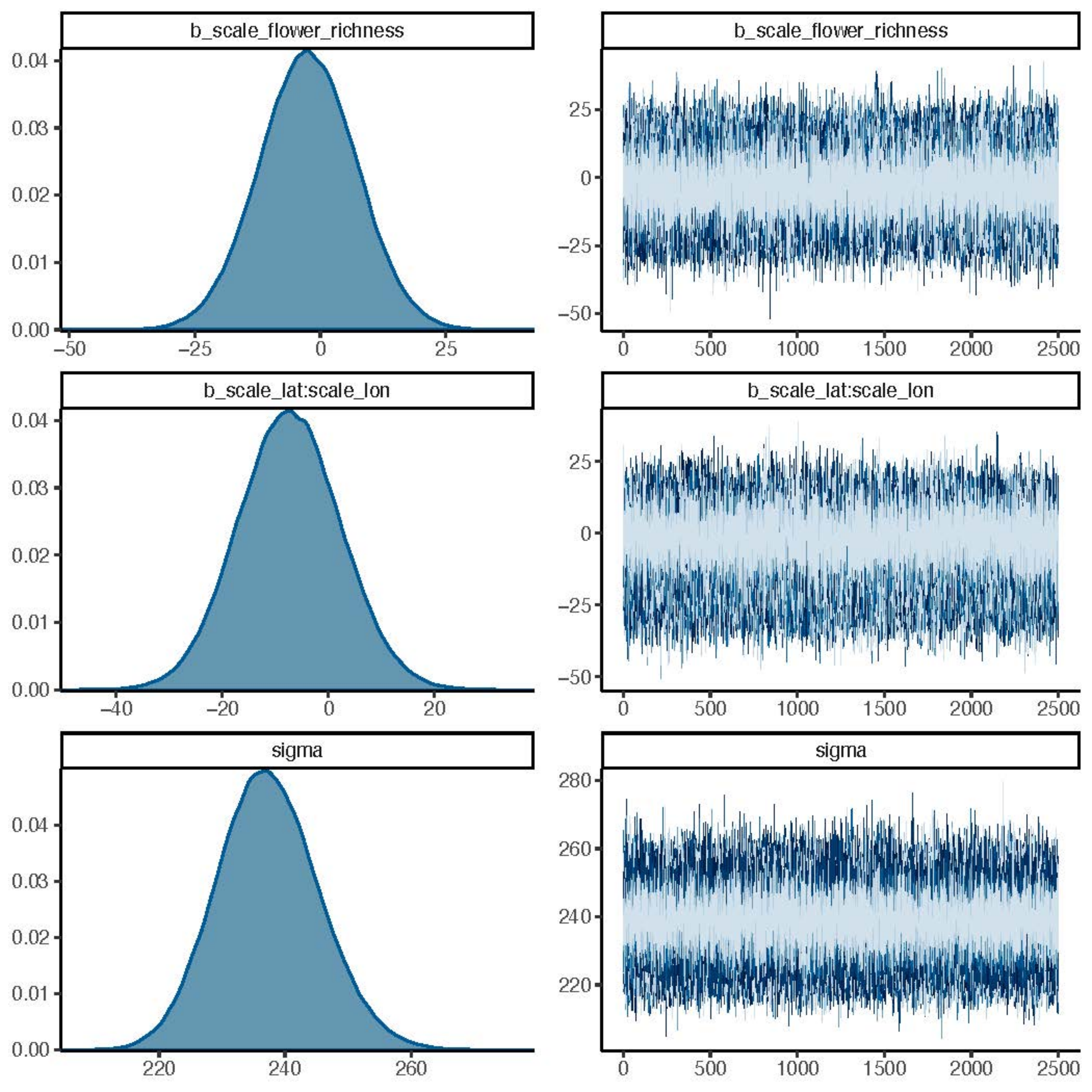
b_scale_flower_richness
b_scale_flower_richness
b_scale_lat:scale_lon
b_scale_lat:scale_lon
sigma
sigma

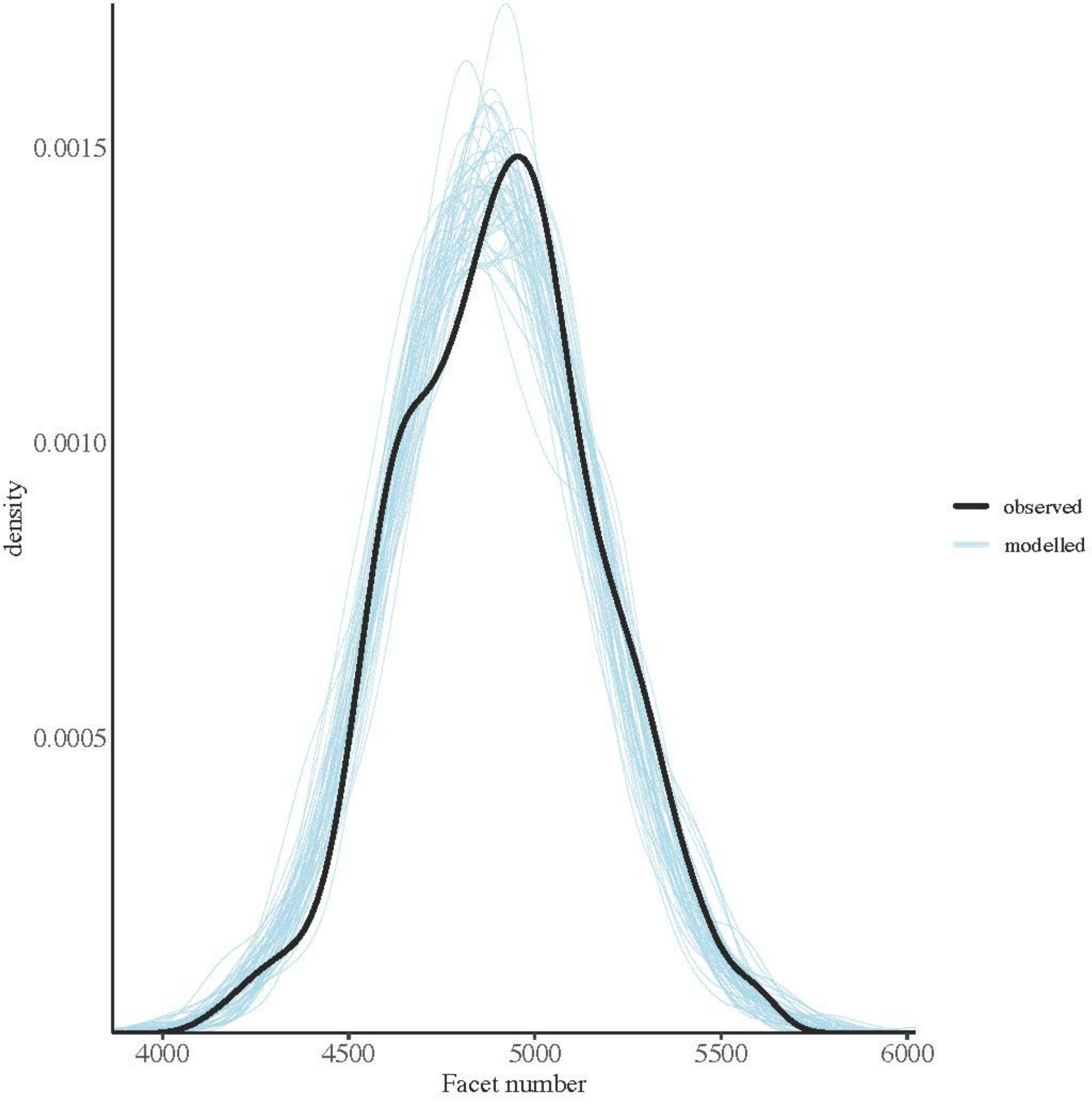

0.0015
0.0010
0.0005
density
4000
4500
5000
5500
6000
Facet number
observed
modelled

Eye parameter (μm.rad)

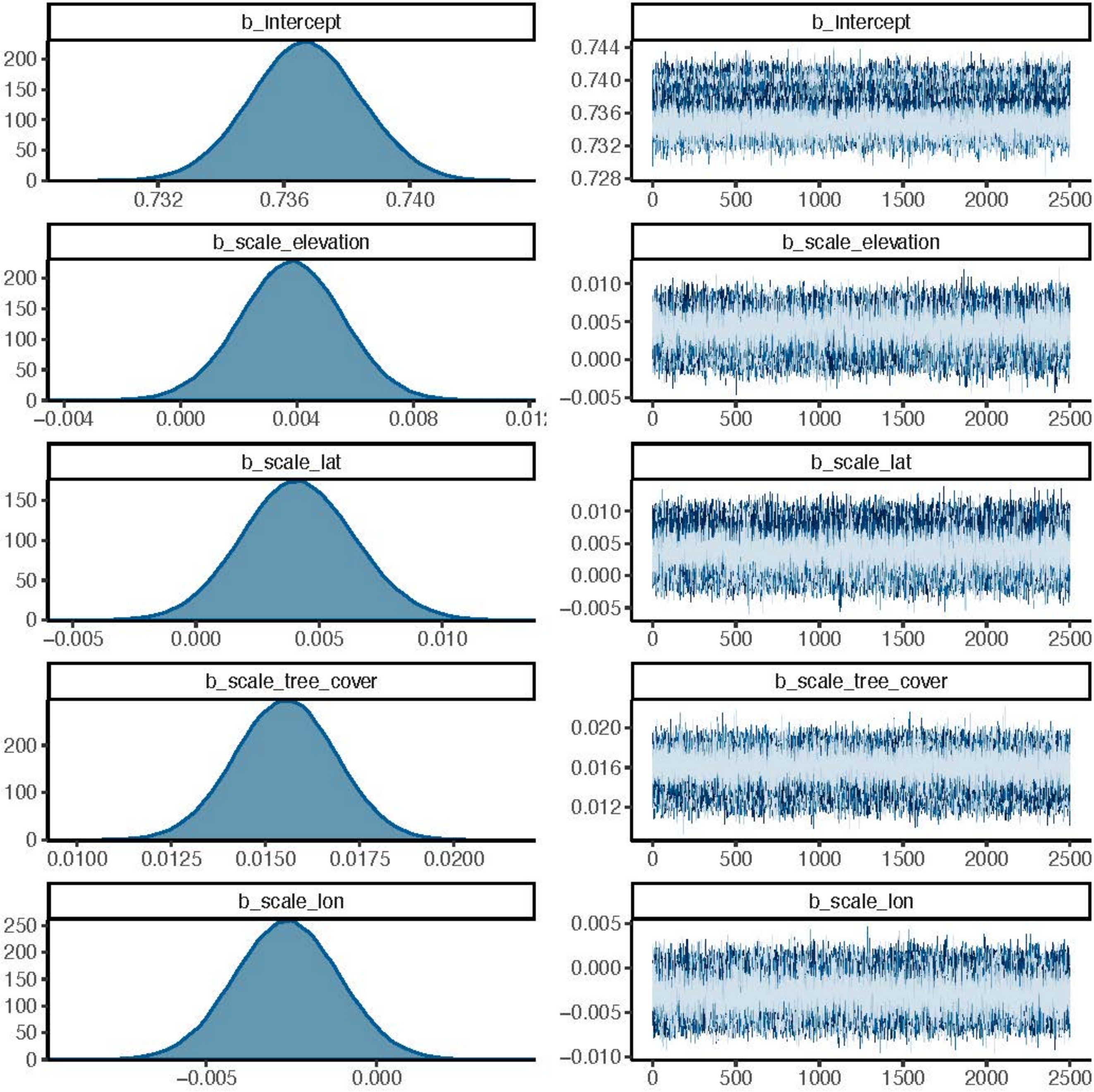

b_Intercept
b_scale_elevation
b_scale_lat
b_scale_tree_cover
b_scale_lon

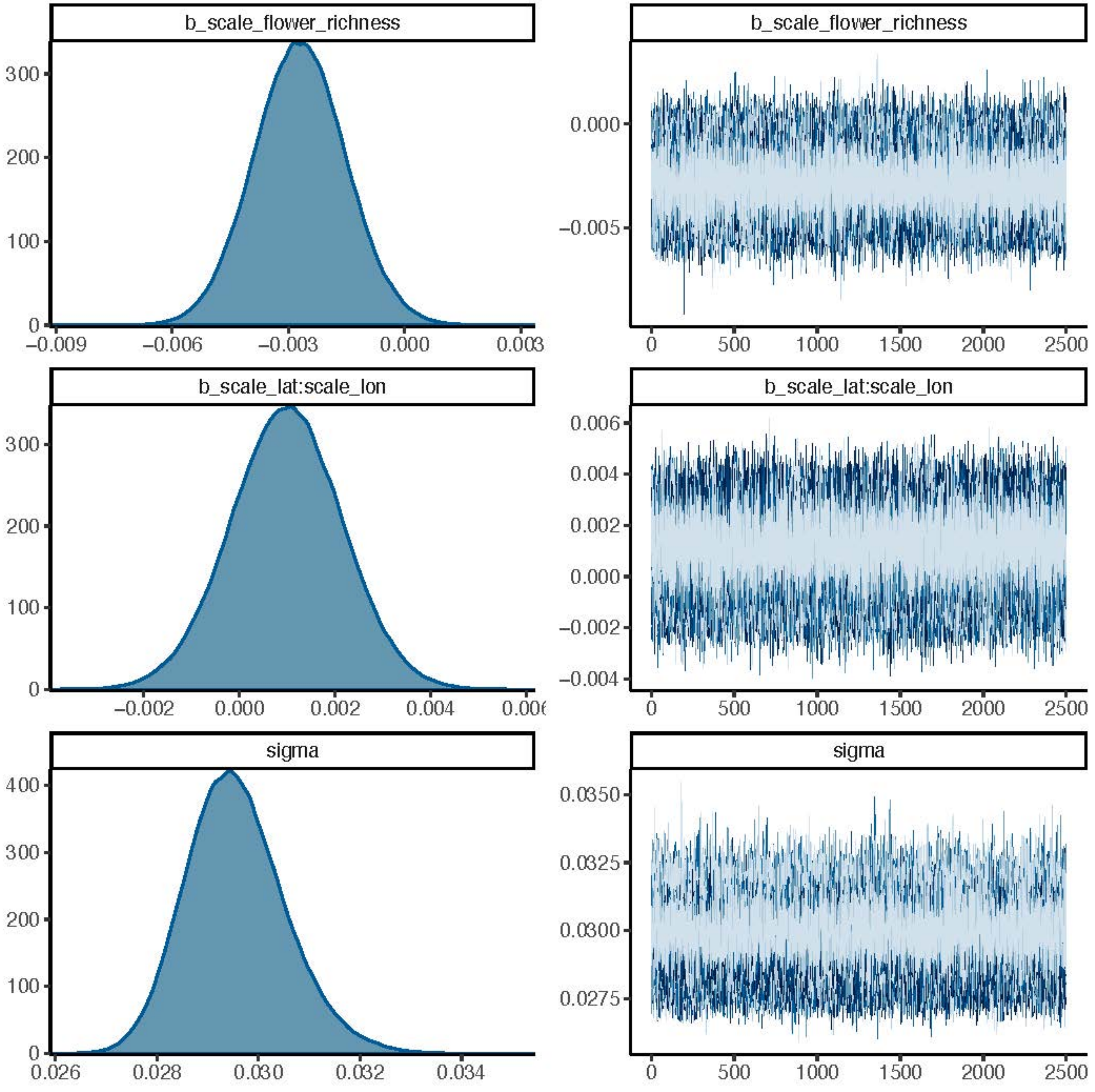

b_scale_flower_richness
b_scale_flower_richness
b_scale_lat:scale_lon
b_scale_lat:scale_lon
sigma
sigma

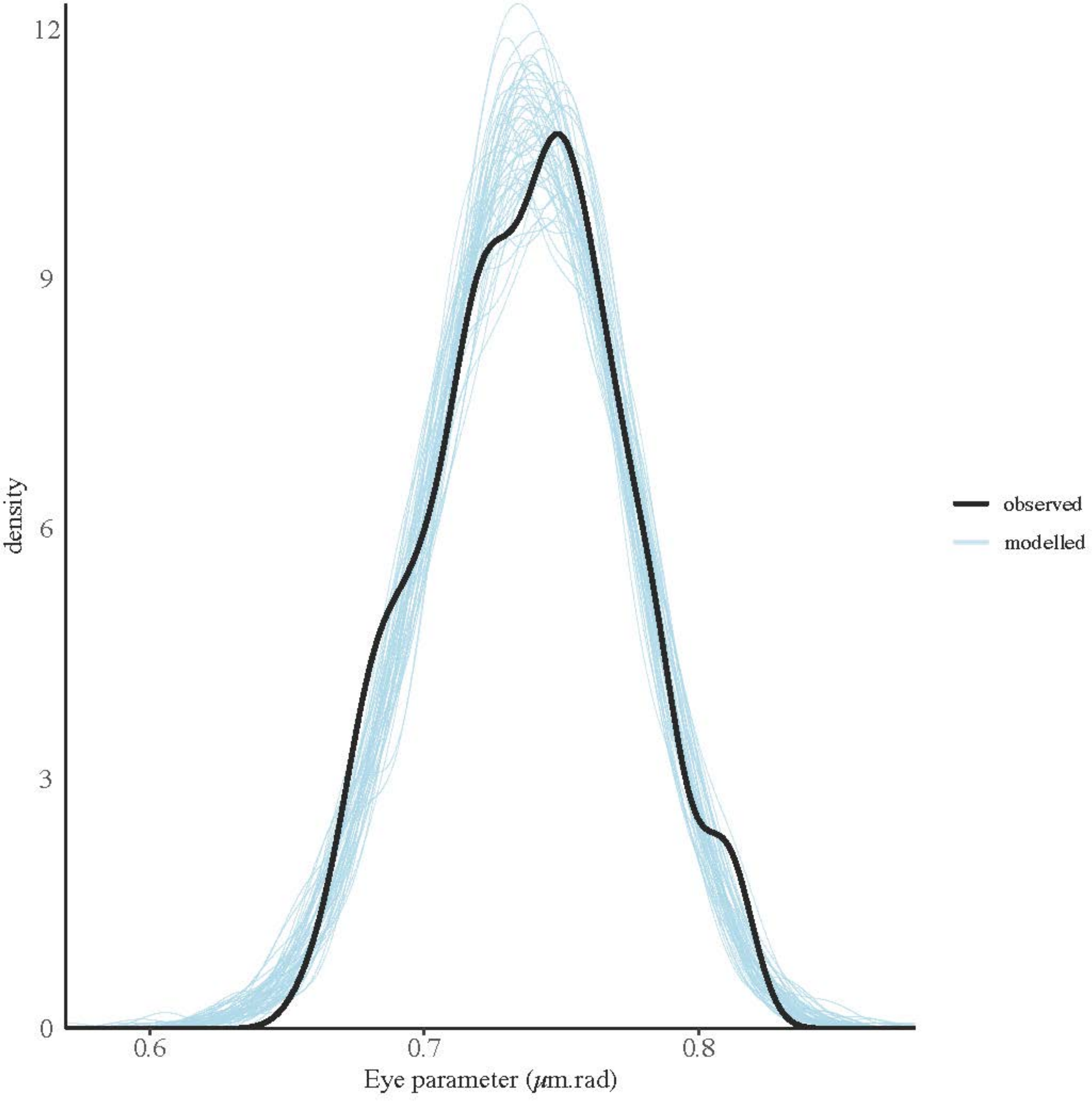
12
9
6
3
0
density
0.6
0.7
0.8
Eye parameter (μm.rad)
observed
modelled

Curvature (μm)

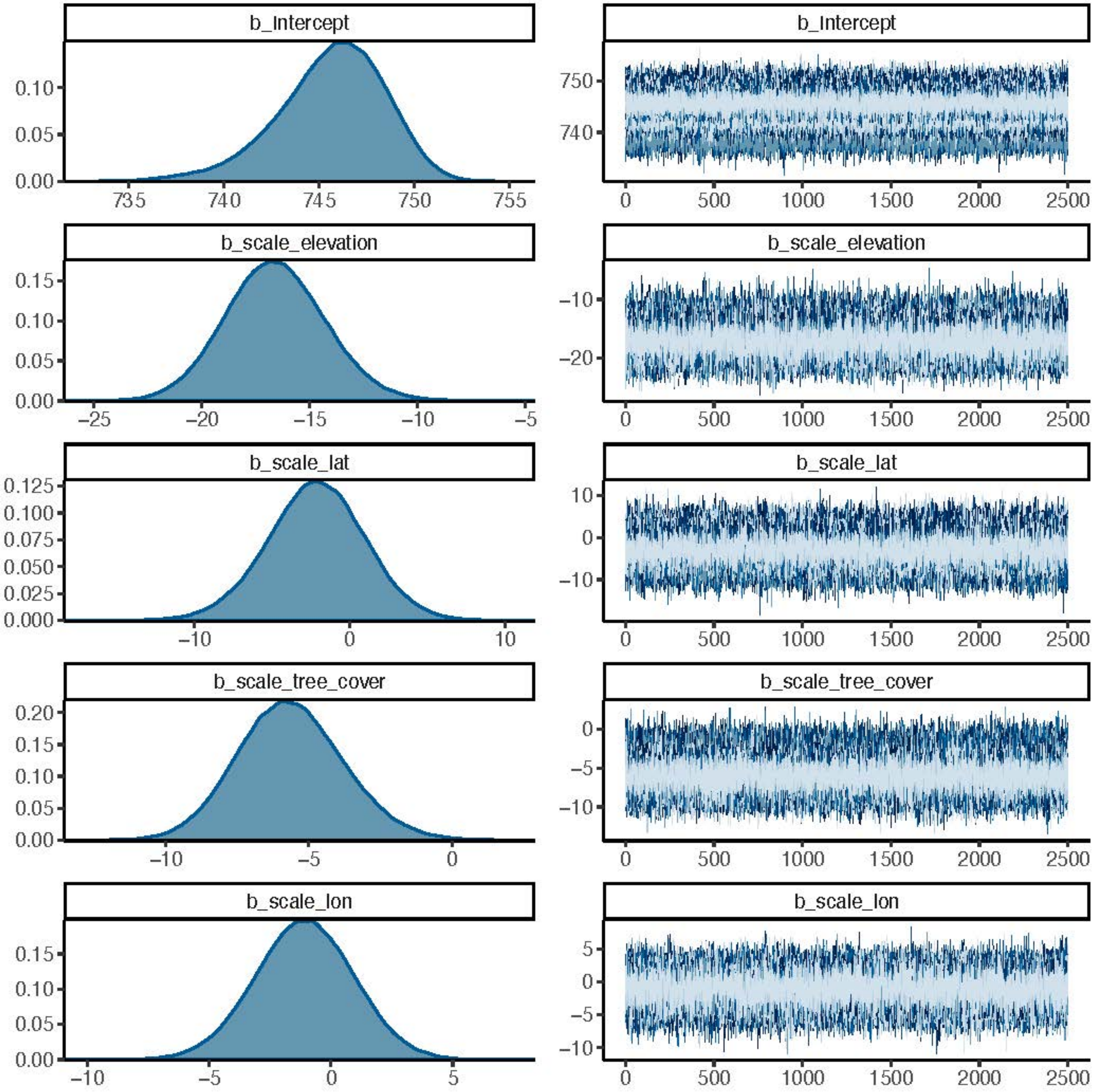

b_Intercept
b_Intercept
b_scale_elevation
b_scale_elevation
b_scale_lat
b_scale_lat
b_scale_tree_cover
b_scale_tree_cover
b_scale_lon
b_scale_lon

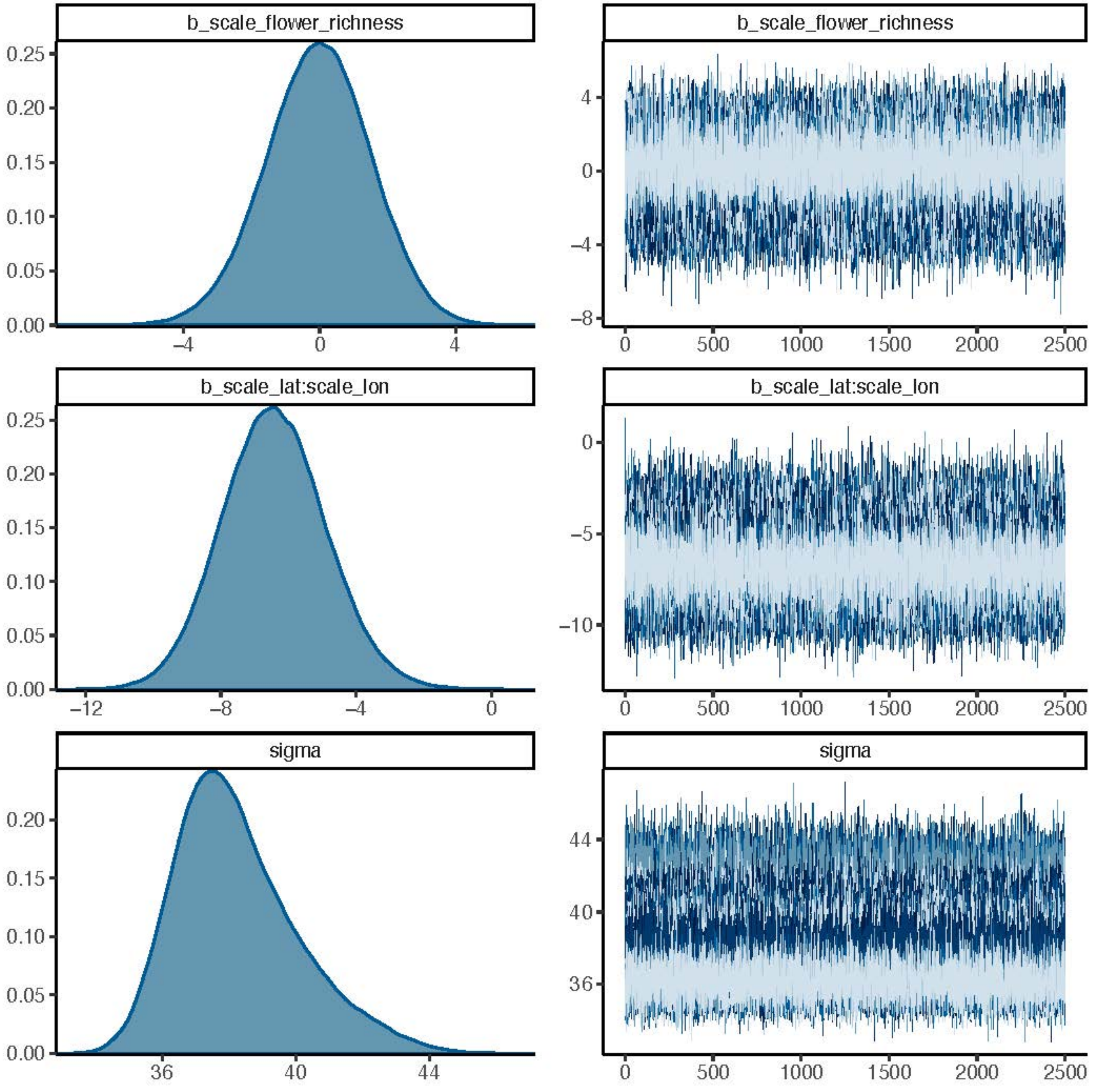
b_scale_flower_richness
b_scale_flower_richness
b_scale_lat:scale_lon
b_scale_lat:scale_lon
sigma
sigma
0.25
0.20
0.15
0.10
0.05
0.00
−4
0
4
−8
−12
36
40
44
−5
−10
500
1000
1500
2000
2500

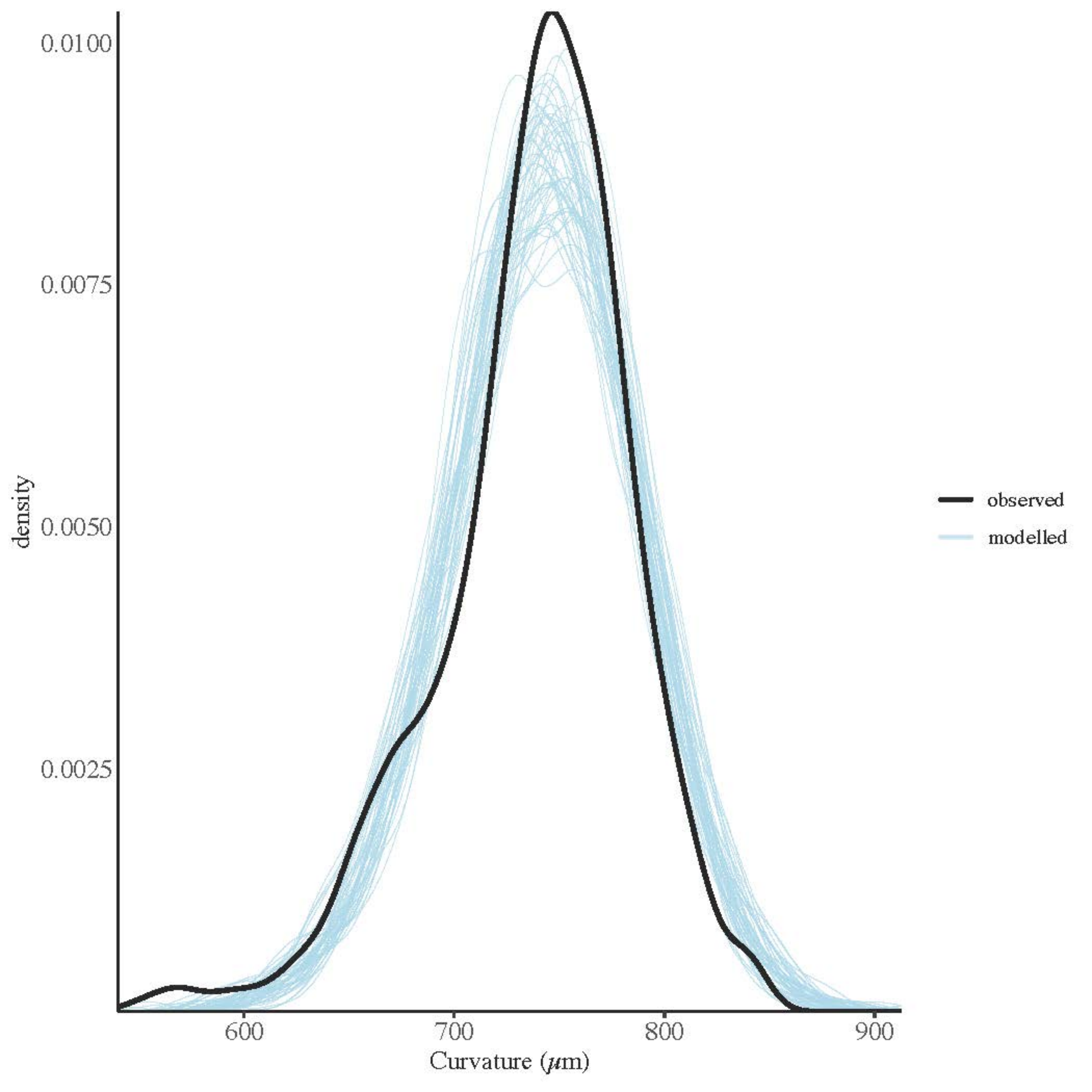

0.0100
0.0075
0.0050
0.0025
density
600
700
800
900
Curvature (μm)
observed
modelled

Central ocellus diameter (μm)

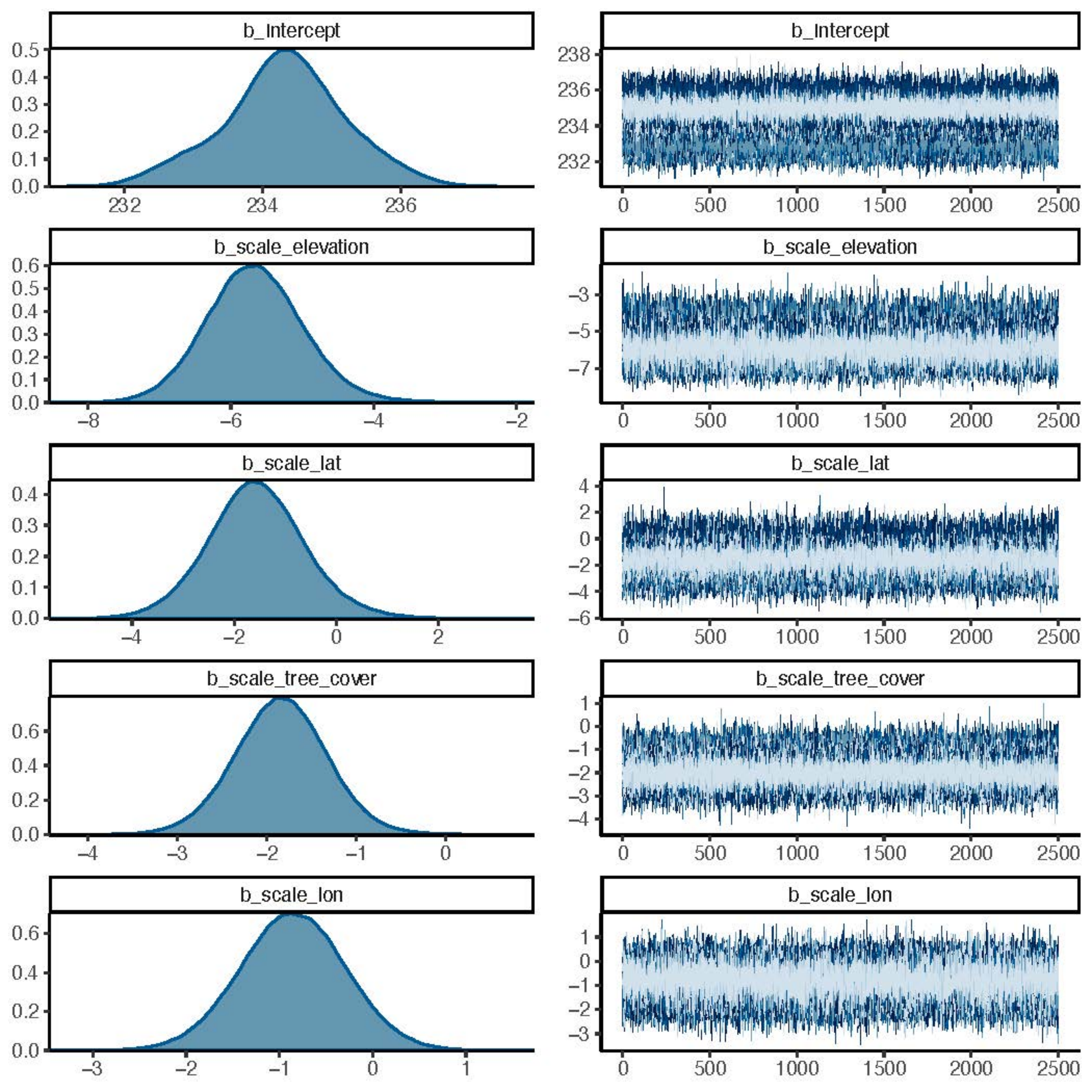
b_Intercept
b_Intercept
b_scale_elevation
b_scale_elevation
b_scale_lat
b_scale_lat
b_scale_tree_cover
b_scale_tree_cover
b_scale_lon
b_scale_lon

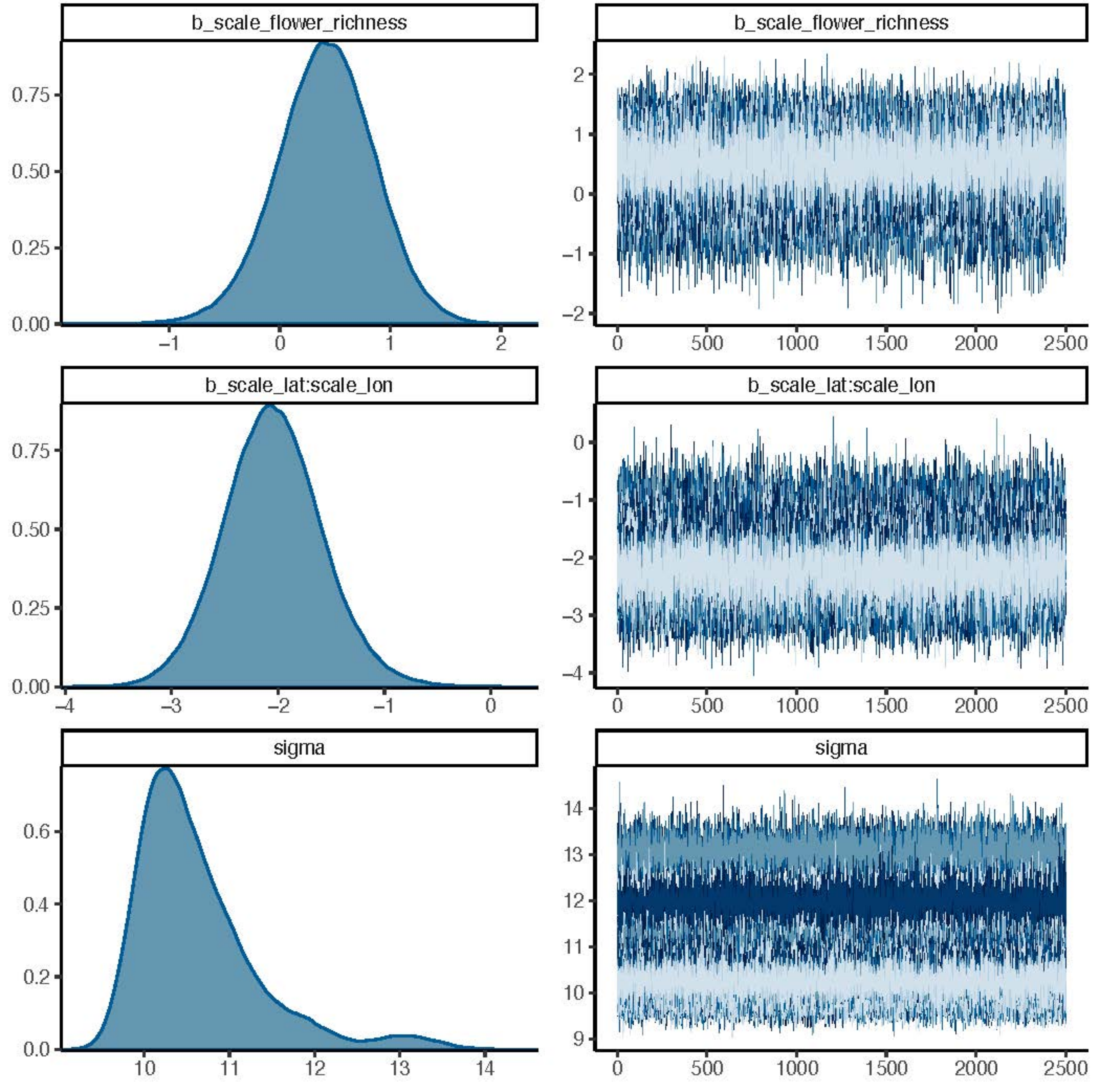

b_scale_flower_richness
b_scale_flower_richness
b_scale_lat:scale_lon
b_scale_lat:scale_lon
sigma
sigma
0.00
0.25
0.50
0.75
−1
0
1
2
−2
−1
0
1
2
0
500
1000
1500
2000
2500
−4
−3
−2
−1
0
0.0
0.2
0.4
0.6
10
11
12
13
14
9
10
11
12
13
14

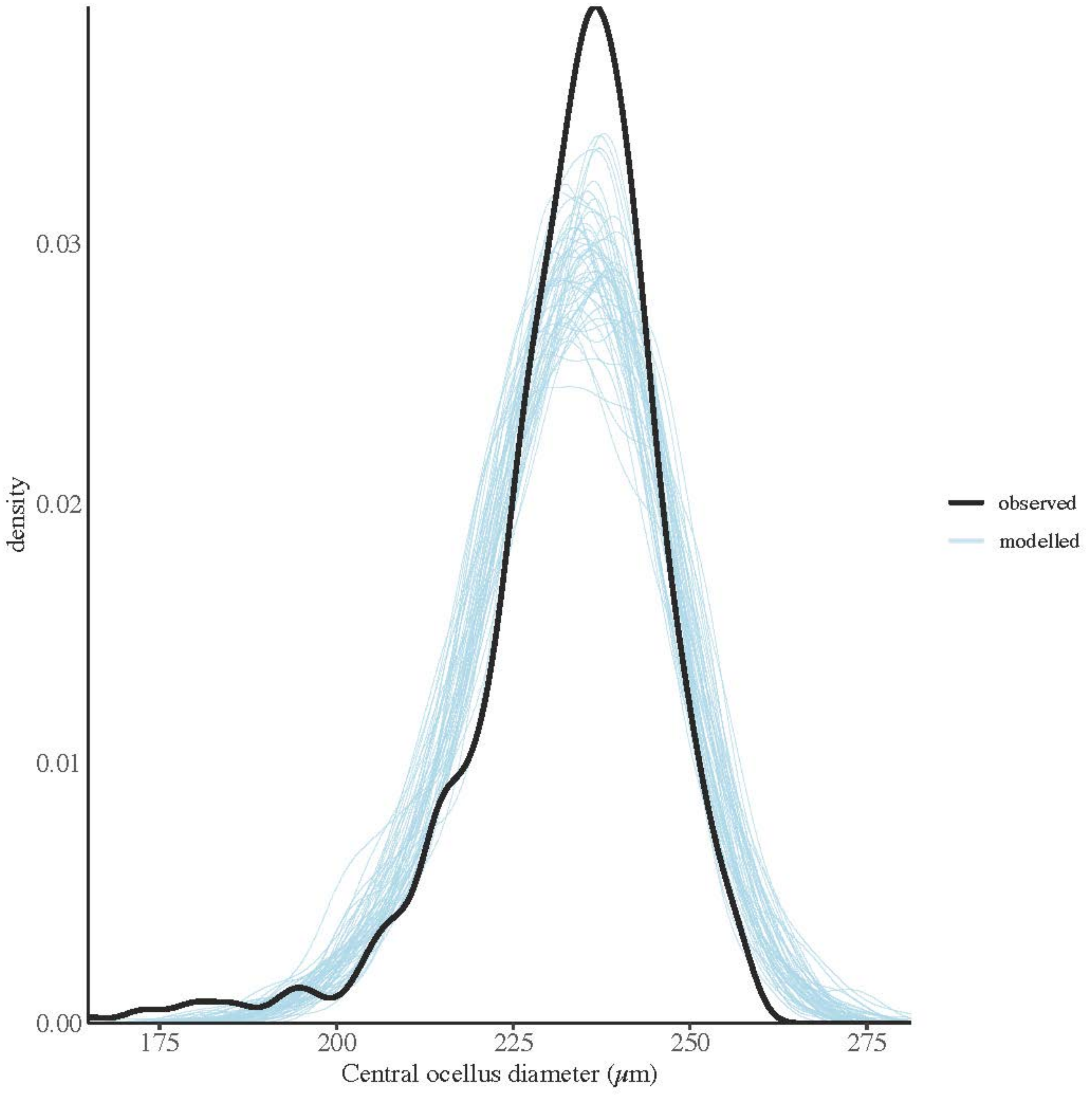

0.03
0.02
0.01
0.00
density
175
200
225
250
275
Central ocellus diameter (μm)
observed
modelled

Lateral ocelli diameter (μm)

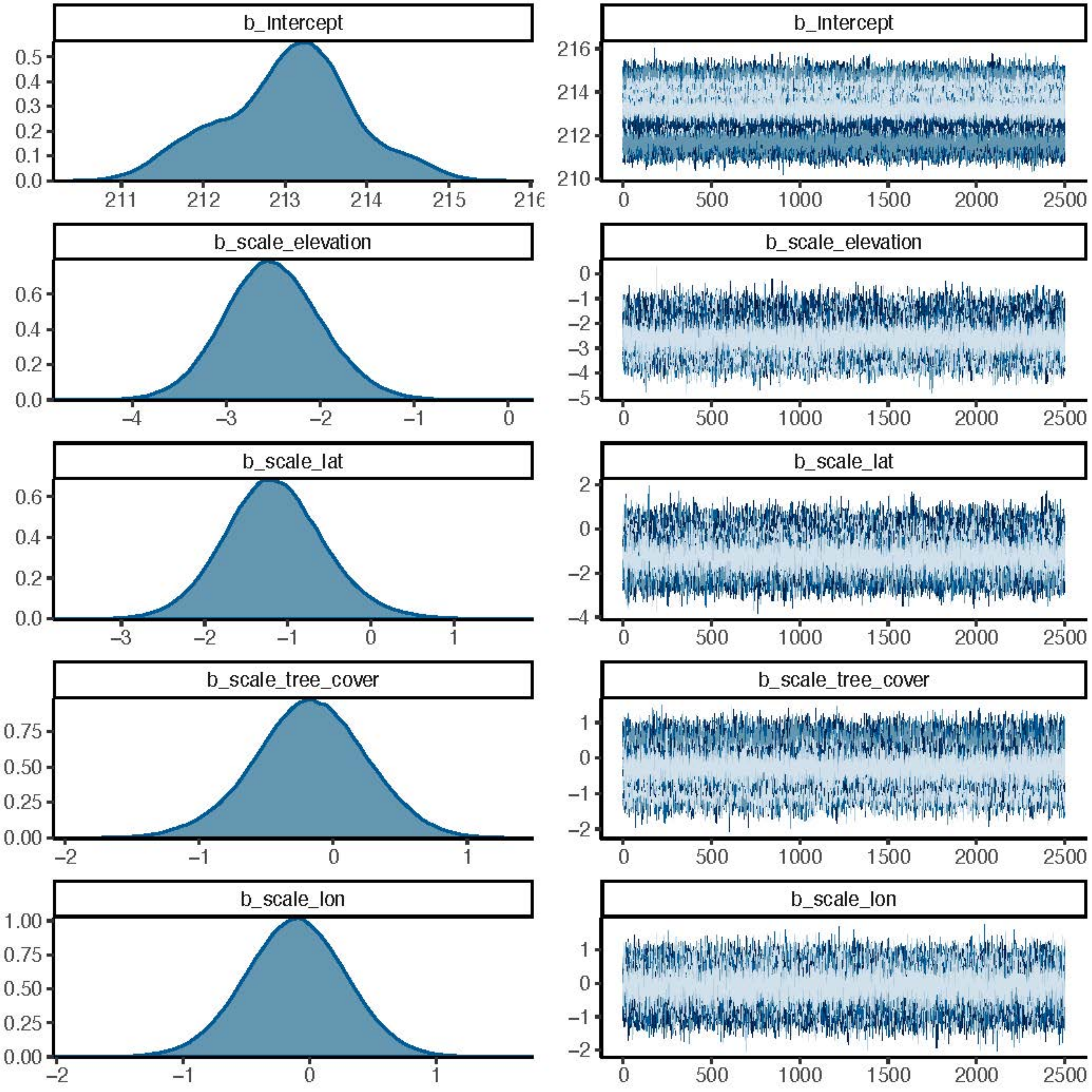
b_Intercept
b_Intercept
b_scale_elevation
b_scale_elevation
b_scale_lat
b_scale_lat
b_scale_tree_cover
b_scale_tree_cover
b_scale_lon
b_scale_lon

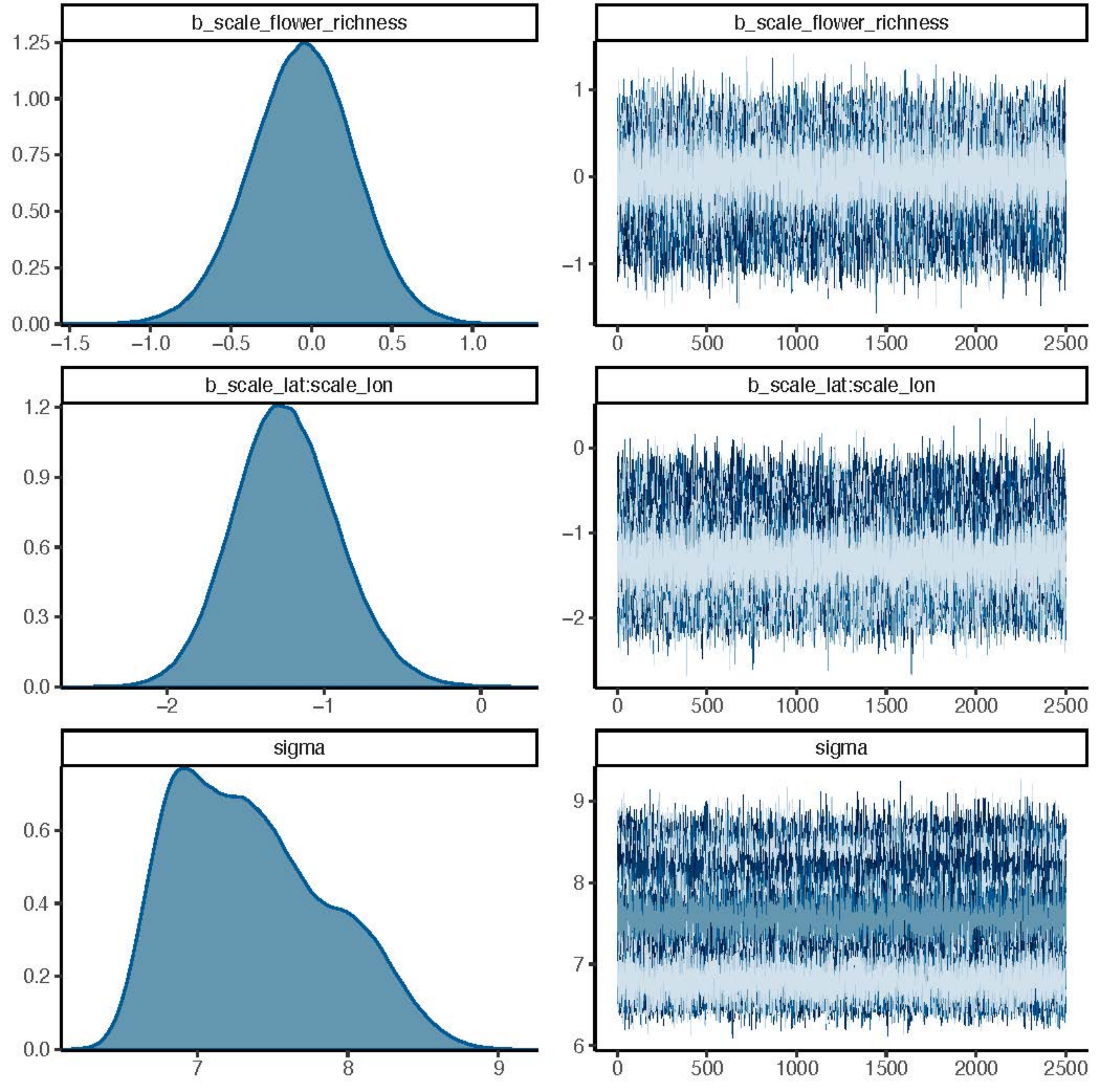

b_scale_flower_richness
1.25
1.00
0.75
0.50
0.25
0.00
−1.5
−1.0
−0.5
0.0
0.5
1.0
b_scale_flower_richness
1
0
−1
0
500
1000
1500
2000
2500
b_scale_lat:scale_lon
1.2
0.9
0.6
0.3
0.0
−2
−1
0
b_scale_lat:scale_lon
0
−1
−2
0
500
1000
1500
2000
2500
sigma
0.6
0.4
0.2
0.0
7
8
9
sigma
9
8
7
6
0
500
1000
1500
2000
2500

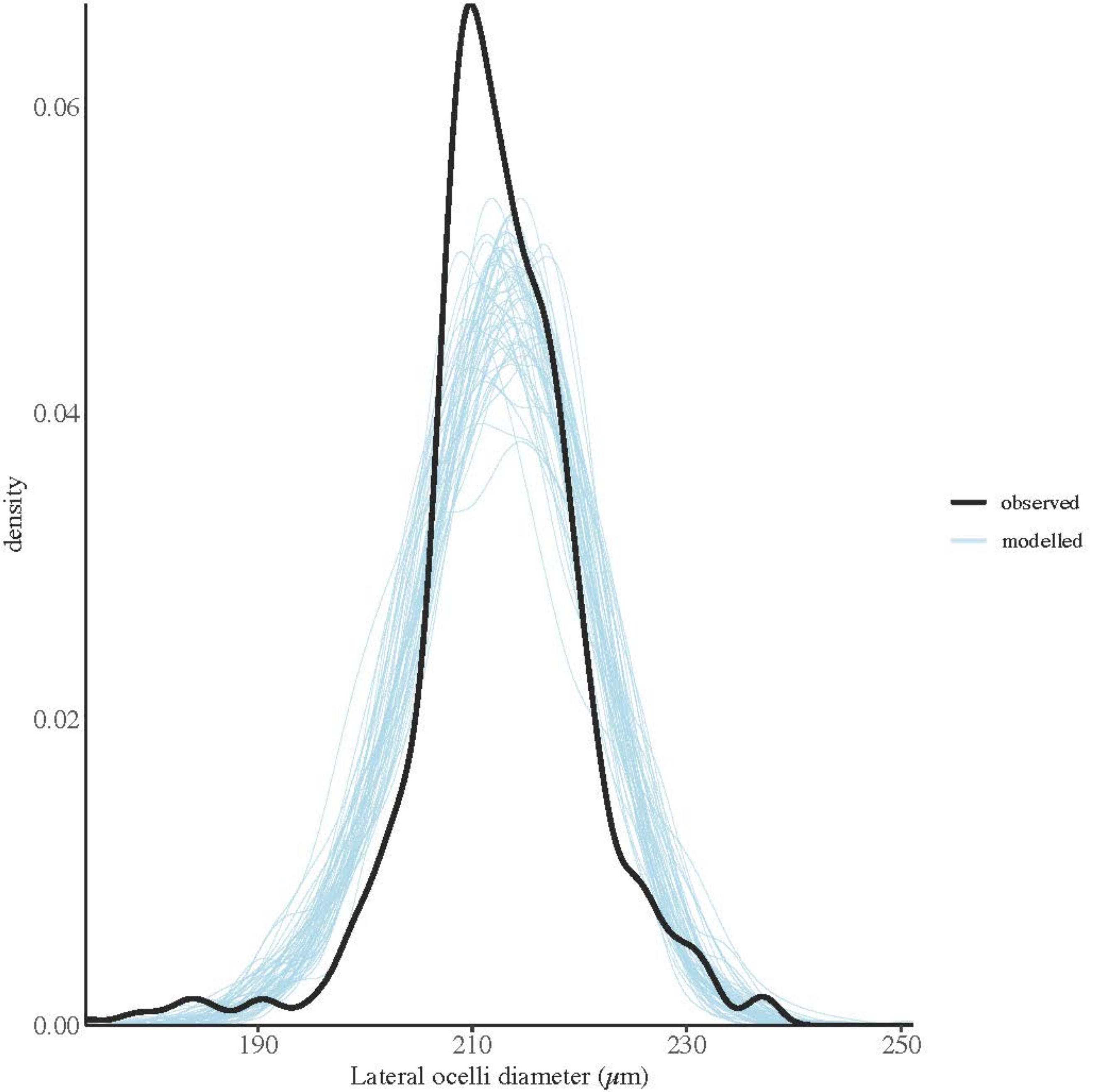

0.06
0.04
0.02
0.00
density
190
210
230
250
Lateral ocelli diameter (μm)
observed
modelled

Ocellar alignment (°)

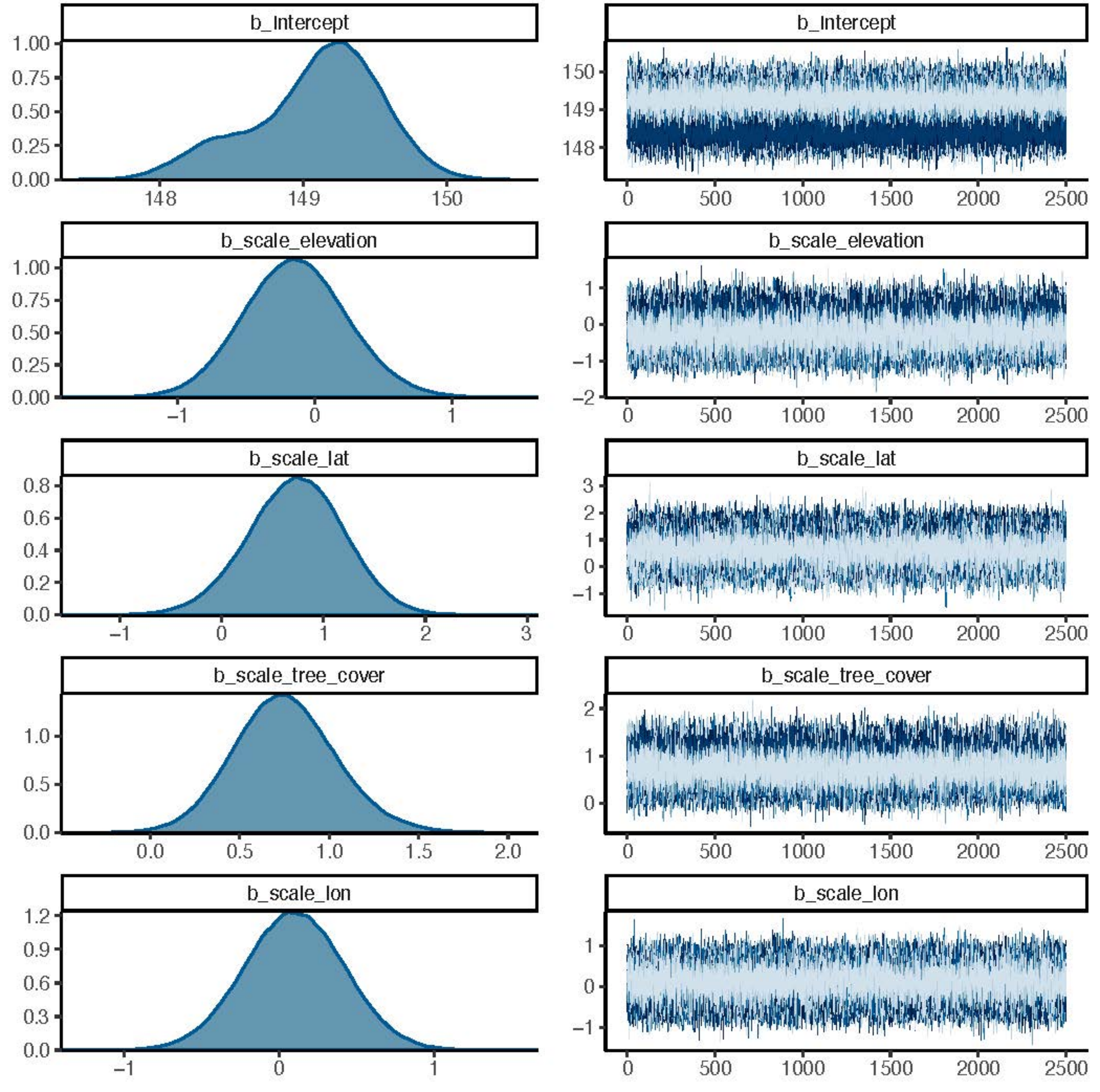

b_Intercept
b_scale_elevation
b_scale_lat
b_scale_tree_cover
b_scale_lon

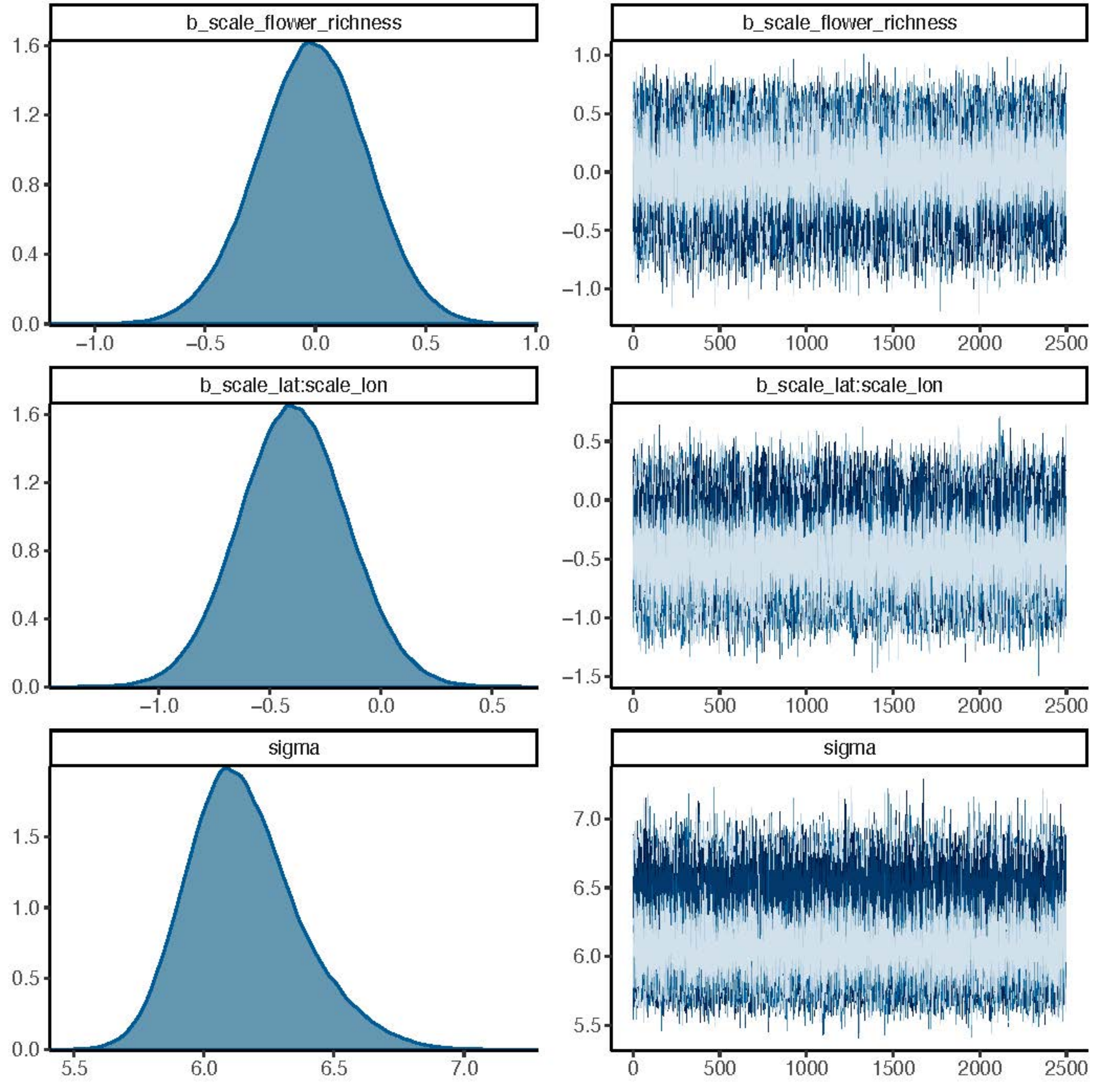
b_scale_flower_richness
b_scale_flower_richness
b_scale_lat:scale_lon
b_scale_lat:scale_lon
sigma
sigma

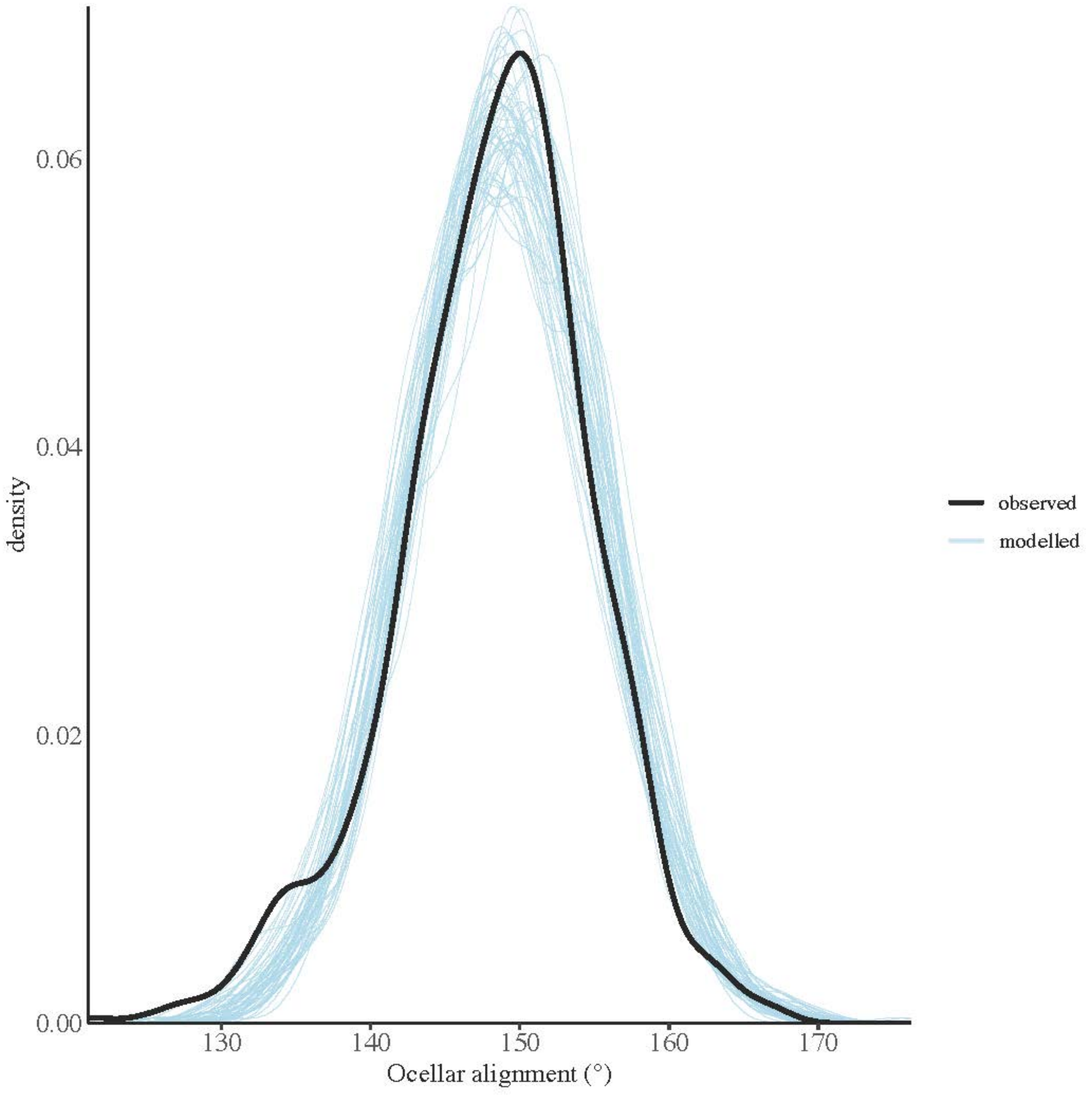
0.06
0.04
0.02
0.00
density
130
140
150
160
170
Ocellar alignment (°)
observed
modelled

Monocular FOV (%)

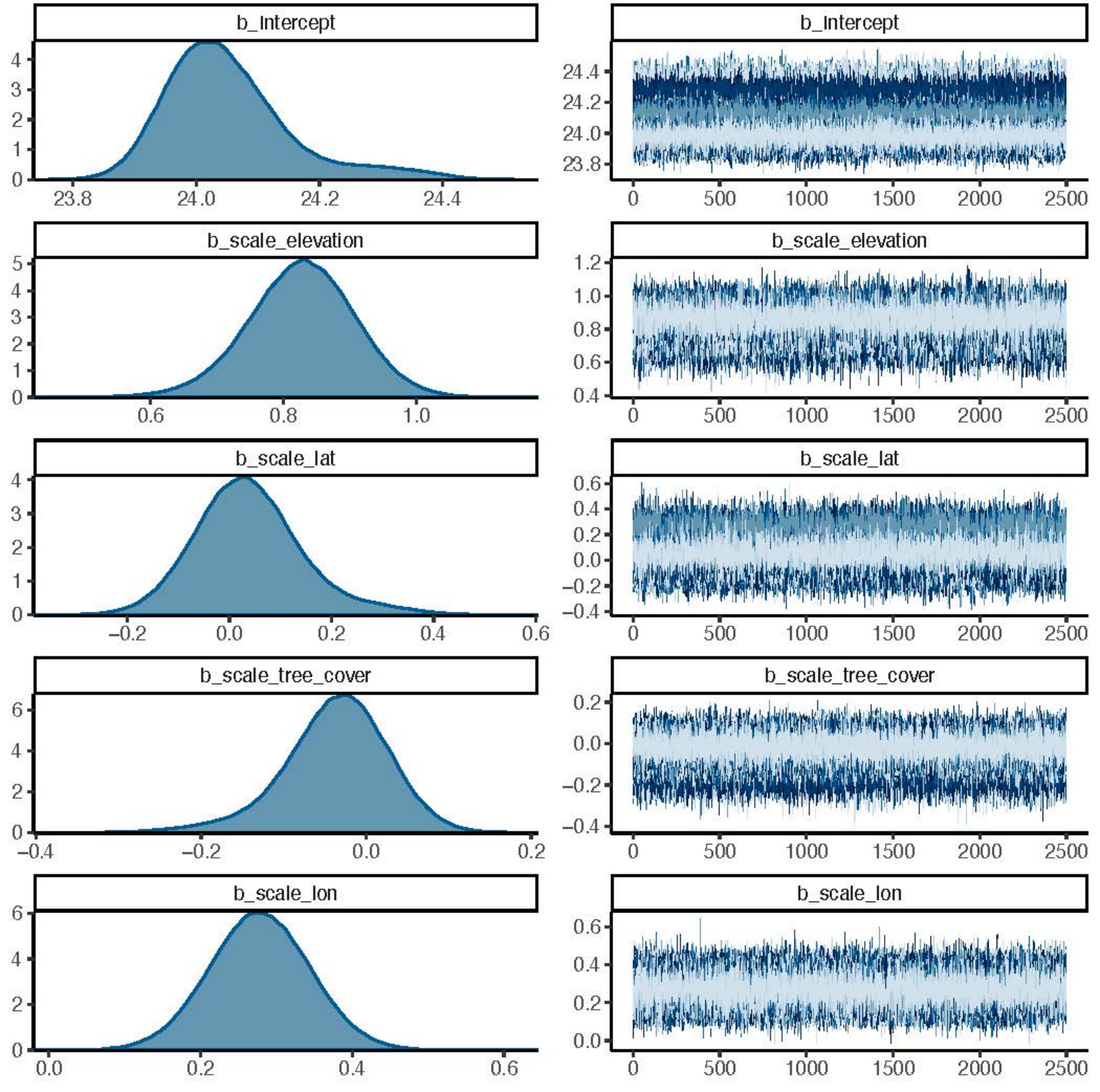
b_Intercept
b_scale_elevation
b_scale_lat
b_scale_tree_cover
b_scale_lon

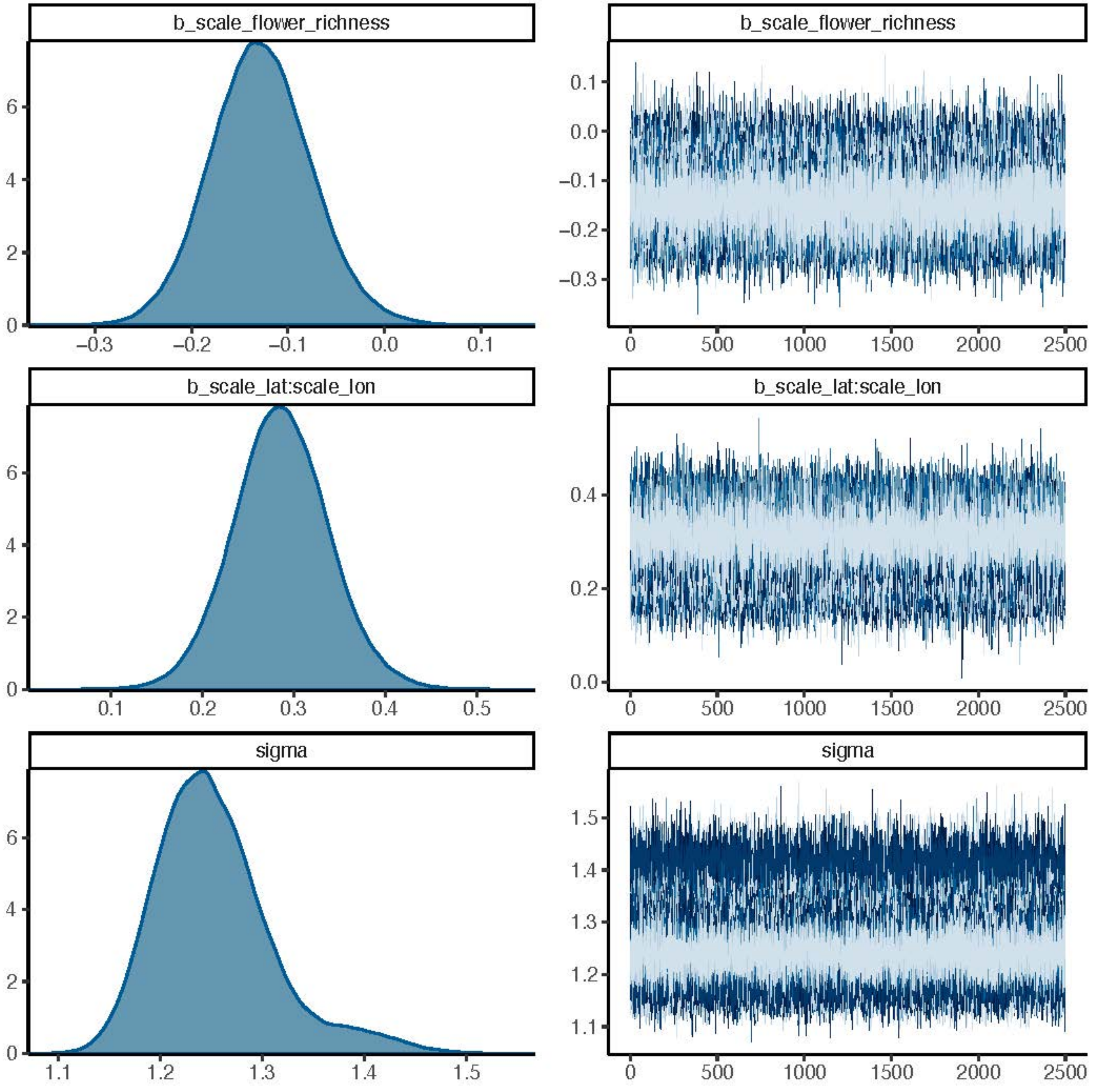
b_scale_flower_richness
b_scale_flower_richness
b_scale_lat:scale_lon
b_scale_lat:scale_lon
sigma
sigma
0
2
4
6
−0.3
−0.2
−0.1
0.0
0.1
0
500
1000
1500
2000
2500
0.1
0.2
0.3
0.4
0.5
1.1
1.2
1.3
1.4
1.5

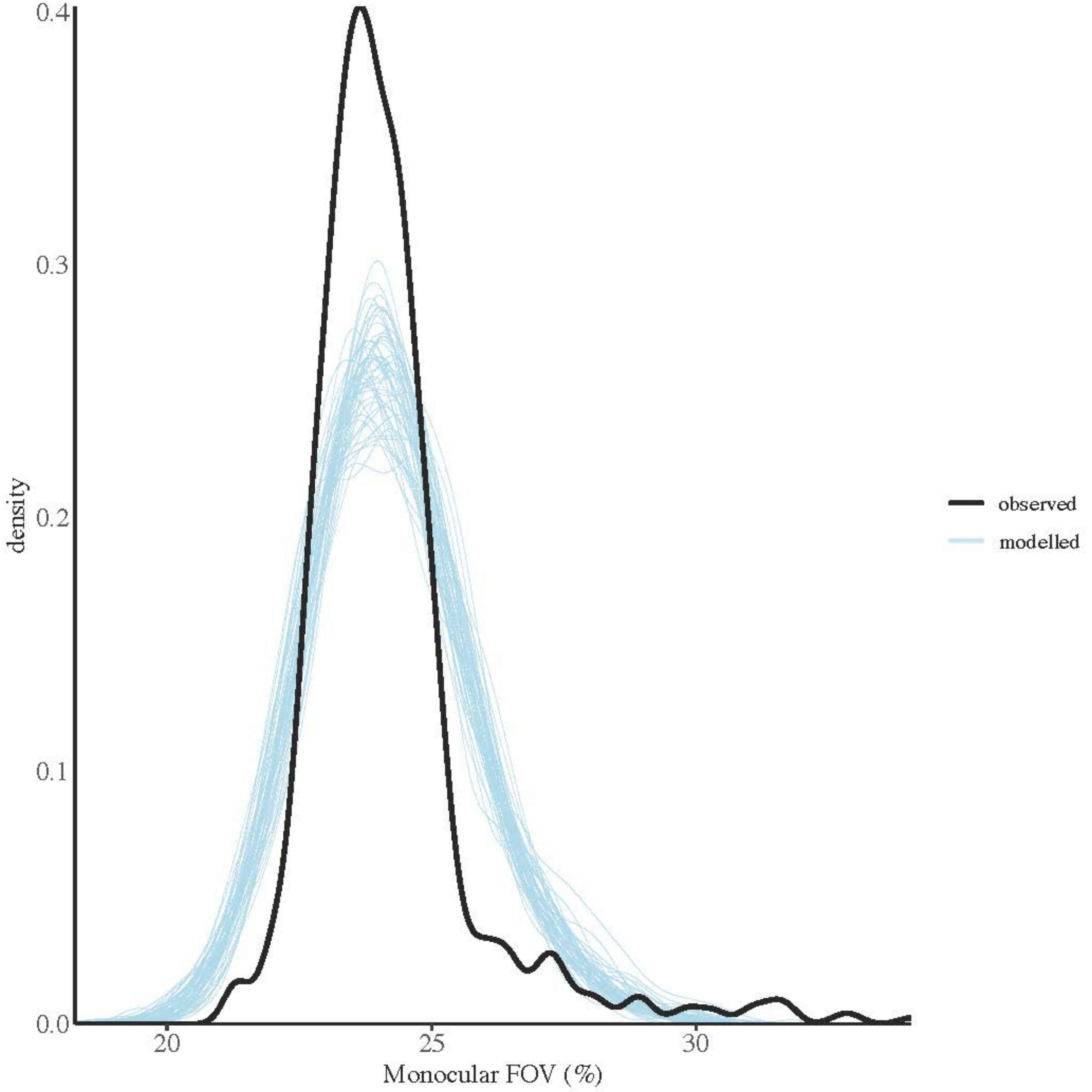
0.4
0.3
0.2
0.1
0.0
density
20
25
30
Monocular FOV (%)
observed
modelled

Binocular overlap (%)

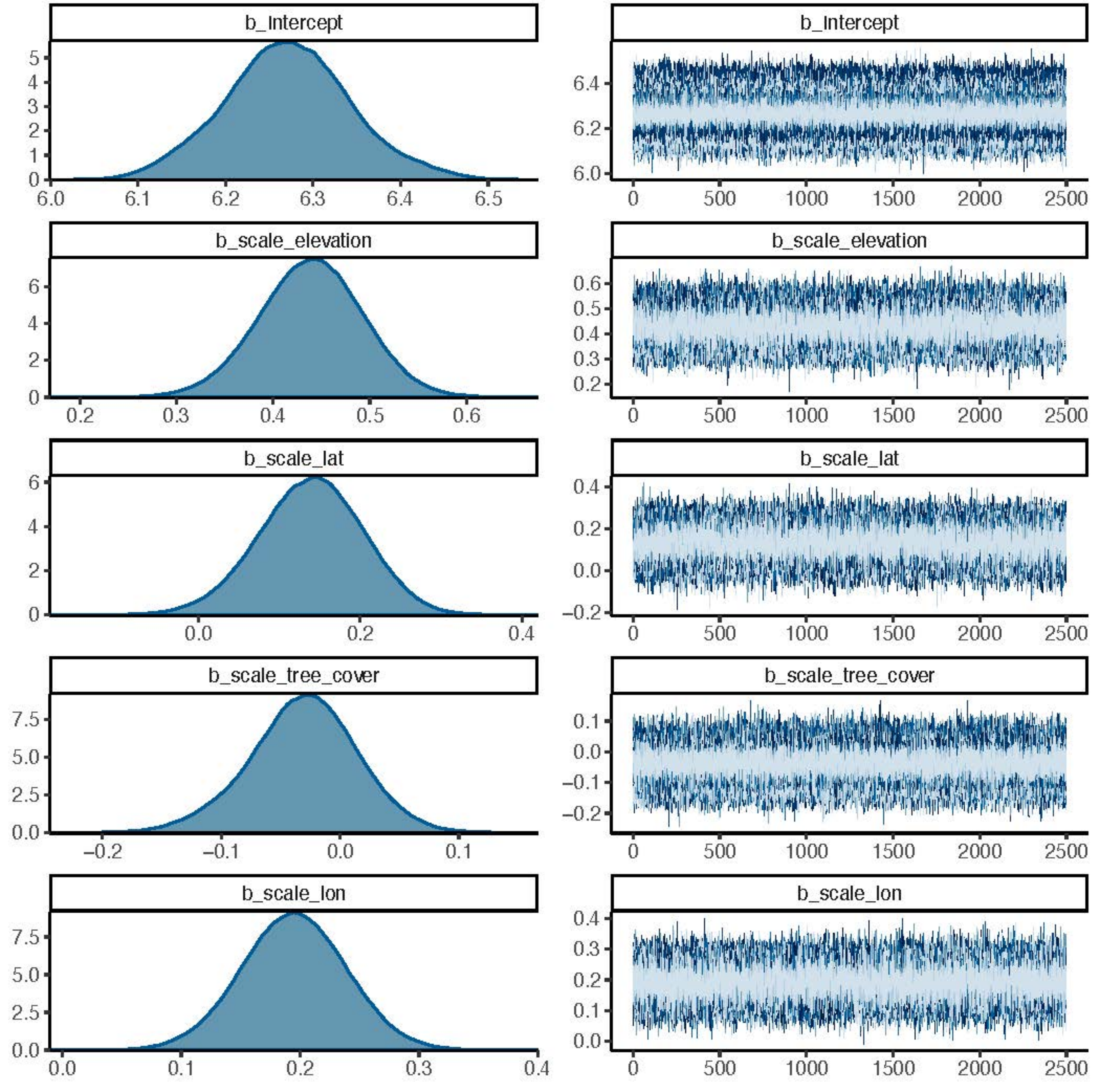

b_Intercept
b_scale_elevation
b_scale_lat
b_scale_tree_cover
b_scale_lon

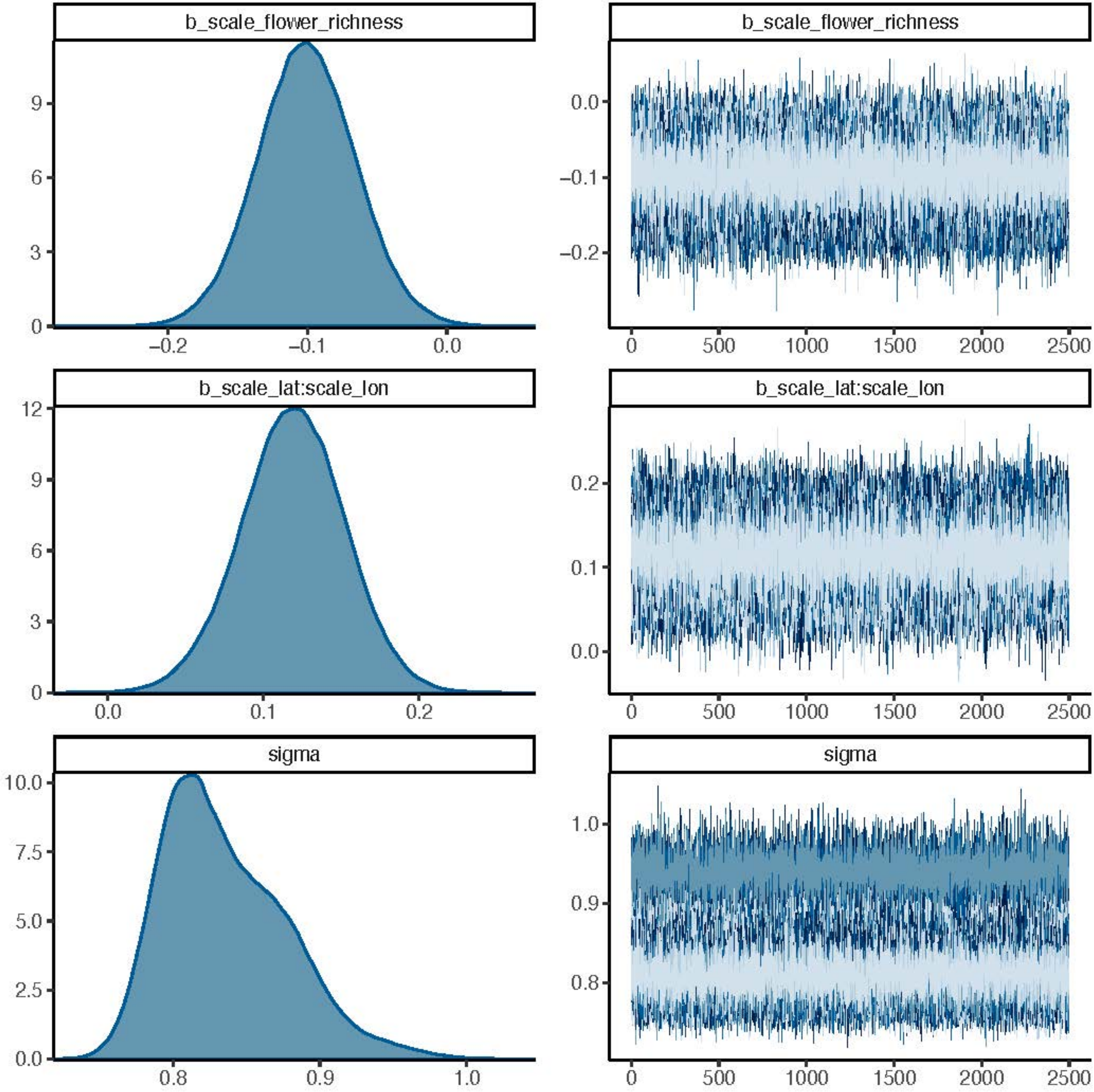

b_scale_flower_richness
9
6
3
0
−0.2
−0.1
0.0
b_scale_flower_richness
0.0
−0.1
−0.2
0
500
1000
1500
2000
2500
b_scale_lat:scale_lon
12
9
6
3
0
0.0
0.1
0.2
b_scale_lat:scale_lon
0.2
0.1
0.0
0
500
1000
1500
2000
2500
sigma
10.0
7.5
5.0
2.5
0.0
0.8
0.9
1.0
sigma
1.0
0.9
0.8
0
500
1000
1500
2000
2500

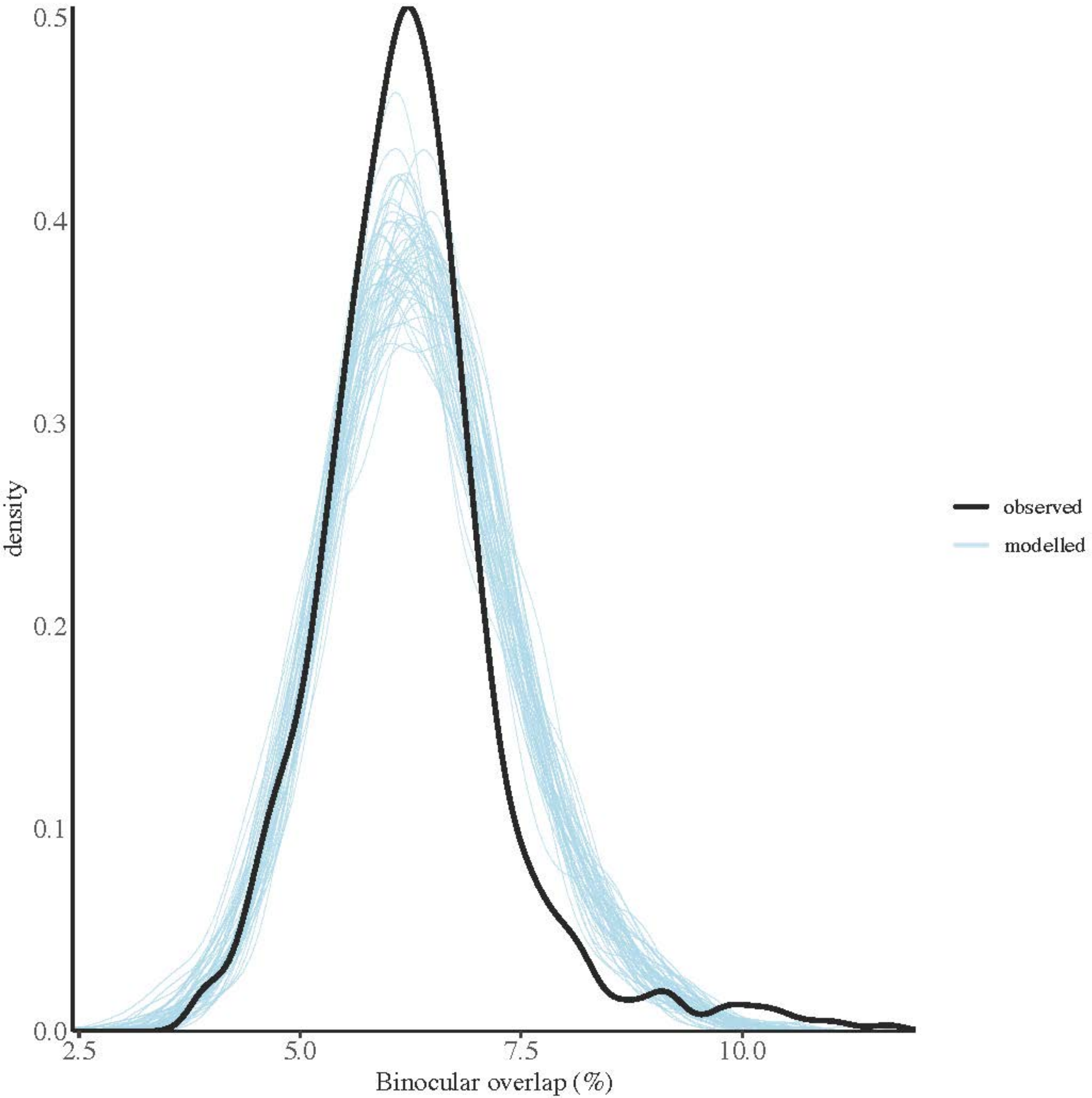
density
0.0
0.1
0.2
0.3
0.4
0.5
2.5
5.0
7.5
10.0
Binocular overlap (%)
observed
modelled

ITD (mm)

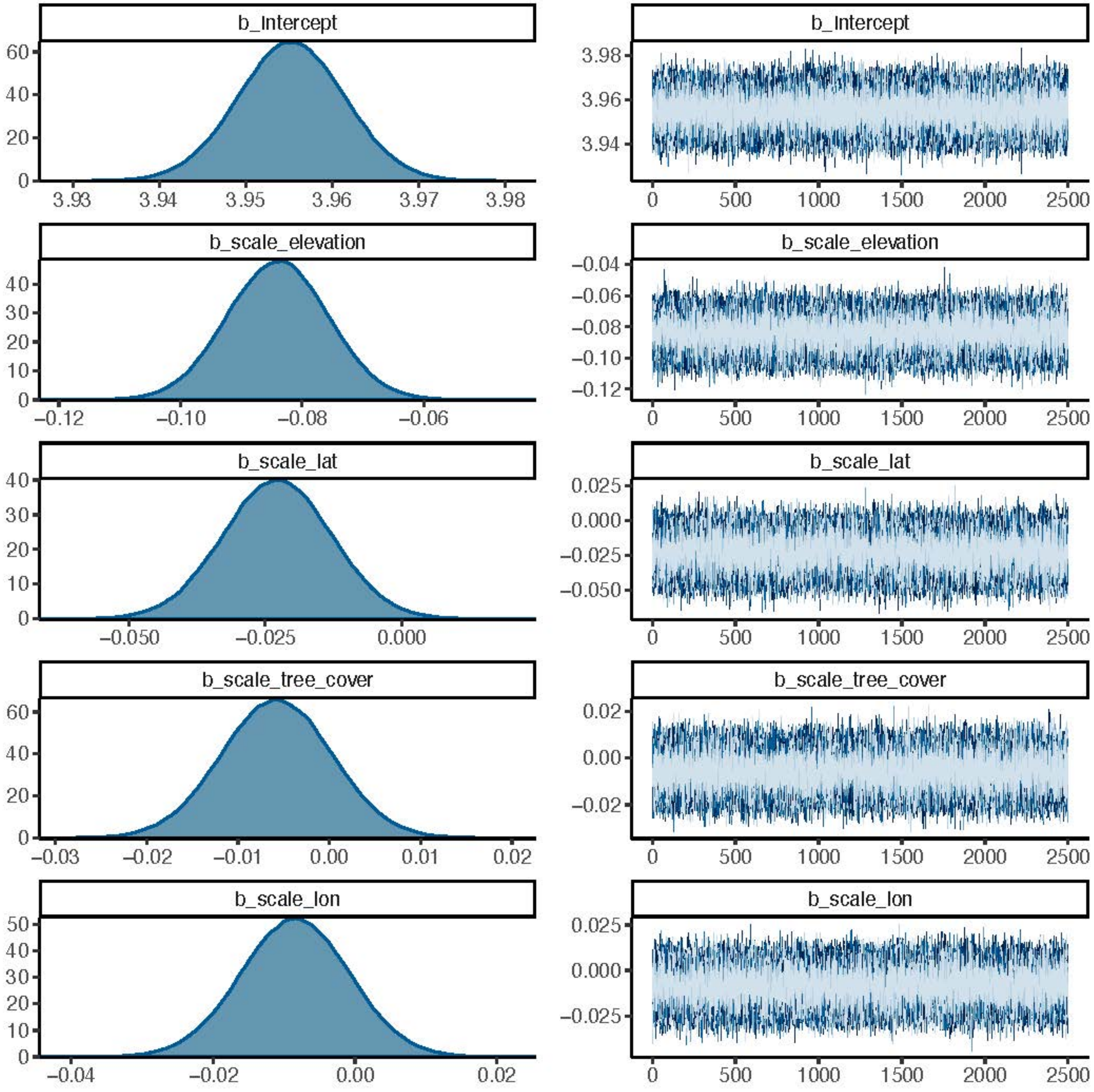

b_Intercept
60
40
20
0
3.93
3.94
3.95
3.96
3.97
3.98
b_Intercept
3.98
3.96
3.94
0
500
1000
1500
2000
2500
b_scale_elevation
40
30
20
10
0
-0.12
-0.10
-0.08
-0.06
b_scale_elevation
-0.04
-0.06
-0.08
-0.10
-0.12
b_scale_lat
40
30
20
10
0
-0.050
-0.025
0.000
b_scale_lat
0.025
0.000
-0.025
-0.050
b_scale_tree_cover
60
40
20
0
-0.03
-0.02
-0.01
0.00
0.01
0.02
b_scale_tree_cover
0.02
0.00
-0.02
b_scale_lon
50
40
30
20
10
0
-0.04
-0.02
0.00
0.02
b_scale_lon
0.025
0.000
-0.025

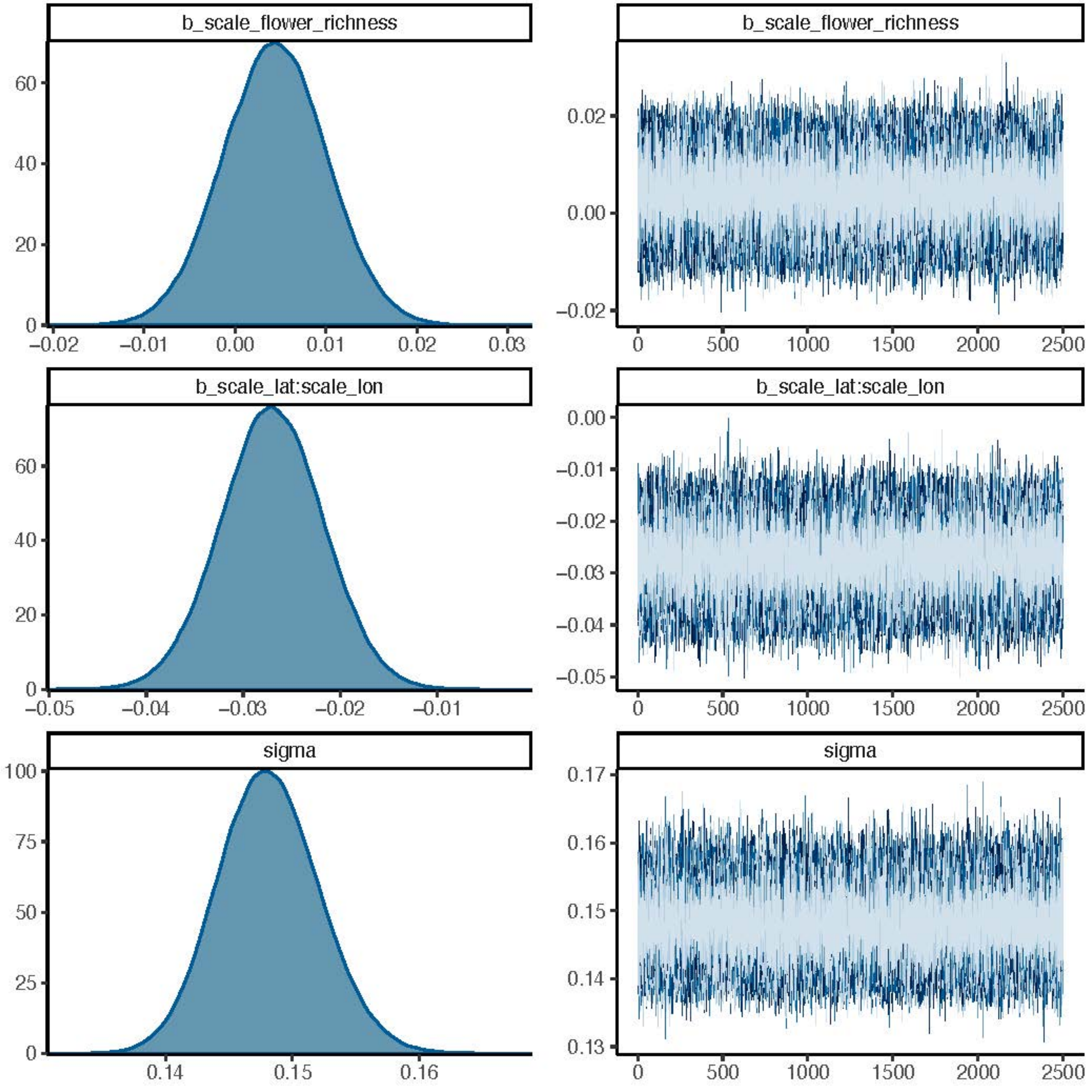

b_scale_flower_richness
b_scale_flower_richness
b_scale_lat:scale_lon
b_scale_lat:scale_lon
sigma
sigma

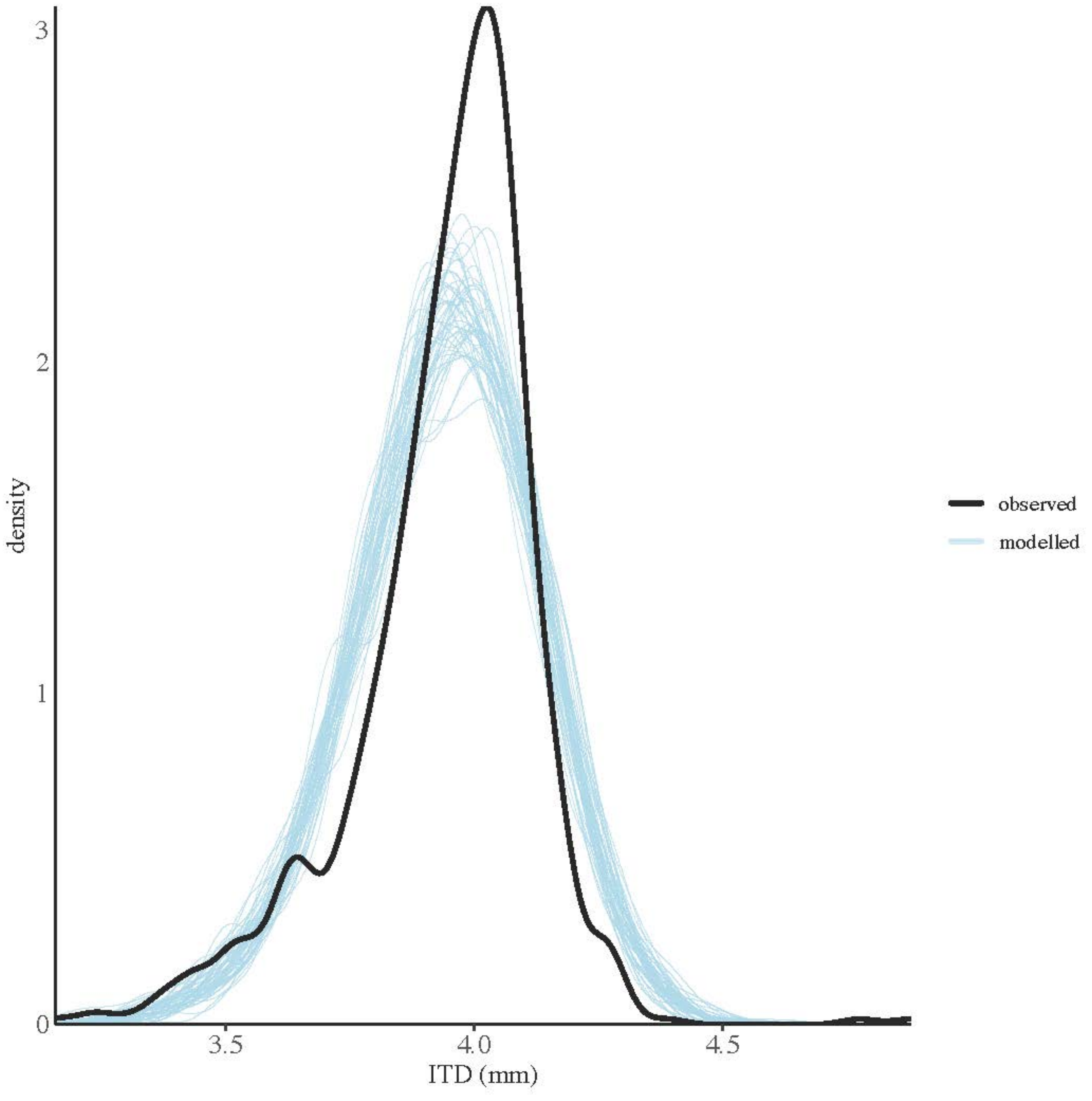

3
2
1
0
density
3.5
4.0
4.5
ITD (mm)
observed
modelled

Effects of tree cover on the observed number of bumblebee species in communities within grasslands across Sweden. Model traces and posterior predictive check for each visual trait modelled with Bayesian inference.

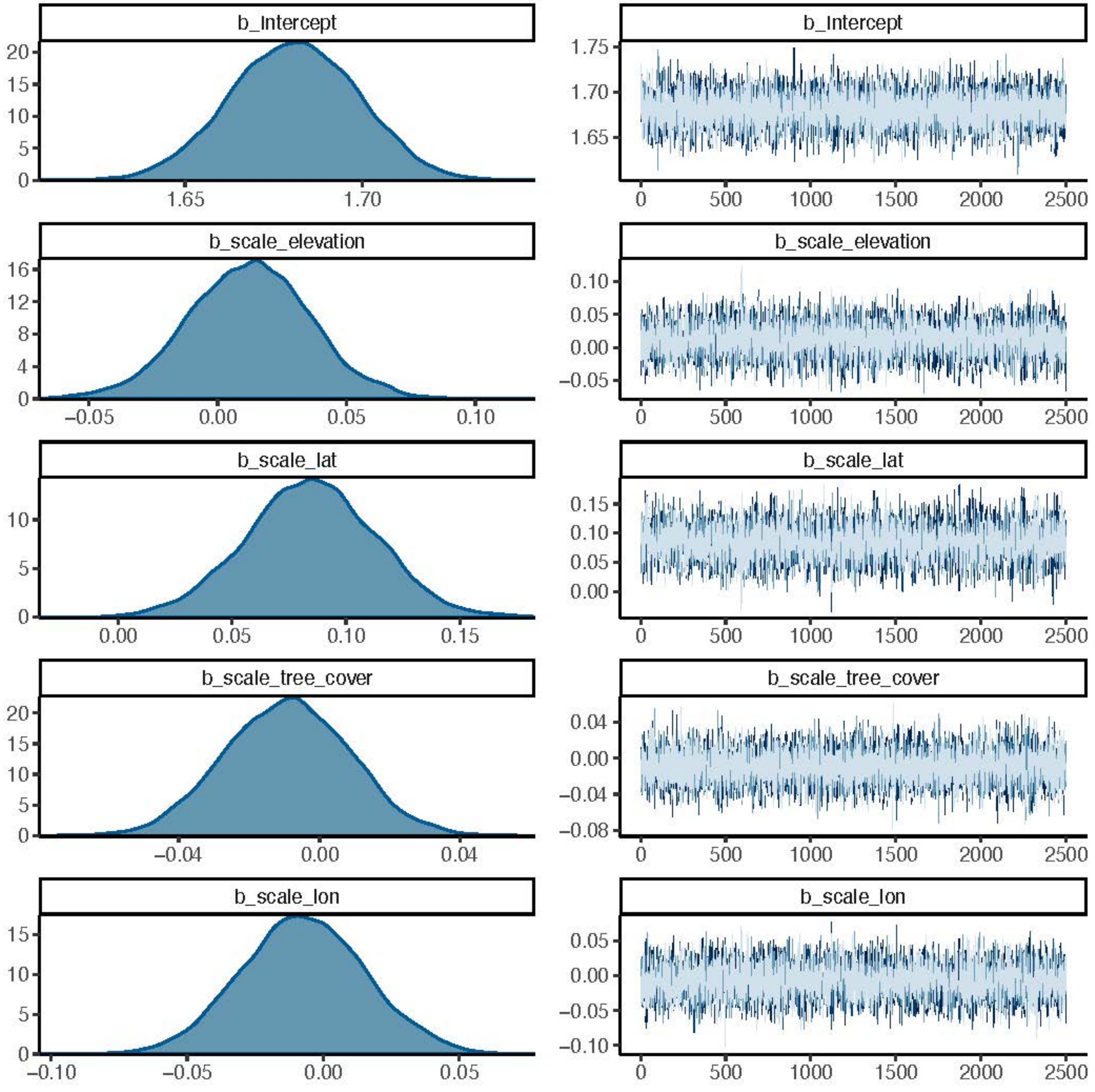
b_Intercept
b_Intercept
b_scale_elevation
b_scale_elevation
b_scale_lat
b_scale_lat
b_scale_tree_cover
b_scale_tree_cover
b_scale_lon
b_scale_lon
0
500
1000
1500
2000
2500

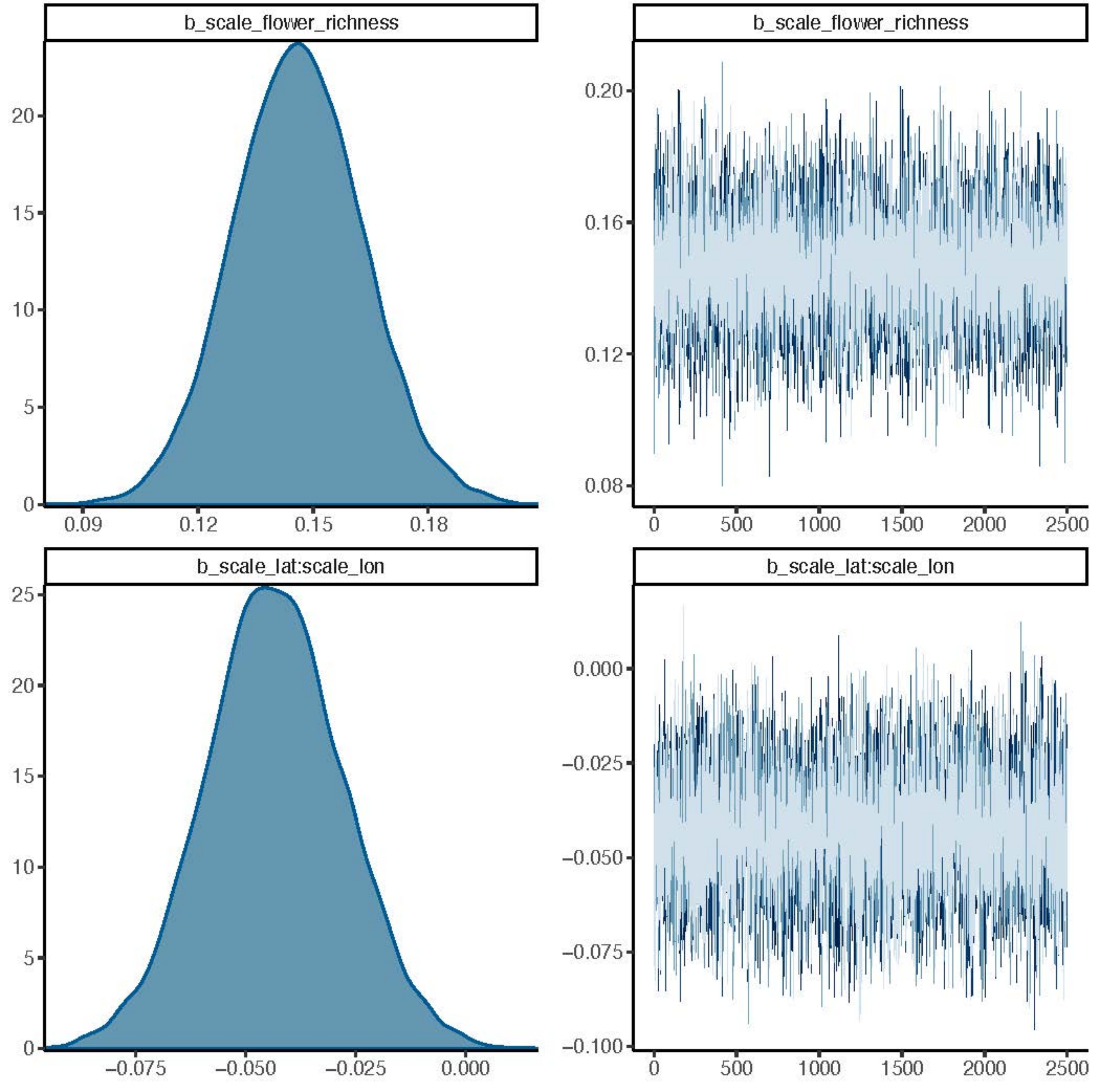

b_scale_flower_richness
0
5
10
15
20
0.09
0.12
0.15
0.18
b_scale_flower_richness
0.08
0.12
0.16
0.20
0
500
1000
1500
2000
2500
b_scale_lat:scale_lon
0
5
10
15
20
25
−0.075
−0.050
−0.025
0.000
b_scale_lat:scale_lon
−0.100
−0.075
−0.050
−0.025
0.000
0
500
1000
1500
2000
2500

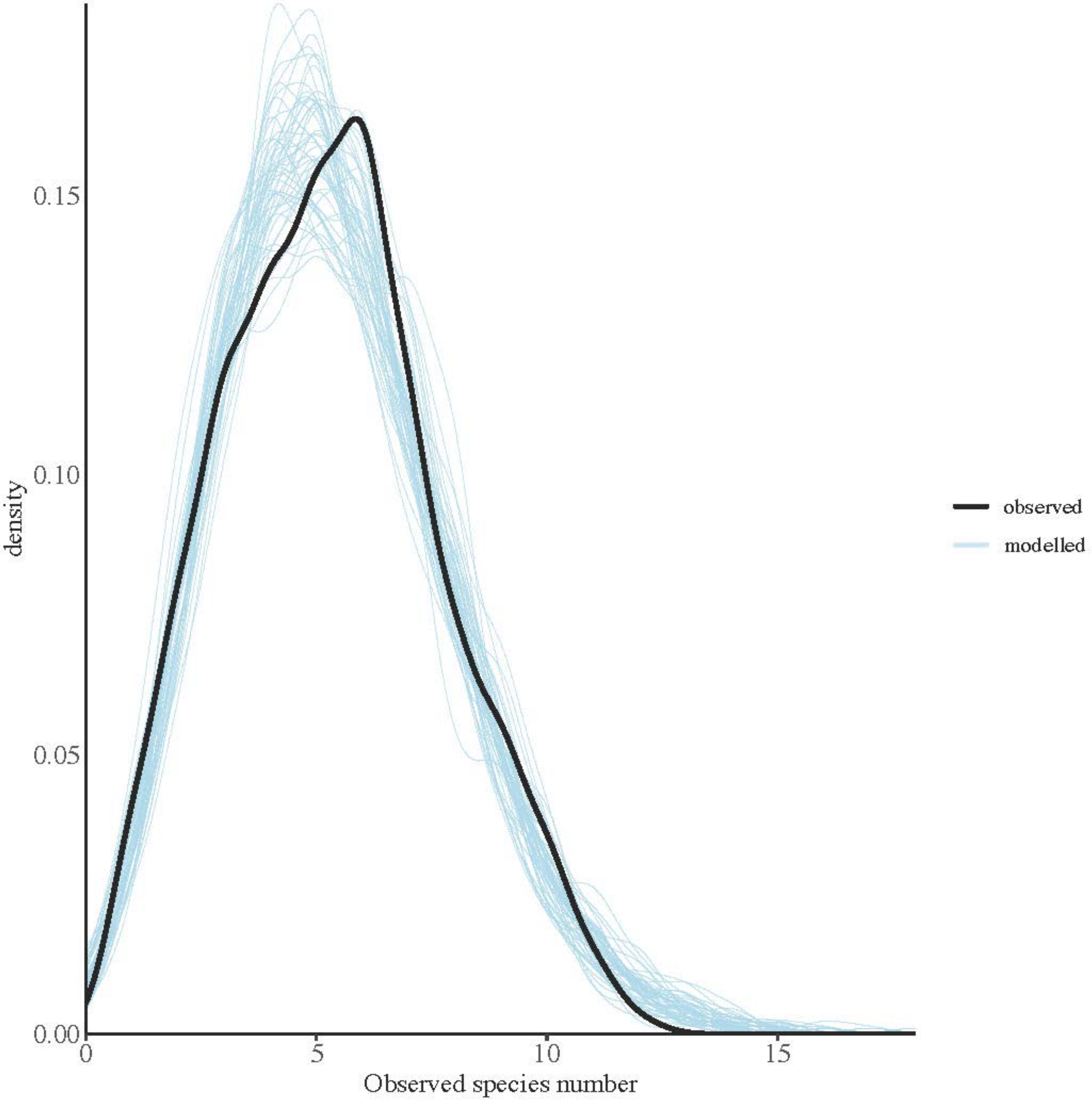

0.15
0.10
0.05
0.00
density
0
5
10
15
Observed species number
observed
modelled

Effects of tree cover on the abundance of the twelve most common species in communities within grasslands across Sweden. Model traces and posterior predictive check for each visual trait modelled with Bayesian inference.

ruderariu

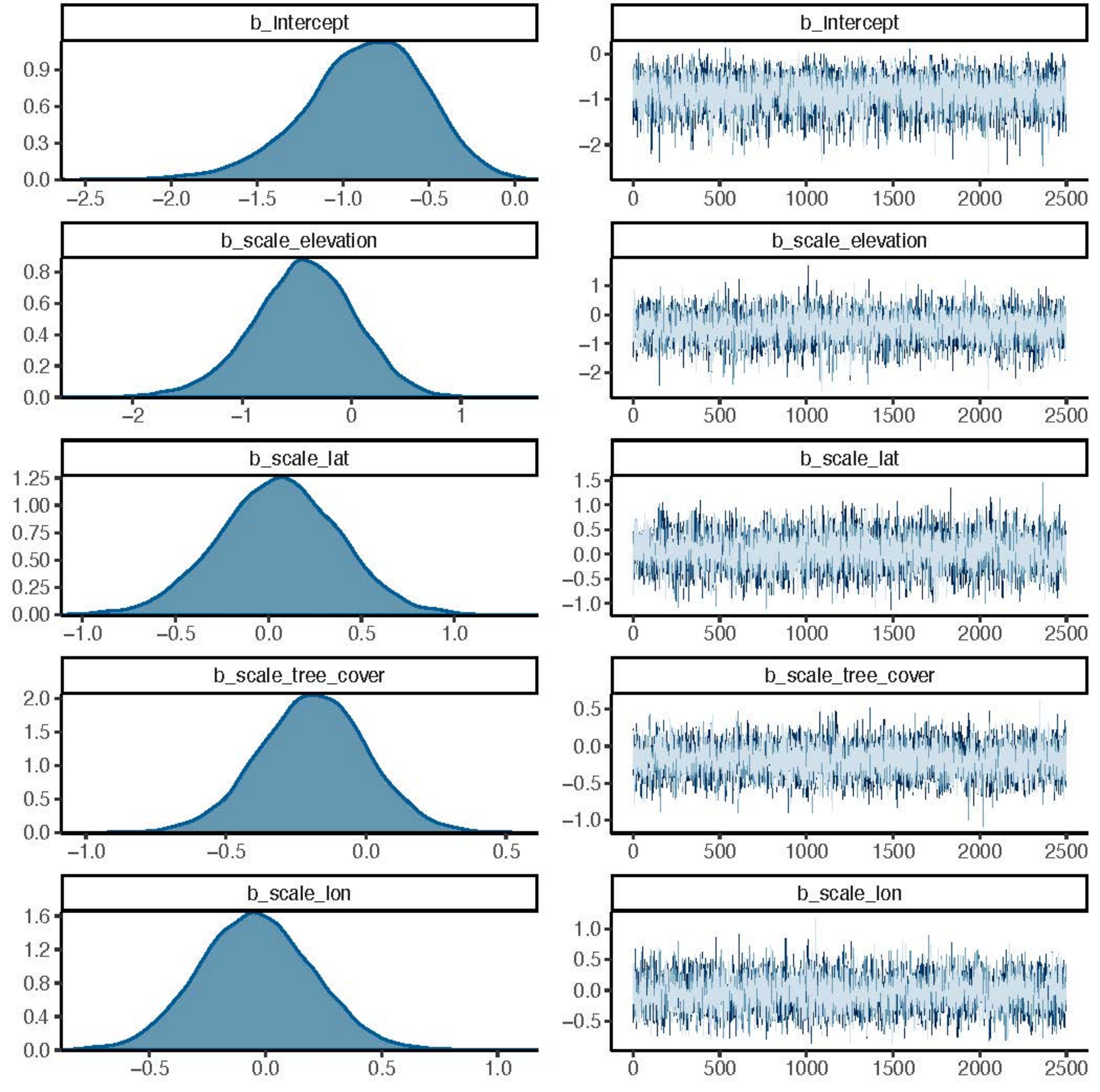
b_Intercept
b_scale_elevation
b_scale_lat
b_scale_tree_cover
b_scale_lon

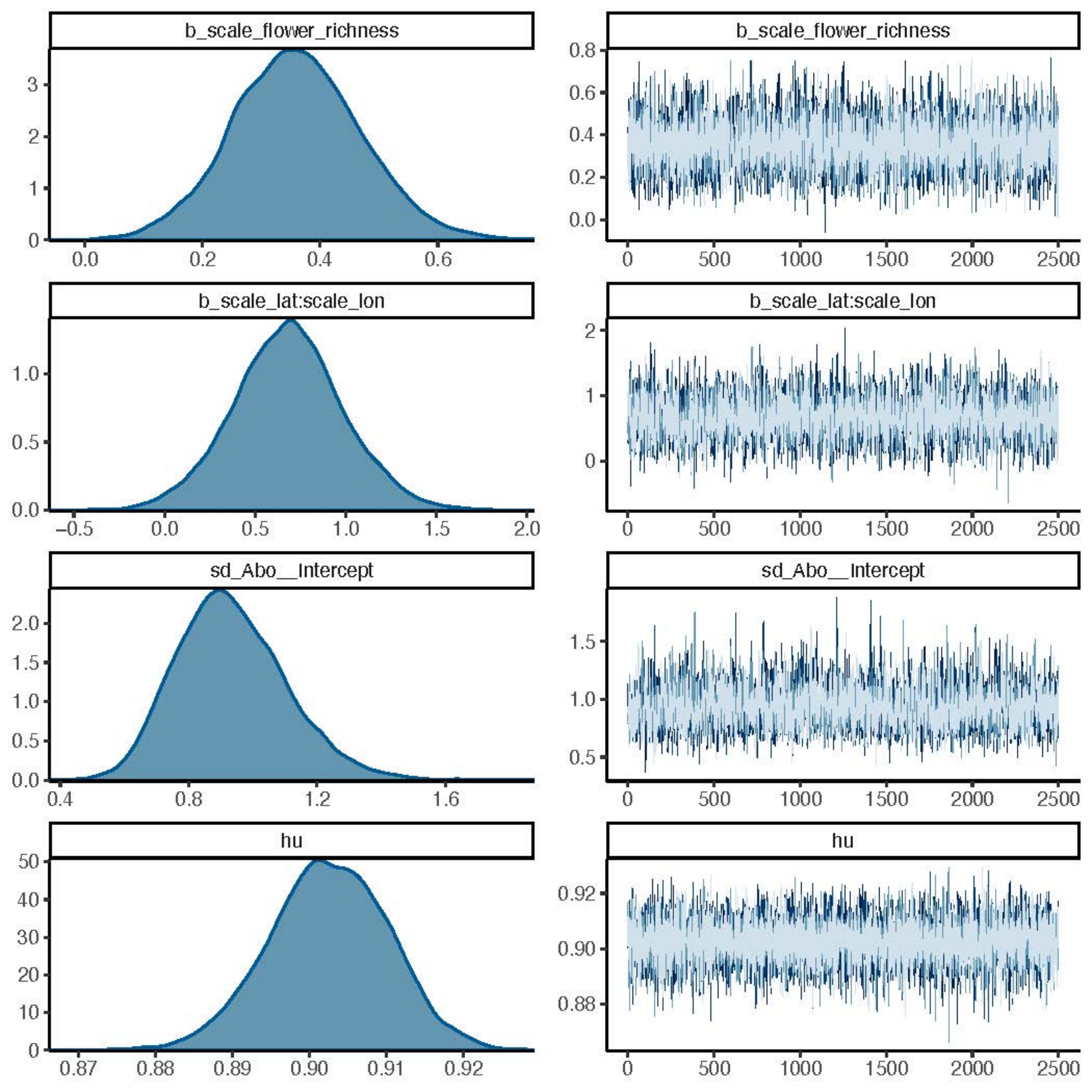
b_scale_flower_richness
b_scale_flower_richness
b_scale_lat:scale_lon
b_scale_lat:scale_lon
sd_Abo__Intercept
sd_Abo__Intercept
hu
hu
0 500 1000 1500 2000 2500

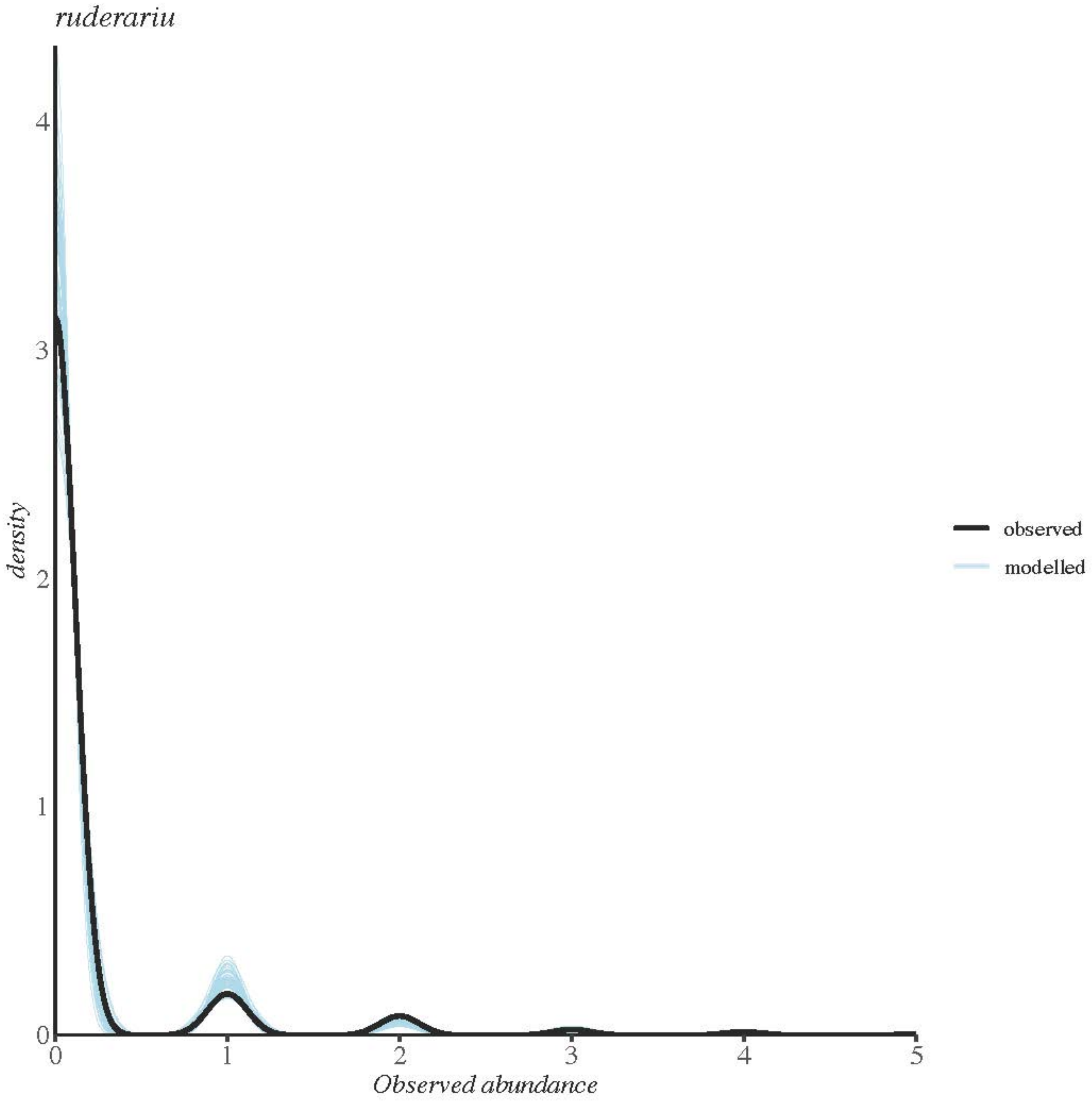
ruderariu
density
4
3
2
1
0
0
1
2
3
4
5
Observed abundance
observed
modelled

sporadicu

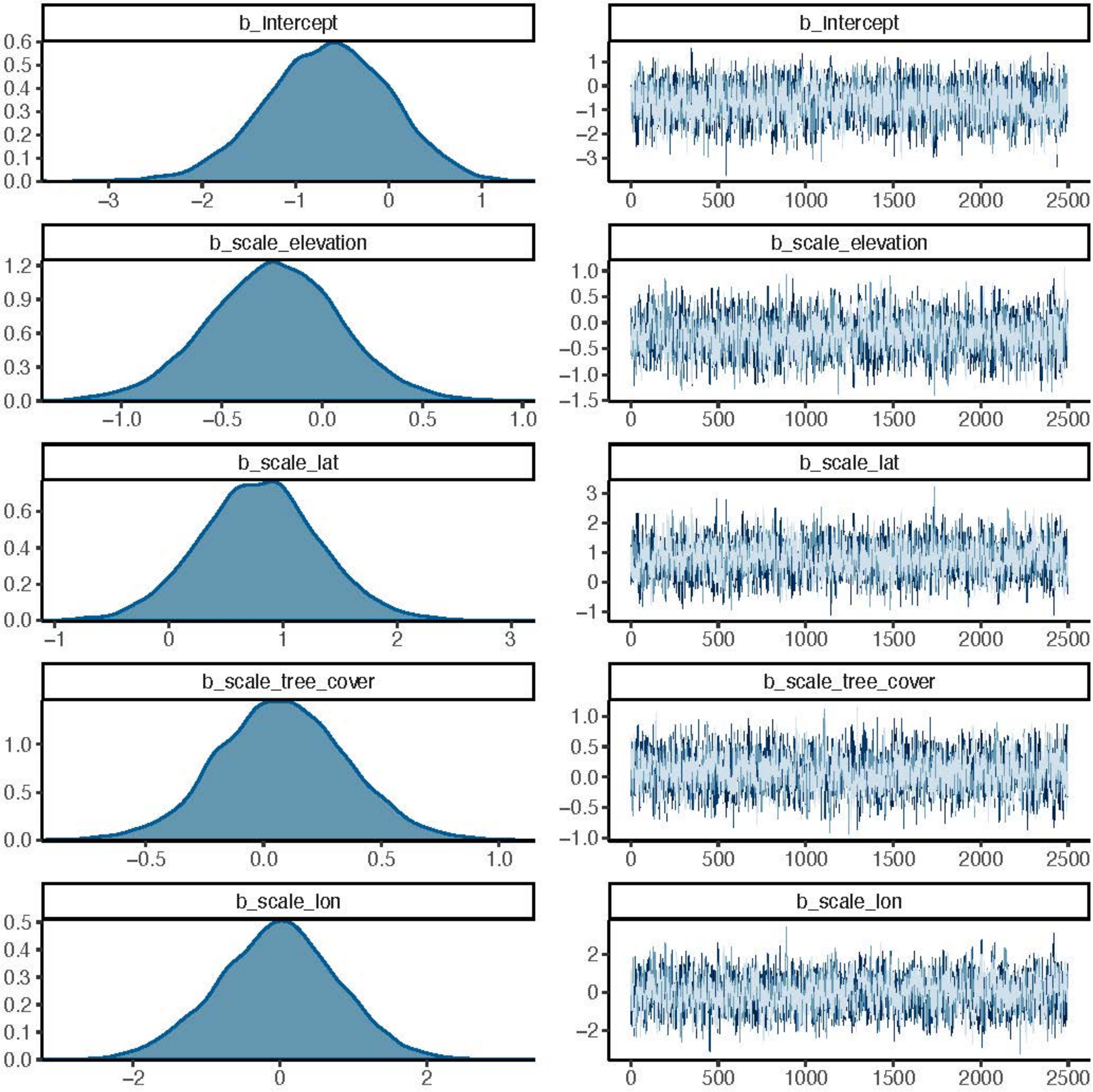
b_Intercept
b_Intercept
b_scale_elevation
b_scale_elevation
b_scale_lat
b_scale_lat
b_scale_tree_cover
b_scale_tree_cover
b_scale_lon
b_scale_lon

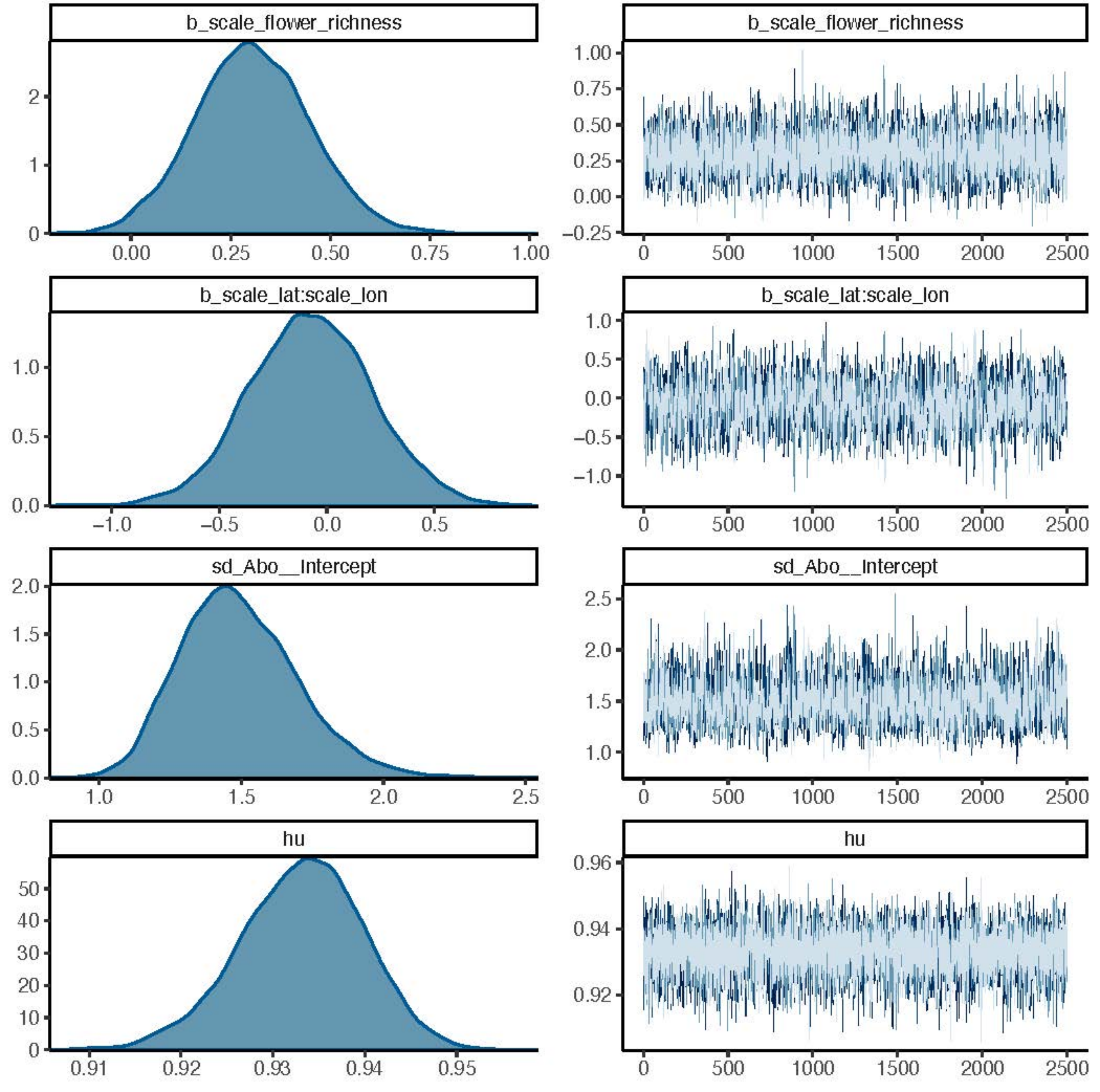
b_scale_flower_richness
0.00
0.25
0.50
0.75
1.00
0
500
1000
1500
2000
2500
b_scale_lat:scale_lon
−1.0
−0.5
0.0
0.5
1.0
sd_Abo__Intercept
1.0
1.5
2.0
2.5
hu
0.91
0.92
0.93
0.94
0.95
0.96

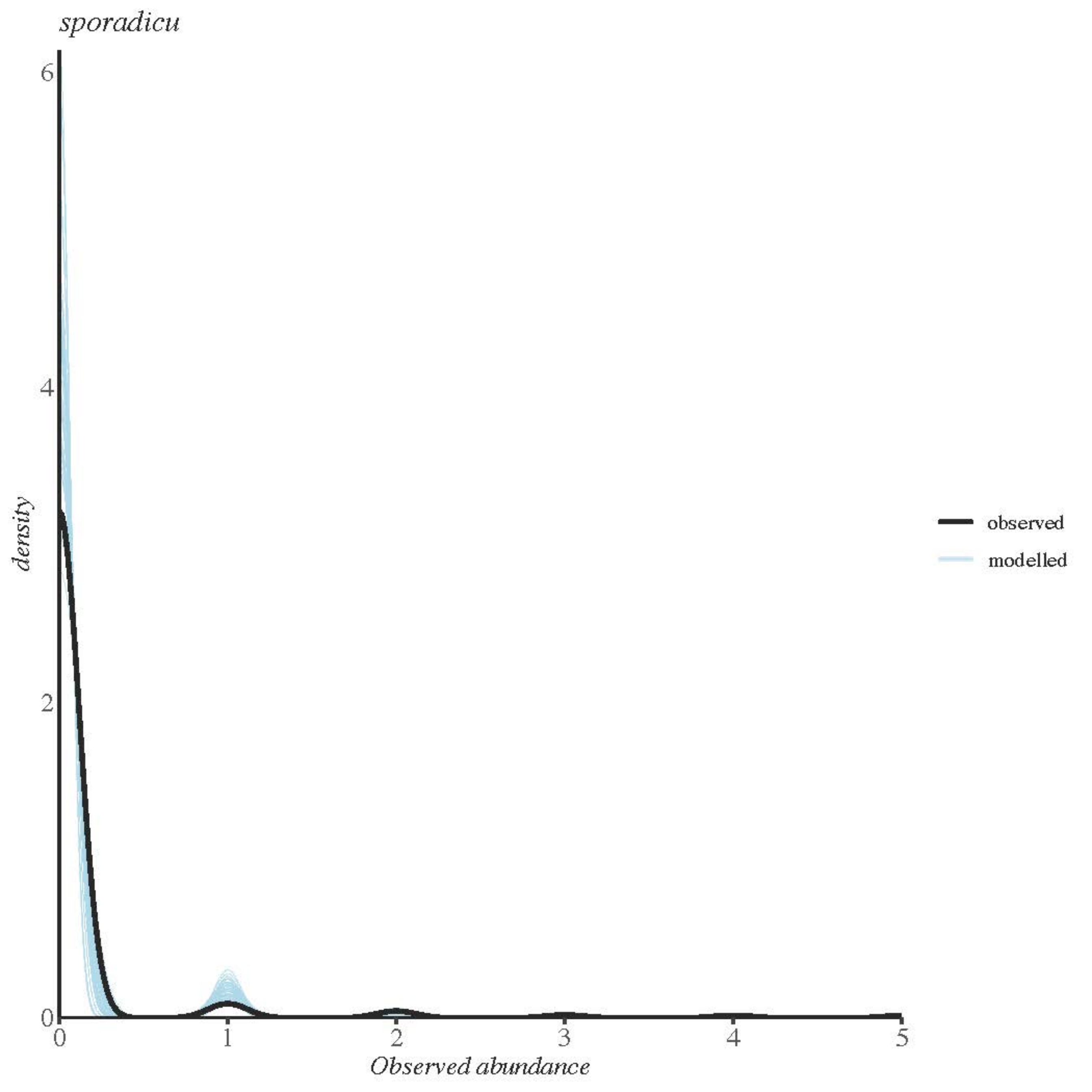
sporadicu
density
6
4
2
0
0
1
2
3
4
5
Observed abundance
observed
modelled

hortorum

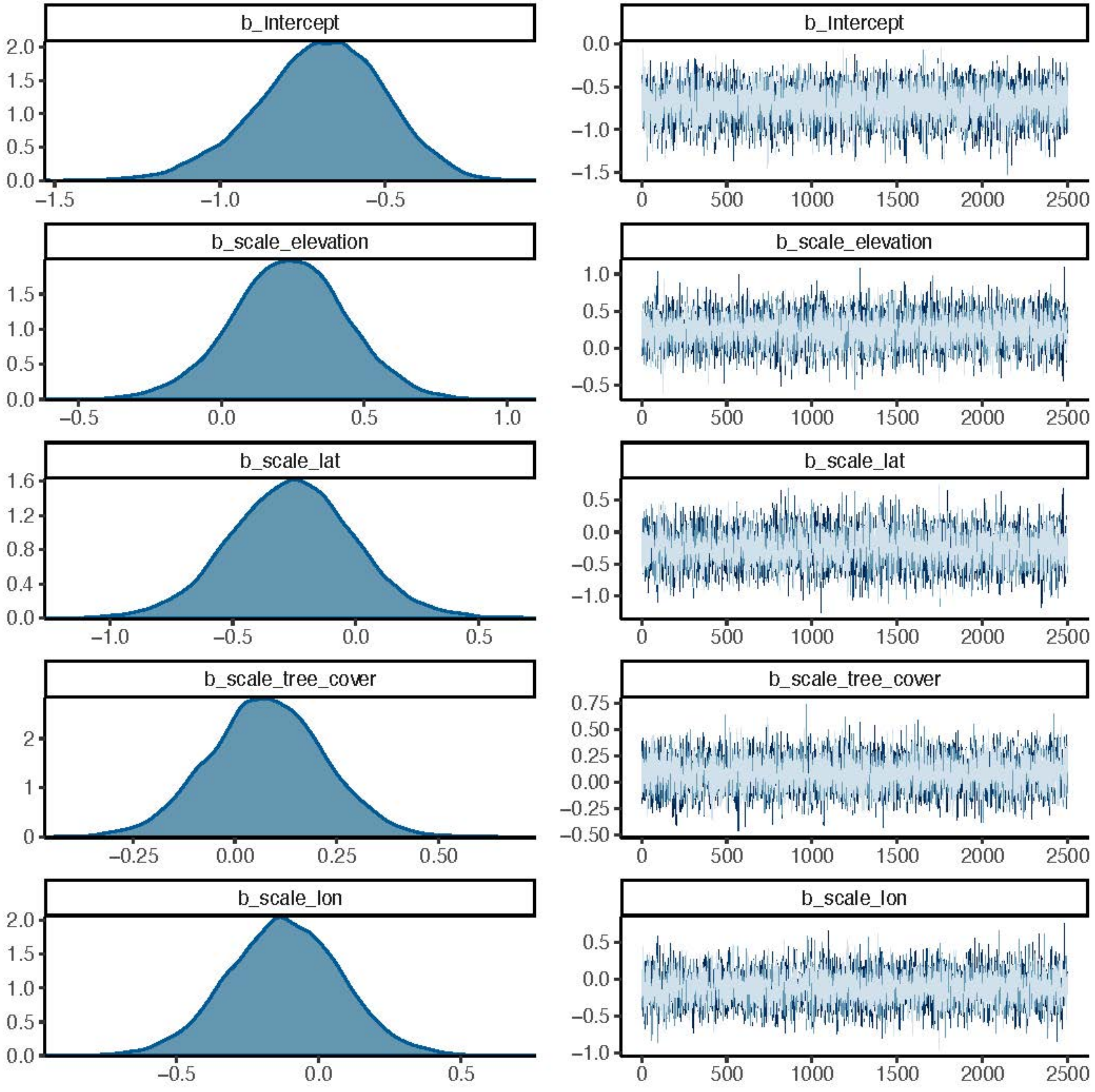
b_Intercept
b_Intercept
b_scale_elevation
b_scale_elevation
b_scale_lat
b_scale_lat
b_scale_tree_cover
b_scale_tree_cover
b_scale_lon
b_scale_lon

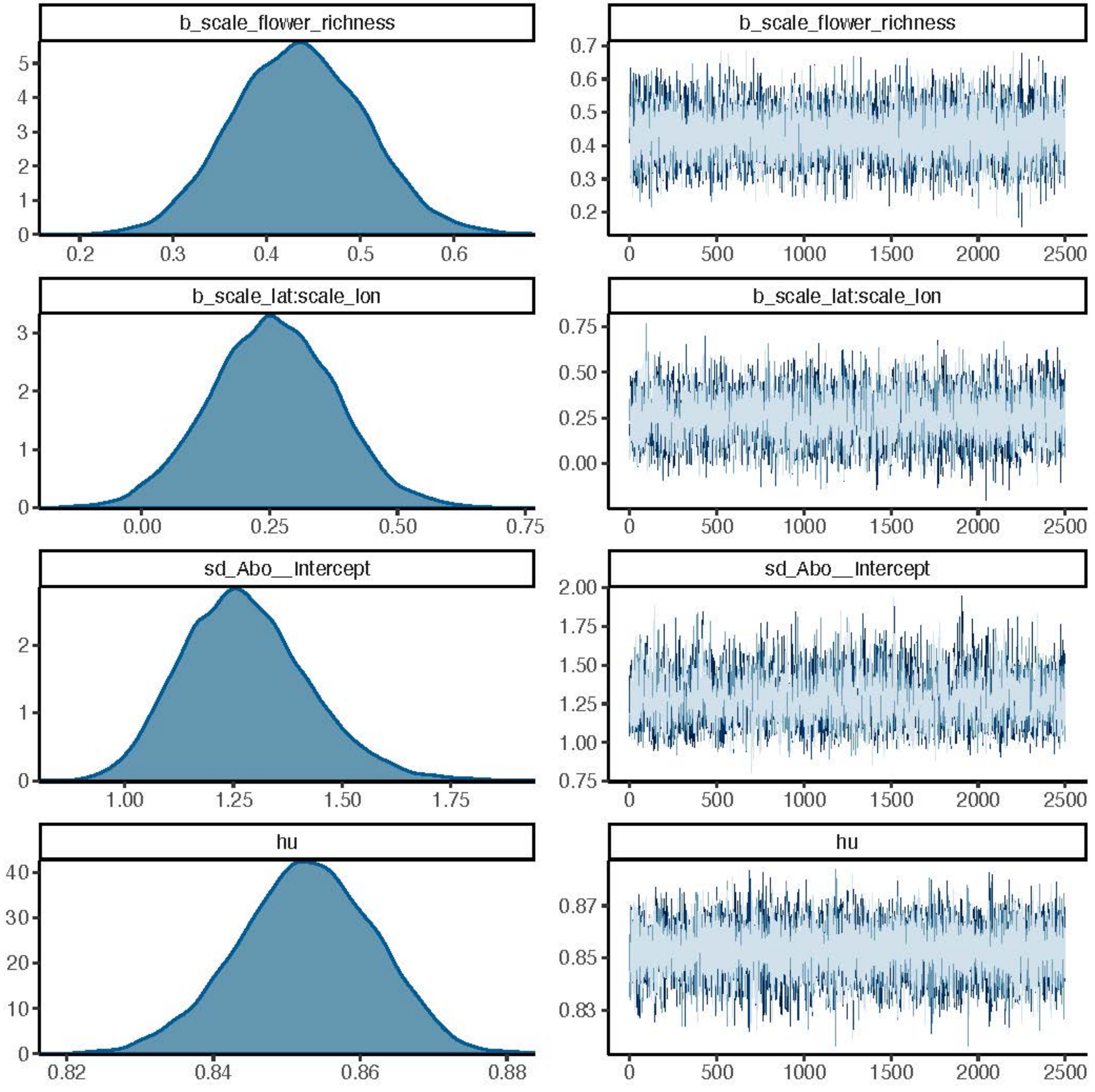
b_scale_flower_richness
0
1
2
3
4
5
0.2
0.3
0.4
0.5
0.6
b_scale_flower_richness
0.2
0.3
0.4
0.5
0.6
0.7
0
500
1000
1500
2000
2500
b_scale_lat:scale_lon
0
1
2
3
0.00
0.25
0.50
0.75
b_scale_lat:scale_lon
0.00
0.25
0.50
0.75
0
500
1000
1500
2000
2500
sd_Abo__Intercept
0
1
2
1.00
1.25
1.50
1.75
sd_Abo__Intercept
0.75
1.00
1.25
1.50
1.75
2.00
0
500
1000
1500
2000
2500
hu
0
10
20
30
40
0.82
0.84
0.86
0.88
hu
0.83
0.85
0.87
0
500
1000
1500
2000
2500

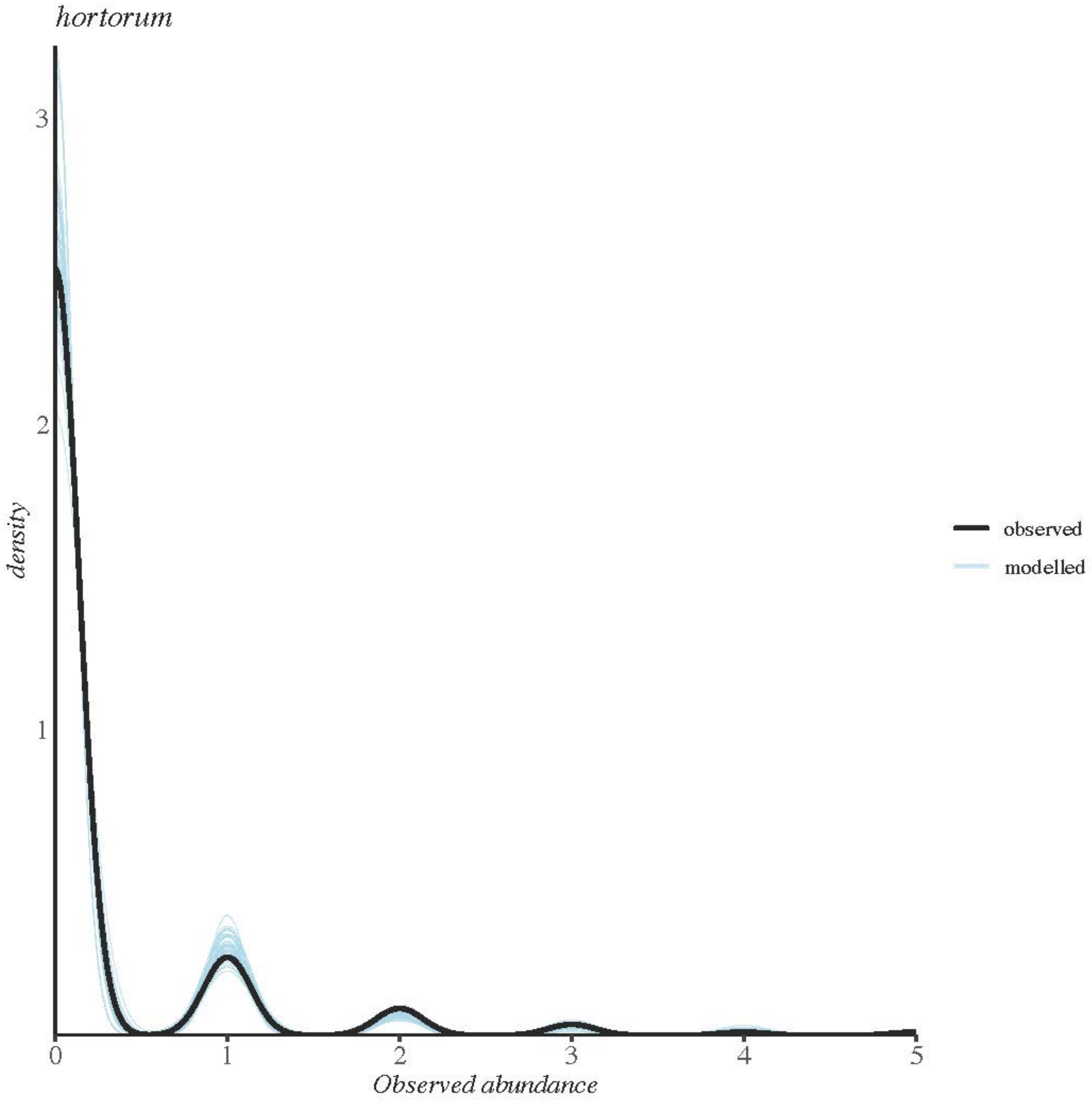
hortorum
density
3
2
1
0
1
2
3
4
5
Observed abundance
observed
modelled

hypnorum

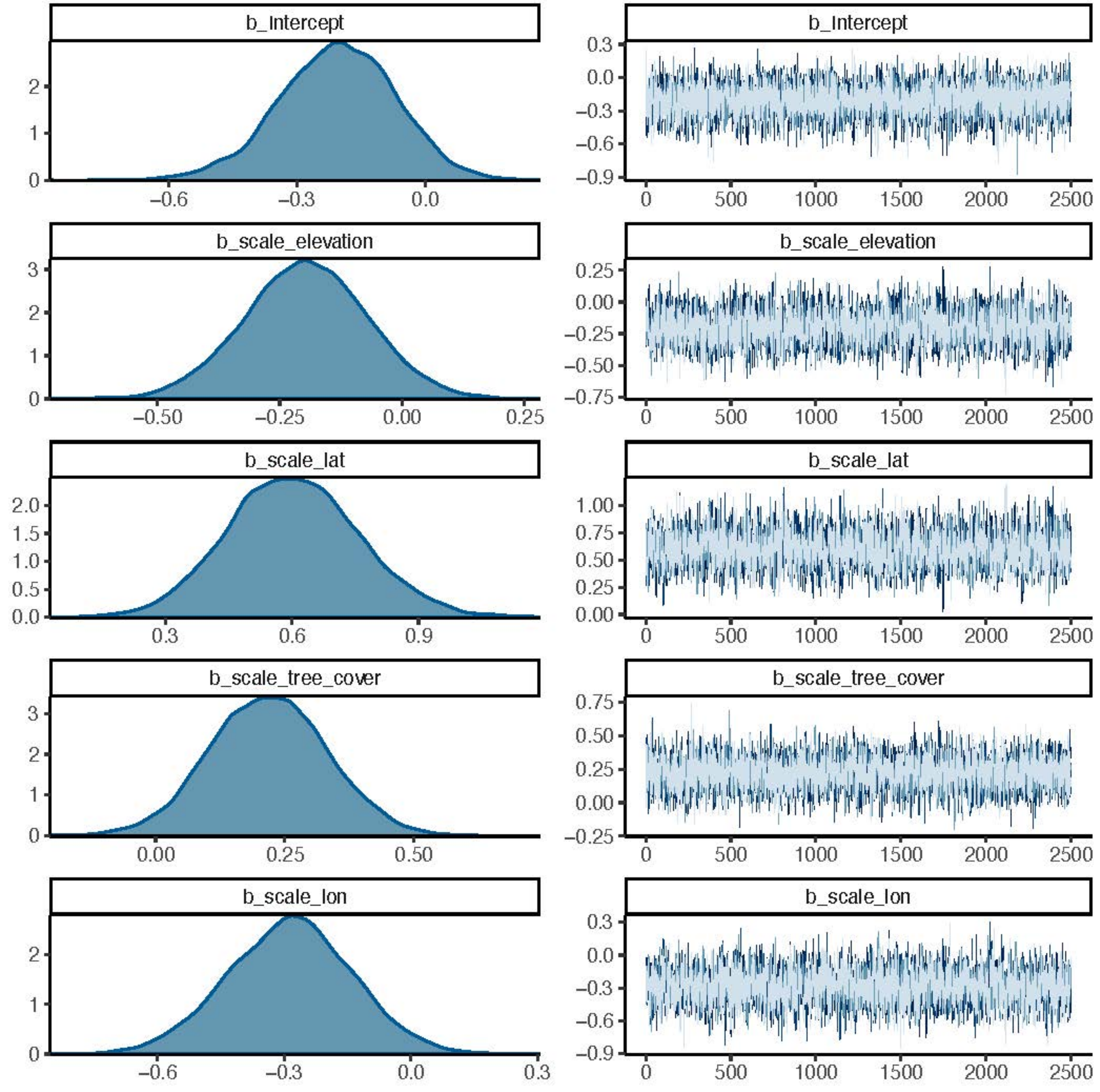

b_Intercept
b_scale_elevation
b_scale_lat
b_scale_tree_cover
b_scale_lon

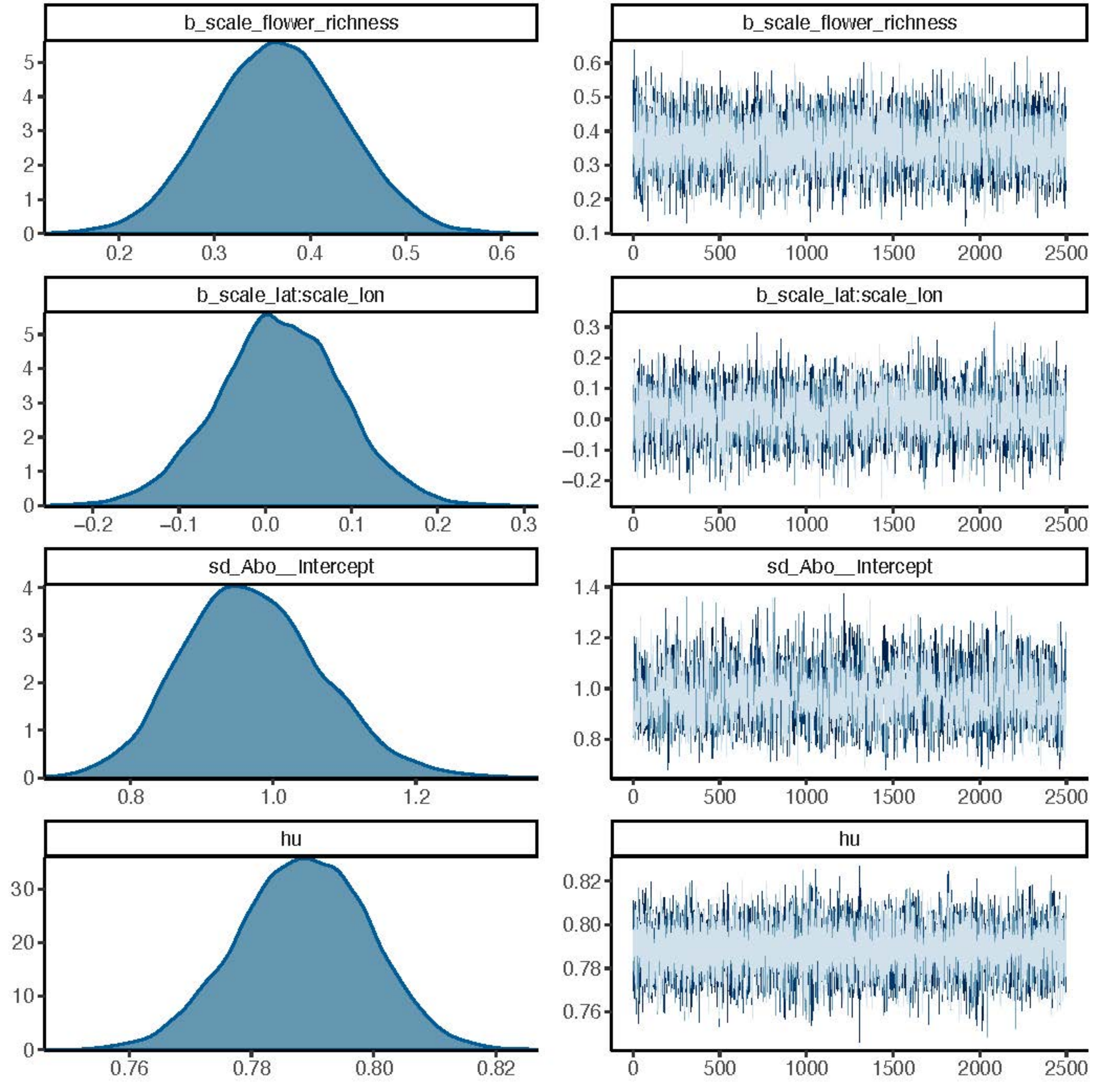

b_scale_flower_richness
b_scale_flower_richness
b_scale_lat:scale_lon
b_scale_lat:scale_lon
sd_Abo__Intercept
sd_Abo__Intercept
hu
hu

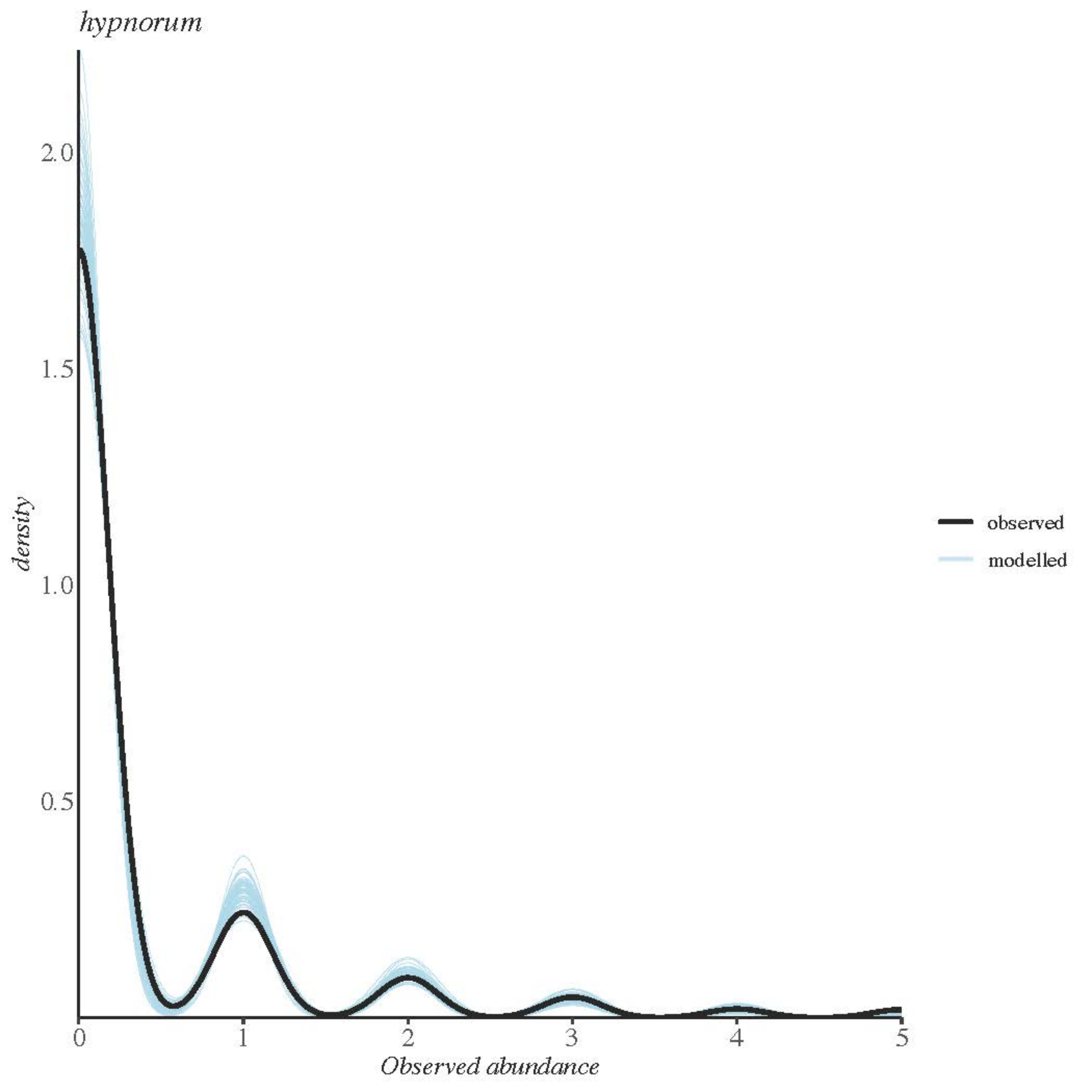

hypnorum
density
2.0
1.5
1.0
0.5
0
1
2
3
4
5
Observed abundance
observed
modelled

sylvarum

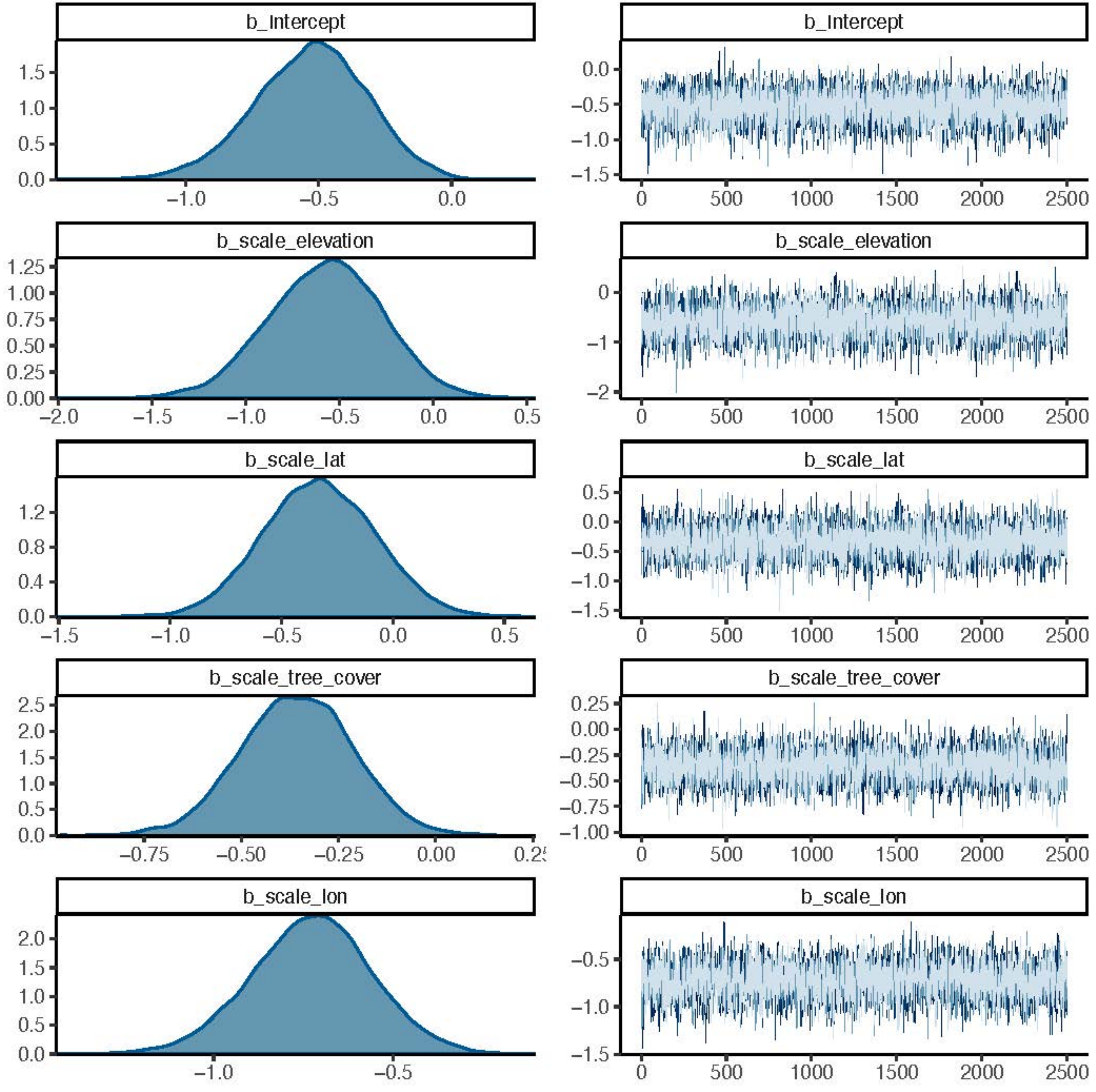
b_Intercept
b_Intercept
b_scale_elevation
b_scale_elevation
b_scale_lat
b_scale_lat
b_scale_tree_cover
b_scale_tree_cover
b_scale_lon
b_scale_lon

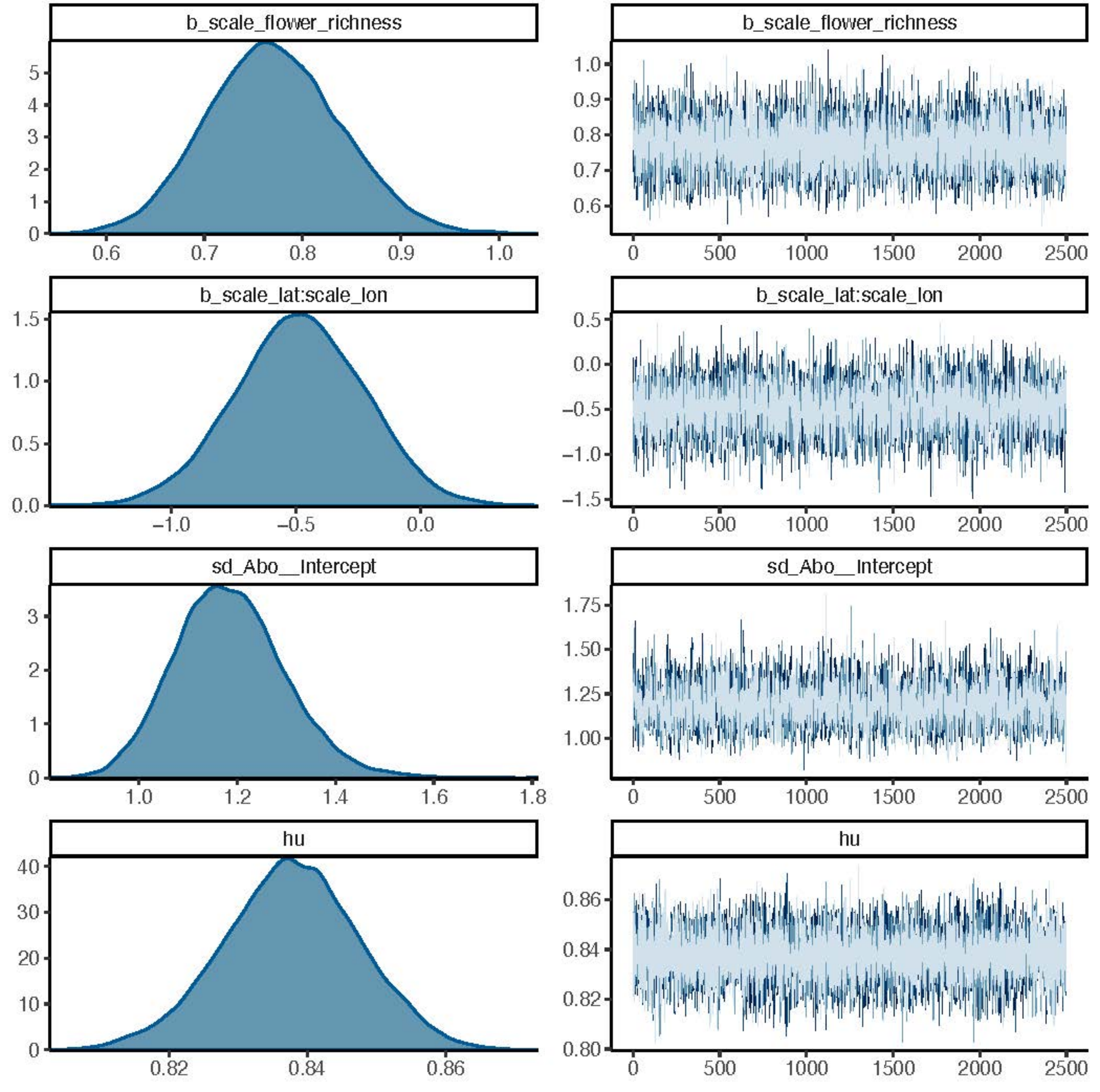

b_scale_flower_richness
b_scale_flower_richness
b_scale_lat:scale_lon
b_scale_lat:scale_lon
sd_Abo__Intercept
sd_Abo__Intercept
hu
hu

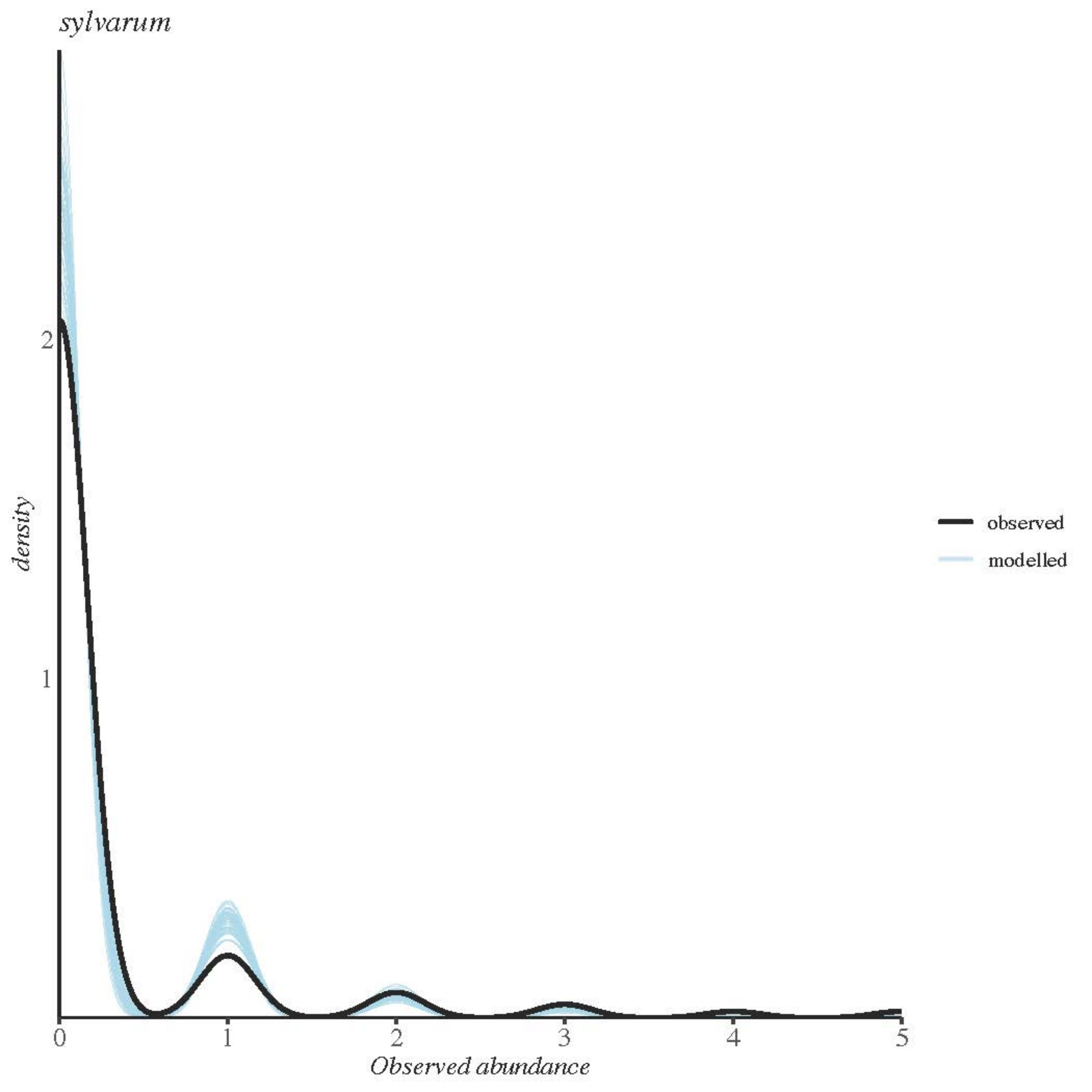

sylvarum
density
2
1
0
1
2
3
4
5
Observed abundance
observed
modelled

jonellus

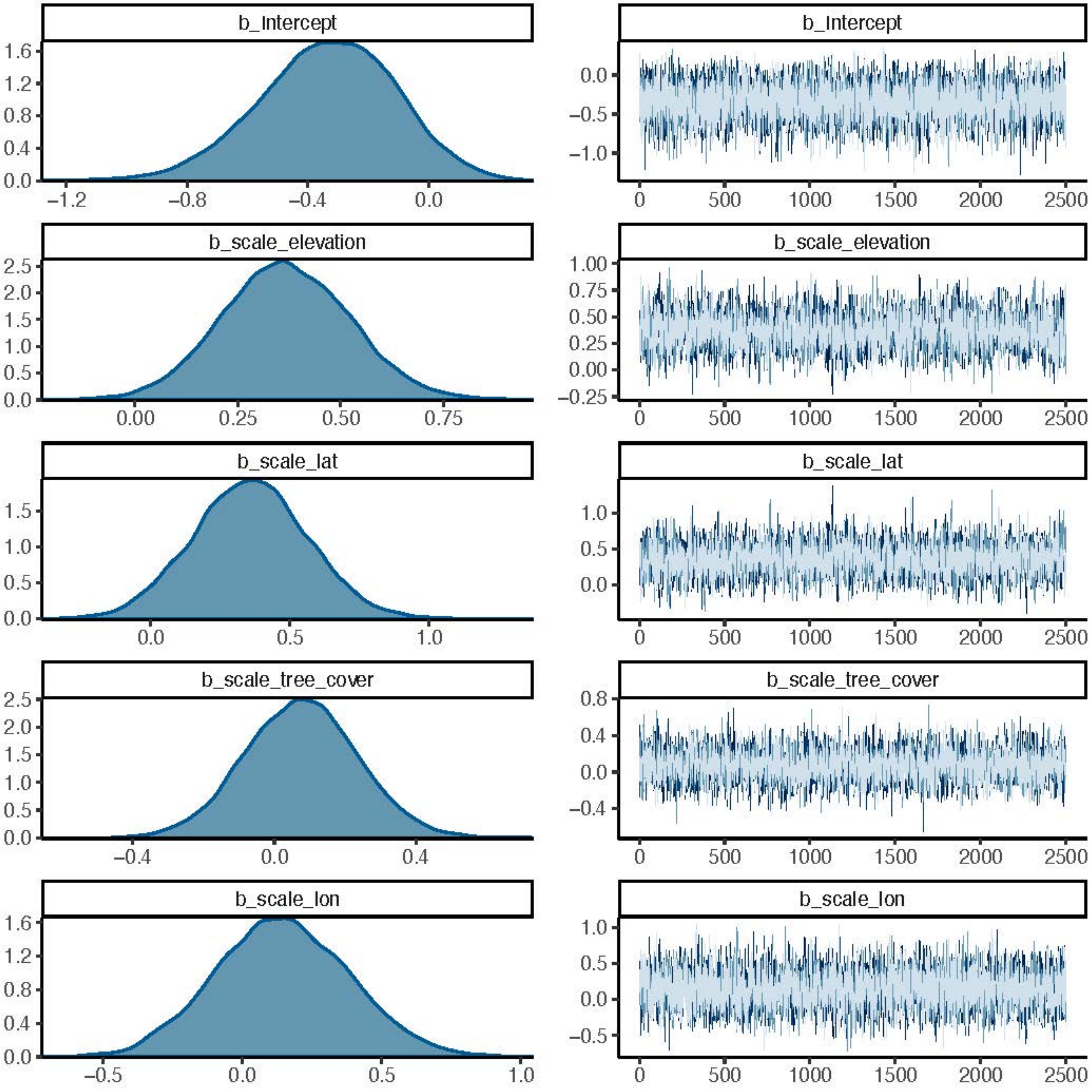

b_Intercept
b_scale_elevation
b_scale_lat
b_scale_tree_cover
b_scale_lon

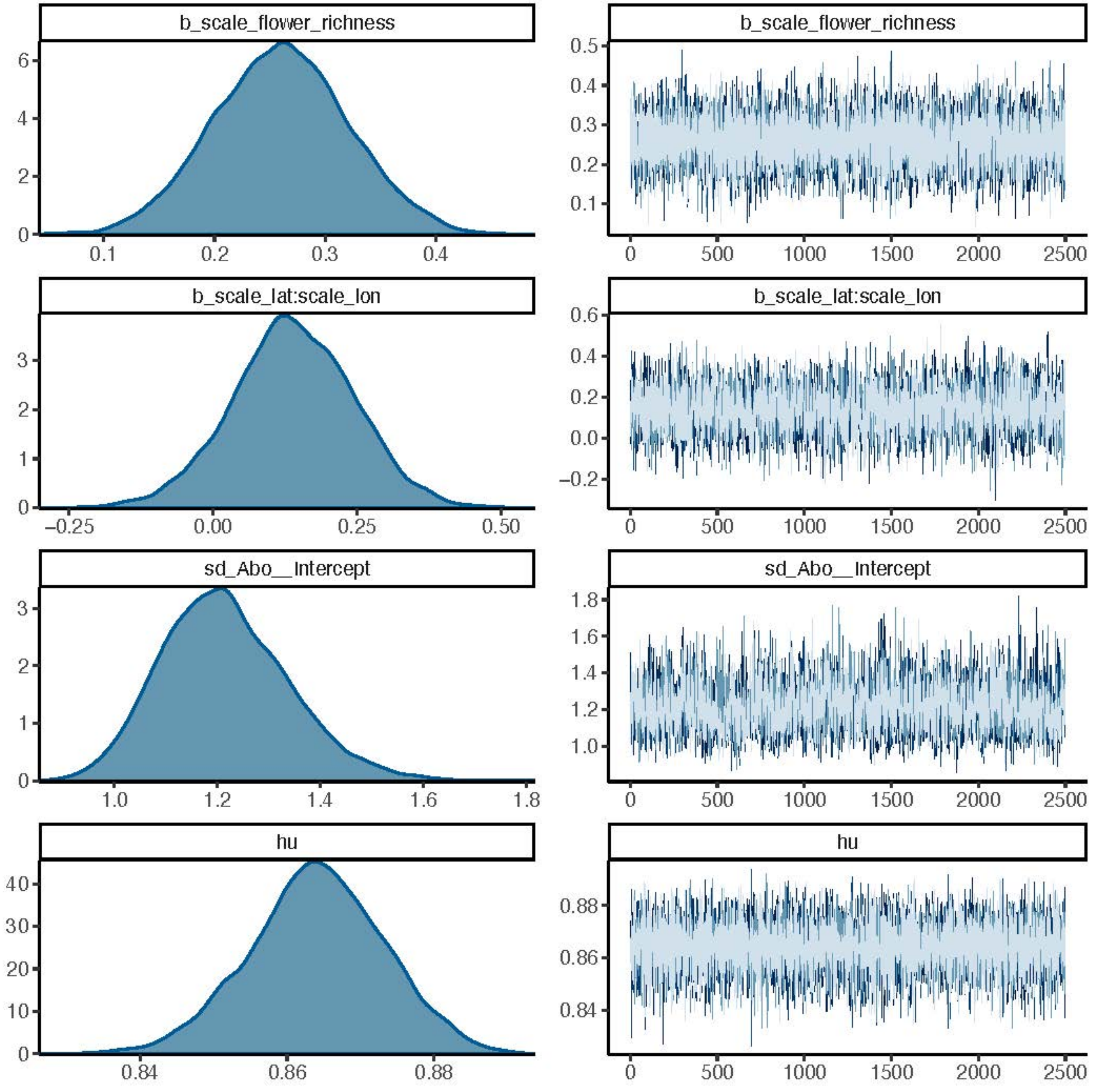

b_scale_flower_richness
b_scale_flower_richness
b_scale_lat:scale_lon
b_scale_lat:scale_lon
sd_Abo__Intercept
sd_Abo__Intercept
hu
hu

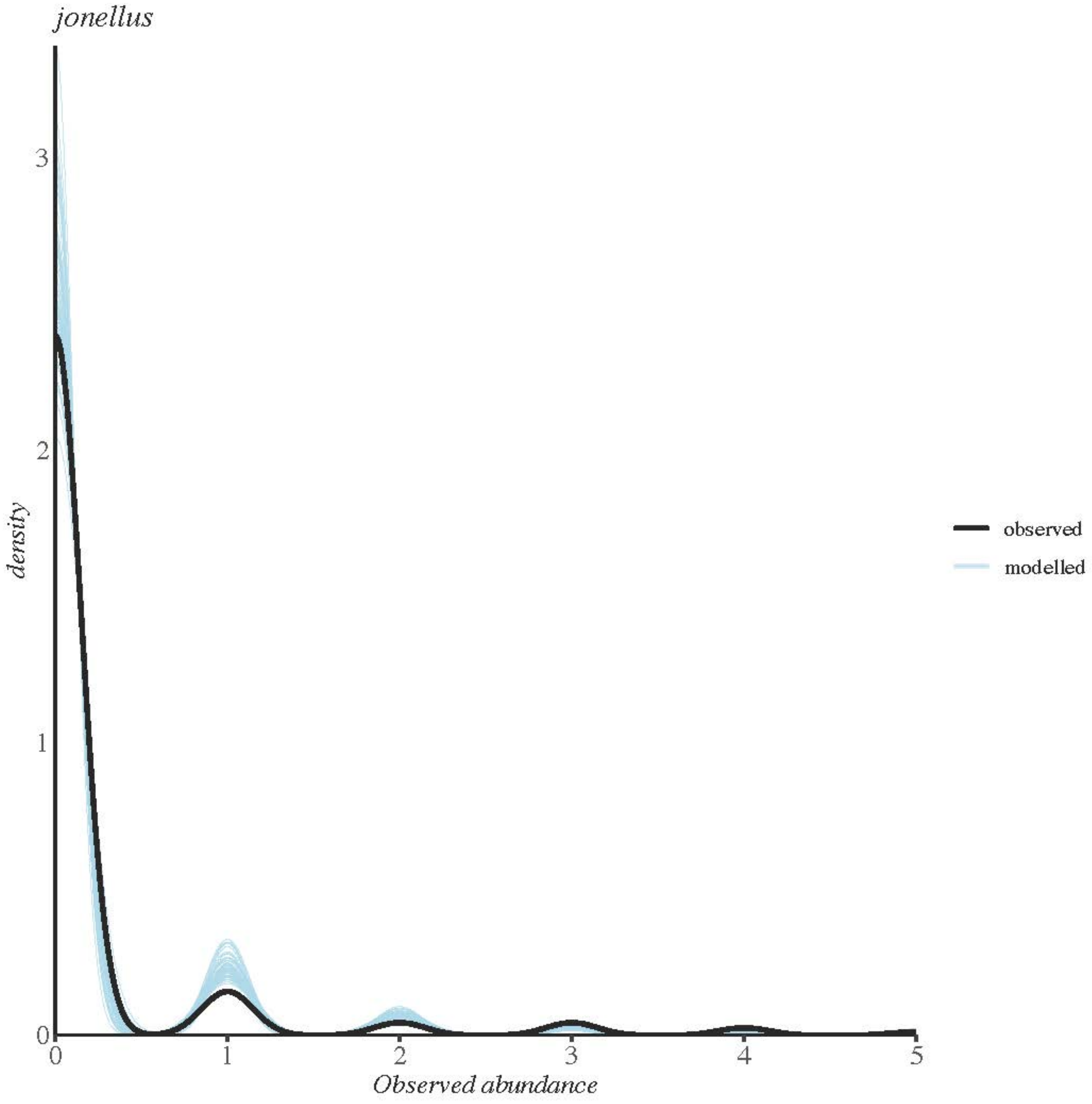
jonellus
density
Observed abundance
observed
modelled
0
1
2
3
4
5
0
1
2
3

soroeensi

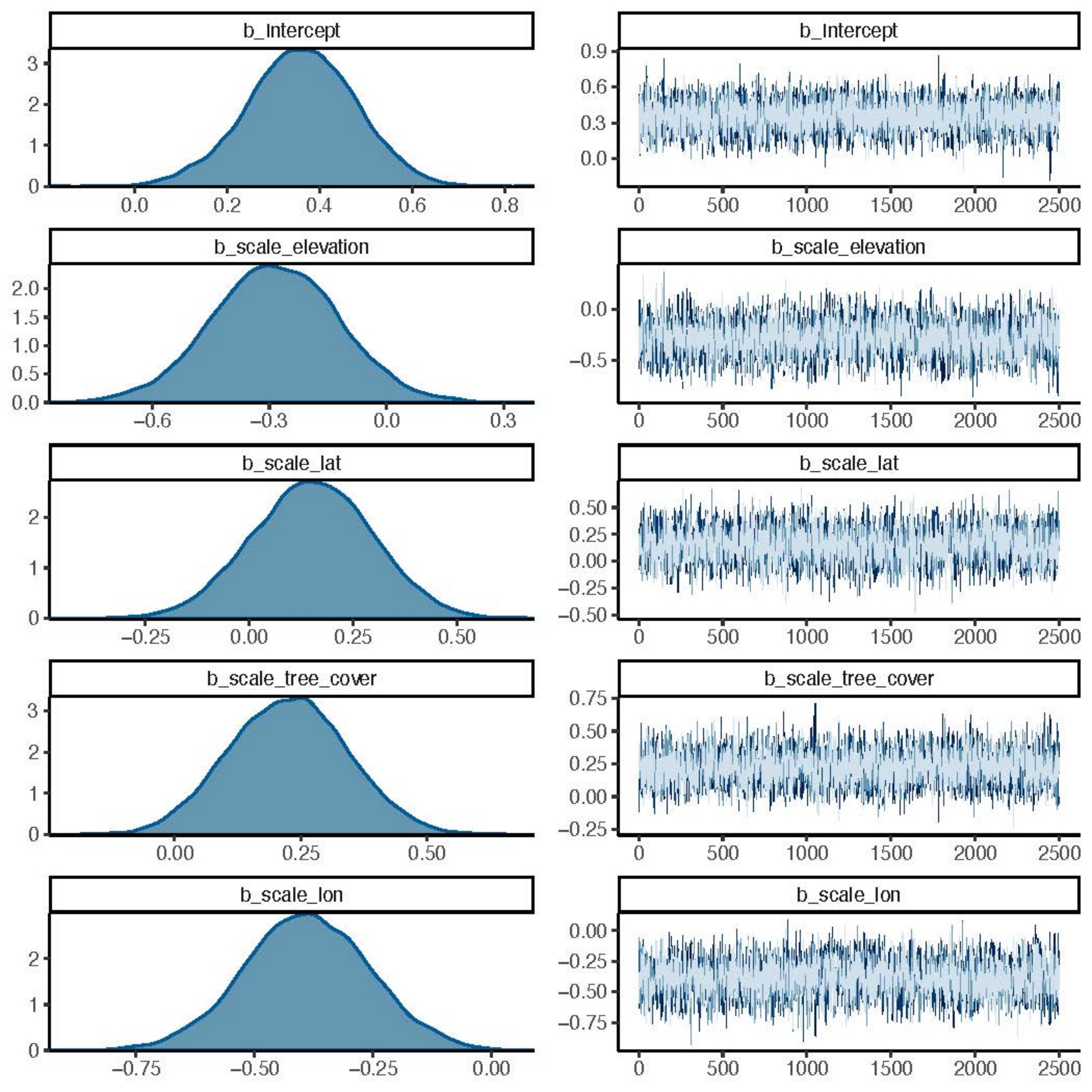

b_Intercept
b_scale_elevation
b_scale_lat
b_scale_tree_cover
b_scale_lon

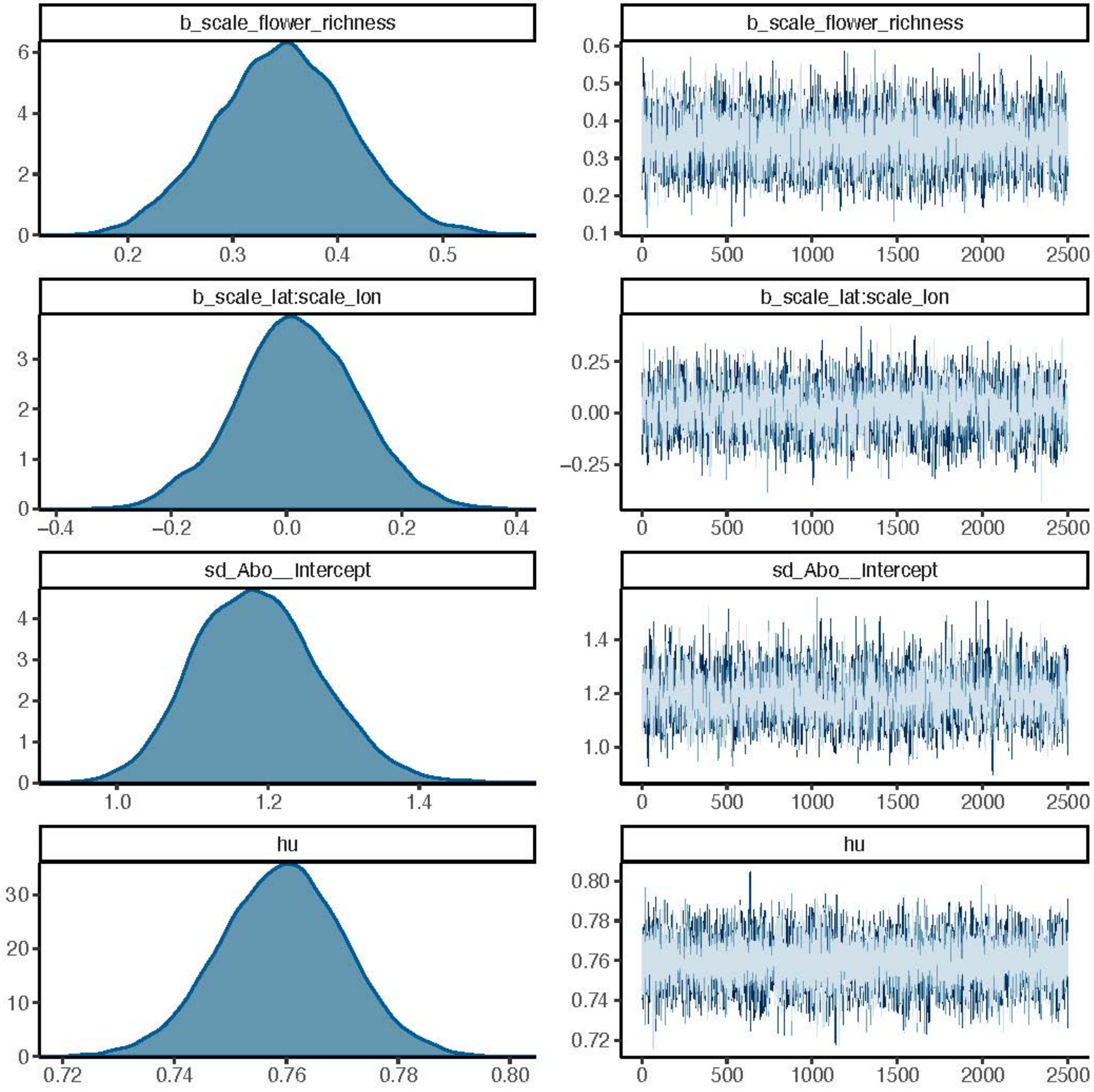

b_scale_flower_richness
b_scale_lat:scale_lon
sd_Abo__Intercept
hu

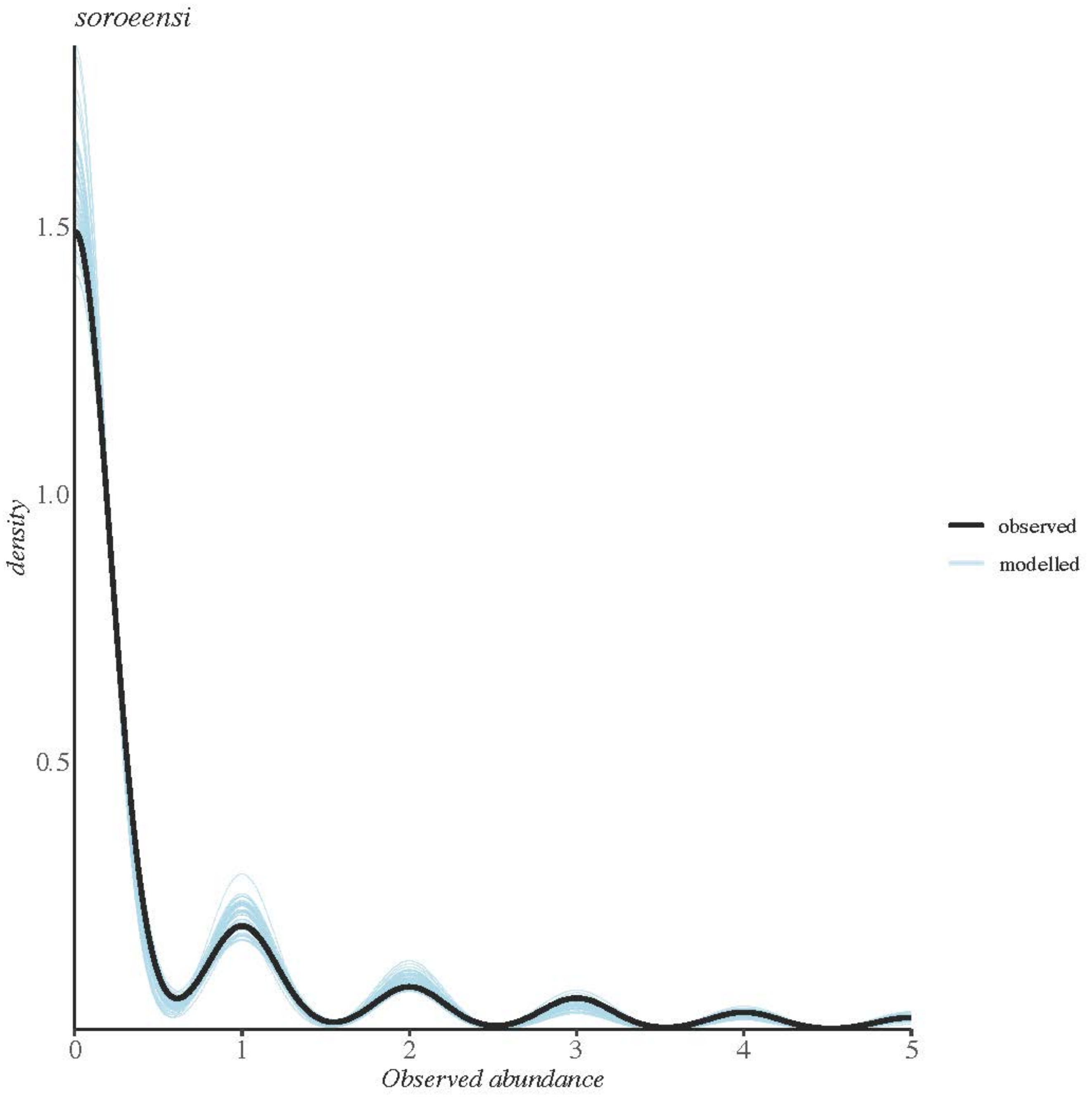
soroeensi
1.5
1.0
0.5
density
0
1
2
3
4
5
Observed abundance
observed
modelled

lapidariu

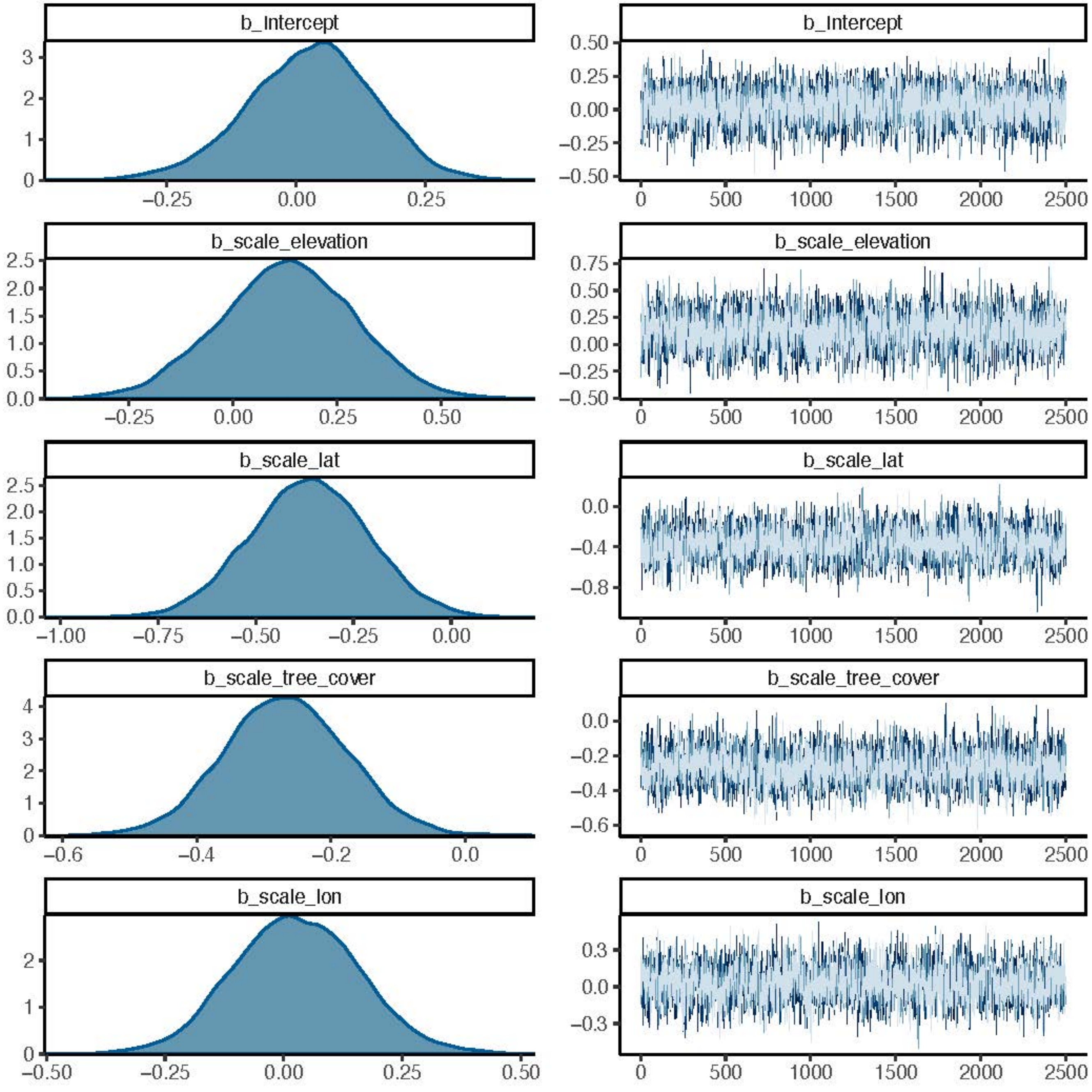
b_Intercept
b_Intercept
b_scale_elevation
b_scale_elevation
b_scale_lat
b_scale_lat
b_scale_tree_cover
b_scale_tree_cover
b_scale_lon
b_scale_lon

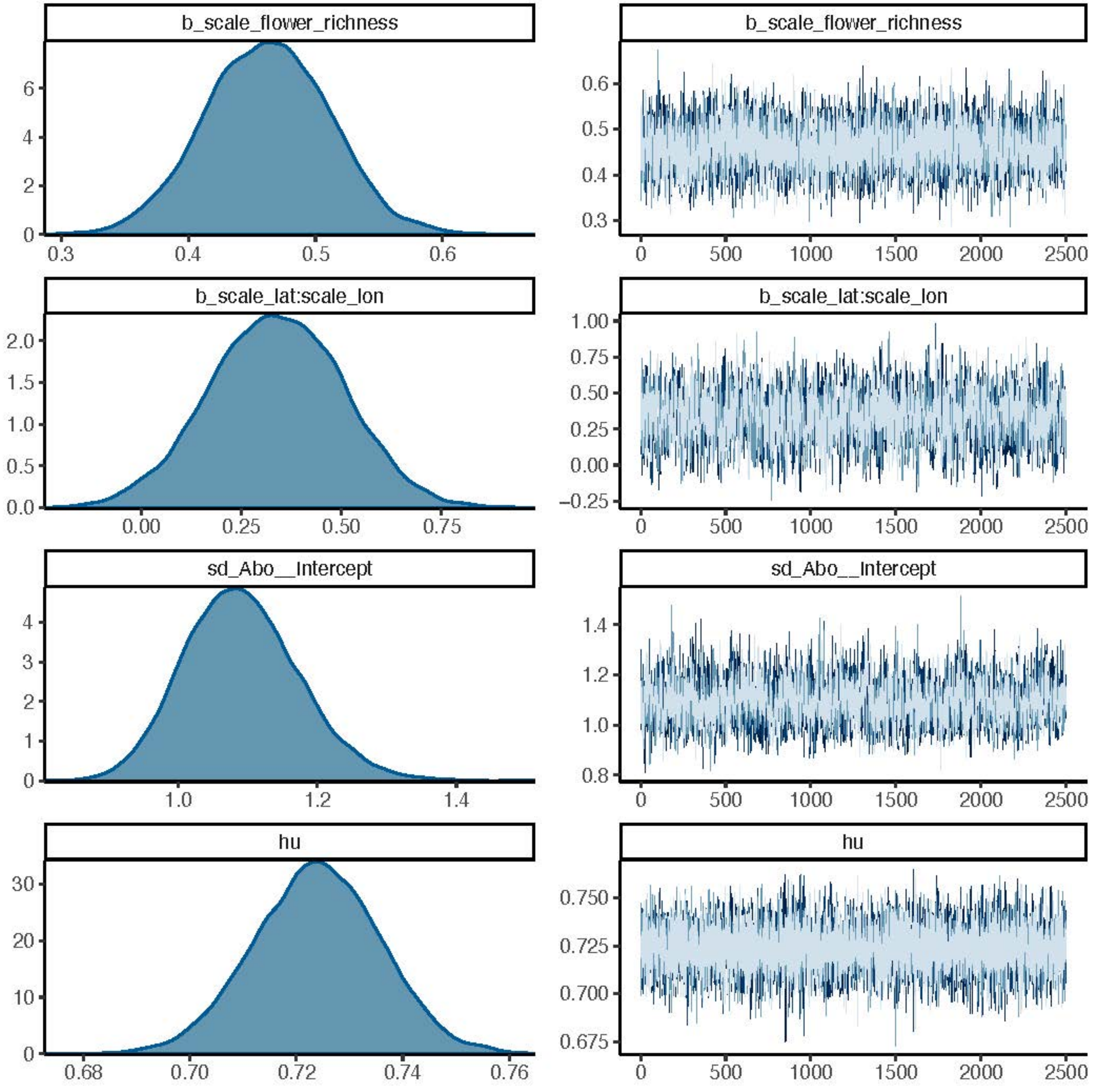

b_scale_flower_richness
b_scale_lat:scale_lon
sd_Abo__Intercept
hu

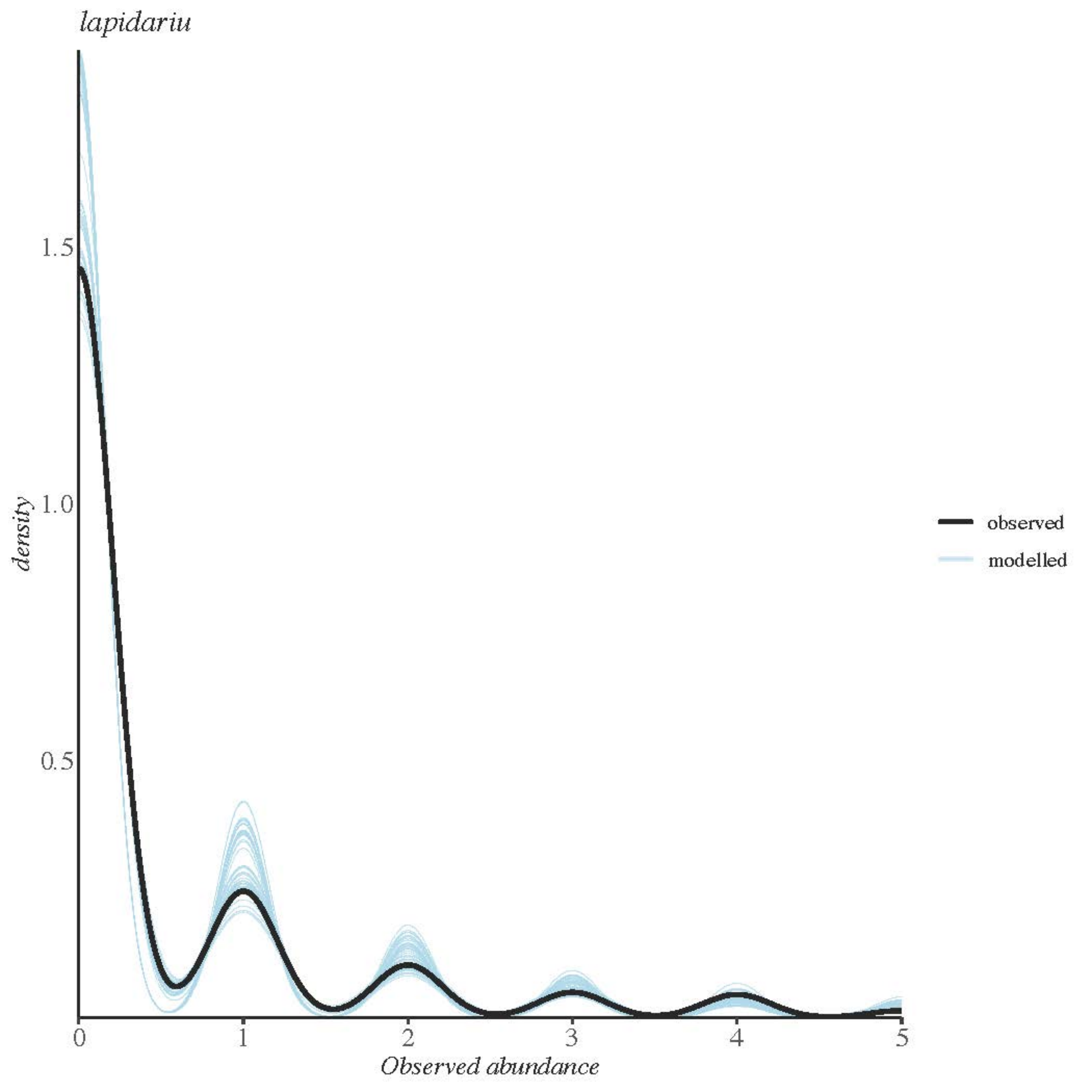

lapidariu
1.5
1.0
0.5
density
0
1
2
3
4
5
Observed abundance
observed
modelled

terrestri

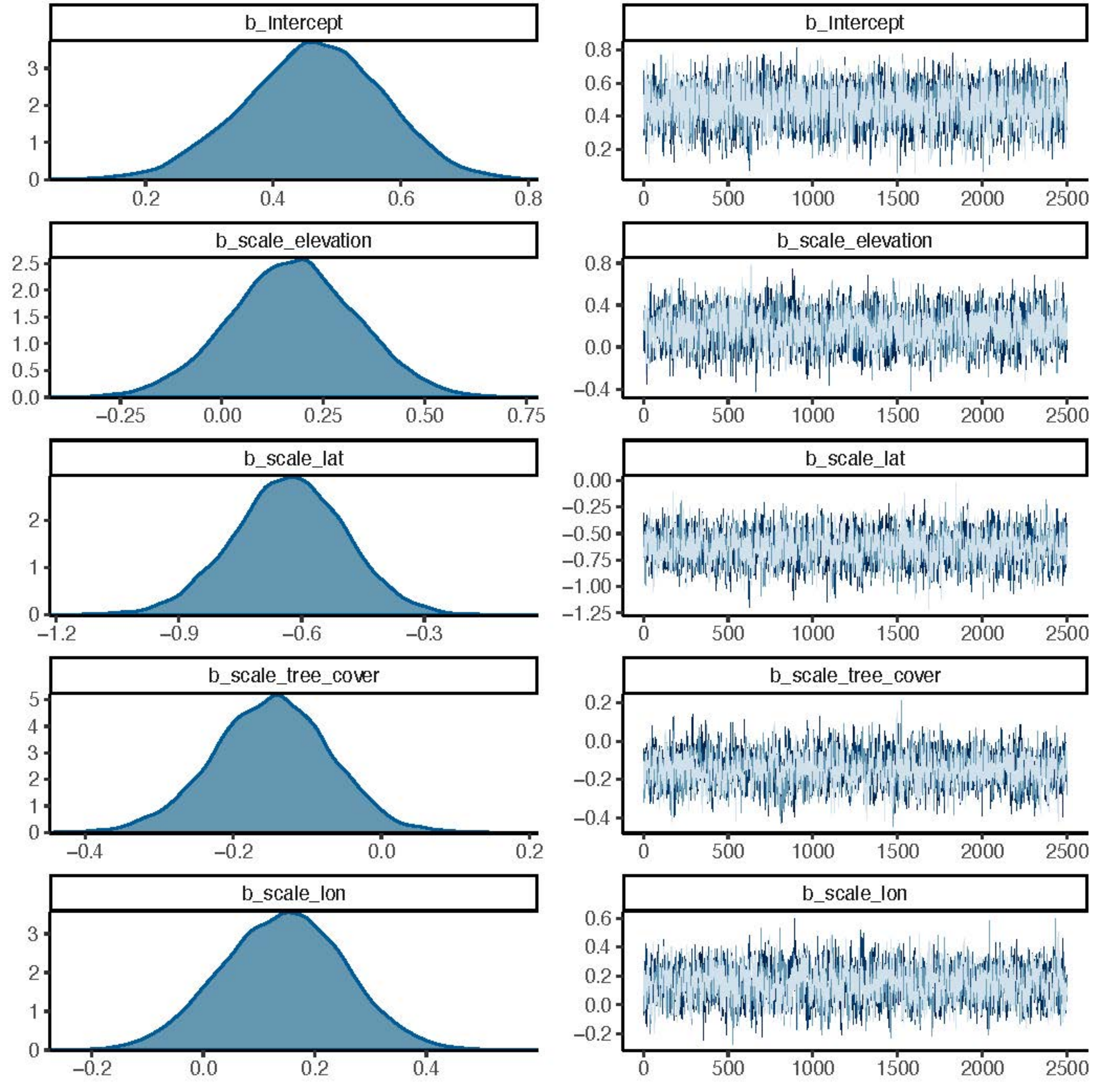

b_Intercept
b_Intercept
b_scale_elevation
b_scale_elevation
b_scale_lat
b_scale_lat
b_scale_tree_cover
b_scale_tree_cover
b_scale_lon
b_scale_lon

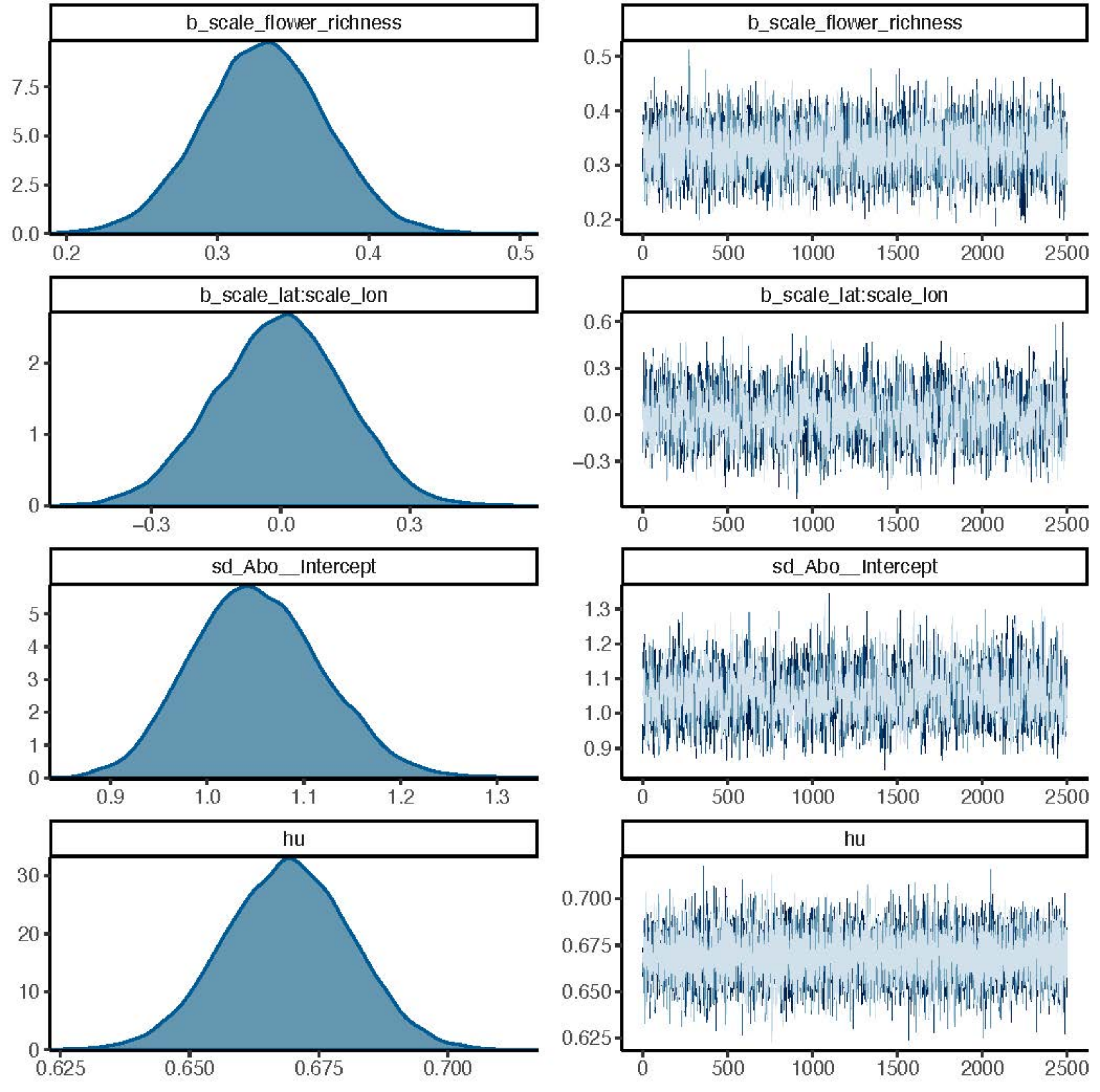
b_scale_flower_richness
b_scale_lat:scale_lon
sd_Abo__Intercept
hu
0.2
0.3
0.4
0.5
0.0
2.5
5.0
7.5
−0.3
0.0
0.3
0.6
0.9
1.0
1.1
1.2
1.3
0.625
0.650
0.675
0.700
0
500
1000
1500
2000
2500

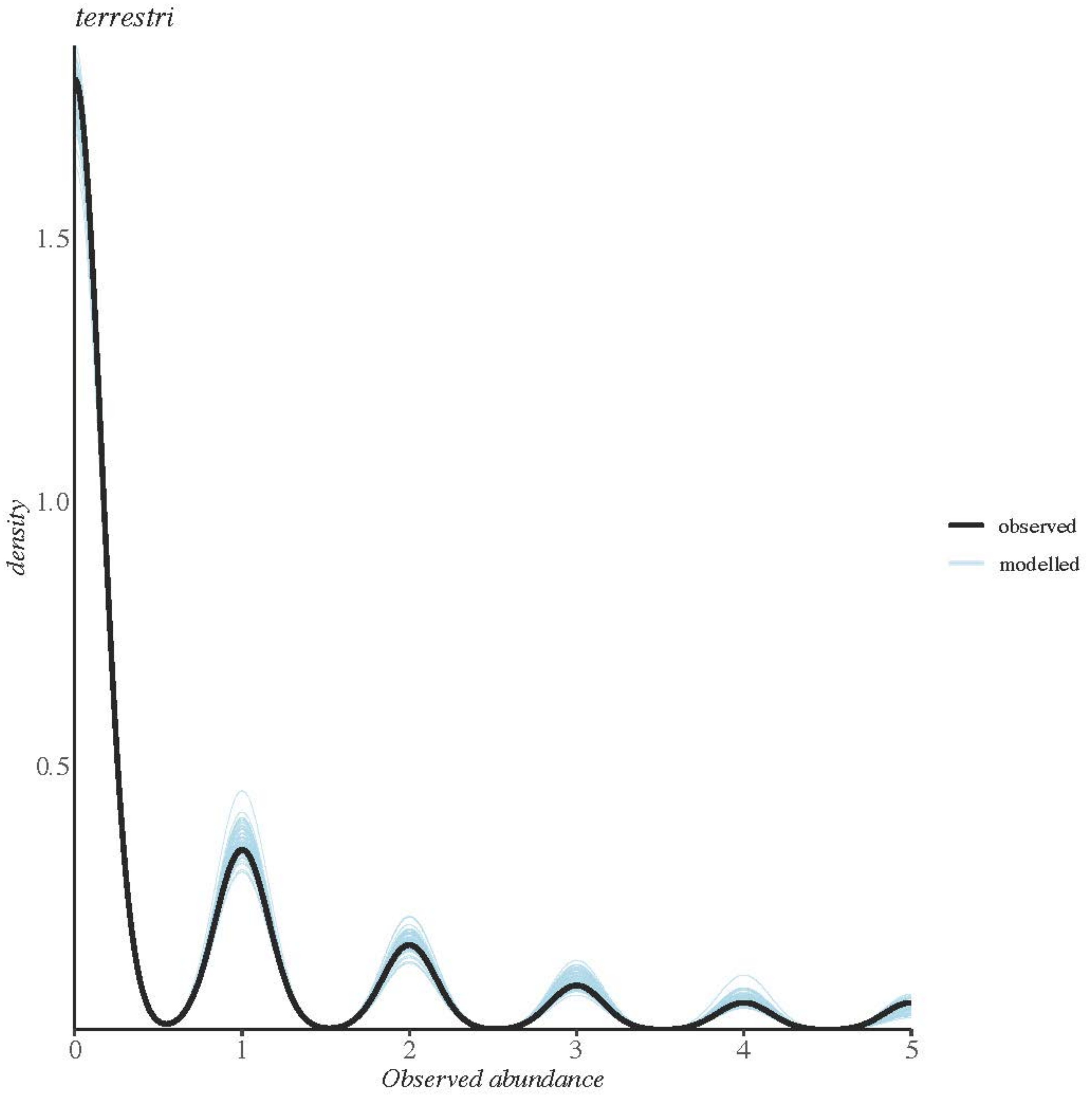
terrestri
density
1.5
1.0
0.5
0
1
2
3
4
5
Observed abundance
observed
modelled

pratorum

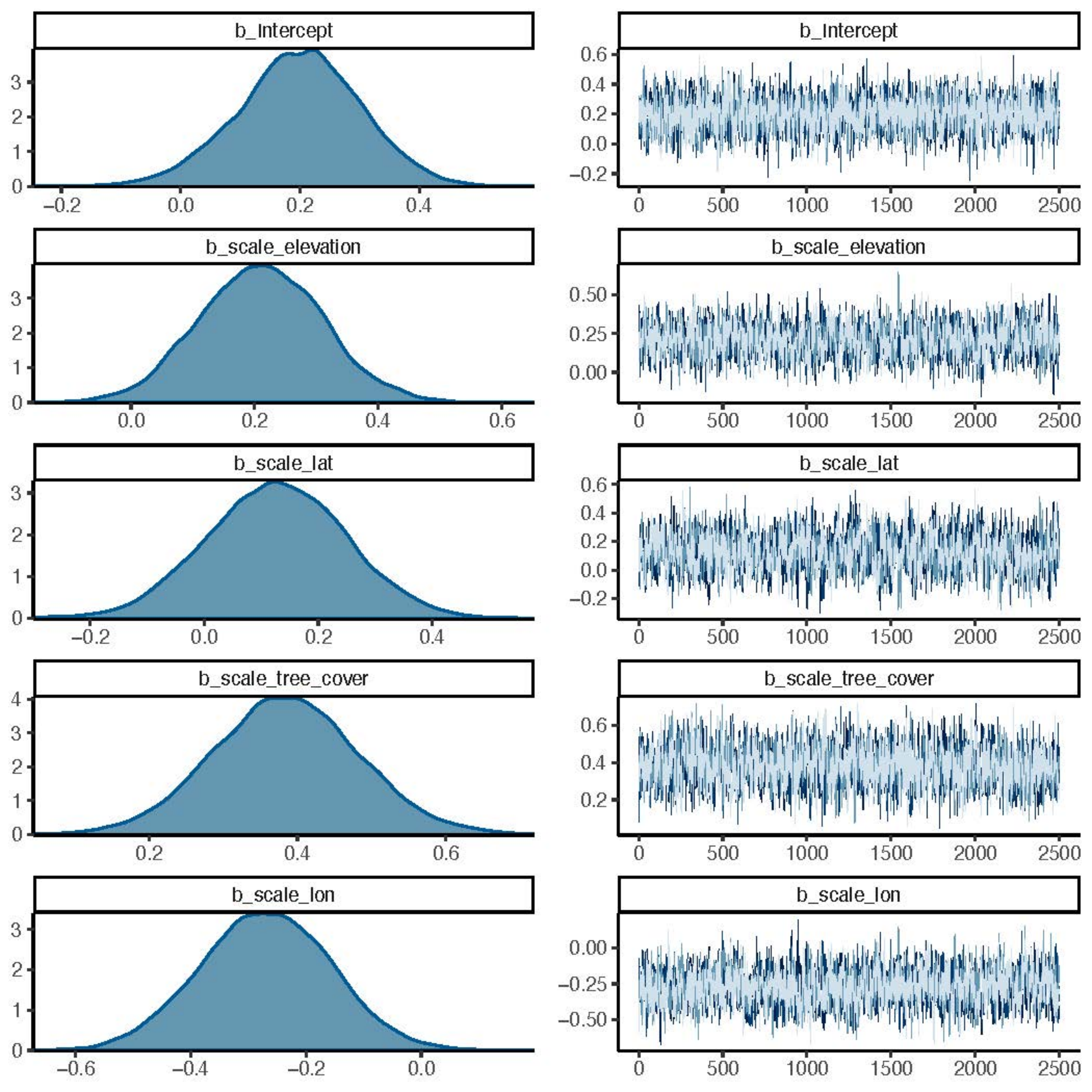
b_Intercept
b_scale_elevation
b_scale_lat
b_scale_tree_cover
b_scale_lon

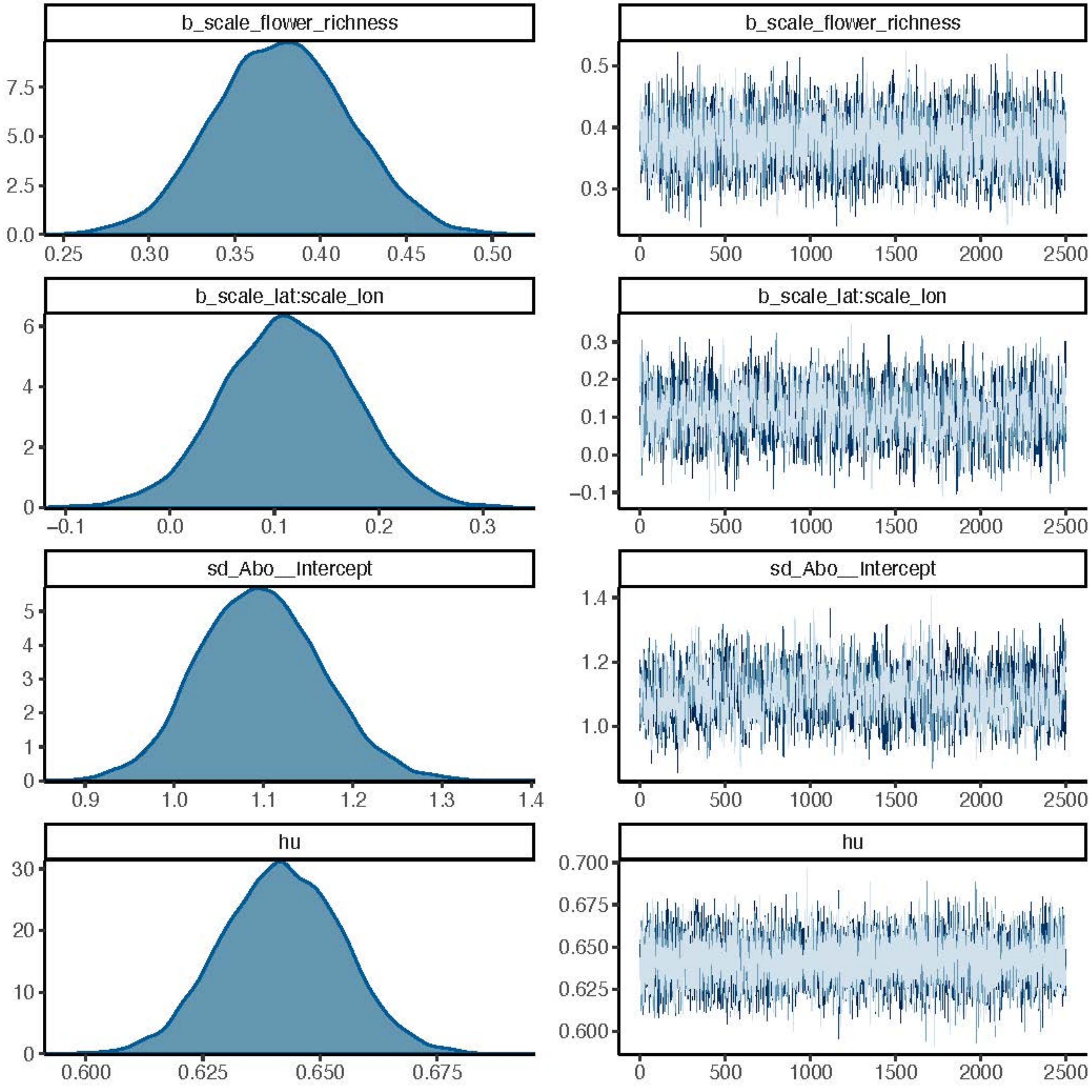

b_scale_flower_richness
b_scale_lat:scale_lon
sd_Abo__Intercept
hu
0.25
0.30
0.35
0.40
0.45
0.50
0
500
1000
1500
2000
2500

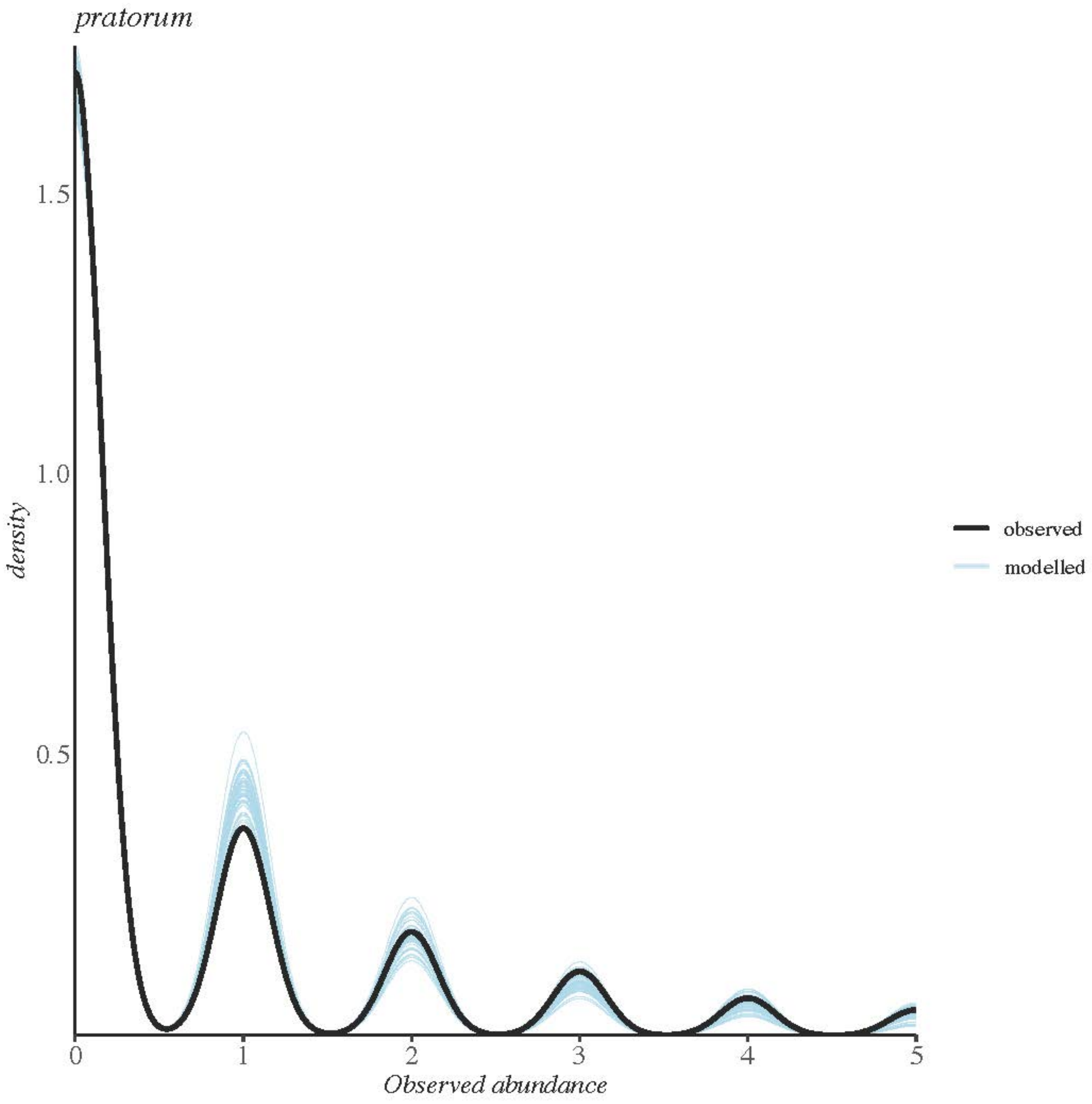
pratorum
density
1.5
1.0
0.5
0
1
2
3
4
5
Observed abundance
observed
modelled

lucorum

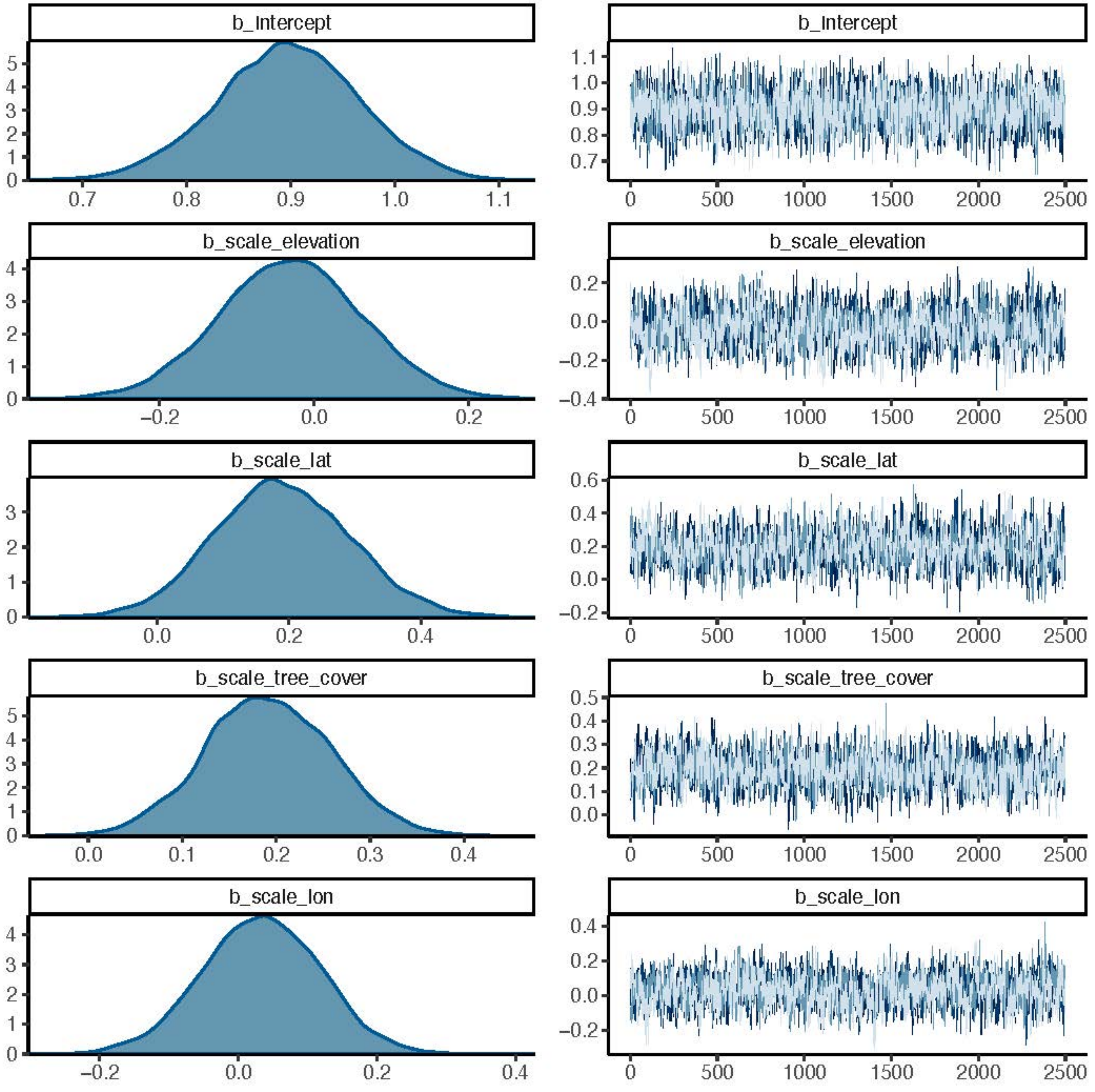

b_Intercept
b_scale_elevation
b_scale_lat
b_scale_tree_cover
b_scale_lon
0.7 0.8 0.9 1.0 1.1
−0.2 0.0 0.2
0.0 0.2 0.4
0.0 0.1 0.2 0.3 0.4
−0.2 0.0 0.2 0.4
0 500 1000 1500 2000 2500

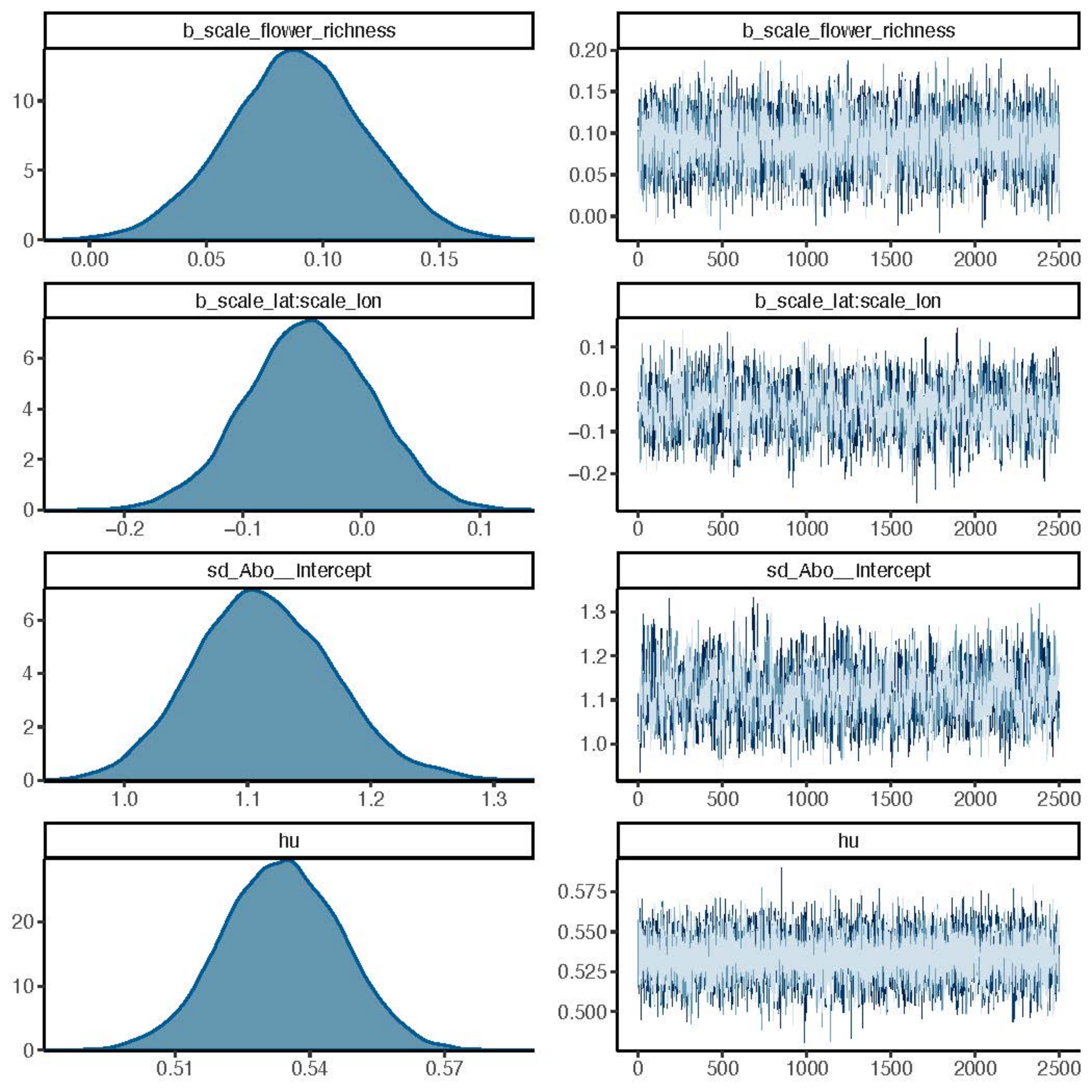

b_scale_flower_richness
b_scale_lat:scale_lon
sd_Abo__Intercept
hu

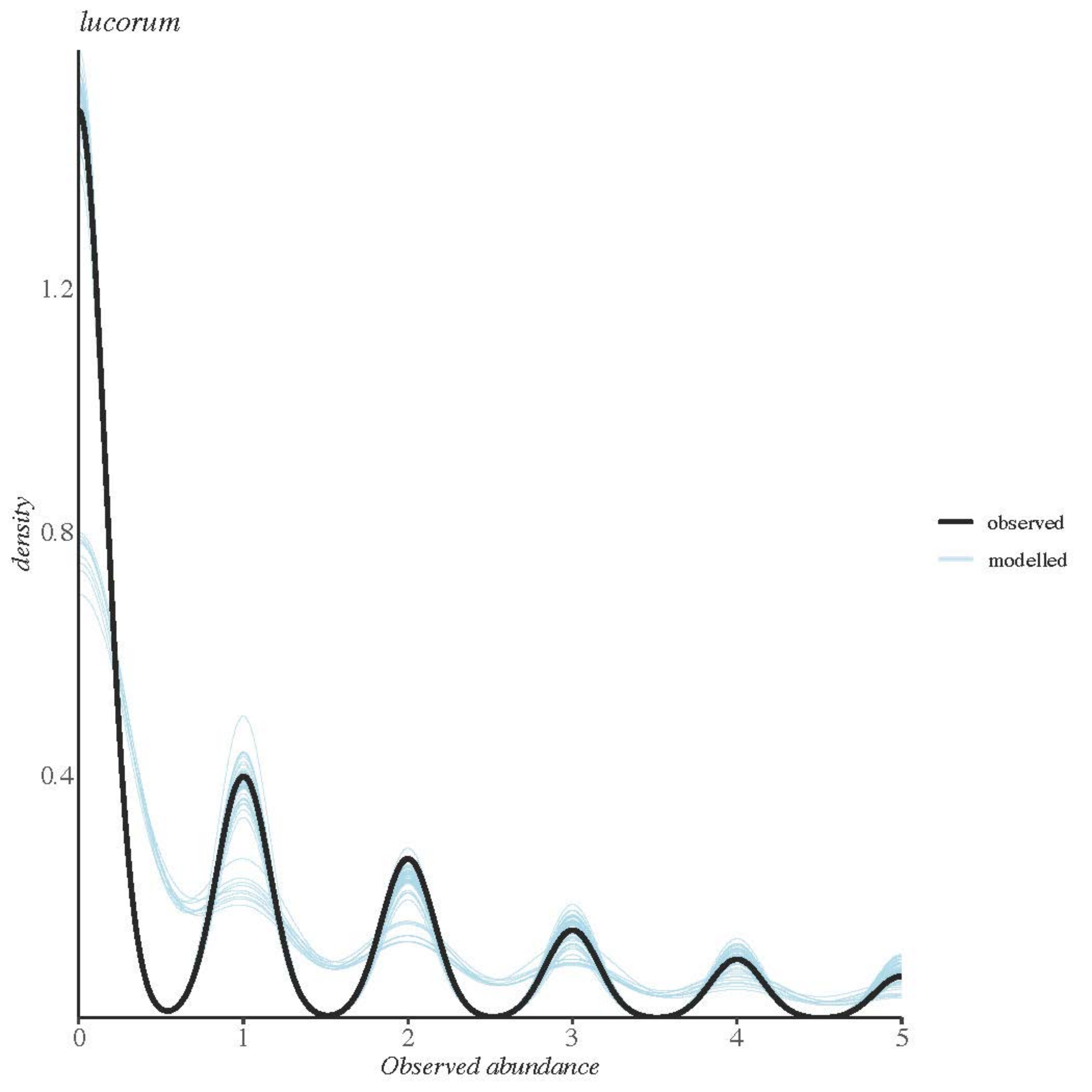

lucorum
density
1.2
0.8
0.4
0
1
2
3
4
5
Observed abundance
observed
modelled

pascuorum

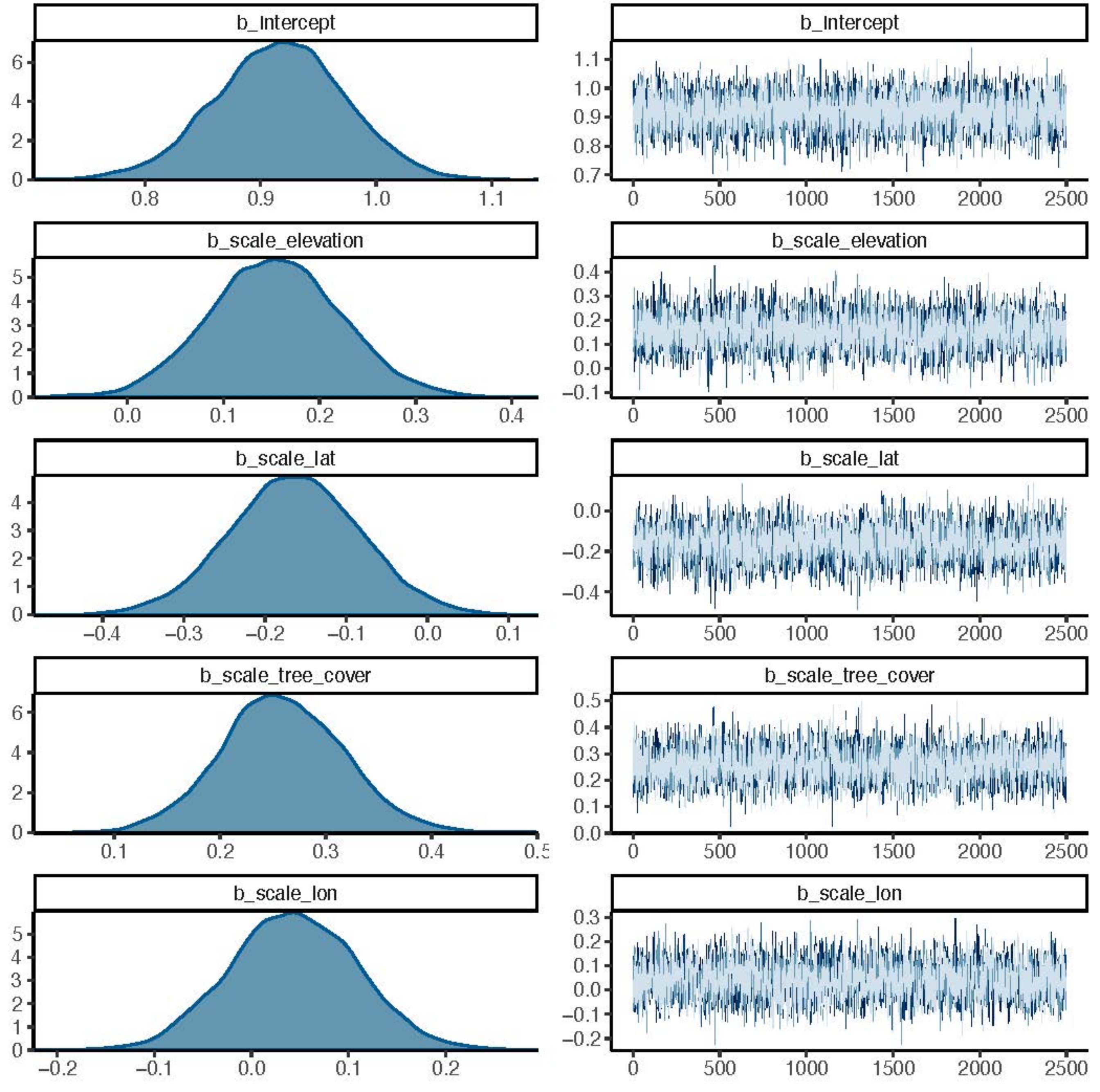
b_Intercept
0 2 4 6
0.8 0.9 1.0 1.1
b_Intercept
0.7 0.8 0.9 1.0 1.1
0 500 1000 1500 2000 2500
b_scale_elevation
0 1 2 3 4 5
0.0 0.1 0.2 0.3 0.4
b_scale_elevation
−0.1 0.0 0.1 0.2 0.3 0.4
0 500 1000 1500 2000 2500
b_scale_lat
0 1 2 3 4
−0.4 −0.3 −0.2 −0.1 0.0 0.1
b_scale_lat
−0.4 −0.2 0.0
0 500 1000 1500 2000 2500
b_scale_tree_cover
0 2 4 6
0.1 0.2 0.3 0.4
b_scale_tree_cover
0.0 0.1 0.2 0.3 0.4 0.5
0 500 1000 1500 2000 2500
b_scale_lon
0 1 2 3 4 5
−0.2 −0.1 0.0 0.1 0.2
b_scale_lon
−0.2 −0.1 0.0 0.1 0.2 0.3
0 500 1000 1500 2000 2500

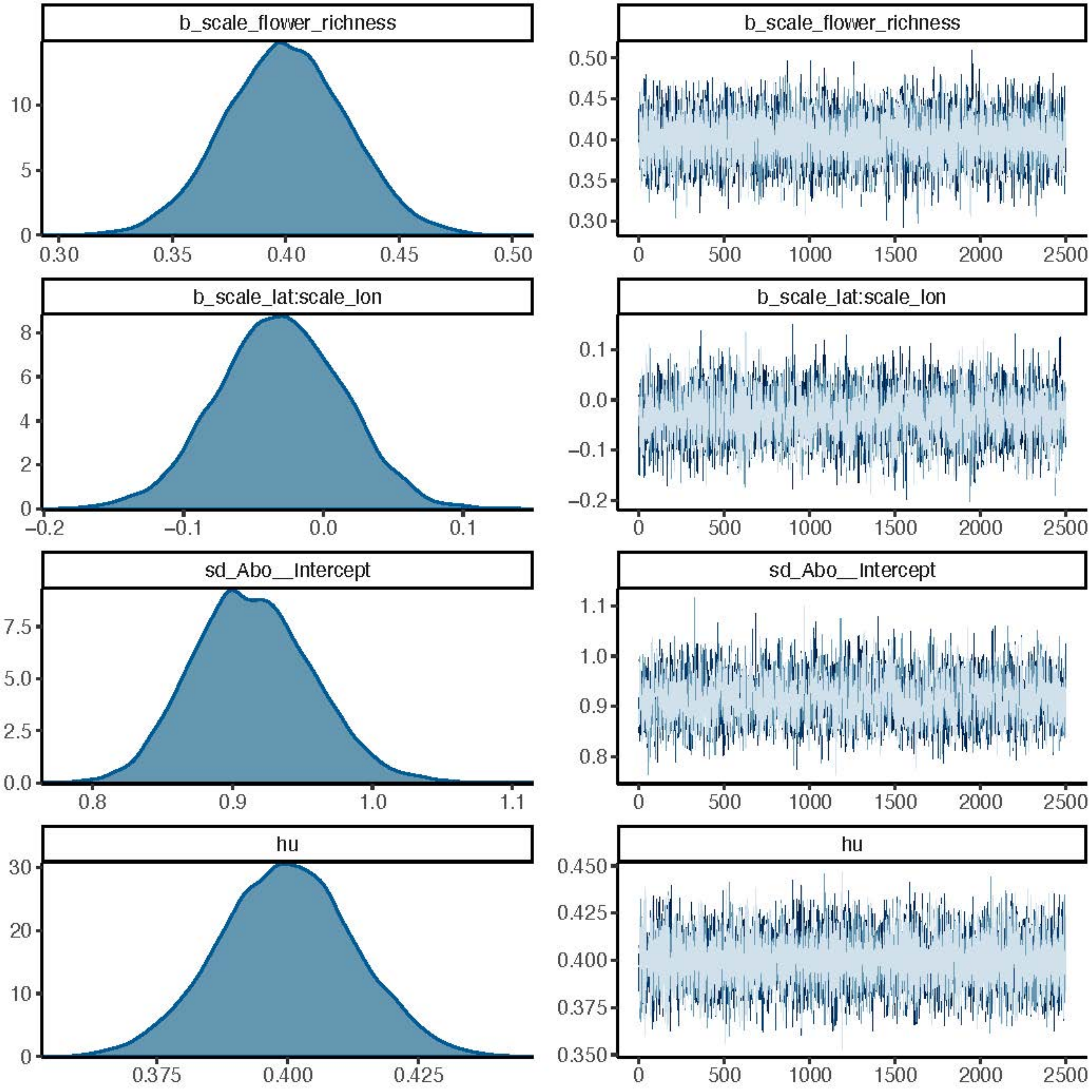

b_scale_flower_richness
b_scale_flower_richness
b_scale_lat:scale_lon
b_scale_lat:scale_lon
sd_Abo__Intercept
sd_Abo__Intercept
hu
hu

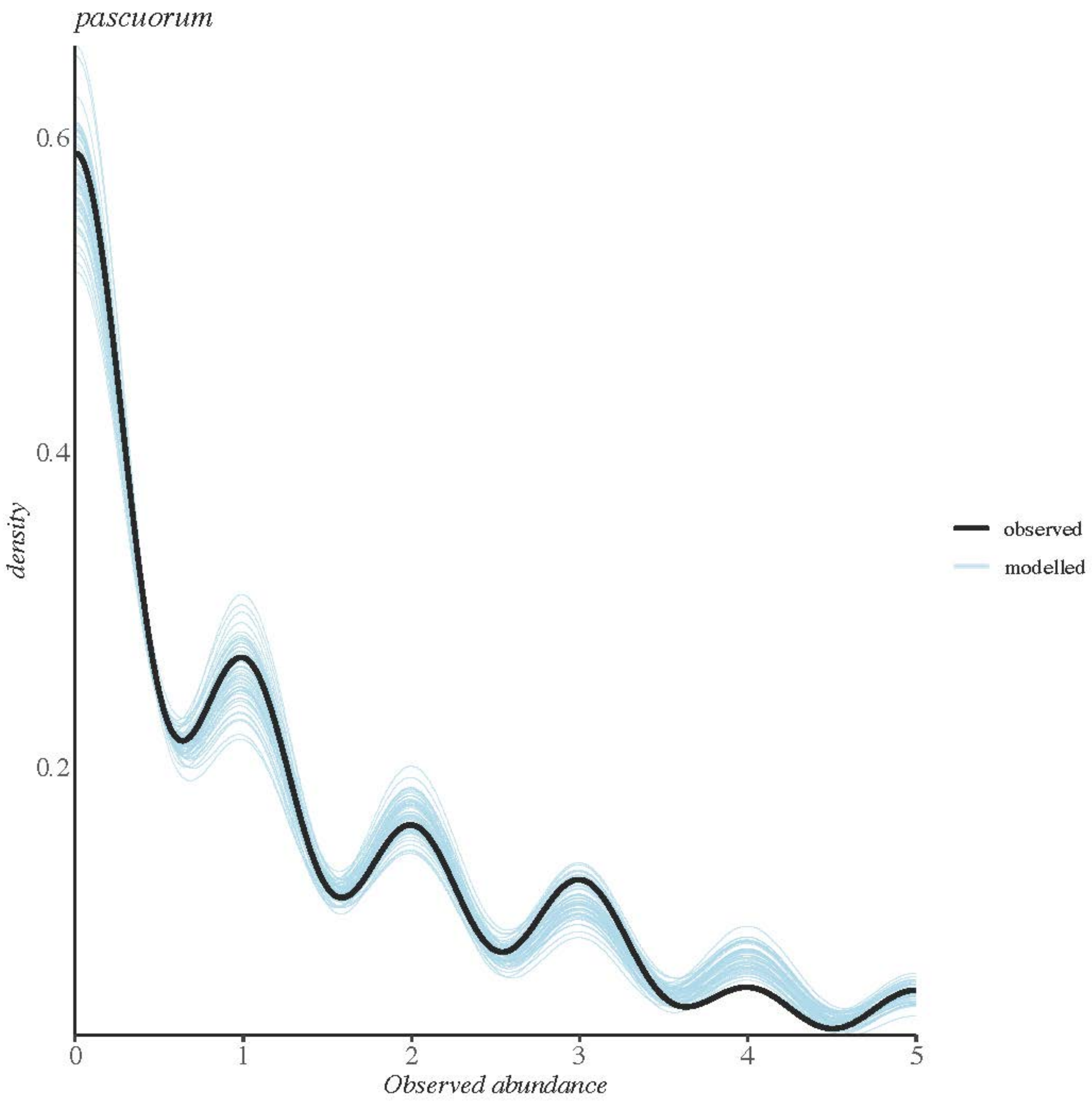

pascuorum
0.6
0.4
0.2
density
0
1
2
3
4
5
Observed abundance
observed
modelled

Effects of tree cover on the community weighted means of visual traits relative to body size in bumblebee communities within grasslands across Sweden. Model traces and posterior predictive check for each visual trait modelled with Bayesian inference.

Eye surface (μm^2)

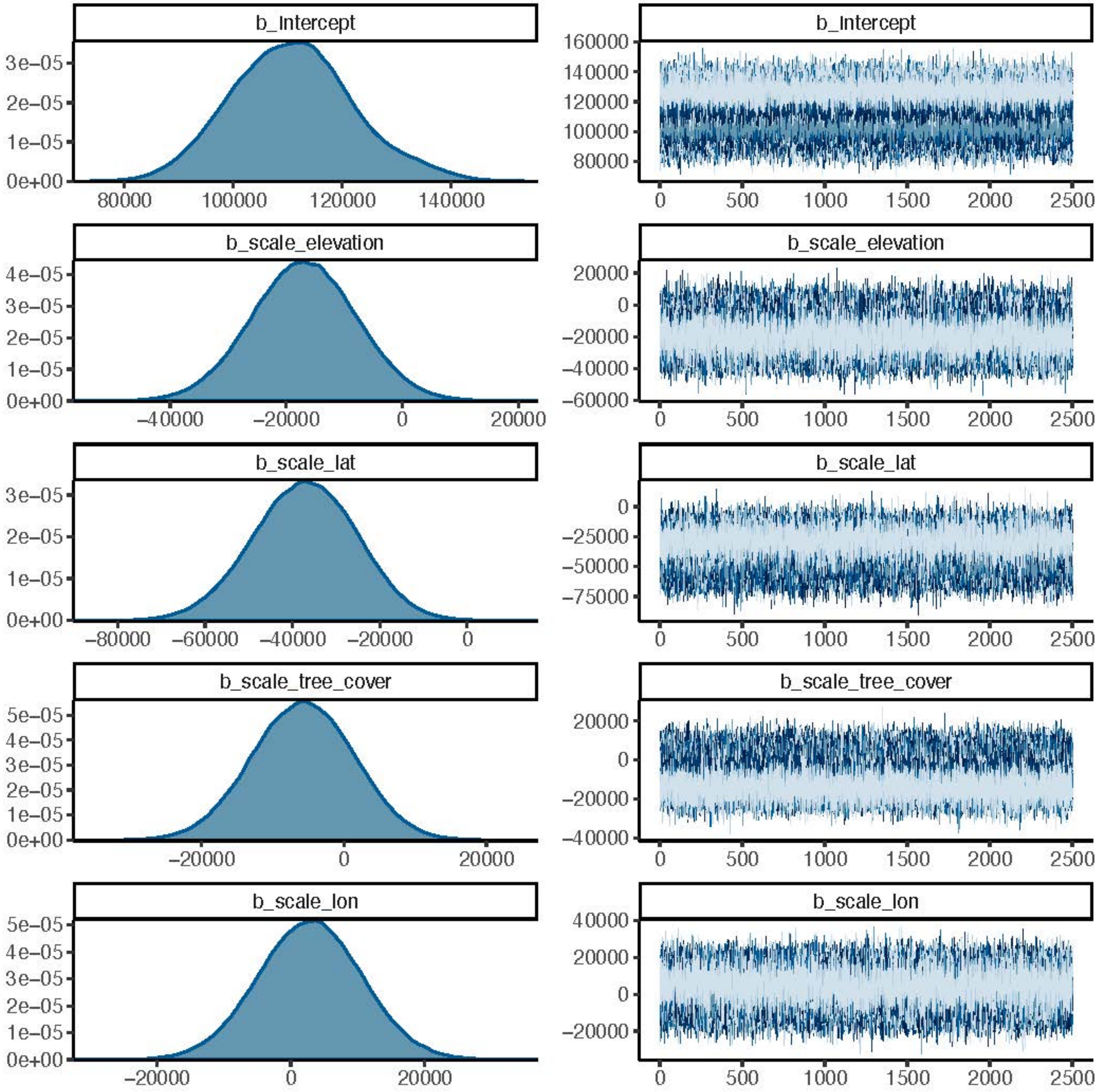

b_Intercept
b_scale_elevation
b_scale_lat
b_scale_tree_cover
b_scale_lon

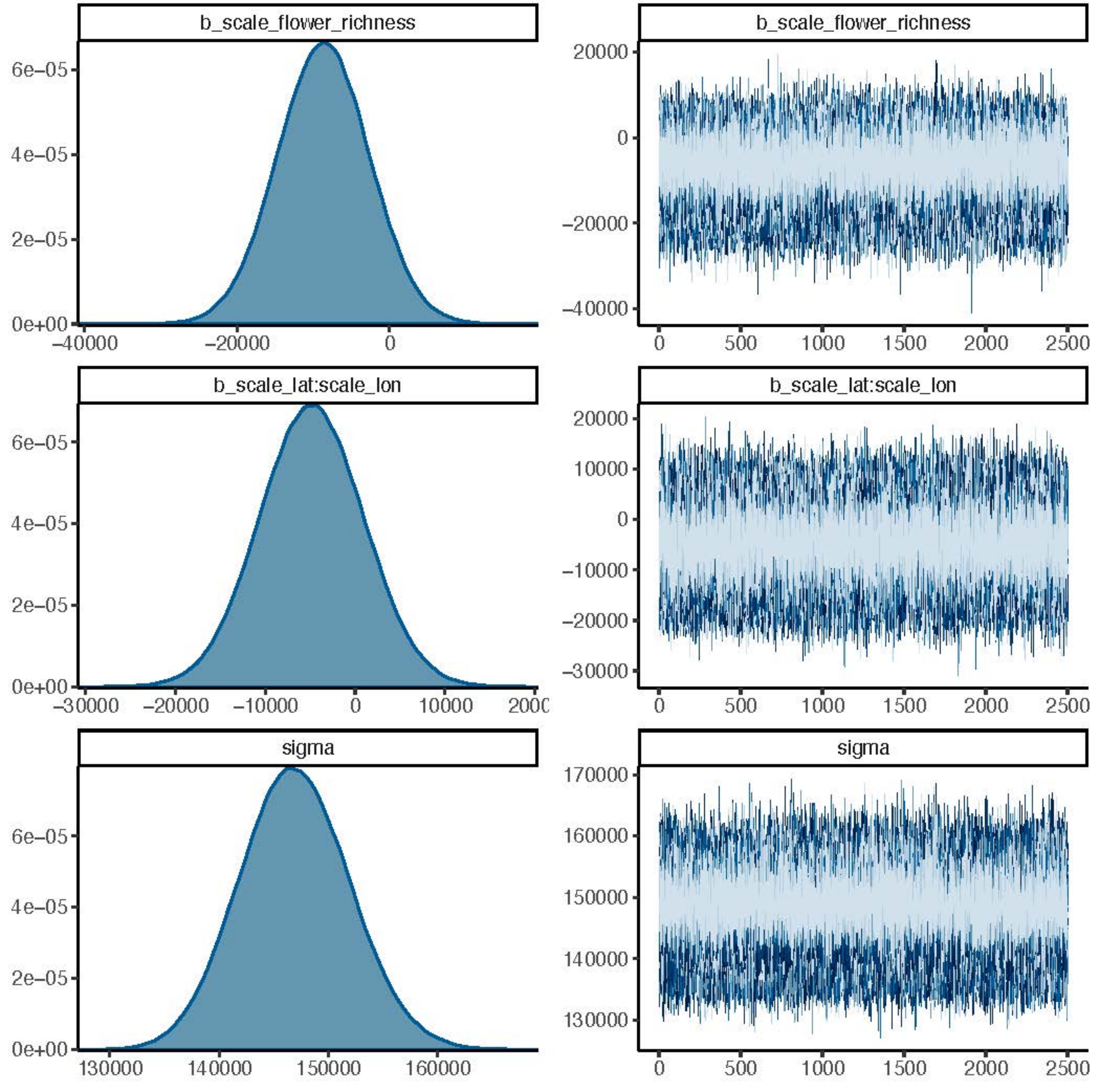
b_scale_flower_richness
b_scale_flower_richness
b_scale_lat:scale_lon
b_scale_lat:scale_lon
sigma
sigma

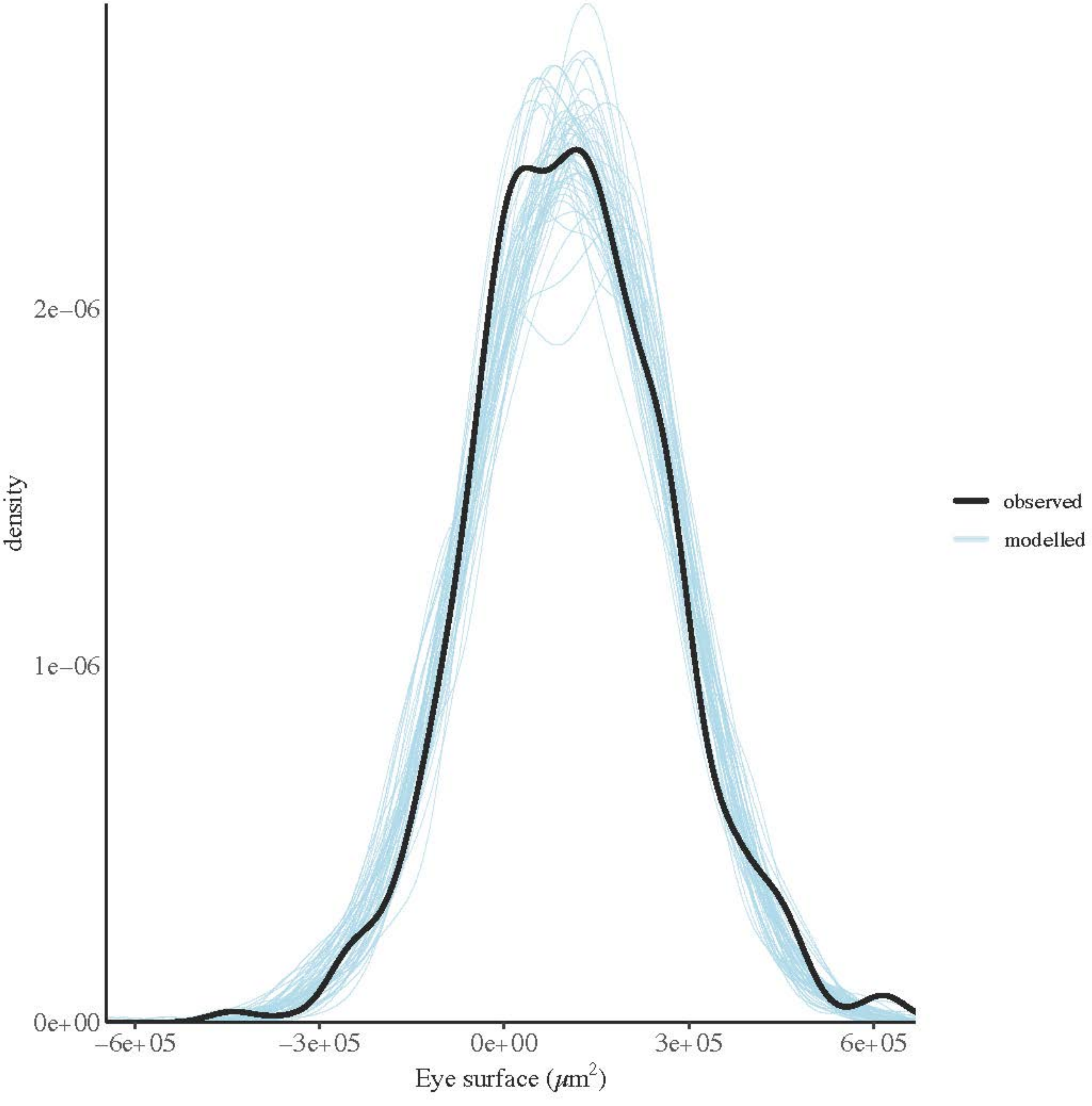
density
2e−06
1e−06
0e+00
−6e+05
−3e+05
0e+00
3e+05
6e+05
Eye surface (μm2)
observed
modelled

Facet diameter (μm)

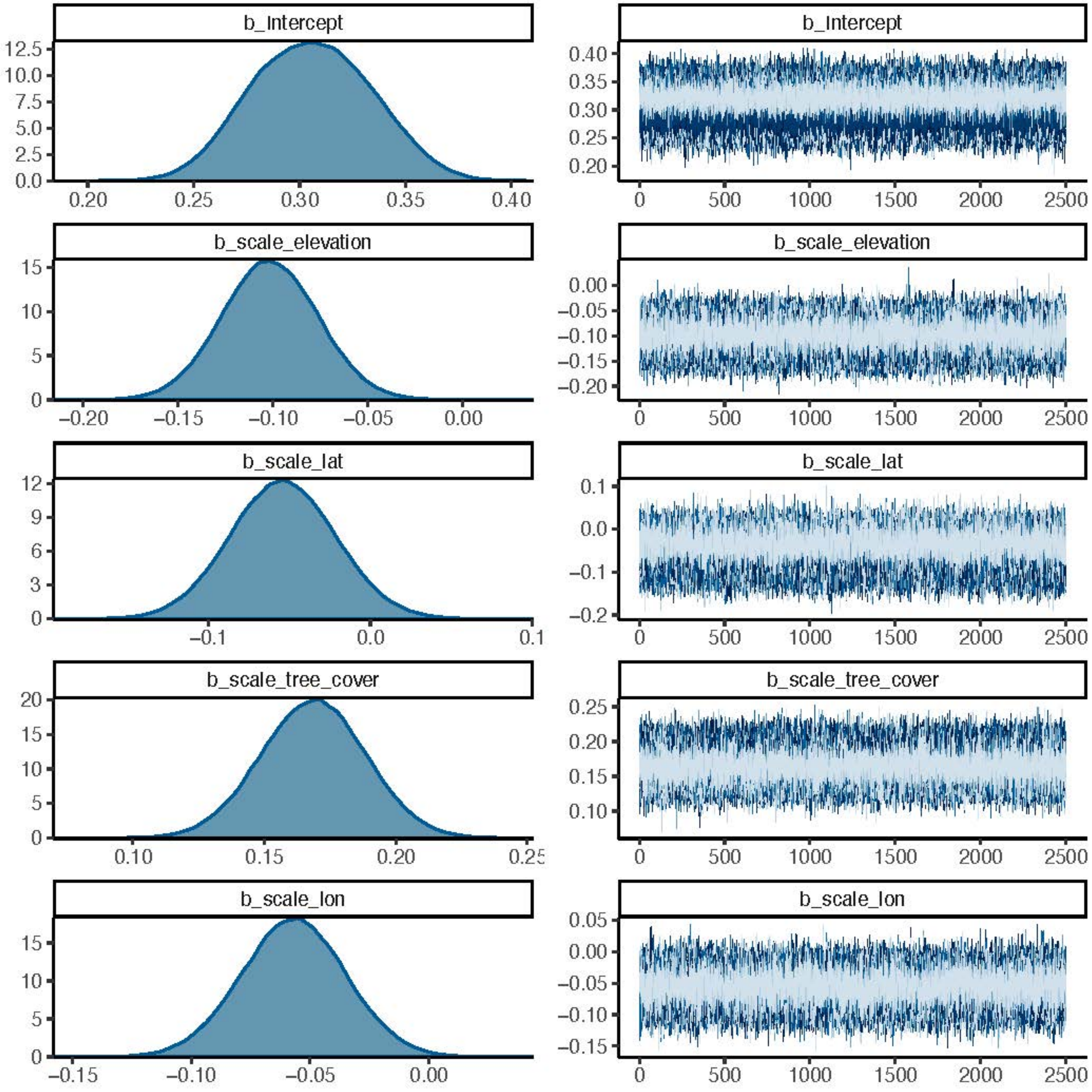

b_Intercept
b_scale_elevation
b_scale_lat
b_scale_tree_cover
b_scale_lon

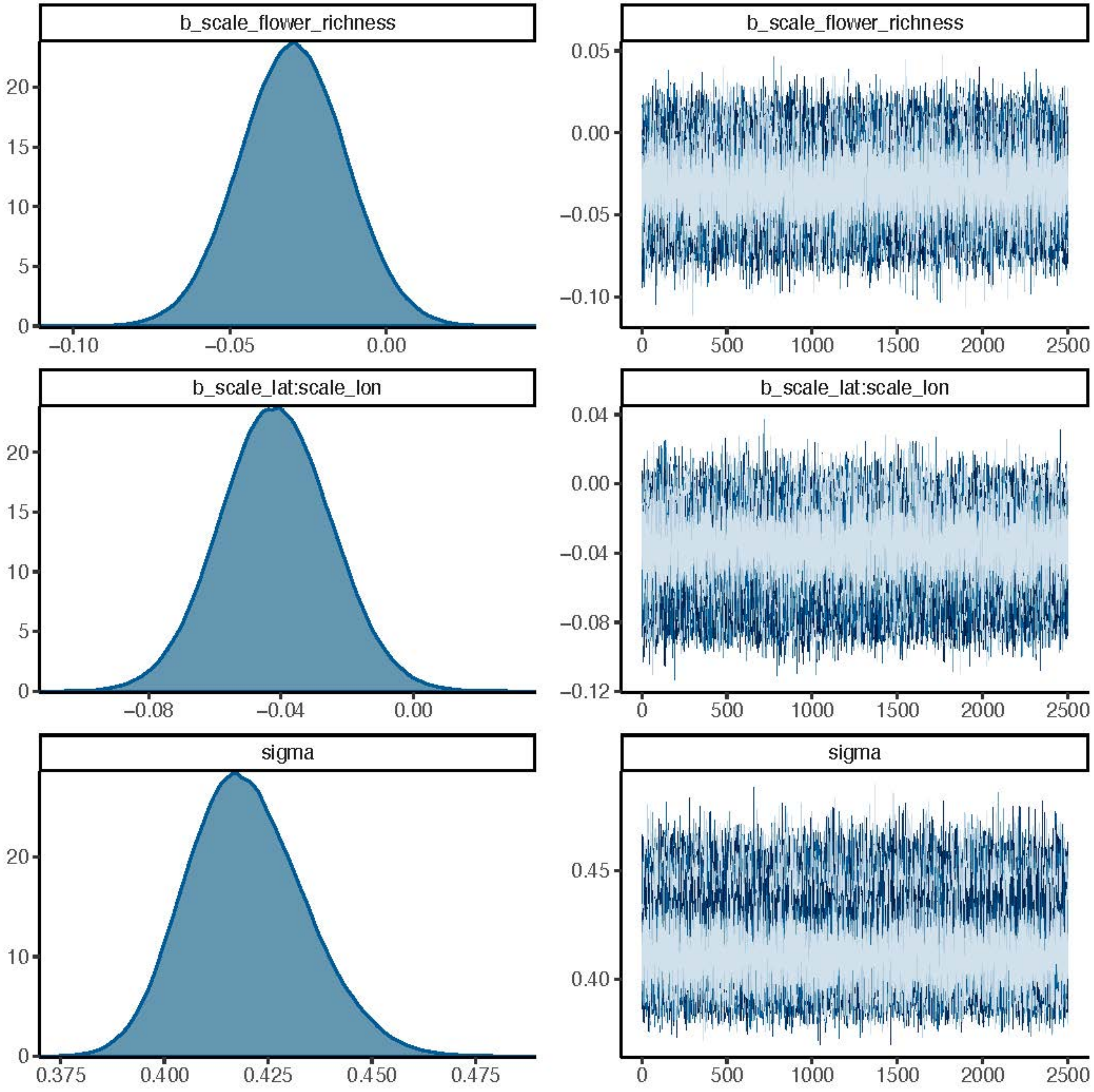

b_scale_flower_richness
b_scale_flower_richness
b_scale_lat:scale_lon
b_scale_lat:scale_lon
sigma
sigma
0
5
10
15
20
−0.10
−0.05
0.00
0.05
−0.10
0
500
1000
1500
2000
2500
−0.08
−0.04
0.00
0.04
−0.12
0.375
0.400
0.425
0.450
0.475
0.45
0.40

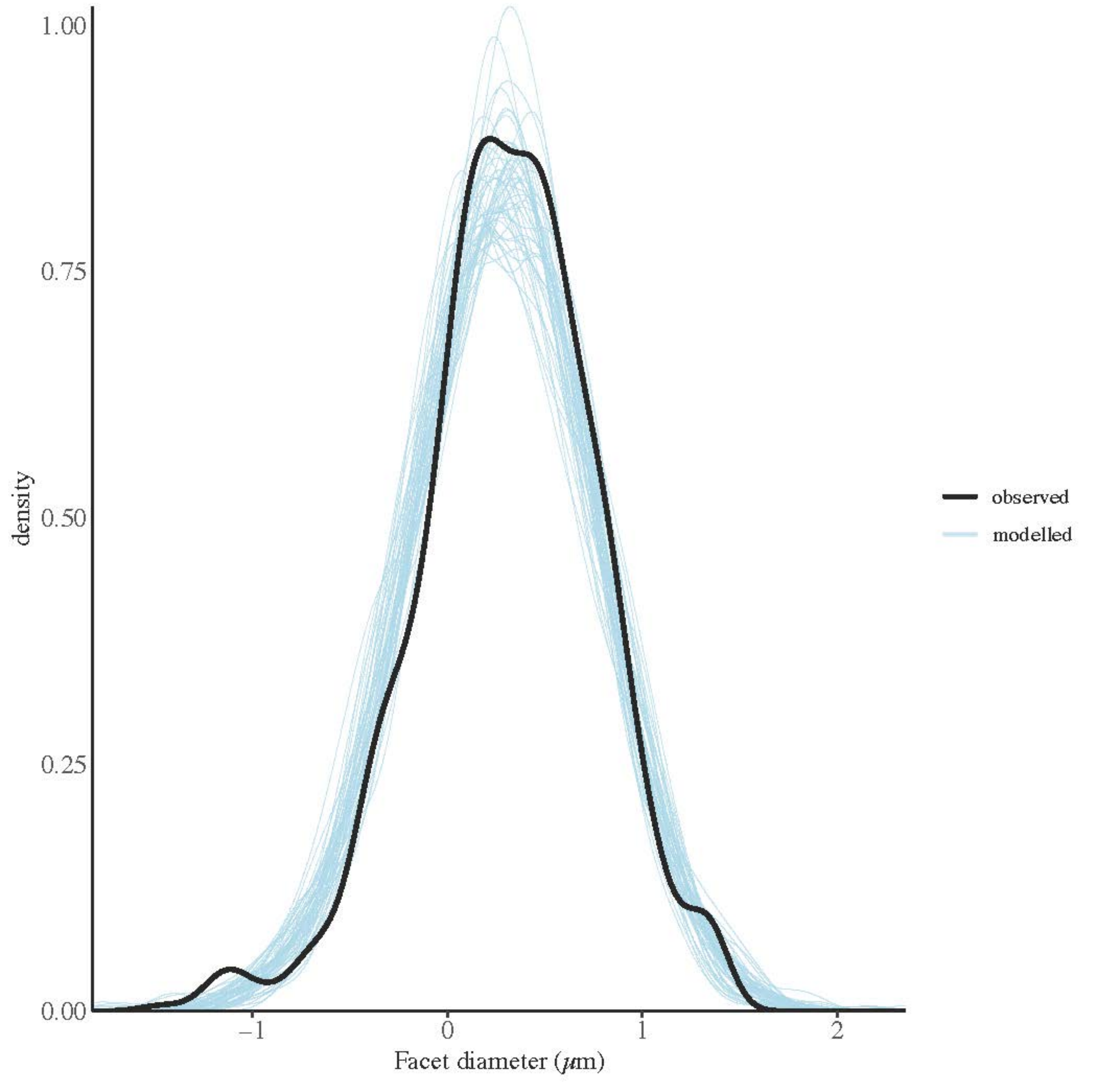
1.00
0.75
0.50
0.25
0.00
density
−1
0
1
2
Facet diameter (μm)
observed
modelled

Inter–ommatidial angle (°)

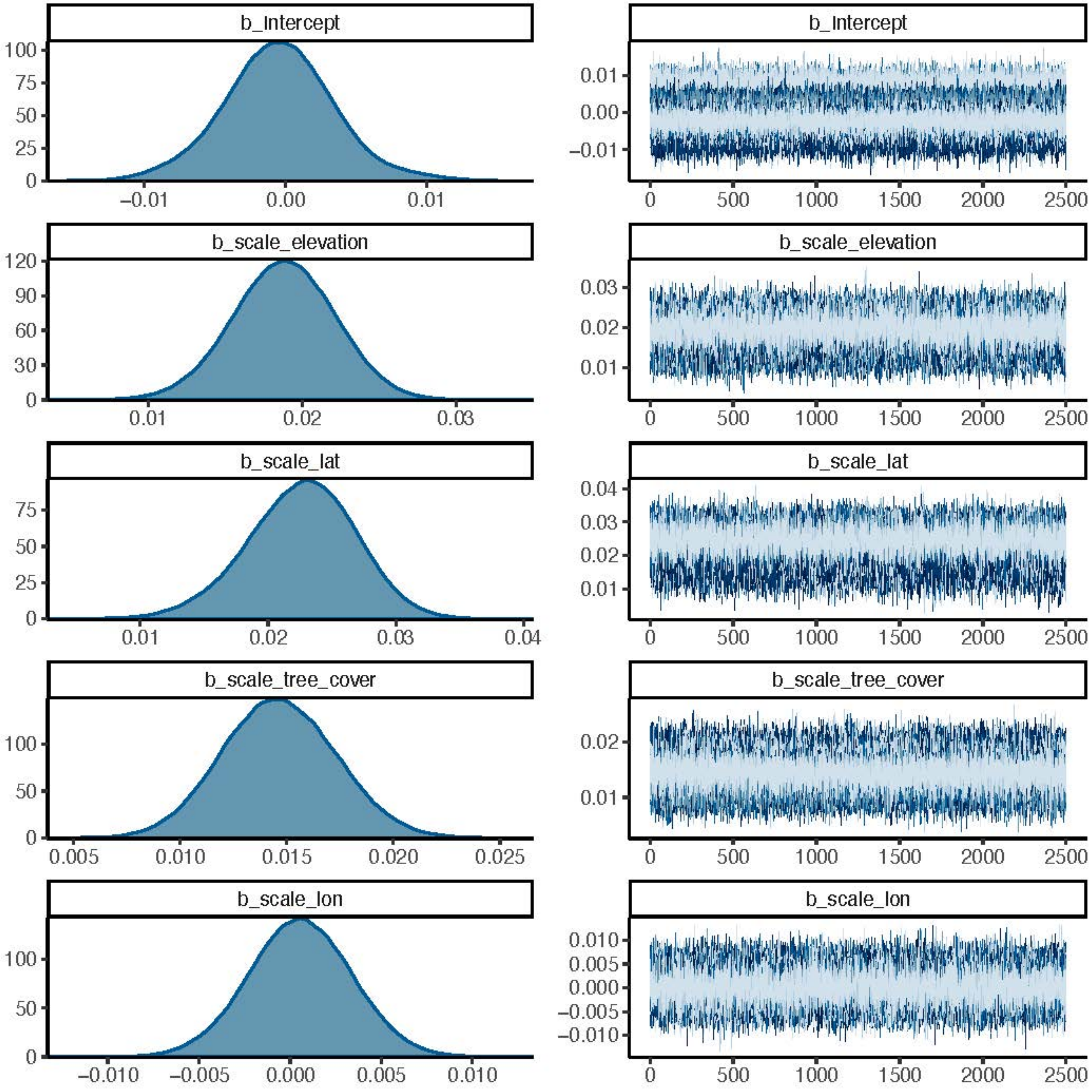

b_Intercept
100
75
50
25
0
−0.01
0.00
0.01
b_Intercept
0.01
0.00
−0.01
0
500
1000
1500
2000
2500
b_scale_elevation
120
90
60
30
0
0.01
0.02
0.03
b_scale_elevation
0.03
0.02
0.01
b_scale_lat
75
50
25
0
0.01
0.02
0.03
0.04
b_scale_lat
0.04
0.03
0.02
0.01
b_scale_tree_cover
100
50
0
0.005
0.010
0.015
0.020
0.025
b_scale_tree_cover
0.02
0.01
b_scale_lon
100
50
0
−0.010
−0.005
0.000
0.005
0.010
b_scale_lon
0.010
0.005
0.000
−0.005
−0.010

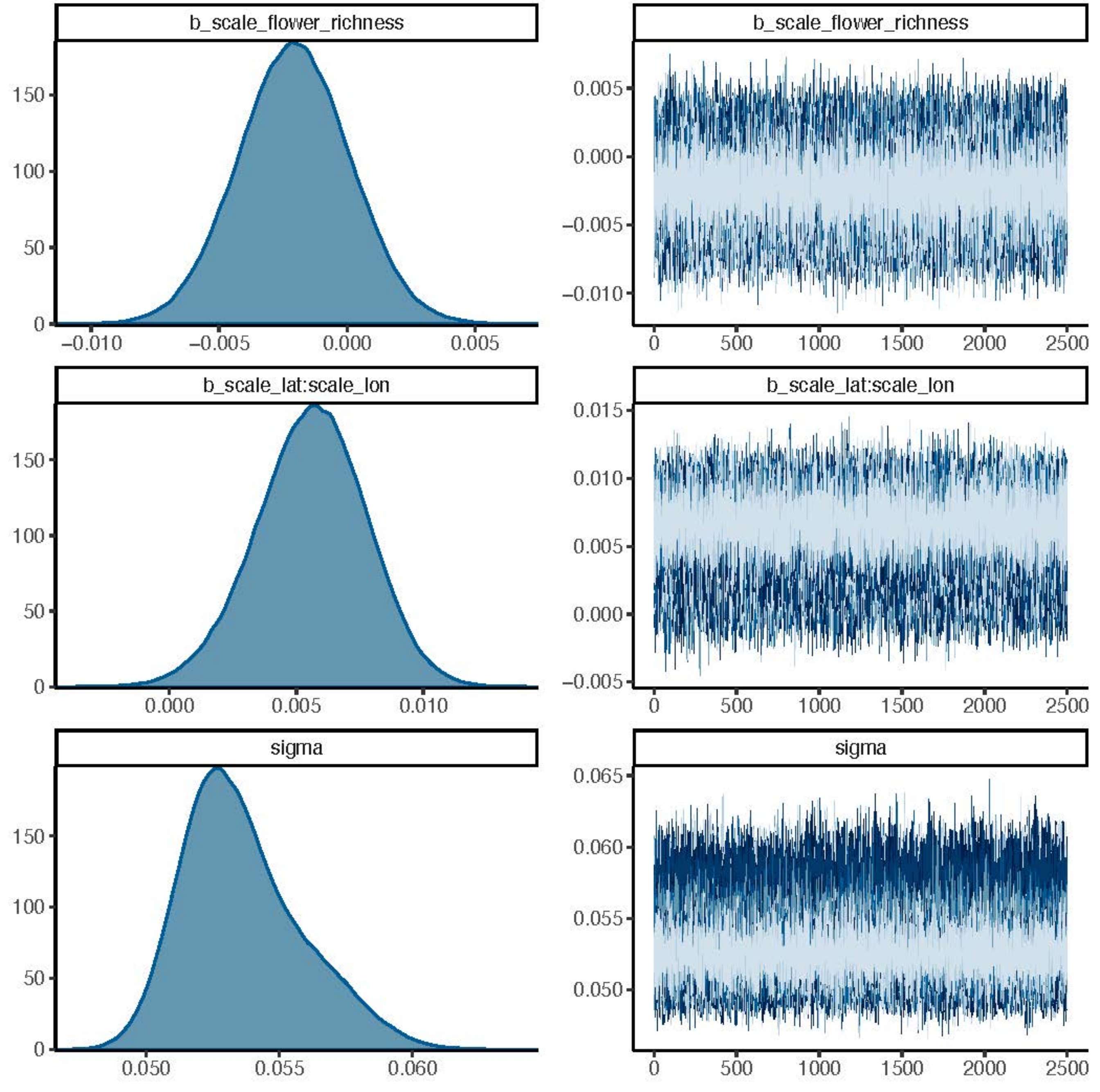

b_scale_flower_richness
150
100
50
0
−0.010
−0.005
0.000
0.005
b_scale_flower_richness
0.005
0.000
−0.005
−0.010
0
500
1000
1500
2000
2500
b_scale_lat:scale_lon
150
100
50
0
0.000
0.005
0.010
b_scale_lat:scale_lon
0.015
0.010
0.005
0.000
−0.005
0
500
1000
1500
2000
2500
sigma
150
100
50
0
0.050
0.055
0.060
sigma
0.065
0.060
0.055
0.050
0
500
1000
1500
2000
2500

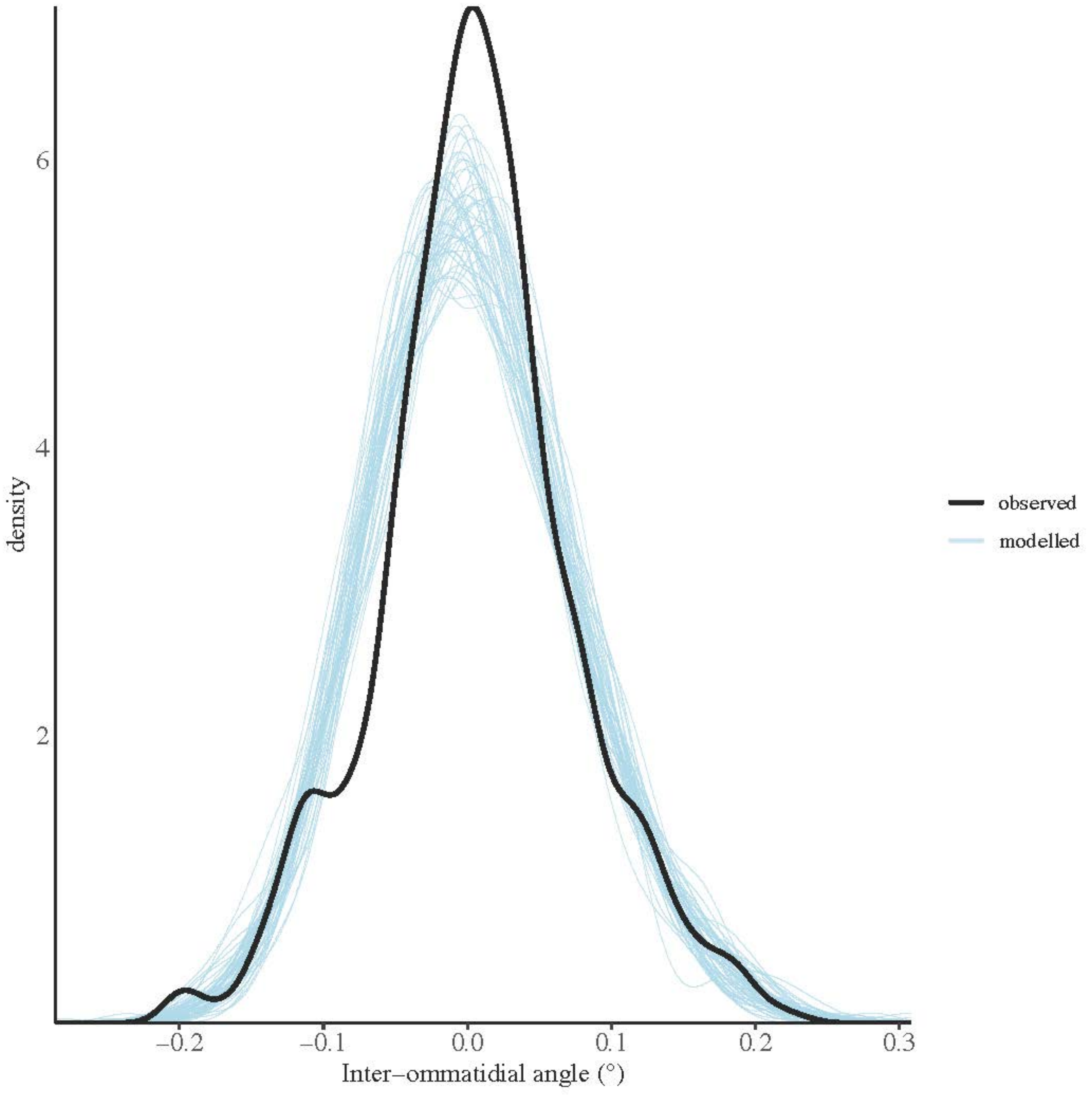
6
4
2
density
−0.2
−0.1
0.0
0.1
0.2
0.3
Inter−ommatidial angle (°)
observed
modelled

Facet number

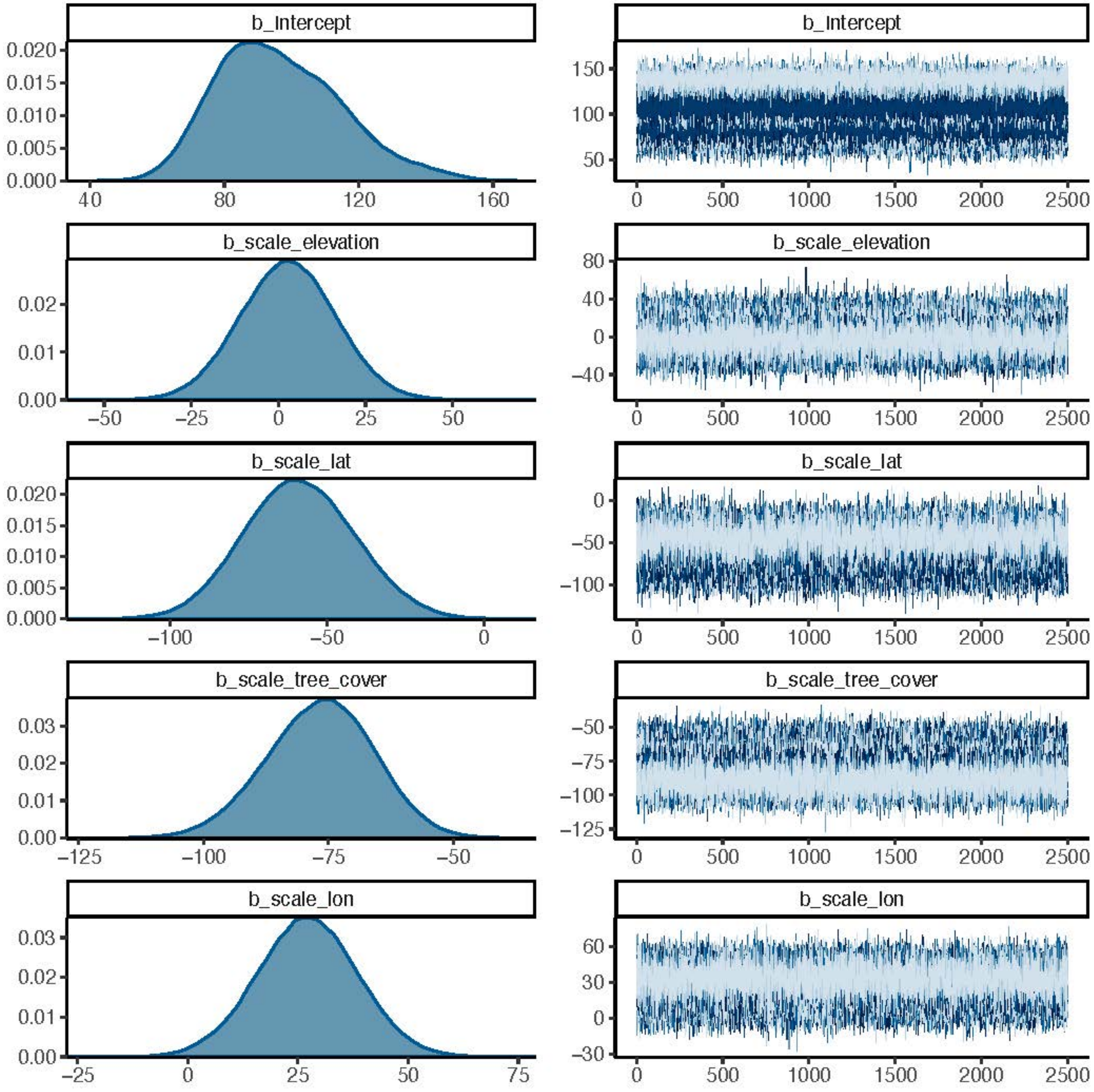

b_Intercept
b_scale_elevation
b_scale_lat
b_scale_tree_cover
b_scale_lon

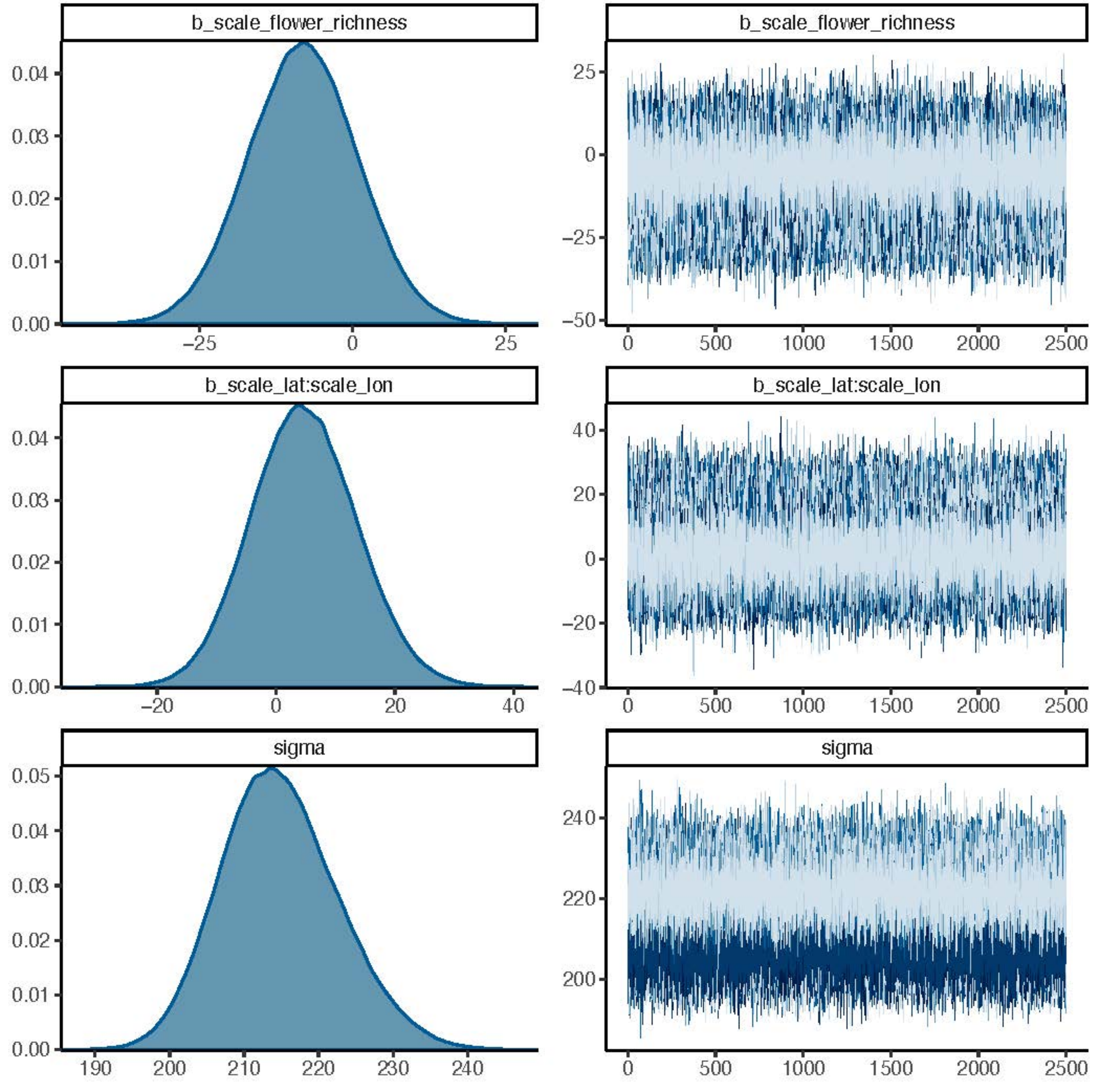
b_scale_flower_richness
0.04
0.03
0.02
0.01
0.00
−25
0
25
b_scale_flower_richness
25
0
−25
−50
0
500
1000
1500
2000
2500
b_scale_lat:scale_lon
0.04
0.03
0.02
0.01
0.00
−20
0
20
40
b_scale_lat:scale_lon
40
20
0
−20
−40
0
500
1000
1500
2000
2500
sigma
0.05
0.04
0.03
0.02
0.01
0.00
190
200
210
220
230
240
sigma
240
220
200
0
500
1000
1500
2000
2500

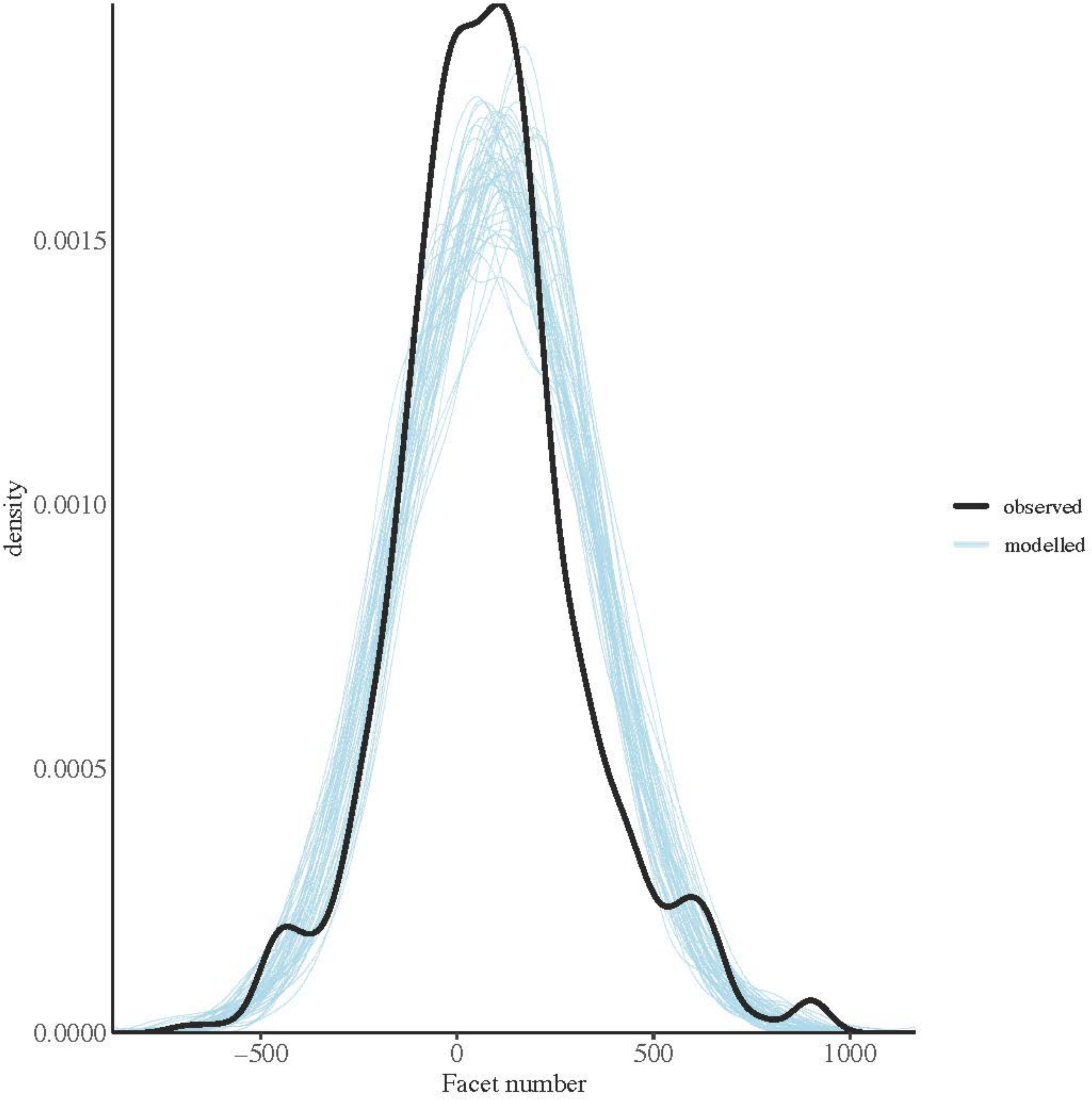
density
0.0000
0.0005
0.0010
0.0015
−500
0
500
1000
Facet number
observed
modelled

Eye parameter (μm.rad)

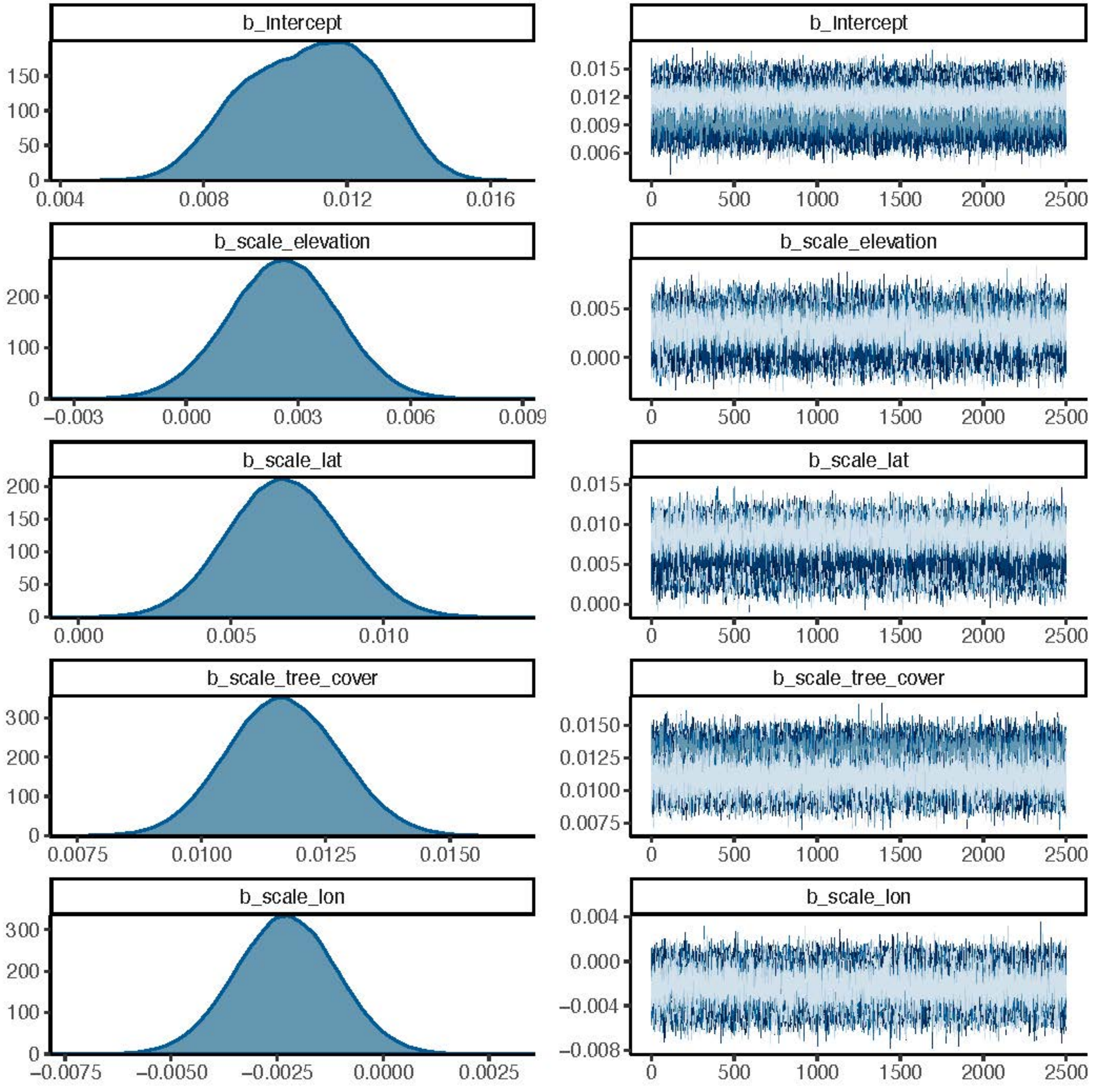
b_Intercept
b_scale_elevation
b_scale_lat
b_scale_tree_cover
b_scale_lon
0.004
0.008
0.012
0.016
0
50
100
150
200
250
300
0.015
0.009
0.006
500
1000
1500
2000
2500
−0.003
0.000
0.003
0.006
0.009
0.005
0.010
0.0075
0.0100
0.0125
0.0150
−0.0075
−0.0050
−0.0025
0.0000
0.0025
0.004
−0.004
−0.008

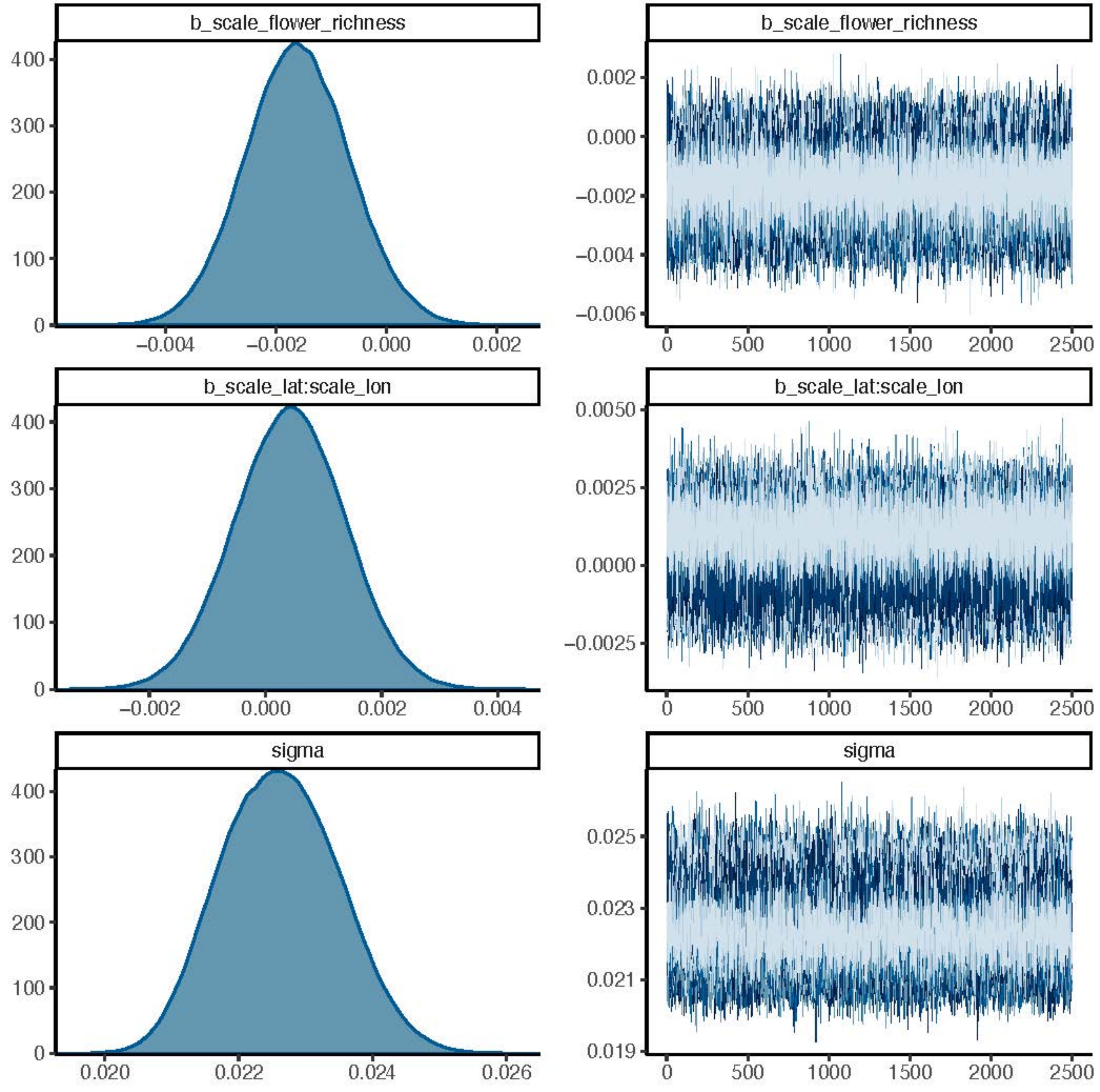
b_scale_flower_richness
b_scale_flower_richness
b_scale_lat:scale_lon
b_scale_lat:scale_lon
sigma
sigma

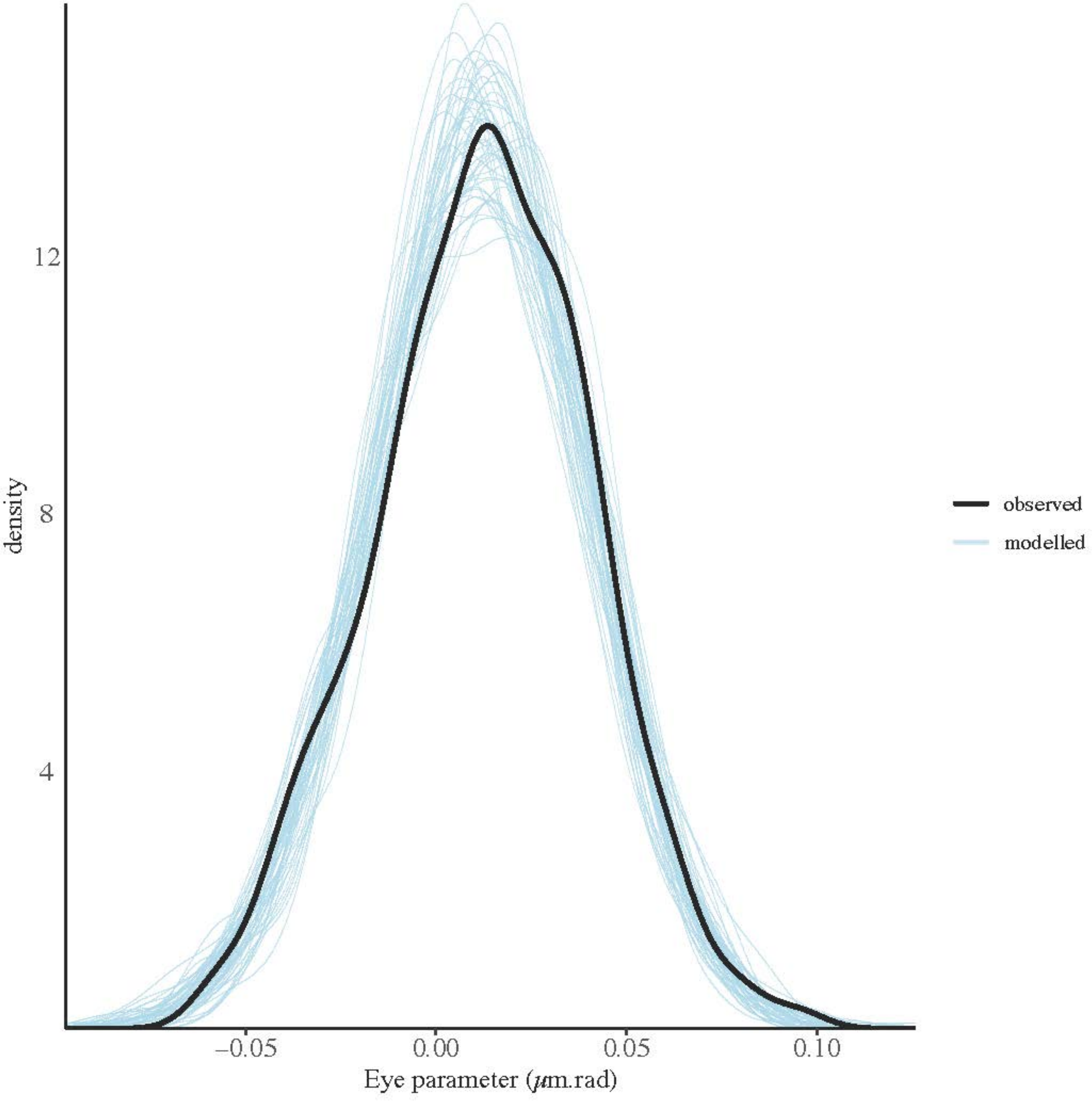

12
8
4
density
−0.05
0.00
0.05
0.10
Eye parameter (μm.rad)
observed
modelled

Curvature (μm)

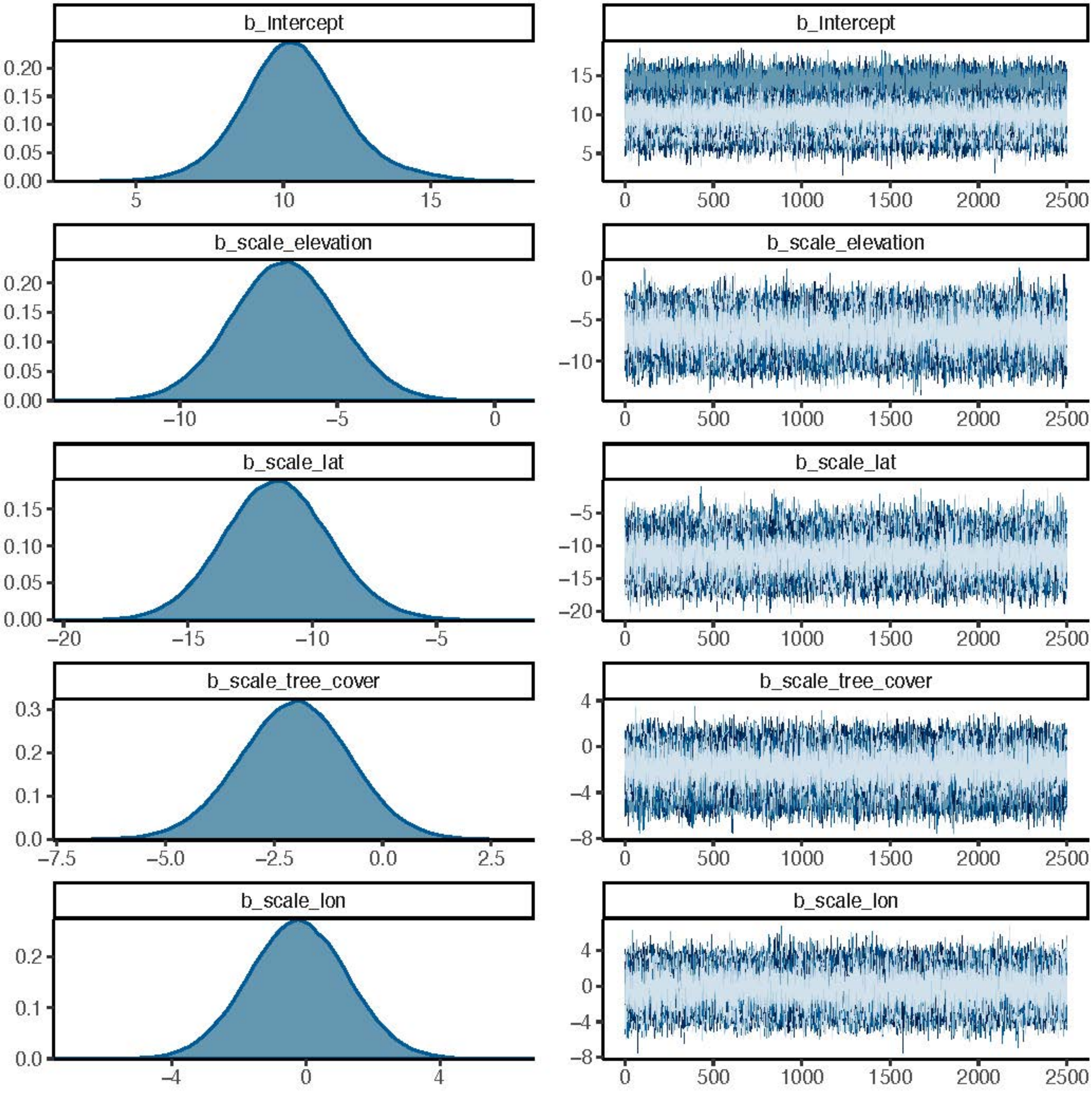
b_Intercept
b_Intercept
b_scale_elevation
b_scale_elevation
b_scale_lat
b_scale_lat
b_scale_tree_cover
b_scale_tree_cover
b_scale_lon
b_scale_lon
0.00
0.05
0.10
0.15
0.20
0.0
0.1
0.2
0.3
5
10
15
−20
−15
−10
−5
0
−7.5
−5.0
−2.5
0.0
2.5
−8
−4
4
500
1000
1500
2000
2500

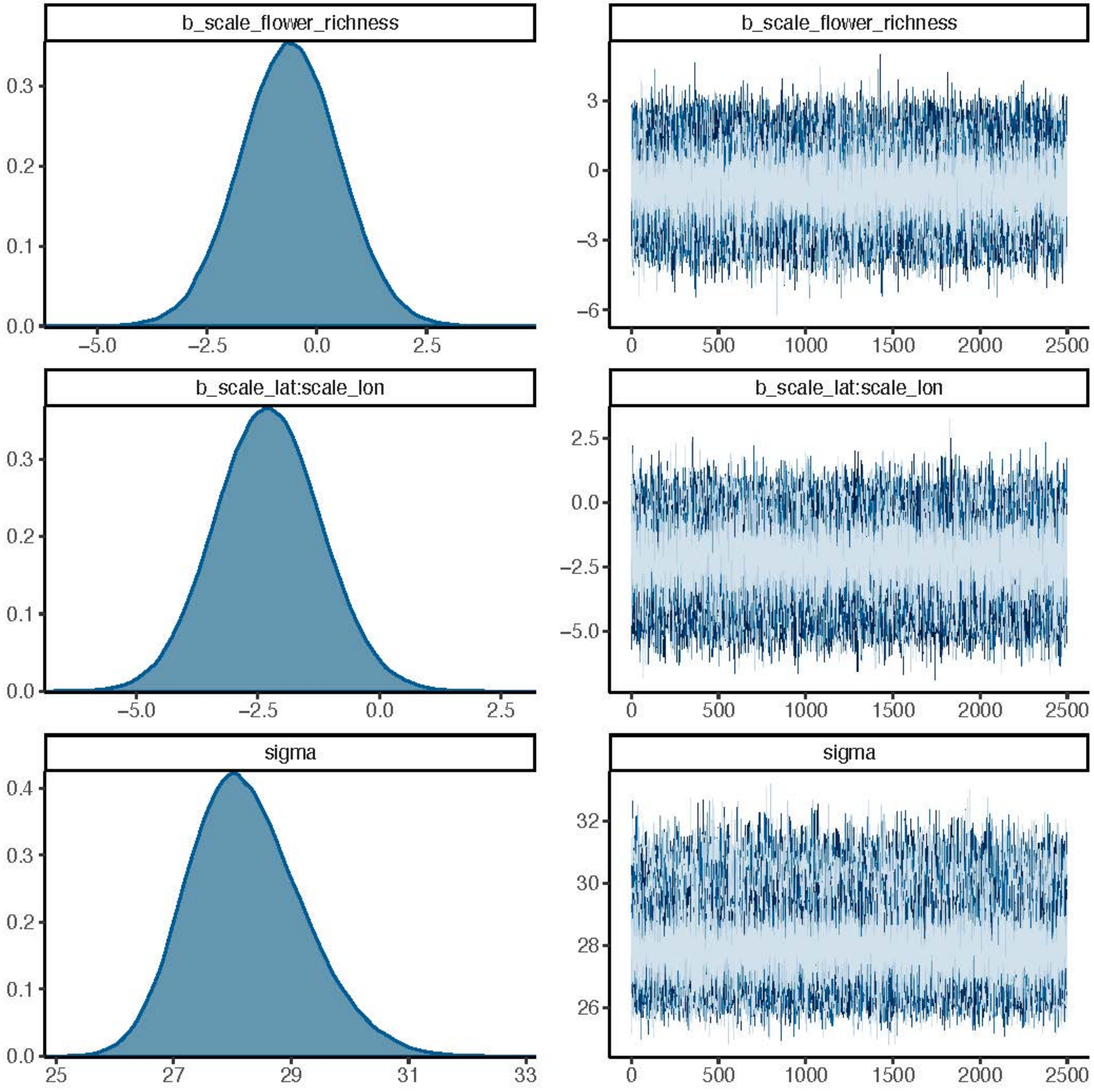
b_scale_flower_richness
b_scale_flower_richness
b_scale_lat:scale_lon
b_scale_lat:scale_lon
sigma
sigma
0.0
0.1
0.2
0.3
0.4
−5.0
−2.5
0.0
2.5
25
27
29
31
33
3
0
−3
−6
−5.0
32
30
28
26
500
1000
1500
2000
2500

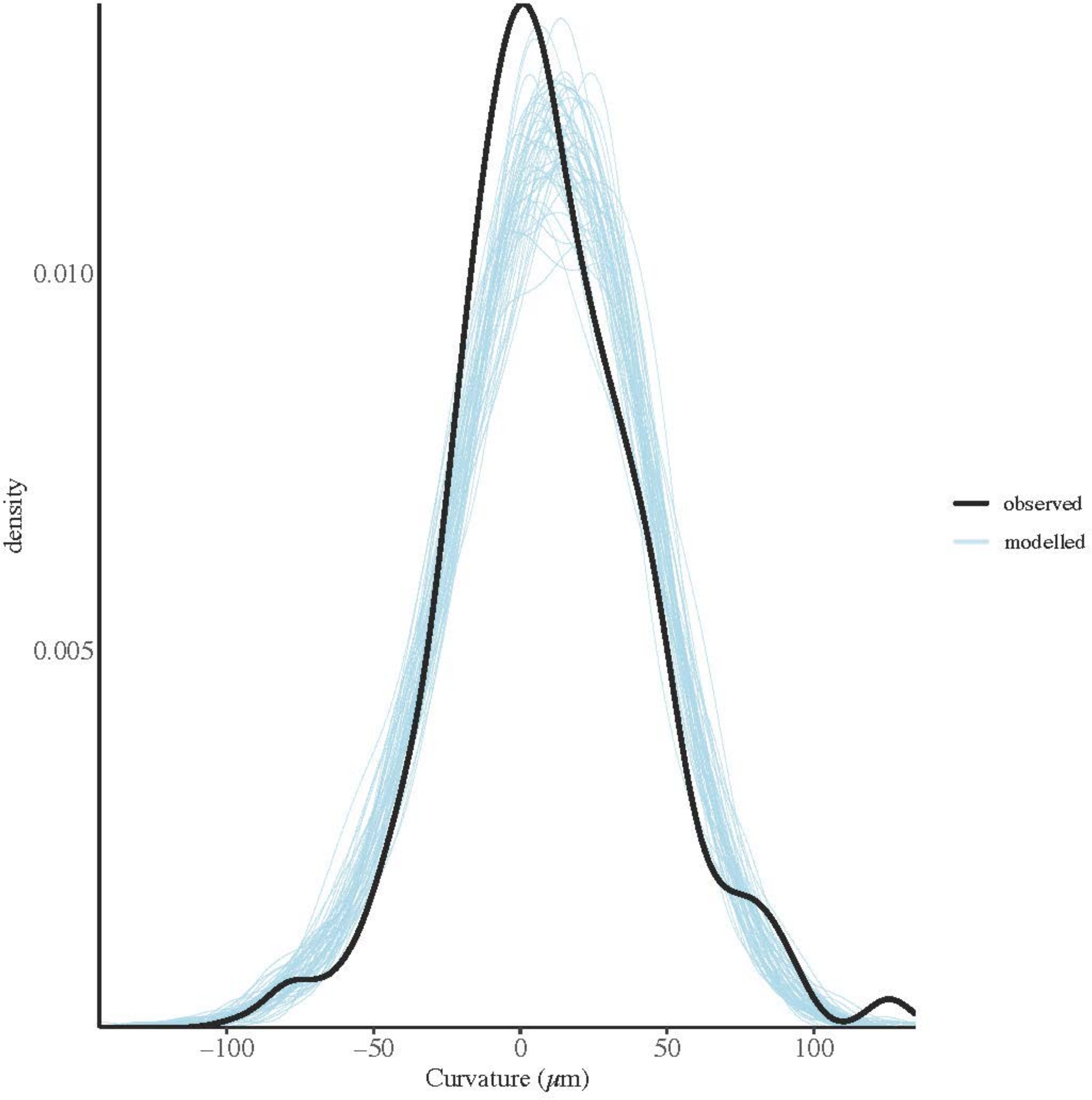

density
0.010
0.005
−100
−50
0
50
100
Curvature (μm)
observed
modelled

Central ocellus diameter (μm)

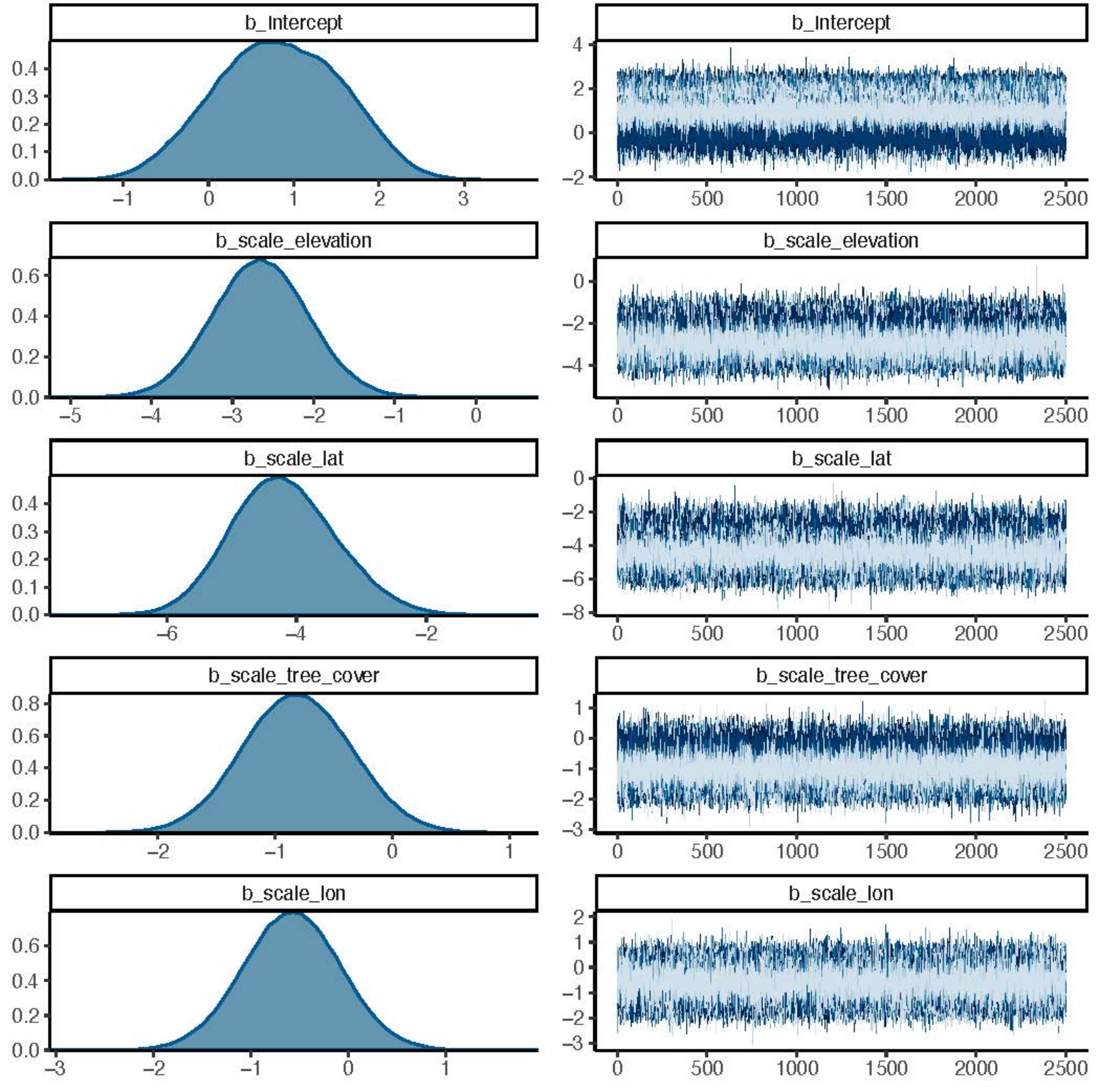
b_Intercept
b_Intercept
b_scale_elevation
b_scale_elevation
b_scale_lat
b_scale_lat
b_scale_tree_cover
b_scale_tree_cover
b_scale_lon
b_scale_lon

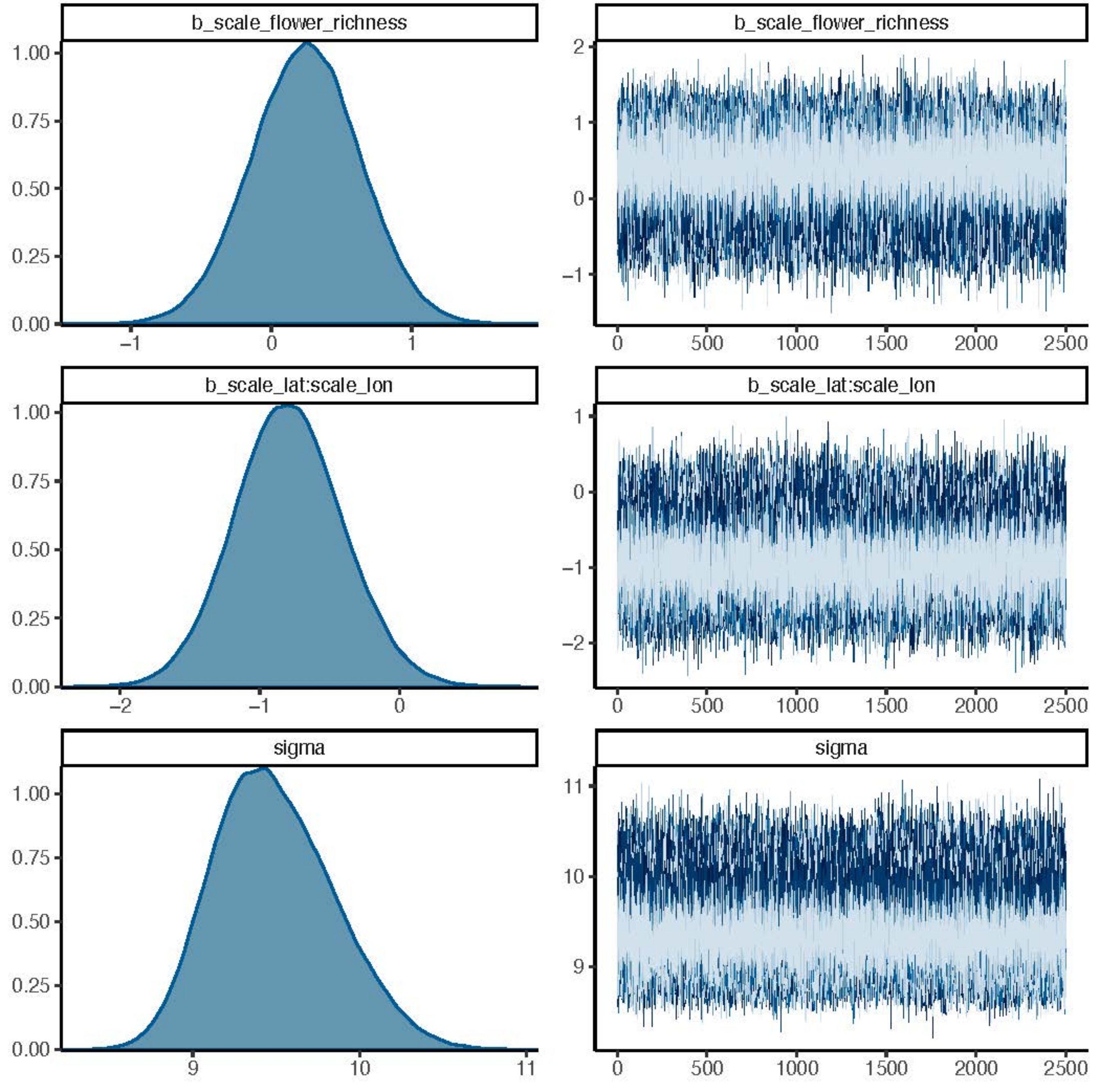
b_scale_flower_richness
b_scale_flower_richness
b_scale_lat:scale_lon
b_scale_lat:scale_lon
sigma
sigma
1.00
0.75
0.50
0.25
0.00
−1
0
1
2
500
1000
1500
2000
2500
−2
9
10
11

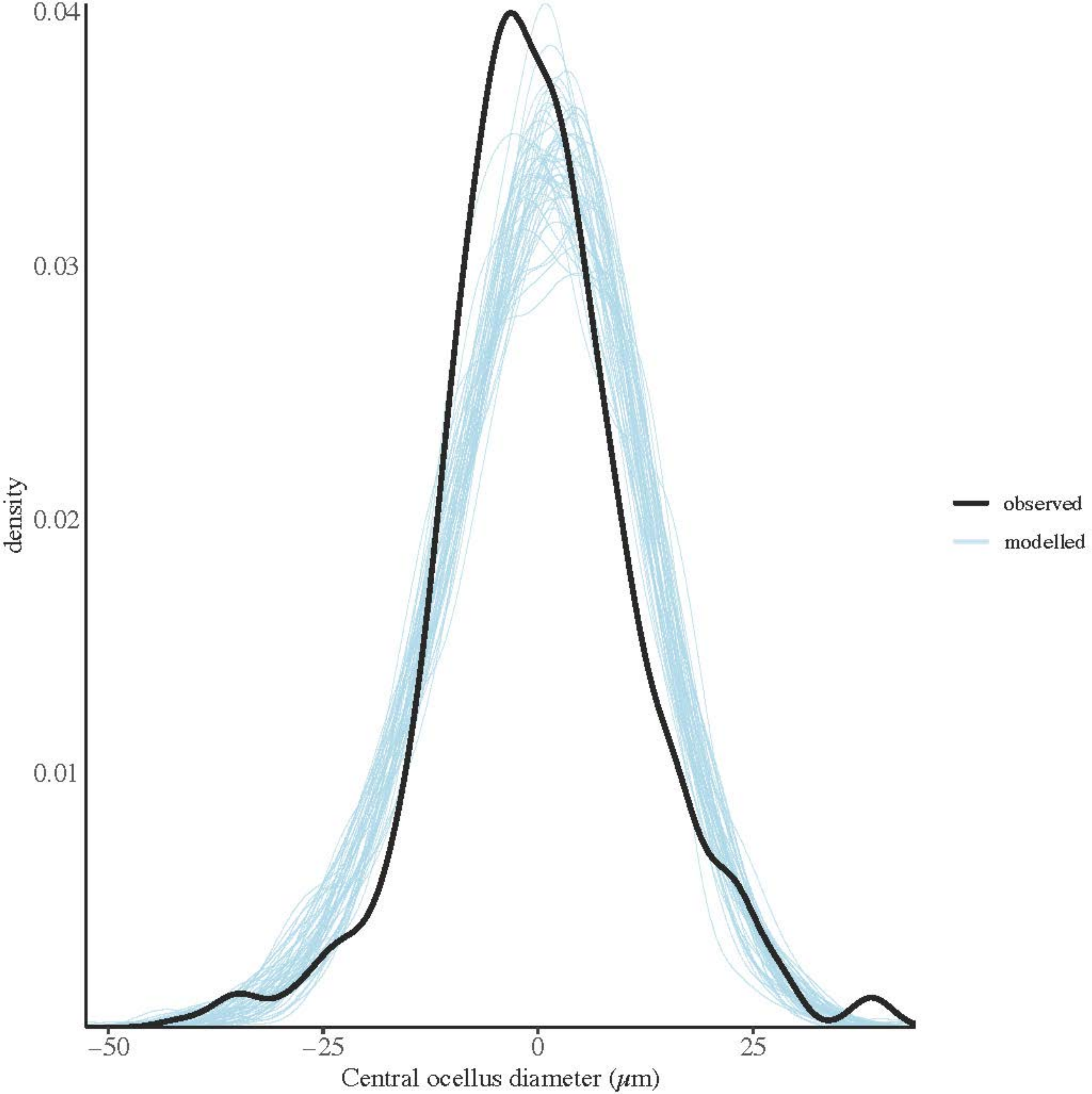
0.04
0.03
0.02
0.01
density
−50
−25
0
25
Central ocellus diameter (μm)
observed
modelled

Lateral ocelli diameter (μm)

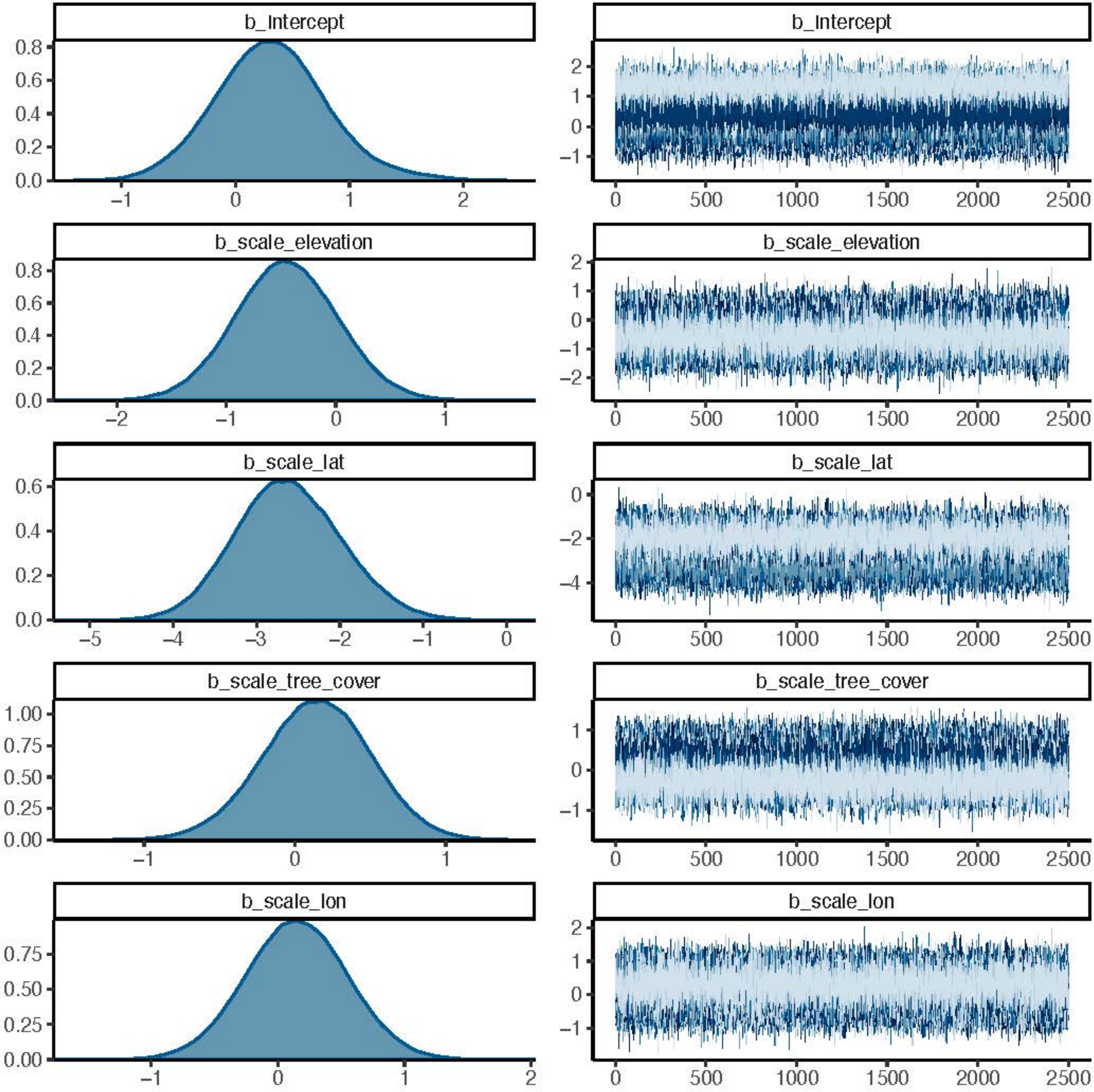

b_Intercept
b_Intercept
b_scale_elevation
b_scale_elevation
b_scale_lat
b_scale_lat
b_scale_tree_cover
b_scale_tree_cover
b_scale_lon
b_scale_lon

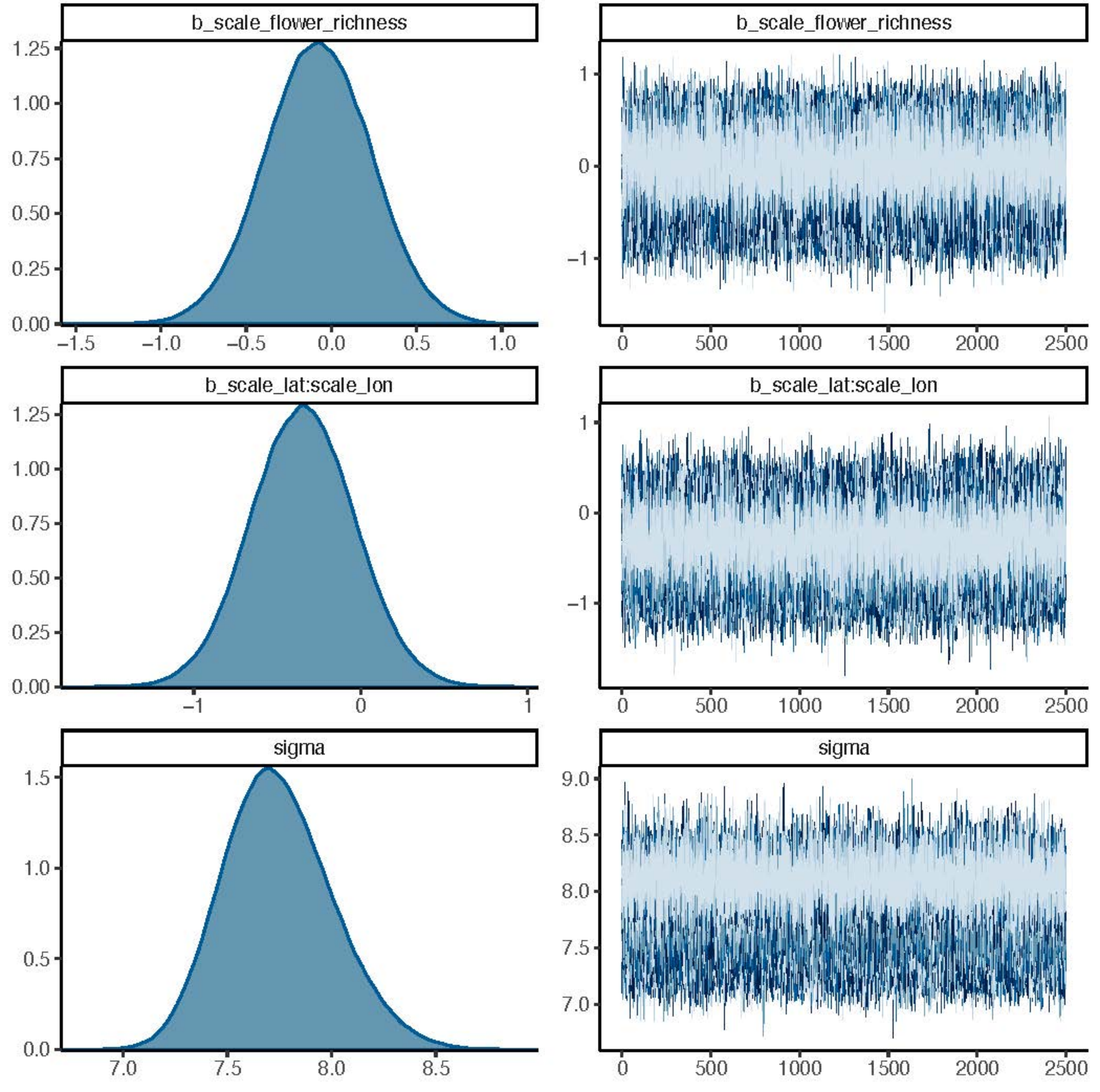

b_scale_flower_richness
b_scale_flower_richness
b_scale_lat:scale_lon
b_scale_lat:scale_lon
sigma
sigma

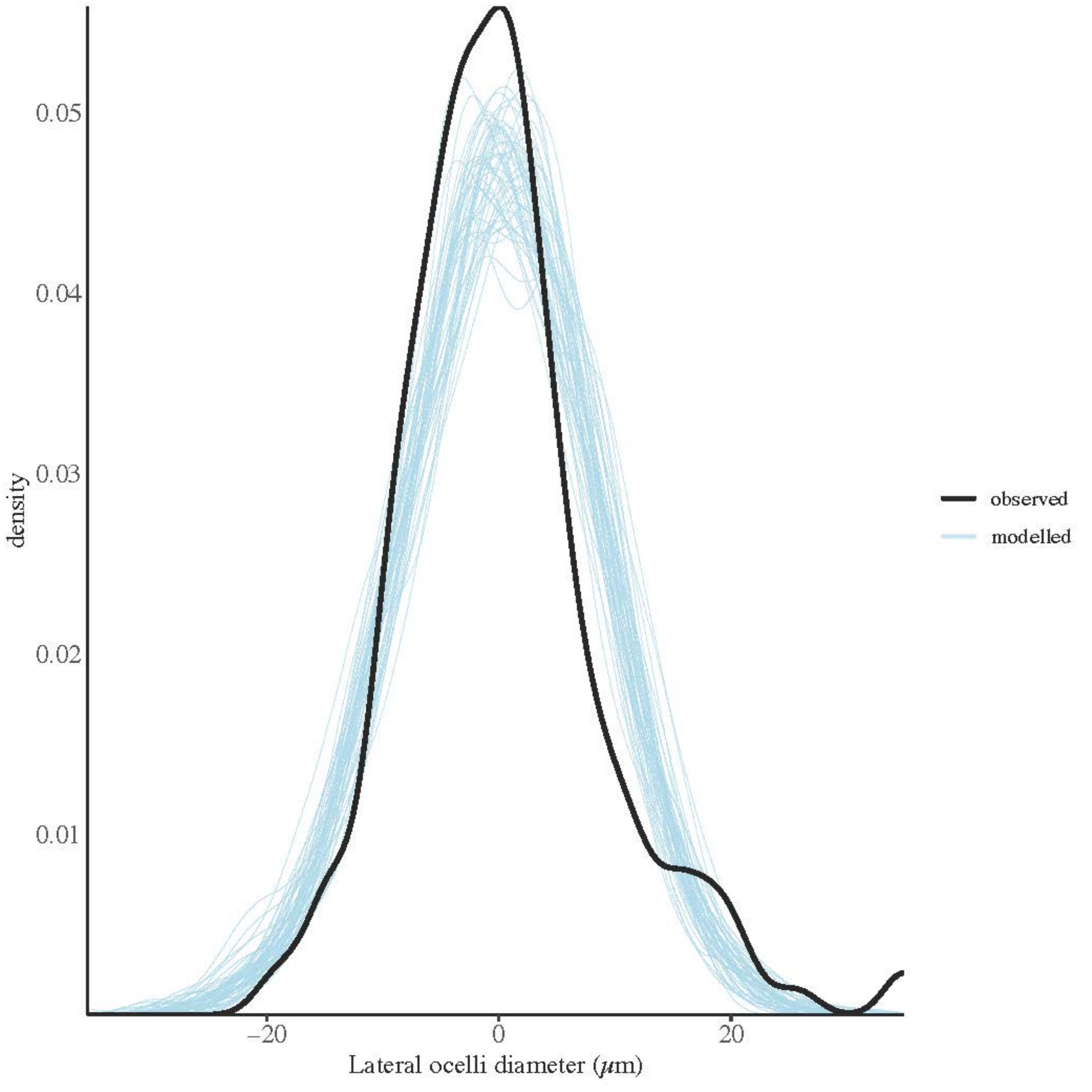
density
0.05
0.04
0.03
0.02
0.01
−20
0
20
Lateral ocelli diameter (μm)
observed
modelled

Ocellar alignment (°)

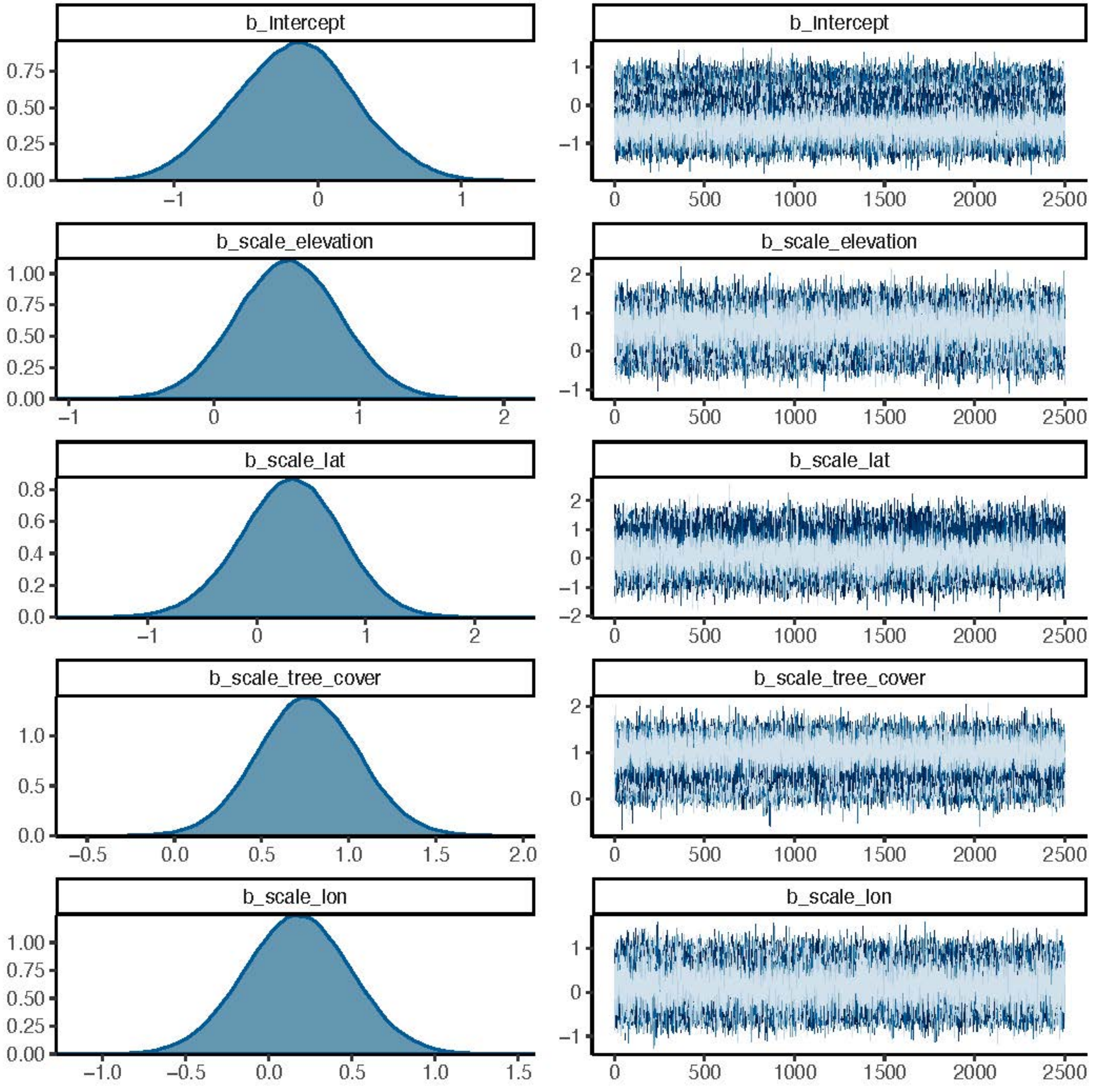
b_Intercept
b_scale_elevation
b_scale_lat
b_scale_tree_cover
b_scale_lon
0.00
0.25
0.50
0.75
1.00
−1
0
1
2
−0.5
0.0
0.5
1.0
1.5
2.0
500
1000
1500
2000
2500

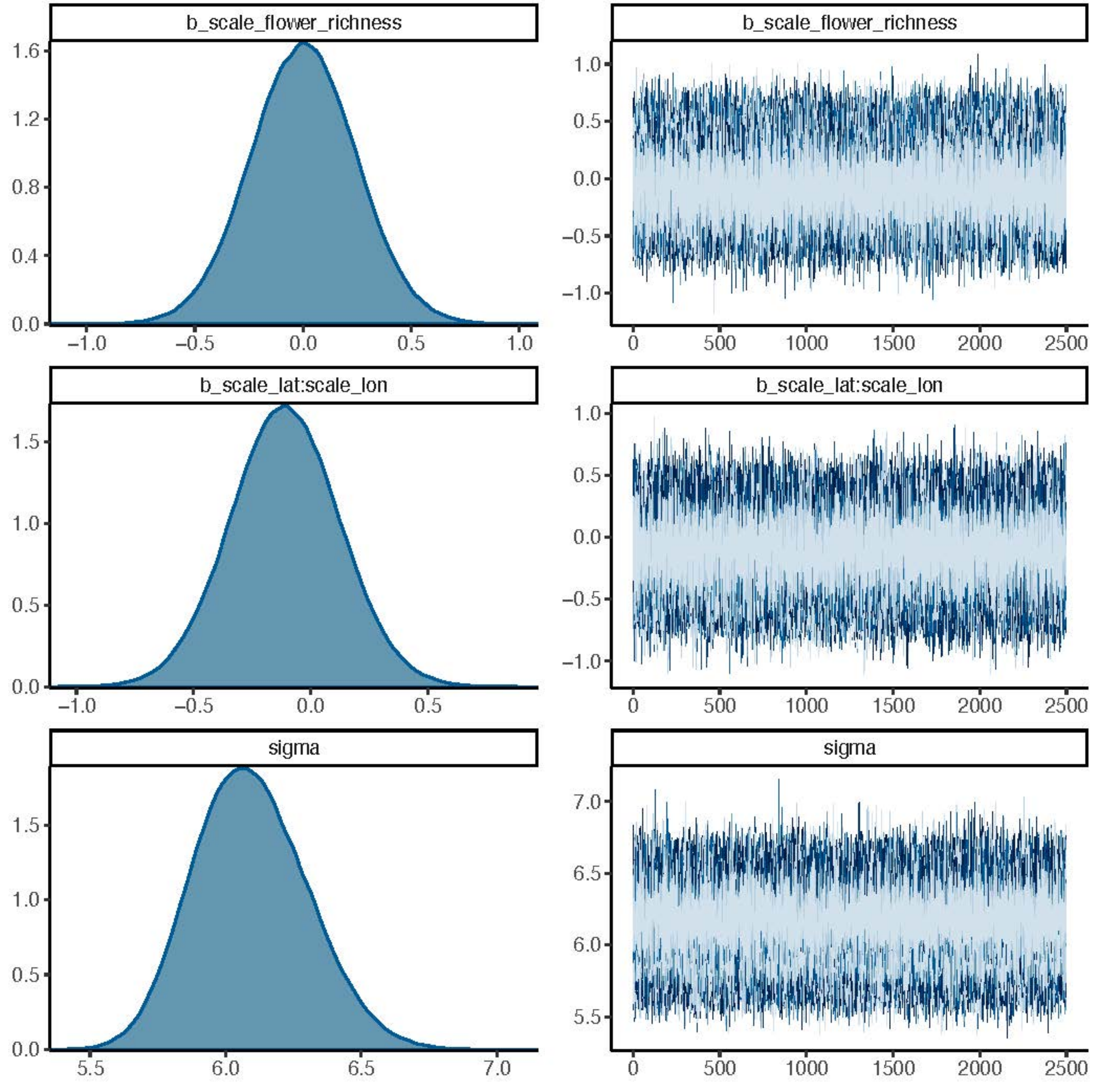

b_scale_flower_richness
b_scale_flower_richness
b_scale_lat:scale_lon
b_scale_lat:scale_lon
sigma
sigma

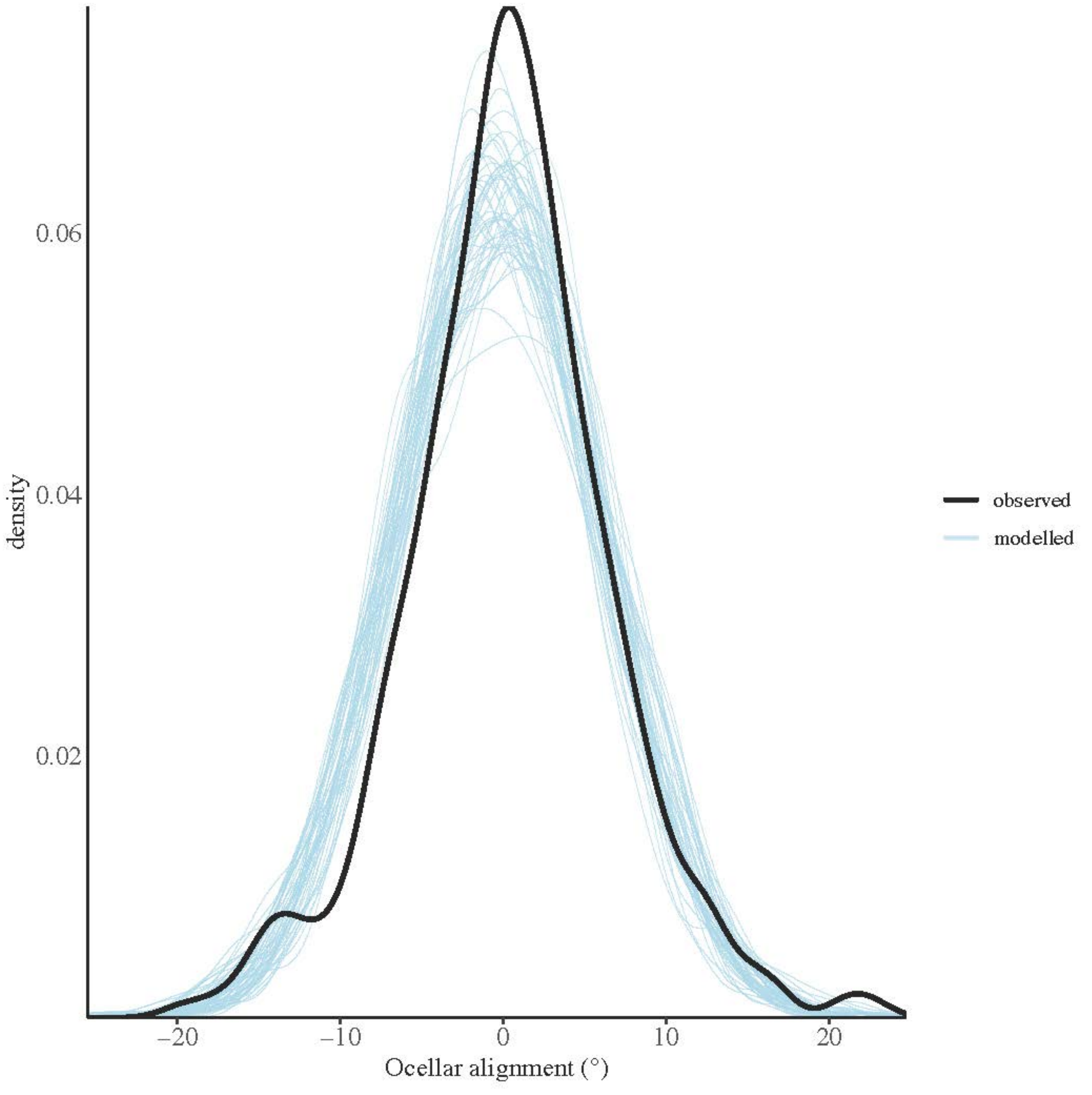

0.06
0.04
0.02
density
−20
−10
0
10
20
Ocellar alignment (°)
observed
modelled

Monocular FOV (%)

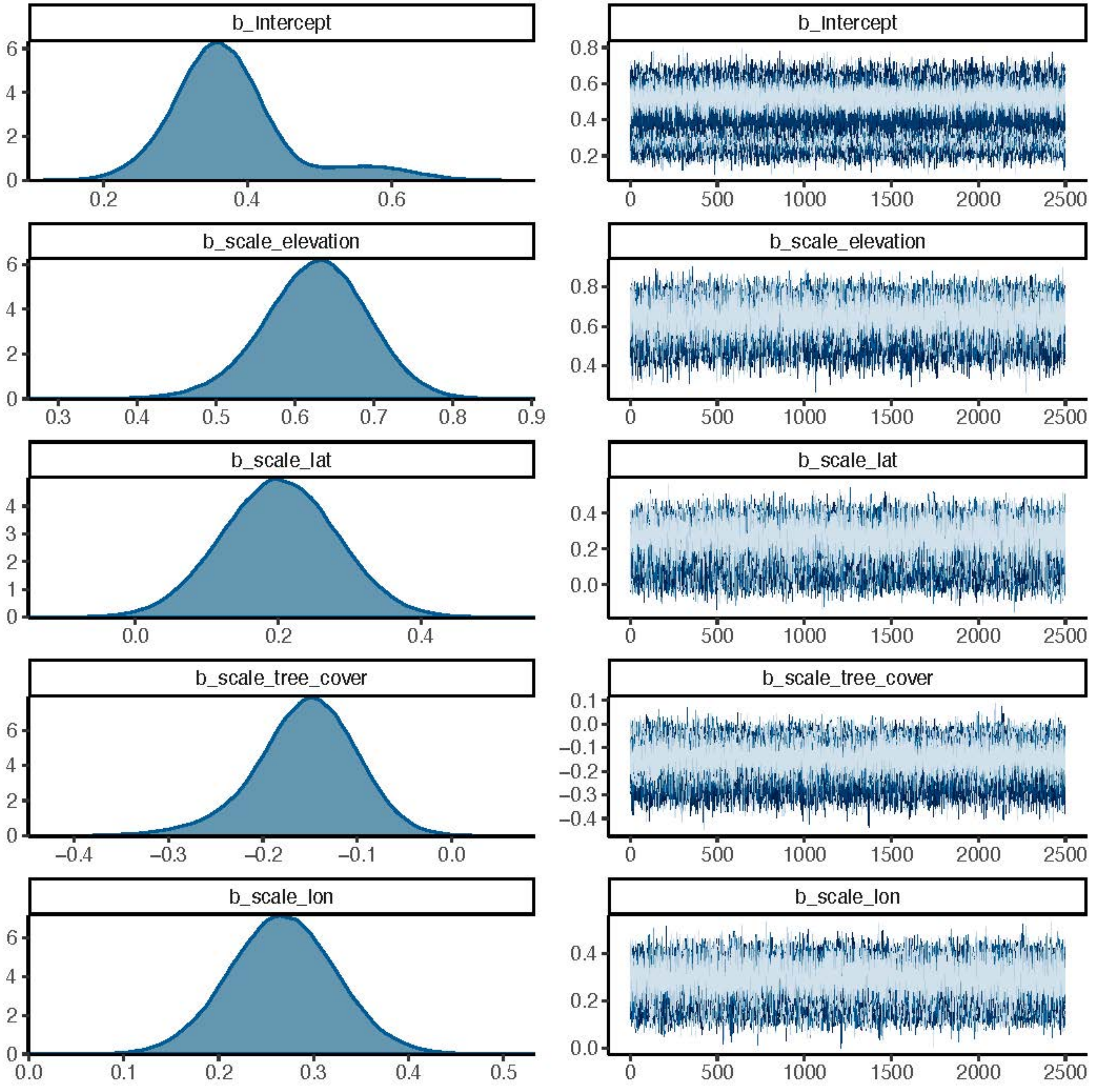
b_Intercept
6
4
2
0
0.2
0.4
0.6
b_Intercept
0.8
0.6
0.4
0.2
0
500
1000
1500
2000
2500
b_scale_elevation
6
4
2
0
0.3
0.4
0.5
0.6
0.7
0.8
0.9
b_scale_elevation
0.8
0.6
0.4
0
500
1000
1500
2000
2500
b_scale_lat
4
3
2
1
0
0.0
0.2
0.4
b_scale_lat
0.4
0.2
0.0
0
500
1000
1500
2000
2500
b_scale_tree_cover
6
4
2
0
−0.4
−0.3
−0.2
−0.1
0.0
b_scale_tree_cover
0.1
0.0
−0.1
−0.2
−0.3
−0.4
0
500
1000
1500
2000
2500
b_scale_lon
6
4
2
0
0.0
0.1
0.2
0.3
0.4
0.5
b_scale_lon
0.4
0.2
0.0
0
500
1000
1500
2000
2500

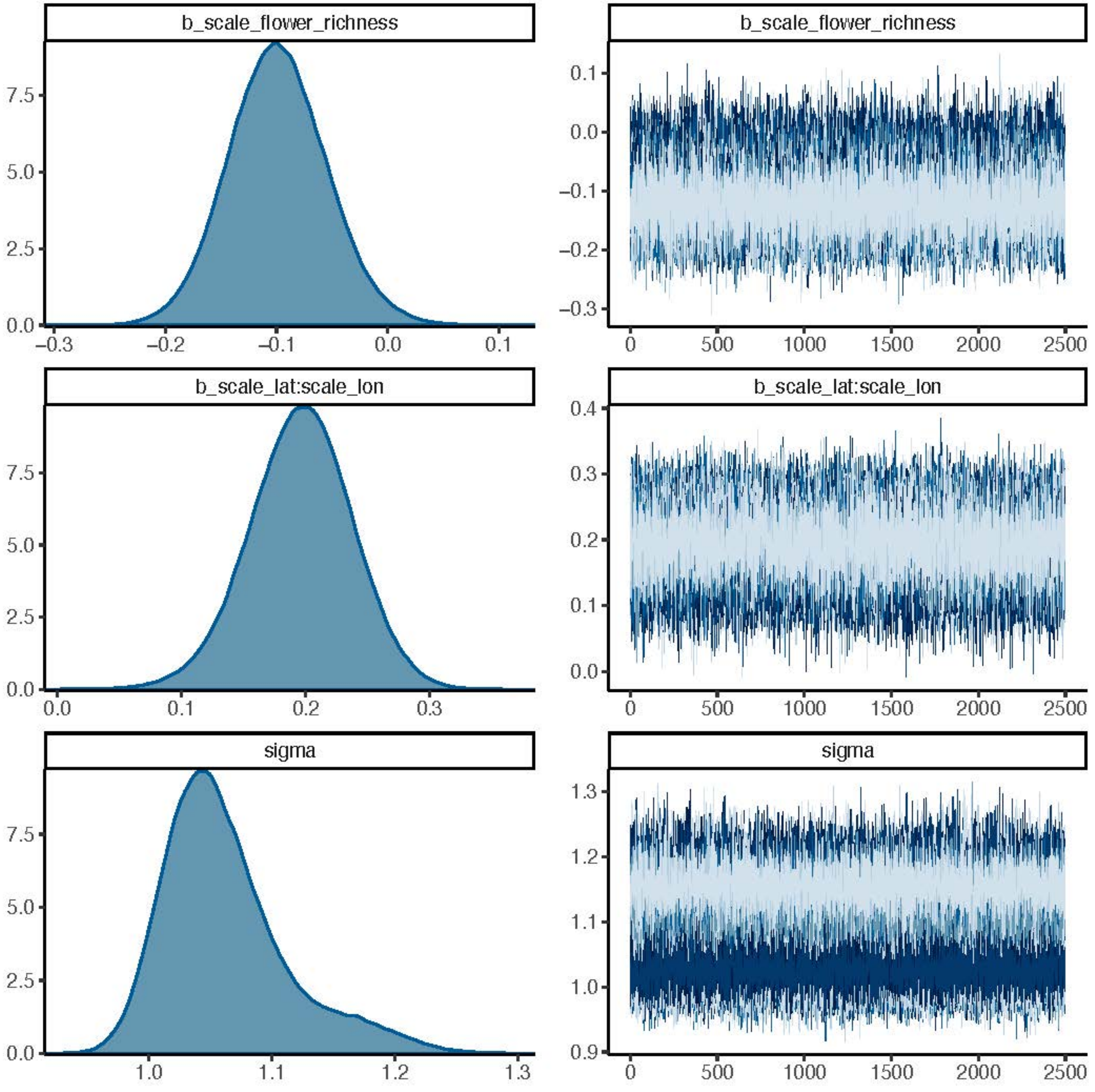

b_scale_flower_richness
b_scale_flower_richness
b_scale_lat:scale_lon
b_scale_lat:scale_lon
sigma
sigma
0.0
2.5
5.0
7.5
−0.3
−0.2
−0.1
0.0
0.1
0
500
1000
1500
2000
2500
0.2
0.3
0.4
1.0
1.1
1.2
1.3
0.9

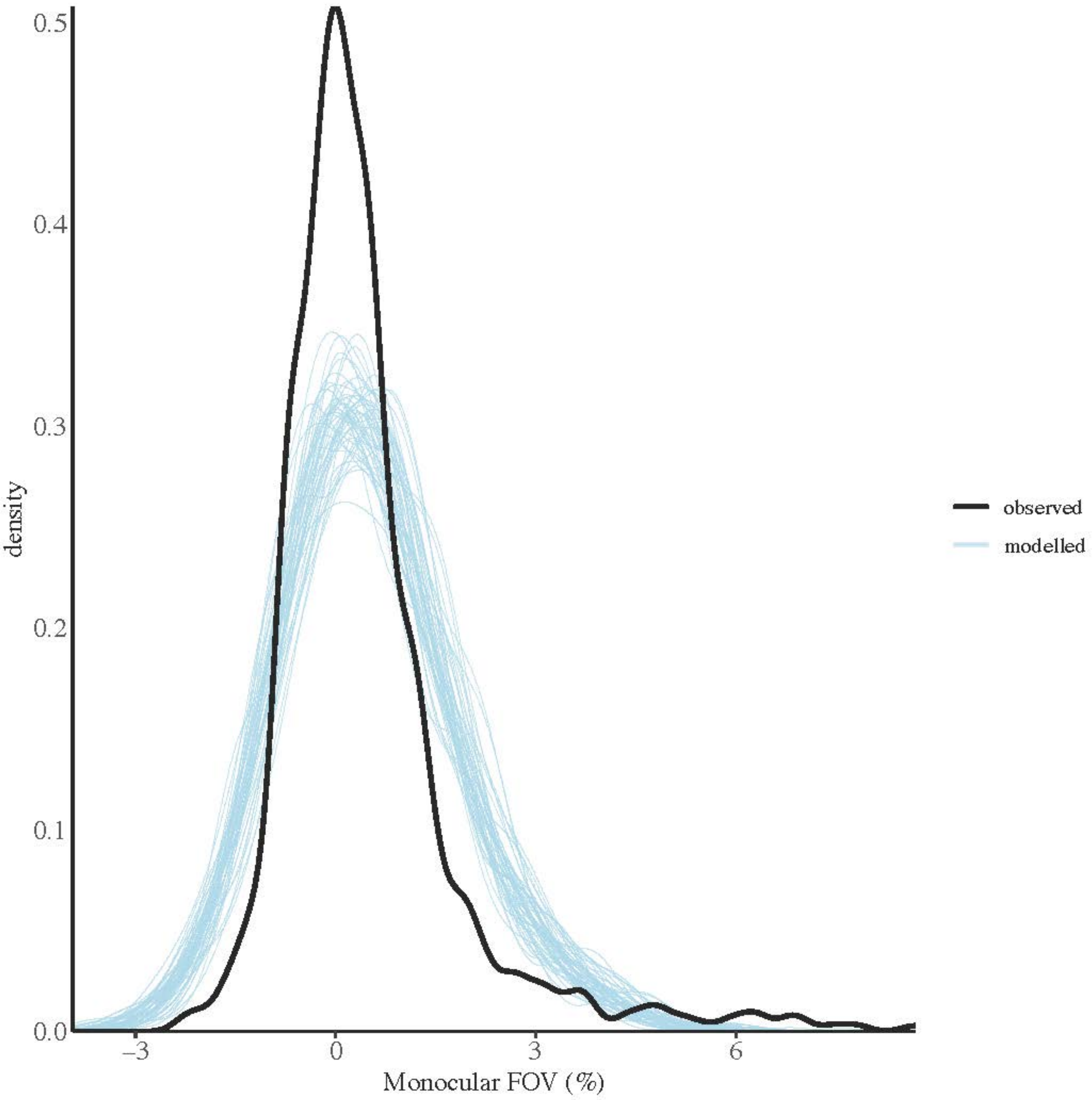
0.5
0.4
0.3
0.2
0.1
0.0
density
−3
0
3
6
Monocular FOV (%)
observed
modelled

Binocular overlap (%)

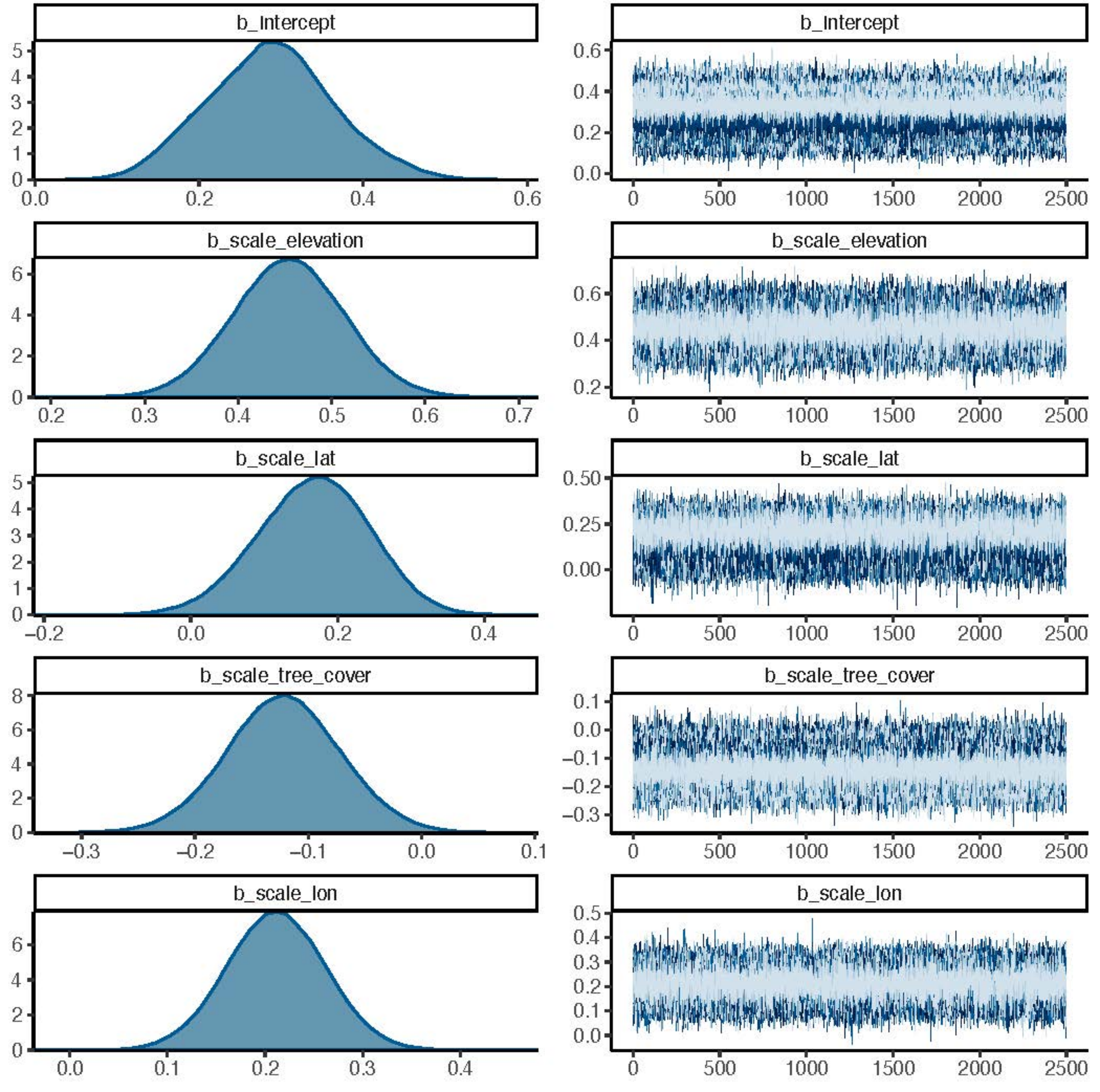

b_Intercept
b_scale_elevation
b_scale_lat
b_scale_tree_cover
b_scale_lon
0
500
1000
1500
2000
2500

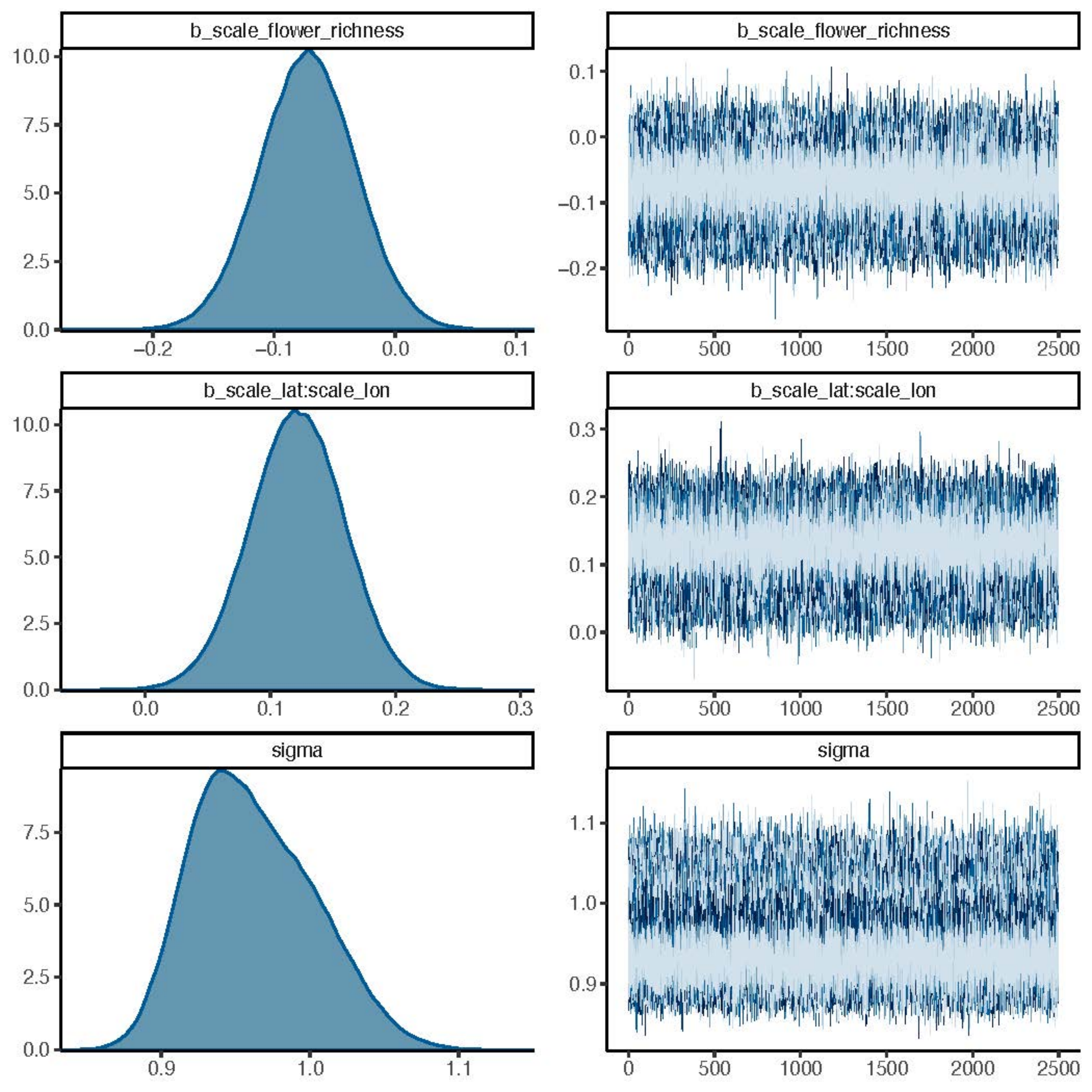
b_scale_flower_richness
b_scale_flower_richness
b_scale_lat:scale_lon
b_scale_lat:scale_lon
sigma
sigma
10.0
7.5
5.0
2.5
0.0
−0.2
−0.1
0.0
0.1
0.1
0.0
−0.1
−0.2
0
500
1000
1500
2000
2500
0.0
0.1
0.2
0.3
0.3
0.2
0.1
0.0
0.9
1.0
1.1
1.1
1.0
0.9

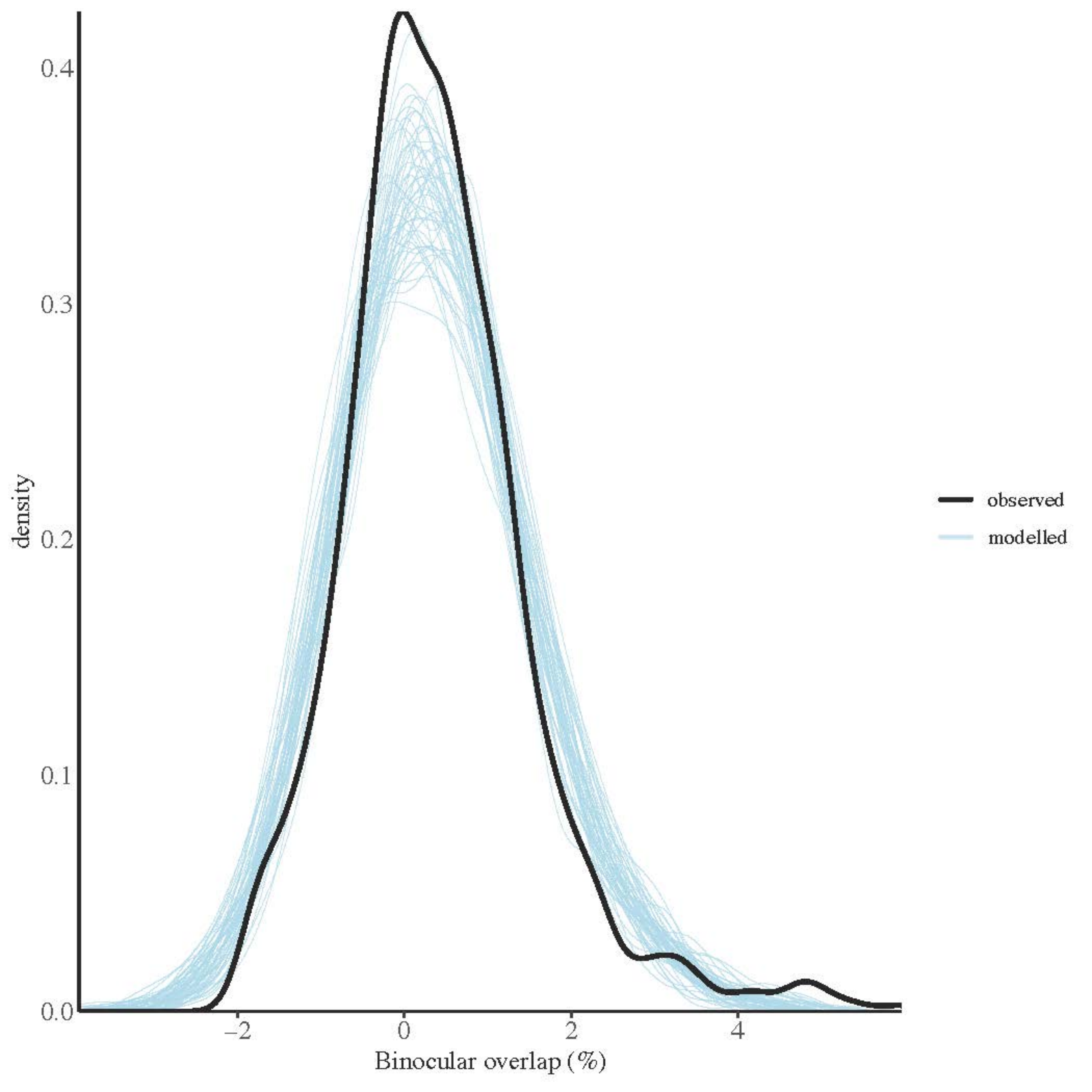

0.4
0.3
0.2
0.1
0.0
density
−2
0
2
4
Binocular overlap (%)
observed
modelled
